# XVI International Symposium on Marine Natural Products|XI European Conference on Marine Natural Products

**DOI:** 10.3390/md18010040

**Published:** 2020-01-06

**Authors:** Rui Pedrosa, Susana P. Gaudêncio, Vitor Vasconcelos

**Affiliations:** 1 MARE—Marine and Environmental Science Centre, Polytechnic of Leiria, 2520-630 Peniche, Portugal; rui.pedrosa@ipleiria.pt; 2 UCIBIO—Applied Molecular Biosciences Unit, Department of Chemistry, Blue Biotechnology and Biomedicine Lab, Faculty for Sciences and Technology, NOVA University of Lisbon, 2829-516 Caparica, Portugal; 3 CIIMAR-Interdisciplinary Centre of Marine and Environmental Research, 4450-208 Matosinhos, Portugal; vmvascon@fc.up.pt; 4 Department of Biology, Faculty of Sciences, Porto University, 4069-007 Porto, Portugal

## Preface

The International Symposium on Marine Natural Products (MaNaPro) happened for the first time in 1975 in the city of Aberdeen, Scotland, organized by Professor Ronald H. Thomson. The European Conference on Marine Natural Products (ECMNP) occurred for the first time in 1997 in Athens, Greece, organized by Professor Vassilios Roussis. The MaNaPro and ECMNP conferences have triennial and biennial frequencies, respectively. Since its first edition, the ECMNP has been set in the alternating years of the Gordon Conferences on Marine Natural Products. In 2019, it was the second time, in 44 years, that a joint organization of the MaNaPro and ECMNP meetings occurred. The first joint meeting of the MaNaPro and the ECMNP occurred in 2013 in Galicia, La Toja, Spain, organized by Dr. Carmen Cuevas from PharmaMar. Over the time, there have been 16 editions of the MaNaPro and 11 editions of the ECMNP held in several countries.

The list of 16 editions of the MaNaPro is shown as following:

I International Symposium on Marine Natural Products, 8–11 September 1975, Aberdeen, Scotland, UK, organized by Ronald H. Thomson;

II International Symposium on Marine Natural Products, 12–15 September 1978, Sorrento, Italy, organized by Mario Piattelli;

III International Symposium on Marine Natural Products, 16–19 September 1980, Brussels, Belgium, organized by Bernard Tursch and Jean-Claude Braekman;

IV International Symposium on Marine Natural Products, 26–30 July 1982, Tenerife, Spain, organized by Antonio G. Gonzáles and Julio D. Martín;

V International Symposium on Marine Natural Products, 2–6 September 1985, Paris, France, organized by Yoel Kashman and R obert H. Dodd;

VI International Symposium on Marine Natural Products, 3–7 July 1989, Dakar, Senegal, organized by Jean-Michel Kornprobst;

VII International Symposium on Marine Natural Products, 5–10 July 1992, Capri, Italy, organized by Luigi Minale;

VIII International Symposium on Marine Natural Products, 10–15 September 1995, Tenerife, Spain, organized by Julio D. Martin;

IX International Symposium on Marine Natural Products, 5–10 July 1998, Townsville, Queensland, Australia, organized by Bruce F. Bowden;

X International Symposium on Marine Natural Products, 24–29 June 2001, Okinawa, Japan, organized by Tatsuo Higa;

XI International Symposium on Marine Natural Products, 4–9 September 2004, Sorrento, Italy, organized by *Ernesto* Fattorusso, Guido Cimino and Raffaele Riccio;

XII International Symposium on Marine Natural Products, 4–9 February 2007, Queenstown, New Zealand, organized by John Blunt and Murray Munro;

XIII International Symposium on Marine Natural Products, 17–22 October 2010, Phuket, Thailand, organized by Khanit Suwanborirux;

XIV International Symposium on Marine Natural Products, 15–20 September 2013, La Toja, Spain, organized by Carmen Cuevas;

XV International Symposium on Marine Natural Products, 29 August–2 September 2016, Fortaleza, Brazil, organized by Leticia V. Costa-Lotufo;

XVI International Symposium on Marine Natural Products, 1–5 September 2019, Peniche, Portugal, organized by Rui Pedrosa, Susana P. Gaudêncio, and Vitor Vasconcelos.

The list of 11 editions of the ECMNP is shown as following:

I European Conference on Marine Natural Products, 2–6 November 1997, Athens, Greece, organized by Vassilios Roussis;

II European Conference on Marine Natural Products, 12–16 September 1999, Santiago de Compostela, Spain, organized by Ricardo Riguera;

III European Conference on Marine Natural Products, 15–20 September 2002, Munich, Germany, organized by Thomas Lindel and Matthias Köck;

IV European Conference on Marine Natural Products, 12–16 September 2005, Paris, France, organized by Marie-Lise Bourguet-Kondracki and Ali Al-Mourabit;

V European Conference on Marine Natural Products, 16–21 September 2007, Naples, Italy, organized by Angelo Fontana;

VI European Conference on Marine Natural Products, 19–23 July 2009, Porto, Portugal, organized by Anake Kijjoa;

VII European Conference on Marine Natural Products, 14–18 August 2011, Strömstad, Sweden, organized by Lars Bohlin;

VIII European Conference on Marine Natural Products, 15–20 September 2013, La Toja, Spain, organized by Carmen Cuevas;

IX European Conference on Marine Natural Products, 30 August–3 September 2015, Glasgow, UK, organized by Ruangelie Edrada-Ebel.

X European Conference on Marine Natural Products, 3–7 September 2017, Crete, Greece, organized by Efstathia Ioannou and Vassilios Roussis.

XI European Conference on Marine Natural Products, 1–5 September 2019, Peniche, Portugal, organized by Rui Pedrosa, Susana P. Gaudêncio, and Vitor Vasconcelos.

The International Board of Directors for the MaNaPro meetings consists of Professor William Fenical from the Scripps Institution of Oceanography (SIO), UCSD, USA, Professor Angelo Fontana from the Institute of Biomolecular Chemistry (ICB), Italy, Dr. Carmen Cuevas from PharmaMar, Spain, Professor Jörn Piel from the Institute of Microbiology, ETH Zurich, Zwitzerland, Professor John Blunt from the University of Canterbury, New Zealand, Professor Marcel Jaspars from the University of Aberdeen, UK, Professor Murray Munro from the University of Canterbury, New Zealand, Professor Ronald Quinn from the Eskitis Institute, Griffith University, Australia, Professor Shigeki Matsunaga from the University of Tokyo, Japan, and Professor Tadeusz Molinski from the Skaggs School of Pharmacy and Pharmaceutical Sciences, UCSD, USA. As for the European counterpart, the European Board of Directors for the ECMNP meetings includes Professor Anake Kijoa from the University of Porto, Portugal, Professor Ali Al Mourabit from the Centre National de la Recherche Scientifique (CNRS), France, Professor Angelo Fontana from the Institute of Biomolecular Chemistry (ICB), Italy, Professor Efstathia Ioannou from the National and Kapodistrian University of Athens, Greece, Professor Marie-Lise Bourguet-Kondracki from the Centre National de la Recherche Scientifique (CNRS), France, Professor Matthias Köck from the Alfred Wegener Institute, Germany, Professor Ruangelie Edrada-Ebel from the University of Strathclyde, UK, and Professor Vassilios Roussis from the National and Kapodistrian University of Athens, Greece.

**Figure 1:**
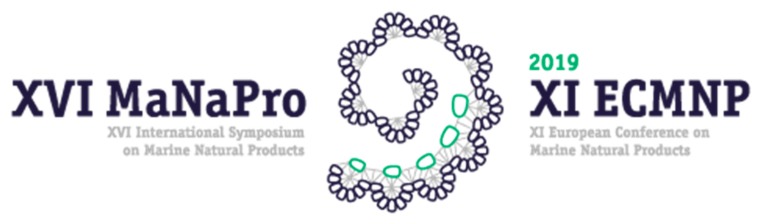
Logotype of the joint XVI MaNaPro and XI ECMNP meeting, 2019.

The MaNaPro and ECMNP meetings have been very successful, with a primary focus on studies related to marine chemistry. The MaNaPro symposia remain the world’s most noteworthy events in this scientific field. The ECMNP arose from the acknowledgment that, mostly due to financial reasons, young European scientists were not able to participate in major international conferences on marine natural products. It is the ECMNP’s main goal to encourage younger researchers’ awareness in the fascinating interdisciplinary fields of marine natural products research and at the same time promote collaborations around the world. Both the meetings are self-financed. The first three editions of the ECMNP were supported by the 5th European Framework Program and the Training and Mobility of Researchers uniquely. In 2019, the joint XVI MaNaPro and XI ECMNP meeting held in Peniche on the 1st to the 5th of September was supported by the financial services of the Polytechnic of Leiria and the sponsorships of several companies. Visit Portugal and PharmaMar were diamond sponsors, and the Royal Society of Chemistry, Hovione, and *Marine Drugs* were gold sponsors. *Marine Drugs* was also the conference scientific publisher. The Royal Society of Chemistry also supported the conference by offering free access to MarinLit during its duration. The conference had the support of many different partners. *Molecules* was the conference media partner, assisting in publicizing the conference. The Oeste Intermunicipal Community, the Oeste Portugal, the Peniche Municipality, the Batalha Monastary, the Alcobaça Monastery, Dinutri, and the FCT/MCTES supported the conference’s rich cultural program. To honor the Portuguese cultural Heritage, the logotype of the 2019 MaNaPro & ECMNP conference edition (Figure 1) was inspired by the Peniche traditional handcraft bobbin lace, representing a marine organism. It was created by the designers of the School of Arts and Design from Polytechnic of Leiria, which was also a conference partner. The conference handbags, which incorporated Portuguese cork, were designed by Polytechnic of Leiria.

The 2019 edition also counted with the high patronage of the President of the Portuguese Republic.

The 2019 conference was jointly organized by three Portuguese research institutes working in marine biotechnology, the MARE—Marine and Environmental Science Centre from the Polytechnic of Leiria, the UCIBIO–Applied Molecular Biosciences Unit from the Faculty of Sciences and Technology of the NOVA University of Lisbon, and the CIIMAR—Interdisciplinary Centre of Marine and Environmental Research from the University of Porto. The conference chairs and organizers, Professor Rui Pedrosa, Dr. Susana P. Gaudêncio, and Professor Vitor Vasconcelos, selected a scientific committee for the joint MaNaPro and ECMNP meeting that included Professor William Fenical, Professor Vassilios Roussis, Professor Leticia Costa-Lotufo, Professor Angelo Fontana, and Professor Matthias Köck. 

The conference in Peniche welcomed 298 participants from both industry and academia, working not only in the field of marine natural product chemistry, but also pharmacology, microbiology, biology, molecular biology, biotechnology, and ecology, amongst others, from 31 countries in five continents with scientists from Australia, Brasil, Canada, Chile, China, Croatia, Ecuador, Egypt, France, Germany, Ghana, Greece, Ireland, Israel, Italy, Japan, Mexico, The Netherlands, New Zealand, Nigeria, Portugal, Russia, Saudi Arabia, Singapore, South Africa, South Korea, Spain, Switzerland, Tunisia, the United Kingdom, and the United States of America.

In this year’s conference, we had the participation of 111 students (PhD, MSc, and others), which was 37% of the overall attendance, including postdoctoral researchers, early career researchers, and senior researchers, as well as the participation of industry experts from Europe and the USA, which included PharmaMar, Biosortia, APIVITA SA, Hovione, Sea4Us, Natura Cosmético, and Pierre Guerin SAS. There were 129 participants with age below 35 years old, which occupied 43% of the overall attendance. The female attendance was 48% of the overall attendance.

The 2019 joint XVI MaNaPro and XI ECMNP meeting had six sessions addressing the scientific themes: 1. marine chemical ecology and ecosystem functioning; 2. isolation and structure elucidation of marine natural products; 3. drug discovery and development; 4. biosynthesis of marine natural products; 5. marine natural products synthesis and medicinal chemistry; and 6. marine natural products chemical biology and biotechnology.

A total of 241 abstracts were included in the conference book of abstracts. The scientific program was divided in 14 plenary lectures, 36 selected oral communications, and 191 poster communications, which this conference report comprised. We had plenary speakers from both industry and academia. The six sessions were chaired by leading scientists in their respective fields: Session 1 on marine chemical ecology and ecosystem functioning was chaired by Professor Gabriele M. König from the University of Bonn; Session 2 on isolation and structure elucidation of marine natural products was chaired by Professor Ruangelie Edrada-Ebel from the University of Strathclyde; Session 3 on drug discovery and development was chaired by Dr. Carmen Cuevas from PharmaMar; Session 4 on biosynthesis of marine natural products was chaired by Professor Matthew Crump from the University of Bristol; Session 5 on marine natural products synthesis and medicinal chemistry was chaired by Professor Daniel Romo from Baylor University; and Session 6 on marine natural products chemical biology and biotechnology was chaired by Professor Norberto P. Lopes, from University of São Paulo.

The first day of the conference was marked by an open ceremony and two keynote speakers, Professor William Fenical and Professor Vassilios Roussis, in the representation of the MaNaPro and ECMNP conferences, respectively. In the following days, each session of the conference had two 35 min plenary lectures, one at the beginning of the session and the other one at the end of the session. There were six selected 10 min oral communications for each session. One of these was exclusive for PhD students, with the attribution of an award that covered the conference registration and accommodation expenses. This was in line with the objectives of the ECMNP in promoting marine natural products research to a younger generation of scientists.

There were three poster sessions: the first including Sessions 1 and 2, with the scientific themes of marine chemical ecology and ecosystem functioning and isolation and structure elucidation of marine natural products, respectively; the second for Sessions 3 and 4, with the themes of drug discovery and development and biosynthesis of marine natural products, respectively; and the third for Sessions 5 and 6, with the themes of marine natural products synthesis and medicinal chemistry and marine natural products chemical biology and biotechnology, respectively.

Professor William Fenical from the University of California San Diego gave the first opening plenary lecture highlighting the importance of integrating chemistry and biology, using genomics to support structure elucidation and absolute configuration assignments. He provided an example of the complete absolute structure assignment of neaumycin B, a complex (19 chiral centers) marine-derived actinomycete metabolite, appointed as an area of frontier research on the knowledge of structures and biosynthesis of plant and invertebrate-derived metabolites. 

Professor Vassilios Roussis from the National and Kapodistrian University of Athens provided a detailed revision of marine biopolymers for biomedical applications. He presented a case study of ulvan and copolymers extracted from green algae *Ulva rigida* for the production of bio-based materials with medical and biotechnological applications, emphasizing the importance of the research on biopolymers extracted from marine resources. 

The first plenary lecture was presented by Professor Eric Schmidt from the University of Utah. To the delight of the audience, he presented genomic and biochemical studies applied to marine invertebrates, specifically to marine mollusks and their symbiotic relations with bacteria. He showed that there are convergent biosynthetic routes for the production of bioactive secondary metabolites in mollusks.

Professor Georg Pohnert from Friedrich Schiller University presented chemical signals responsible for algal–algal, diatom–bacteria, and diatom–diatom interactions that structure the marine environment. He provided a particular example of zwitterionic metabolites from microalgae, which regulate metabolic interactions and contribute to the global sulfur cycling. 

Professor Jon Clardy from Harvard Medical School focused his presentation on structure elucidation of challenging metabolites. He underlined the importance of unveiling the metabolites modes of action in addition to their biosynthetic pathways and structure elucidation. 

Professor Angelo Fontana from the Consiglio Nazionale delle Ricerche, Institut of Biomolecular Chemistry (BOCU) described the implementation of a screening platform for the selection of immunomodulatory compounds. He presented the research that was conducted for the discovery of a new class of immunomodulators from marine protists, named SULFAVANTs.

Dr. José María Fernández from PharmaMar stunned the marine natural products community with the fantastic research story of plitidepsin (Aplidin^®^), a cyclodepsipeptide originally isolated from the marine tunicate *Aplidium albicans*, recently approved in Australia as a first-in-class drug for the treatment of multiple myeloma. The PharmaMar work reinforces the premise that marine natural products will play a key role in providing new drugs in future years.

Dr. Guy T. Carter from Biosortia Pharmaceuticals explored the aquatic microbiomes for drug discovery. He described the genomic and metabolomics analysis of harmful algal blooms, dominated by *Planktothrix* cyanobacteria from Grand Lake St. Marys State Park, reporting the discovery of a novel class of metabolites, cyanobufalins, which are cardiotonic steroid toxins. 

Professor Bradley S. Moore from the University of California, San Diego emphasized the importance of genomics in the discovery of bioactive marine natural products. His presentation focused on the development of genetic platforms and methods that advanced the natural product biosynthesis and synthetic biology fields, highlighting their use in marine bacteria, invertebrates, and algae. 

Dr. Pedro N. Leão from the CIIMAR of the University of Porto provided an overview of biosynthetic pathways associated with the incorporation of fatty acids into cyanobacterial secondary metabolites. He showed examples of unprecedented fatty acid modifications.

Professor Emmanuel A. Theodorakis from the University of California San Diego presented synthetic methodologies to attain the total syntheses of bacterial-inspired antitumor and antibiotic spirotetronate polyketides. He showed the synthesis of class I spirotetronates and the strategies to pursue the synthesis of classe II spirotetronates.

Professor Tobias A. M. Gulder from the Technical University of Dresden presented biocatalytic total synthesis strategies, inspired in marine natural products biosynthetic concepts, to achieve complex marine microbial metabolites. As examples, he unveiled the total syntheses of sorbicillinoids and ikarugamycin. 

Professor Leticia V. Costa-Lotufo from the Instituto de Ciências Biomédicas, USP proposed a reverse affinity methodology to verify the interaction of marine natural products, by loading resins with two categories of cellular proteins that play important roles in tumoral carcinogenesis, proliferation, and chemoresistance. She obtained prodigiosin derivatives as promising modulating agents. 

The plenary lectures culminated with the one presented by Professor Jaclyn M. Winter from the University of Utah College of Pharmacy. She brilliantly demonstrated the use of genomic approaches to supporting the discovery of two new classes of metabolites, bonnevillamides and salinipeptins, isolated from actinomycetes collected from the Great Salt Lake. 

The joint XVI MaNaPro and XI ECMNP meeting was very successful and met the objectives of both the MaNaPro and the ECMNP conferences by providing high-quality research to the scientific community and promoting marine natural products research to younger generations of scientists, who will lead the future investigation into marine chemistry and its associate fields in ecology, molecular biology, pharmacology, microbiology, and biotechnology. In fact, the expectations of the participating members of the Board of Directors for the MaNaPro and the ECMNP meetings, and the participants in general were exceeded. The chairs and organizers of the 2019 joint MaNaPro and ECMNP conference earned a great deal of praise and compliments from both the Board of Directors and the participants for the excellent scientific program and the successful cultural program, as well as for the outstanding organization.

### ***Acknowledgments*** 

We would like to thank all the members of the local organizing committee for their amazing work and support, particularly to Susana Mendes from MARE-IPLeiria for her enormous support in all the steps of the conference organization, conference secretariat, and web design. This conference was funded by the financial services of the Polytechnic of Leiria. This work was supported by the Applied Molecular Biosciences Unit—the UCIBIO, which is financed by national funds from FCT/MCTES (UID/Multi/04378/2019). Thanks are given to the CIIMAR, which is financed by the FCT/MCTES project UID/Multi/04423/2019. S.P.G. is grateful to FCT/MCTES for the project IF/00700/2014.

### ***Conflicts of Interest*** 

The authors declare no conflict of interest.

## Session 1: Plenary Speakers Abstracts

### ***Opening Lectures*** 

#### **Exploiting Where Chemistry and Biology Collide** 

FenicalWilliamCo-WorkersCenter for Marine Biotechnology and Biomedicinen, Scripps Institution of Oceanography, University of California, San Diego, La Jolla, CA 92093-0204, USA

As time passes, it is becoming more and more clear that natural products chemistry is linked to specific gene clusters that code for the production of terpenes, polyketides, alkaloids and other classes of natural products. This is where chemistry and biology intersect to create some exciting integrated science. Consequently, the study of natural products chemistry can no longer be viewed as a simple chemistry enterprise. These revelations have been recognized in microorganisms where the size of their genomes and the ability to locate gene clusters can be readily exploited due to many years of gene cluster sequence data. Obviously, combining genomics and gene cluster sequence information can greatly assist in understanding molecular structures and how they are formed. Unfortunately, this effective approach has yet to be fully applied to marine plants and invertebrates. Consequently, knowledge of the structures and biosynthesis of plant and invertebrate-derived molecules is an area of frontier research. A brief example of the complete absolute structure assignment of a complex (19 chiral centers) marine bacterial metabolite using knowledge relying on biosynthetic enzyme specificities illustrates this point.

#### **The Big Ones Revisited: Marine Biopolymers for Biomedical Applications** 

TzivelekaLeto-AikateriniKikionisStefanosIoannouEfstathiaRoussisVassiliosSection of Pharmacognosy and Chemistry of Natural Products, Department of Pharmacy, National and Kapodistrian University of Athens, 15771 Athens, Greece

Natural polymers, due to their inherent unique properties of biocompatibility and biodegradability, are highly appreciated as valuable ingredients of biomaterials and therefore are widely exploited in the biomedical field. Polysaccharides, incorporating various functionalities in their structures and exhibiting interesting physicochemical properties and significant biological activities, are considered attractive materials for the development of novel systems for bioapplications, such as drug delivery and tissue engineering. In this context, natural carbohydrates are increasingly utilized either in their native form or after chemical modification, so as to deliver tailor-made materials for specific applications. Marine algae produce polysaccharides that are regarded as safer and non-immunogenic, in contrast to those of animal origin. Ulvan, a sulphated polysaccharide located in the cell walls of green algae that possesses unique structural properties albeit its repeating unit shares chemical affinity with glycosoaminoglycans, such as hyaluronan and chondroitin sulphate, has been increasingly studied over the years for applications in the pharmaceutical field. The physicochemical properties and pharmacological activity of ulvan have been systematically investigated and a wide range of biological activities have been reported. Fibrous biocomposites comprising ulvan extracted from *Ulva rigida* and a number of copolymers were successfully prepared using the electrospinning technique. Moreover, novel hybrid scaffolds based on chemically cross-linked ulvan revealing highly porous interconnected structures were prepared, characterized and used as substrates for cell cultures. Our current results, in combination with the inherent cytocompatibility of ulvan, highlight the potential uses of the prepared ulvan-based biomaterials either as novel formulations for peptide/protein and/or drug delivery systems or as prospective scaffolds in tissue engineering.

### ***Theme 1. Chemical Ecology and Ecosystem Functioning*** 

#### **Biosynthesis and Ecology of Metabolites in the Marine Mollusks** 

SchmidtEric W.Department of Medicinal Chemistry, 307 Skaggs Hall, 30 South 2000 East, Salt Lake City, UT 84112-5820, USA

Marine mollusks use small molecules for chemical defense, for offense and predation, and as signaling hormones. Classical precursor feeding studies demonstrate that mollusks obtain many compounds from their diets, while others are made within the animals themselves. Here, we use genomic and biochemical techniques to investigate the origin of different classes of metabolites made within mollusks. We show that mollusk symbiotic bacteria often make defensive metabolites found within the organisms. Animal enzymes also synthesize a somewhat surprising array of diverse and complex metabolites. Thus, there are convergent routes to active secondary metabolites in mollusks.

#### **Exploration of Chemical Signals from Marine Microalgae that Structure the Marine Environment** 

PohnertGeorgDepartment of Bioorganic Analytics, Friedrich Schiller University, Lessingstr. 8, D-07749 Jena, Germany

Unicellular algae in the plankton and in biofilms have established means to interact with other organisms in their environment. Especially interactions mediated by chemical compounds have gained a lot of attention during the last years. Algal exudates and metabolites stored in the cells can mediate feeding activity of herbivores and algal/algal interactions but also communication of an alga with the surrounding microbial community. We introduce an approach to address such chemically mediated interactions based on the metabolomic investigation of the cellular and released metabolites of microalgae. These surveys are based on elaborate mass spectrometric methods and reveal that algae exhibit a high plasticity of metabolite production during their development. Bioassays that are guided by metabolomic evidence reveal that these specific compounds can mediate mate finding, chemical defense or algal/algal interactions in a highly dynamic manner. We extend this concept also to the highly complex chemistry of zwitterionic metabolites involved in osmoregulation as well as chemical interactions (see Figure 2). These metabolites not only regulate an intricate network of metabolic interactions but also contribute substantially to global sulfur cycling. Examples of diatom bacteria interactions and of diatom-diatom interactions will be introduced. Consequences for future investigations of plankton ecology, algal physiology and chemical interactions are discussed.

References

1. Thume, K.; Gebser, B.; Chen, L.; Meyer, N.; Kieber, D.; Pohnert, G. The metabolite dimethylsulfoxonium propionate extends the marine organosulfur cycle. *Nature*
**2018**, *563*, 412–415.

### ***Theme 2. Isolation and Structure Elucidation of Marine Natural Products*** 

#### **Elusive Microbial Metabolites** 

ClardyJonHarvard Medical School, Boston, MA 02115, USA

Today there so many ways to find and characterize bacterial and fungal metabolites that it seems unlikely that any could elude discovery and characterization. But some do, and this talk will be several recent examples from my laboratory. They will include metabolites that play key roles in electron shuttling, chemical defenses, unusual strains, and some that are hiding in plain sight. The talk will deal with structure elucidation of challenging metabolites including some new NMR approaches, structures that emerged from new biosynthetic sources, and metabolites whose structures cannot be remotely inferred from genetic information.

#### **Natural Products of Marine Protists—From Basic Research to Novel Immunimodulatory Agents** 

FontanaAngeloManzoEmilianoNuzzoGenoveffaGalloCarmelaLandiSimoned’IppolitoGiulianaConsiglio Nazionale delle Ricerche, Istitute of Biomolecular Chemistry, Bio-Organic Chemistry Unit (BOCU), Via Campi Flegrei 34, 80078 Pozzuoli, Napoli, Italy

In the last years, the activity of our group has focused on the development of biotechnological products based on marine metabolism, including processes (for example, production of biohydrogen or novel bio-based methods for CO_2_ mitigation) and functional ingredients for cosmetic or nutraceutical applications [1,2]. However, our core activities remain anchored to the chemistry and biochemistry of natural products in the context of basic studies (chemoecology, biosynthesis, physiology) or applied research (early drug discovery) [3–8]. In this frame, we have been implementing a screening platform for selection of immunomodulatory compounds as potential therapeutic agents in cancer, infectious and neurodegenerative diseases. Here, I summarize the main results obtained working with marine protists. The presentation draws the progression of these studies and focuses on the development of a new class of immunomodulators (collectively named SULFAVANTs, Figure 3) that trigger the immune response by unconventional activation of Antigen Presenting Cells [9,10].

References

1. D’Ippolito, G.; Dipasquale, L., Fontana, A. Recycling of Carbon Dioxide and Acetate as Lactic Acid by the Hydrogen-Producing Bacterium *Thermotoga neapolitana*. *ChemSusChem*
**2014**, *7*, 2678–2683.
2. Vella, F.M.; Sardo, A.; Gallo, C.; Landi, S.; Fontana, A.; d’Ippolito, G. Annual outdoor cultivation of the diatom *Thalassiosira weissflogii*: productivity, limits and perspectives. *Algal Res.*
**2019**, *42*, 101553.
3. Gallo, C.; d’Ippolito, G.; Nuzzo, G., Sardo, A.; Fontana, A. Autoinhibitory sterol sulfates mediate programmed cell death in a bloom-forming marine diatom. *Nat. Commun.*
**2017**, *8*, 1292.
4. Ruocco, N.; Costantini, S.; Zupo, V.; Lauritano, C.; Caramiello, D.; Ianora, A.; Budillon, A.; Romano, G.; Nuzzo, G.; d’Ippolito, G.; et al. Toxigenic effects of two benthic diatoms upon grazing activity of the sea urchin: morphological, metabolomic and *de novo* transcriptomic analysis. *Sci. Rep.*
**2018**, *8*, 5622.
5. Adelfi, M.G.; Vitale R.M., d’Ippolito, G.; Nuzzo, G.; Gallo, C.; Amodeo, P.; Manzo, E.; Pagano, D.; Landi, S.; Picariello, G.; et al. Patatin-like lipolytic acyl hydrolases and galactolipid metabolism in marine diatoms of the genus *Pseudo-nitzschiaBiochim. Biophys. Acta—Mol. Cell Biol. Lipids*
**2019**, *1864*, 181–190.
6. Nuzzo, G.; De Araújo Gomes, B.; Gallo, C.; Amodeo, P.; Sansone, C.; Pessoa, O.D.L.; Manzo, E.; Vitale, R.M.; Ianora, A.; Santos, E.A.; et al. Potent Cytotoxic Analogs of Amphidinolides from the Atlantic Octocoral *Stragulum bicolor. Mar. Drugs*
**2019**, *17*, 58.
7. Cutignano, A.; Nuzzo, G.; Ianora, A.; Luongo, E.; Romano, G.; Gallo, C.; Sansone, C.; Aprea, S.; Mancini, F.; D’Oro, U.; et al. Development and Application of a Novel SPE-Method for Bioassay-Guided Fractionation of Marine Extracts. *Mar. Drugs*
**2015**, *13*, 5736–5749.
8. Barra, G.; Lepore, A.; Gagliardi, M.; Somma, D.; Matarazzo, M.R.; Costabile, F.; Pasquale, G.; Mazzoni, A.; Gallo, C.; Nuzzo, G.; et al. Sphingosine Kinases promote IL-17 expression in human T lymphocytes. *Sci. Rep.*
**2018**, *8*, 13233.
9. Manzo, E.; Gallo, C.; Fioretto, L.; Nuzzo, G.; Barra, G.; Pagano, D.; Krauss, I.R.; Paduano, L.; Ziaco, M.; Dellagreca, M.; et al. Diasteroselective Colloidal Self-Assembly Affects the Immunological Response of the Molecular Adjuvant Sulfavant. *ACS Omega*
**2019**, *4*, 7807–7814.
10. Manzo, E.; Cutignano, A.; Pagano, D.; Gallo, C.; Barra, G.; Nuzzo, G.; Sansone, C.; Ianora, A.; Urbanek, K.; Fenoglio, D.; et al. A new marine-derived sulfoglycolipid triggers dendritic cell activation and immune adjuvant response. *Sci. Rep.*
**2017**, *7*, 6286.

### ***Theme 3. Drug Discovery and Development Nobody is a Prophet in His Own Land. The Aplidin^®^ Story*** 

FernándezJosé MaríaPharmaMar, S. A., 28770-Colmenar Viejo, Madrid, Spain

Multiple myeloma (MM) is a cancer that begins in plasma cells. Normal plasma cells are white blood cells, which are found in the bone marrow and form a key part of the body’s immune system, producing the antibodies necessary for fighting infections. Abnormal plasma cells produce a type of antibody that does not benefit the body and can accumulate, thus preventing normal cells from functioning properly.

In 2016, there were about 130,000 cases of multiple myeloma worldwide and 100,000 deaths. In 2015, 26,850 new cases were diagnosed in the US, and about 11,200 people died from this still incurable disease. In Europe, the incidence is 4.5–6.0 out of 100,000 diagnosed per year. Plitidepsin (Aplidin) is a cyclodepsipeptide originally isolated from the marine tunicate Aplidium albicans that is now manufactured by total chemical synthesis. It is a first-in-class drug that binds to a protein called EF1A2, specifically targeting the non-canonical role of protein EF1A2, resulting in tumor cell death via apoptosis (programed death). Plitidepsin has received orphan drug designation in the European Union and the United States of America.

Plitidepsin has been first approved in Australia for the treatment of patients that relapse after three lines of treatment, including proteasome or cereblon inhibitors (immune modulators). It can also be administered as 3rd line treatment, when the patient has already received two prior lines and is refractory or intolerant to proteasome inhibitors or immune modulators. This first approval by the Australian regulatory authority (TGA), opens the door to many other markets in South America, Mexico, Canada, Asia Pacific, Middle East and North Africa, among others, who will review Aplidin^®^ following TGA’s decision, and where PharmaMar has partners for this product.

#### **Mega-Metabolomic Exploration of Aquatic Microbiomes for Drug Discovery** 

CarterGuy T.Biosortia Pharmaceuticals, Inc., 3210 Merryfield Row, JLABS SUITE 5900, San Diego, CA 92121, USA

Aquatic microbiomes contain an amazing array of microbial constituents, depending on ecological and environmental conditions. Genomic analyses of aquatic microorganisms provide evidence that highly diverse biosynthetic machinery is present. This biosynthetic capacity typically has little obvious impact on the surrounding environment, since the production of metabolites is repressed or is at such low concentration that no overt biological effects are observed. Under “bloom” conditions, however, the production of secondary metabolites can soar. Fresh water systems are increasingly susceptible to HAB events, and Grand Lake St. Marys in Ohio (GLSM) is a case in point. Owing to the recurrent, potent HAB events in GLSM, this lake has been used as the primary test site for the development of Biosortia’s harvesting technologies. Investigation of the metabolomes of several eutrophic fresh water systems across the United States has revealed unique chemistry that has been captured for screening. Biosortia employs a “deep-dive” approach to uncover novel biologically active chemistry, facilitated by our proprietary harvesting technologies.

A water treatment facility in Muskegon, Michigan, dominated by the cyanobacterium *Microcystis aeruginosa*, proved to be an exceptionally rich source of novel chemistry. Several of the metabolites contained serially-homologated tyrosine residues as characteristic features. In the case of microcystin 1107, the homologated residues confer significantly enhanced potency against cancer cell lines [1] (Figure 4).

The meta-metabolome of GLSM blooms that are dominated by *Planktothrix* cyanobacteria has been shown to contain several typical cyanotoxins, including mircrocystins and saxitoxin. However, we have recently discovered a novel class of toxins related to cardio-active steroids that we named cyanobufalins [2] (Figure 4). Although cyanobufalins contain distinctive structural features that distinguish them from plant- or animal-derived congeners, the compounds are qualitatively identical to bufalin in their biological profiles. This is the first time that cardiotonic steroid toxins have been found associated with microorganisms in an aquatic environment.

References

1. He, H.; Wu, S.; Wahome, P.G.; Bertin, M.J.; Pedone, A.C.; Beauchesne, K.R.; Moeller, P.D.R.; Carter, G.T. Microcystins Containing Doubly Homologated Tyrosine Residues from a Microcystis aeruginosa Bloom: Structures and Cytotoxicity. *J. Nat. Prod.*
**2018**, *81*, 1368–1375.
2. He, H.; Wu, S.; Bertin, M.J.; Wahome, P.G.; Beauchesne, K.R.; Youngs, R.O.; Zimba, P.V.; Moeller, P.D.R.; Carter, G.T. Cyanobufalins: Cardioactive Toxins from Cyanobacterial Blooms. *J. Nat. Prod.*
**2018**, *81*, 2576–2581.

### ***Theme 4. Biosynthesis of Marine Natural Products*** 

#### **Emerging Platforms and Opportunities in Marine Natural Product Biosynthesis** 

MooreBradley S.Scripps Institution of Oceanography & Skaggs School of Pharmacy and Pharmaceutical Sciences, University of California San Diego, La Jolla, CA 92093, USA

Marine organisms continue to amaze for their capacity to produce chemically unique and biologically important small molecules. Marine natural products mediate key interactions between ocean dwelling organisms, have improved human health as medicines, and helped illuminate many foundational cellular processes germane to life. Despite the remarkably diverse biological origins and chemical structures of marine natural product molecules, they all have one thing in common—they are all DNA-encoded chemicals. With advances in DNA sequencing, the ability to connect genes to chemistry and function has transformed many fields of science that study the chemistry of the sea and its inhabitants. This presentation will focus on the development of genetic platforms that have enabled the rapid growth in natural product biosynthesis and synthetic biology, highlighting recent examples specific to the marine realm. Methods such as genome mining, gene cluster editing, and heterologous expression will be discussed in relation to bioactive marine natural products from bacteria, invertebrates, and algae studied in the Moore laboratory at UC San Diego.

#### **Fatty Acid Incorporation into Cyanobacterial Secondary Metabolites** 

LeãoPedro N.Interdisciplinary Centre of Marine and Environmental Research (CIIMAR/CIMAR), University of Porto, Terminal de Cruzeiros do Porto de Leixoes, Av. General Norton de Matos s/n, 4450-208 Matosinhos, Portugal

Cyanobacteria have a differentiated lifestyle among bacteria, which is reflected in the unique structures of many of their secondary metabolites. One peculiarity of cyanobacterial natural products is the common presence of fatty acid moieties. The incorporation and modification of fatty acids in cyanobacterial secondary metabolism entails rich biological chemistry. Examples include the halogenation of alkyl moieties, or C-C bond forming reactions between two fatty acid precursors. Cyanobacterial genomes contain a large number of biosynthetic gene clusters predicted to encode fatty acid incorporating or -modifying enzymes. Most of these gene clusters have no associated natural product. Likewise, the structures of several natural products from cyanobacteria are reminiscent of fatty acid incorporation or modification events that are of unclear biosynthetic nature. As such, there are abundant opportunities for the discovery of natural products and enzymes by studying how fatty acids are transformed by these organisms.

In this talk, I will provide an overview of the biosynthetic events associated with activating, incorporating and modifying fatty acids into cyanobacterial secondary metabolites. Gene clusters that likely encode new fatty-acid derived secondary metabolites will be highlighted. Furthermore, we will provide examples from our research of unprecedented fatty acid modifications, including C-C and C-O bond forming enzymes. Finally, we will put forward an hypothesis that potentially explains why cyanobacteria tend to divert fatty acids into their secondary metabolism.

### ***Theme 5. Synthesis of Marine Natural Products and Medicinal Chemistry*** 

#### **Synthetic Strategies toward Spirotetronate polyketides** 

BraddockAlexander A.ZhaoNanTheodorakisEmmanuel A.Department of Chemistry & Biochemistry, University of California San Diego, La Jolla, CA 92093-0358, USA

Spirotetronates are bacterial metabolites that possess an architecturally intriguing structure and promising chemotherapeutic properties as antitumor antibiotics [1,2]. These compounds are structurally identified by the presence of a tetronic acid spiro-linked to a cyclohexene (or a cyclohexane) ring (Figure 5a, red motif). Spirotetronates can be further divided into two sub-classes: class I, which contain the spirotetronate motif integrated within a macrocycle and class II, which also contain an integrated decalin (Figure 5a, blue motif) and frequently, oligosaccharide chains attached to the decalin and/or the macrocycle (Figure 5a, green circle). Class I spirotetronates, such as abyssomicins, have succumbed to laboratory synthesis that, in turn, has accelerated mode-of-action studies [1,2]. On the other hand, class II spirotetronates still remain unmet synthetic challenges. We will present our efforts to develop a modular synthesis of these compounds that may expedite mode-of-action studies. 

References

1. Lacoske, H.M.; Theodorakis, E.A. Spirotetronate polyketides as leads in drug discovery. *J. Nat. Prod.*
**2015**, *78*, 562–575.
2. Braddock, A.A.; Theodorakis, E.A. Marine Spirotetronates; Biosynthetic Edifices that Inspire Drug Discovery. *Mar. Drugs*
**2019**, *17*, 232.

#### **Biocatalytic Total Synthesis of Complex Marine Microbial Metabolites** 

GulderTobias A. M.Chair of Technical Biochemistry, Technical University of Dresden, Dresden, 01069, Germany

Marine microbial natural products are an important source of novel chemical scaffolds for biomedical applications. Many of these compounds are encoded by large, multi-enzyme biosynthetic machineries, in particular polyketide synthases (PKS), non-ribosomal peptide synthetases (NRPS), as well as mixed PKS/NRPS systems.

The flexibility of PKS and NRPS systems to assemble small organic building blocks and further structurally alter the resulting biosynthetic intermediates in order to shape complex secondary metabolites results in an inexhaustible diversity of natural product structures and functions. Their pronounced biomedical potential, often combined with tremendous architectural complexity, makes marine natural products attractive targets in total synthesis.

We are particularly interested in elucidating unusual natural strategies yielding complex, polycyclic natural products and in applying the underlying biosynthetic concepts to structurally manipulate such chemical scaffolds by biosynthetic engineering and chemo-enzymatic approaches. We particularly apply late-stage biosynthetic tailoring enzymes that introduce molecular complexity in key steps of biocatalytic routes to our target molecules. In this talk, strategies for the streamlined chemo-enzymatic synthesis of microbial marine natural products and new structural analogs will be presented.

### ***Theme 6. Marine Natural Products Chemical Biology and Biotechnology*** 

#### **Targeting Cancer Relevant Proteins with Marine Natural Products** 

Costa-LotufoLeticia V.CollaboratorsDepartamento de Farmacologia, Instituto de Ciências Biomédicas, University of São Paulo, São Paulo 05508-000, Brazil

The search of novel targeted therapies designed to interfere with a specific molecular target(s) thought to play an integral role in tumorigenesis is recognized as important strategy to the development of new drugs. In our screening program, the proposed therapeutic targets include two categories of cellular proteins: inhibitory apoptosis proteins (survivin, livin and XIAP), and the transcription factor TBX2, both playing important roles in carcinogenesis, proliferation and chemoresistance of various tumors. Here, we resort to a targeted approach based on a specialized reverse-affinity procedure, to verify the interaction of marine natural products to the target and bound compounds were analyzed by LC-MS. This assessment is followed by affinity studies and by in vitro assays to measure protein expression in tumor and nontumor cells lines and whether cytotoxicity can be linked to protein expression levels, and downstream pathways modulation. Considering TBX2 protein, DNA-binding agents chromomycins A 5 (CA 5) and A 6 (CA 6), cosmomycin D and doxorubicin were assayed as potential ligands. CA 5 and doxorubicin were significantly recovered from their respective protein-loaded resins. Microscale thermophoresis analysis showed a K D in the M range for CA 5, CA 6 and doxorubicin while reaching values above 400 M for cosmomycin D. Cytotoxicity analysis showed significant activity against TBX2-driven breast cancer, melanoma, non-small lung carcinoma and rhabdomyosarcoma for all compounds tested, and CA 5 was consistently more active, with IC 50 values in the nM range. Although, a significant correlation between TBX2 protein expression and IC 50 value was not reached among the assessed cell lines, those with higher expression levels seemed especially sensitive to CA 5. The present data suggest that CA 5 is a putative modulator of the transcription factor TBX2 in cancer cells. The use of survivin as a target brought prodiginines as promising modulating compounds. We have isolated prodigiosin (PG), cyclononylprodigiosine (CNP) and nonylprodigiosine (NP) from marine bacteria extracts. These compounds showed affinity to survivin in a fluorescent based assay. IC 50 values showed a weak, but significative, negative correlation with survivin relative expression in cancer cells. These compounds were able to modulate survivin expression in both naïve and vemurafenibe- resistant melanoma cells, inducing apoptosis of treated cells.

##### **Acknowledgments** 

The authors thank FAPESP (2018/08400-1; 2019/02008-5; 2015/17177-6; 2014/50926-0) and CNPQ (INCTBioNat).

#### **Exploring the Chemical Potential of Great Salt Lake Microorganisms** 

WinterJaclyn M.College of Pharmacy, University of Utah, 30 S 2000 E, SKH 307 Salt Lake City, UT 84112-5820, USA

Biological pressures can influence the chemical diversity of secondary metabolites and microorganisms isolated from extreme environments have proven to ideal resources for drug discovery efforts and for characterizing novel biosynthetic pathways. The Great Salt Lake, also recognized as America’s Dead Sea, is an endorheic hypersaline lake in Utah. Recently, we started a natural product drug discovery program aimed at interrogating halophilic bacteria isolated from this unique environment. From our initial isolation campaign, we successfully isolated ten new actinomycetes and sequenced and assembled all ten genomes. Genome mining revealed a wealth of biosynthetic clusters in the Great Salt Lake actinomycetes that have little similarity to other biosynthetic clusters in public databases. Fermentation studies with two of these strains led to the isolation and elucidation of the bonnevillamides and salinipeptins, which are new classes of linear nonribosomal heptapeptides and ribosomally synthesized and post-translationally modified peptides, respectively. The bonnevillamides and salinipeptins contain unprecedented amino acid building blocks and the discovery and characterization of these new chemical entities, as well as their corresponding biosynthetic machinery will be discussed.

## Session 2: Abstracts Selected for Oral Presentations

### ***Theme 1. Chemical Ecology and Ecosystem Functioning*** 

#### **Exploration of Deep-Water Sponges yields new Sponge Species, Novel Microbiomes and Potentially New Bioactive Compounds** 

SchuppPeter J.^1^SteinertGeorg^1^KellermannMatthias^1^ErpenbeckDirk^2^DohrmannMartin^2^BushKathrin^3^WörheideGert^2^HentschelUte^3^1Institute for Chemistry and Biology of the Marine Environment, Carl-von-Ossietzky University, Oldenburg, Schleusenstr. 1, 26382 Wilhelmshaven, Germany2Department of Earth and Environmental Sciences, Ludwig-Maximilians-Universität München, Richard-Wagner-Straße 10, 80333 München, Germany3Marine Microbiology, GEOMAR Helmholtz Centre for Ocean Research Kiel, Düsternbrooker Weg 20, 24105 Kiel, Germany

Sponges (phylum Porifera) are in many marine habitats a main component of sessile benthic communities with more than 8500 described species. They fulfill important ecological functions and are a key player in benthic pelagic coupling. Sponge holobionts harbor often symbiotic association of microorganisms, which are often very diverse and complex. While there have been numerous studies on the microbial diversity of sponge associated bacteria, most studies focused on temperate and tropical shallow water environments. This has resulted in a lack of data on sponge microbial associations from deep-water sponges, especially hexactinellid or glass sponges, as this group of sponges is almost exclusively found in the deep-sea at depth below 400 m.

Here we present first data of sponges which were collected with the ROV (ROV Kiel 6000) during the cruise SO254 (aboard RV Sonne). The aim of the expedition was to assess the biodiversity of the deep-water fauna, which included sea cucumbers, octocorals and sponges. The second aim was to investigate the associated microorganisms and marine natural products from the various macroorganisms. A total of 219 sponges specimens belonging to 111 sponge (Porifera) OTUs were collected from depths down to 4800 m. About half of the collected sponges belonged to the Demospongiae, the others were Hexactinellida. A total of 600 bacteria were purified from these sponges. So far Proteobacteria, Chloroflexi, Actinobacteria, Bacteroidetes, Acidobacteria are the dominating phyla from the isolates. Initial tests of bacterial isolates and sponge extracts showed various antimicrobial bioactivities and indicated potential novel marine natural products.

#### **Mapping Seaweed and Seagrass Surfaces by Untargeted Metabolomics and DESI-Mass Spectrometry Imaging Integrated with Microbiome and Bioassay Studies** 

TasdemirDenizGEOMAR Centre for Marine Biotechnology, Research Unit Marine Natural Products Chemistry, GEOMAR Helmholtz Centre for Ocean Research Kiel, Kiel, 24106, Germany

The surfaces are crucial in overall health and biotic interactions of marine organisms. However, they are prone to rapid microbial colonization leading to biofilm formation and fouling. Seaweeds protect their surfaces by mechanical as well as chemical methods by exuding secondary metabolites (SMs) onto their surfaces. Due to methodological difficulties, however, our understanding on surface chemistry and surface-based hostmicrobe interactions are limited. This presentation addresses this issue on two organisms, the brown seaweed *Fucus vesiculosus* (*FV*) and the eelgrass *Zostera marina* (*ZM*). Their surfaces were extracted with optimized solvent mixtures or with C18, and analyzed comparatively against the whole metabolome by an untargeted metabolomics using molecular networks and other automated tools. In both studies, surface metabolome was chemically diverse and different from that of the whole organism. In *FV*, many surface compounds putatively dereplicated had bacterial or fungal origin, prompting to study the epibiome of *F. vesiculosus* by amplicon sequencing, SEM and CARD-FISH imaging. An untargeted spatial metabolomics by DESI-IMS localized various primary and SMs on surface imprints and in algal cross-sections providing information on their potential ecological functions. The surface metabolome of *ZM* was dominated by sulfated flavonoids (SFs) and lipids, including fatty acids (FAs). DESIIMS showed that FAs, e.g., palmitic acid, were distributed evenly on the leaf surface, while SFs were localized centrally with a high accumulation at the apex. The individual metabolites demonstrated differential activity against growth and/or settlement of epiphytic yeasts. To our knowledge, these studies represent the most in-depth studies on seaweed and seagrass surfaces.

#### **Interdisciplinary Approach to Study the Biotechnological Potential of Microbial Communities from Mangrove Sediments in Sisal, Yucatan, Mexico** 

Marfil-SantanaMiguel D.^1^Ruíz-HernándezAnaluisa^1^Remes-RodríguezClaudia A.^1^De La LuzJosé A.^1^Márquez-VelázquezNorma A.^1^Piñón-ChávezMarco. A.^2^Prieto-DavóAlejandra^1^1Laboratorio de Ecología Microbiana y Productos Naturales Marinos, Unidad de Química en Sisal, Facultad de Química, Universidad Nacional Autónoma de México, Sisal, Yucatán 97356, Mexico2Facultad de Química, Universidad Autónoma del Estado de Mexico, Toluca e Lerdo 50120, Mexico

In the last decades the use of metagenomics to study microbial communities has revealed their crucial role in sustaining ecological balances. Moreover, this technique can also be used as a strategy to identify genes involved in the biosynthesis of compounds of special interest, for example, those with biological activities. Mangrove sediments are, in general, environments with high diversity and richness of microorganisms which are adapted to particular environmental conditions involving changes in salinity and fresh water inputs. When analyzing three metagenomes from microniches in these sediments (canal, roots, middle) the three were basically replicates of each other with Proteobacteria, Cyanobacteria, Firmicutes, Bacteroidetes and Actinomycetes as the dominant Phyla in the three sites and them sharing the same main functions. A deeper analysis of genes involved in secondary metabolism suggested that sediments from the canal had the best biotechnological potential since they showed the highest number of genes for PKS (106) and NRPS (86) biosynthesis. From these sediments, 114 strains with actinomycete morphological characteristics were isolated and 90% showed presence of PKS genes by PCR amplification. Further targeted massive sequencing of 20 representative strains showed they possess genes involved with PKS and NRPS biosynthesis similar to those involved in the production of scabichelin, epothilone, actinomycin and oxazolomycin, amongst others. Taxonomic identification shows these strains belong to the genera *Streptomyces*, *Nocardiopsis* and *Pseudonocardia*. Further work on the chemistry in extracts from these strains will be presented. This work highlights the importance of using an interdisciplinary approach for natural product discovery. 

#### **Establishment of the Core Microbiome of the Ascidian *Synoicum adareanum* near Palmer Station, Antarctica** 

AvalonNicole E.^1^MurrayAlison E.^2^KokkaliariSofia^1^LoChien-Chi^3^DichosaArmand E. K.^3^DavenportKaren^3^ChainPatrick S. G.^3^BakerBill J.^1^1Department of Chemistry, University of South Florida, Tampa, FL 33620, USA2Division of Earth and Ecosystem Sciences, Desert Research Institute, Reno, NV 89512, USA3Los Alamos National Labs, Los Alamos, NM 87545, USA

Antarctic invertebrates are rich sources of secondary metabolites with high potential for bioactivity. Due to harsh conditions and the Antarctic Circumpolar Current, Antarctica has a distinct marine environment with rich biodiversity and novel chemistry. Many of the secondary metabolites isolated from invertebrates are thought to be produced, in whole or in part, by bacteria that are associated with the host organism. A macrolide with significant antitumor bioactivity has been isolated from the Antarctic ascidian, *Synoicum adareanum*, though the microbial producer of the compound of interest has not yet been established. Within 5 nautical km from Palmer Station, Antarctica, a total of 63 *S. adareanum* samples were collected—3 lobes of 3 different colonies from each of 7 distinct sites. The microbial community was determined and then bioinformatically standardized to 20,000 Amplicon Sequence Variants (ASVs) per sample. Evaluation of the microbial community through genomic approaches and multivariate analysis has been utilized to establish (1) the core microbiome, (2) the microbes that may serve key metabolic roles, but are not consistently associated with the host, and (3) the bacteria that are only transiently associated with the host, likely as a food source or due to presence in the water column at the time of sampling. Establishing the core microbiome of the ascidian will aid in determining the microbial producer of the key marine macrolide, which is found across all samples collected.

#### **Natural Products Involved in Parasite-Micro Algae Interactions** 

ValletMarine^1^BaumeisterTim U. H.^1^KaftanFilip^2^SvatošAleš^2^PohnertGeorg^1^,^3^1Fellow Group Plankton Community Interactions, Max Planck Institute for Chemical Ecology, 07743 Jena, Germany2Research Group Mass Spectrometry/Proteomics, Max Planck Institute for Chemical Ecology, 07743 Jena, Germany3Institute for Inorganic and Analytical Chemistry Bioorganic Analytics, Friedrich Schiller University Jena, 07745 Jena, Germany

Parasitism is one of the most common lifestyle in marine ecosystems and many pathogenic microorganisms thrive by infecting mammals, fishes, seaweeds, sponges, and corals. One often overlooked host is the phytoplankton, a highly diverse group of microalgae living as unicellular cells in the water column. Under eutrophic conditions, these algal cells can form a bloom and produce exudates such as harmful toxins. Marine parasites can be attracted by algal exudates and infect single cells in an orchestrated process, sometimes resulting in the bloom termination. Despite their relevance, very few of these marine parasites are investigated and the molecular mechanisms of the pathogenesis are unknown. To decipher the chemical ecology of parasite-microalgae interactions, we investigated two pathosystem models involving the specialist oomycete *Lagenisma coscinodisci* and its diatom host *Coscinodiscus*, and the generalist alveolate *Parvilucifera* that kills dinoflagellates. The metabolites involved in the pathogenesis were identified using infection experiments and comparative metabolomics based on high-resolution mass spectrometry. The role of the identified alkaloids were characterized in functional bioassays. Furthermore, the development of a new matrix-free laser desorption/ionization mass spectrometry approach enabled the identification and health assessment of single algal cells based on their cellular metabolites. The characterization of the infection markers will be compared with in situ observations in field studies.

#### **The *Phyllidiella pustulosa* Species Complex (Gastropoda, Nudibranchia): Do Secondary Metabolites Aid in Species Delimitation?** 

BogdanovAlexander^1^AdelfiaPapu^2^UndapNaniSchilloDorothee^2^KehrausStefan^1^SchäberleTill F.^3^WägeleHeike^2^KönigGabriele M.^1^1Institute for Pharmaceutical Biology, University of Bonn, 53115 Bonn, Germany2ZFMK Zoologisches Forschungsmuseum Alexander König, 53113 Bonn, Germany3Institute for Insekt Biotechnology, University of Gießen, 35392 Gießen, Germany

Members of the Phyllidiidae are brightly colored nudibranch gastropods, inhabiting many marine habitats, especially the coral reefs of the tropical Indo-Pacific. The lack of a protective shell is suggested to be compensated by a leather-like body tissue and toxic isocyanide and isothiocyanide terpenes (Garson MJ & Simpson JS 2004), which are sequestered from prey sponges. Despite the high abundance in nature, the phylogenetic relationships between more than 100 described members of Phyllidiidae are very controversial. 

Our reconstruction of Phyllidiid phylogeny using molecular data of more than 500 specimens collected from various localities and during different seasons in North Sulawesi, Indonesia indicates that *Phyllidiella pustulosa* is a species complex with at least six well supported clades. Subsequent metabolomic investigation of several of these specimens applying HR-LCMS demonstrated specific chemical profiles for the different *P. pustulosa* clades. It also became evident that secondary metabolites are not dependent on seasonal or geographic variations. Detailed chemical analysis of specimens from the *P. pustulosa* complex led to the isolation of sesquiterpenes bearing a characteristic isocyanide moiety. Guided by molecular networking (GNPS), seven new metabolites, i.e., dichloroimine sesquiterpenes (**1**–**2**) and their derivatives (**3**–**7**) were obtained from a distinct *P. pustulosa* clade (Figure 6). These findings will be discussed with regard to phylogenetic relationship, and are envisaged to support the description of new *Phyllidiella* species.

### ***Theme 2. Isolation and Structure Elucidation of Marine Natural Products*** 

#### **Marine Natural Products from Invertebrates. “Necessity Is the Mother of Invention”** 

MolinskiTadeusz F.^1^,^2^1Department of Chemistry and Biochemistry, University of California, 9500 Gilman Dr. MC0358, La Jolla, CA 92093, USA2Skaggs School of Pharmacy and Pharmaceutical Sciences, University of California, 9500 Gilman Dr. MC0358, La Jolla, CA 92093, USA

Recent studies in our lab have expanded the repertoire of techniques in NMR and chiroptical spectroscopy to define relative and absolute configuration in cases where existing technique were lacking. That ‘necessity is the mother of invention’ will be illustrated with inventive solutions to outsized problems in structure elucidation. In this talk, I will present recent results from investigations of new marine-derived alkaloids, glycolipids and halogenated natural products from marine sponges and ascidians, including new pulse sequence for detection of ^37,35^Cl isotope shifts in ^13^C at the microgram-scale. In addition, the first results of investigations reveal isotope fractionation that is informative of divergent mechanisms of halogenation in biosyntheses of marine natural products. 

#### **Tutuilamides A-C: Vinyl-Chloride Containing Cyclodepsipeptides from Two Marine Cyanobacteria** 

KellerLena^1^SuzukiBrian M.^2^CanutoKirley^1^,^3^AlmalitiJehad^1^,^4^SikandarAsfandyar^5^NamanC. Benjamin^1^GlukhovEvgenia^1^LuoDanmeng^6^DugganBrendan M.^2^LueschHendrik^6^KoehnkeJesko^5^O’DonoghueAnthony J.^2^GerwickWilliam H.^1^,^2^1Center for Marine Biotechnology and Biomedicine, Scripps Institution of Oceanography, University of California, La Jolla, CA 92093, USA2Skaggs School of Pharmacy and Pharmaceutical Sciences, University of California, La Jolla, CA 92093, USA3Embrapa Agroindústria Tropical, Fortaleza 60511-110, Brazil4Department of Pharmaceutical Sciences, Faculty of Pharmacy, University of Jordan, Amman 11942, Jordan5Workgroup Structural Biology of Biosynthetic Enzymes, Helmholtz Institute for Pharmaceutical Research Saarland, Helmholtz Centre for Infection Research, Saarland University, 66123 Saarbrücken, Germany6Department of Medicinal Chemistry and Center for Natural Products, Drug Discovery and Development (CNPD3), University of Florida, Gainesville, FL 32610, USA

Marine cyanobacteria have an enormous capacity to produce structurally diverse natural products that exhibit a broad spectrum of potent biological activities against mammalian cells and microbes such as fungi, bacteria and protozoa. Using mass spectrometry-guided fractionation together with molecular networking, a cyanobacterial field collection from American Samoa (*Schizothrix* sp.) yielded two new cyclic peptides, tutuilamide A and B. A second collection of *Coleofasciculus* sp. from Palmyra Atoll yielded a closely related compound, tutuilamide C. Their structures were established by spectroscopic techniques including 1D and 2D NMR, HR-MS, and chemical derivatization. Structure elucidation was facilitated by employing advanced NMR techniques including non-linear sampling (NUS) in combination with 1,1-ADEQUATE. These cyclic peptides are characterized by the presence of several unusual residues including 3-amino-6-hydroxy-2-piperidone (Ahp) and 2-amino-2-butenoic acid (abu) together with a novel vinyl chloride-containing residue. Activity of the serine protease, elastase, was potently inhibited by tutuilamide A (IC_50_ = 4.0 nM) and B (IC_50_ = 3.6 nM). In addition, these compounds displayed moderate potency in H-460 lung cancer cell cytotoxicity assays (IC_50_ = 0.53 μM and 1.26 μM, respectively). The binding mode to elastase was analyzed by X-ray crystallography, revealing a reversible binding mode similar to the natural product lyngbyastatin 7. The presence of an additional hydrogen bond with the amino acid backbone of the flexible side chain of tutuilamide A, compared to lyngbyastatin 7, facilitates its stabilization in the elastase binding pocket.

#### **Chemical Investigation of Tongan Invertebrates** 

TaufaTaitusi^1^†BracegirdleJoe^1^†SinghA. Jonathan^1^,^2^McConeJordan A. J.^1^RobertsonLuke P.^3^HumePaul^1^CarrollAnthony R.^3^NorthcotePeter T.^1^,^2^KeyzersRobert A.^1^,^4^1School of Chemical & Physical Sciences, and Centre for Biodiscovery, Victoria University of Wellington, Wellington 6140, New Zealand2Currently at Ferrier Research Institute, Victoria University of Wellington, Lower Hutt 5043, New Zealand3Griffith Institute for Drug Discovery, Griffith University, Brisbane 4111, Australia4Maurice Wilkins Centre for Molecular Biodiscovery, Wellington 6140, New Zealand†Equal contributions.

The Pacific Islands have historically been a rich source of new chemistry, although Tonga has been less of a focus than other groups like Fiji or Samoa. Consequently, we have made several collections of various marine invertebrate taxa from around Tonga and have been able to compare the chemistry of specific organisms from across this nation. 

We present the identification of the new and potent (nM) microtubule-stabilizing agents zampanolides B-E (Figure 7) from *Cacospongia mycofijiensis* and the unexpected SAR of these geometrical isomers. The discrepancy between zampanolide and dactylolide, compounds sharing the same core but the opposite absolute configurations, is also resolved. 

In a separate study, use of Global Natural Products Social molecular networking to screen tunicate-derived compounds has yielded a series of new sulfated lamellarins from *Didemnum ternerratum*, extending the number of sulfated lamellarins from 9 to 18. Several of the newly isolated lamellarins exhibit optical activity, with the absolute configuration of the atropisomeric lamellarin B1-sulfate being solved by ECD methods. This is the first time the absolute configuration of chiral lamellarins has been identified.

#### **Bioactive Secondary Metabolites from Deep-Sea Derived Microorganisms, Discovery and Extending** 

LiDehaiKey Laboratory of Marine Drugs, Chinese Ministry of Education, School of Medicine and Pharmacy, Ocean University of China, Qingdao 266003, China

Microorganisms derived from the deep-sea (depth > 1000 m) are a promising resource for natural products with novel chemical structures and specific bioactivities. According to our literature reviews and statistics, nearly 60% of the natural products from deep-sea-sample-derived microorganisms were reported to possess bioactivity with more than 30% demonstrating beneficial cytotoxicity. Although many attentions have been attracted, only about 400 deep-sea derived new secondary metabolites have been reported in the past decades, among the over 30,000 marine natural products. Moreover, most of the gene clusters encoding bioactive molecules are silent. For exploring the bioactive secondary metabolites from deep-sea derived microorganisms, we collected over 100 deep-sea samples with the depth from 1000 to 10,000 m and over 1500 strains were isolated from them. Employing modern natural products strategies and the silent gene activation methods (OSMAC, epigenetic modification, co-cultivation, genome manipulation, etc.), we have reported over 80 new deep-sea derived compounds (Figure 8) and some of them showed promising cytotoxic, antibacterial, antiviral, lipid-lowering activities, in the past 15 years. 

#### **Inconspicuous Carbon NMR Signals and Fluxional Effects Complicate the Structural Elucidation of Noriterpenes from *Goniobranchus coi*** 

ForsterLouise C.^1^PierensGregory K.^2^CheneyKaren L.^3^GarsonMary J.^1^1School of Chemistry and Molecular Biosciences, The University of Queensland, Brisbane 4072, Australia2Centre for Advanced Imaging, The University of Queensland, Brisbane 4072, Australia3School of Biological Sciences, The University of Queensland, Brisbane 4072, Australia

An individual *Goniobranchus coi* specimen collected from Mooloolaba, Australia, was found to contain an array of secondary metabolites. In addition to ten known rearranged oxygenated diterpenes, a series of keto functionalised norditerpenes were isolated. The investigation was complicated by fluxional behaviour of ring A, [1,2] which resulted in broaded signals in both ^1^H and ^13^C NMR spectra. The carbon framework and relative configurations were explored by decoupling and variable temperature NMR experiments at 700 MHz, informed by molecular modelling, DFT calculations and coupling constant predictions [3,4]. Palaemon shrimp palatability assays were conducted on selected compounds.

**Figure:**
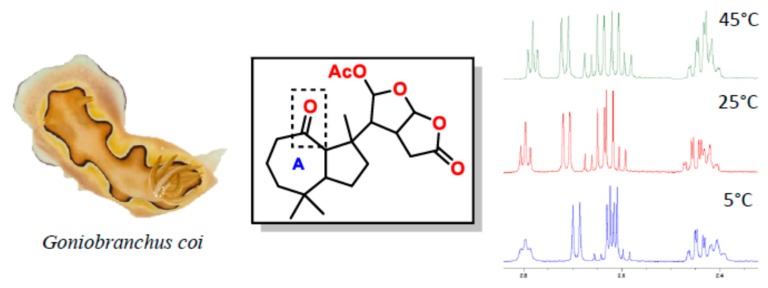
**Graphical Abstract**

References

1. House, H.O.; Gaa, P.C.; VanDerveer, D. Perhydroazulenes. 3. Conformations of the 4-oxoperhydroazulenes. *J. Org. Chem.*
**1983**, *48*, 1661–1670.
2. House, H.O.; Gaa, P.C.; Lee, J.H.; VanDerveer, D. Perhydroazulenes. 4. 6-tert-Butyl-4-oxoperhydroazulene system. *J. Org. Chem.*
**1983**, *48*, 1670–1678.
3. Forster, L.C.; Pierens, G.K.; Garson, M.J. Elucidation of Relative and Absolute Configurations of Highly Rearranged Diterpenoids and Evidence for a Putative Biosynthetic Intermediate from the Australian Nudibranch *Goniobranchus geometricus*. *J. Nat. Prod.*
**2019**, *82*, 449–455.
4. Dewi, A.S.; Pierens, G.K.; Cheney, K.L.; Blanchfield, J.T.; Garson, M.J. Chromolactol, an Oxygenated Diterpene from the Indo-Pacific Nudibranch *Goniobranchus coi*: Spectroscopic and Computational Studies. *Aust. J. Chem.*
**2018**, *71*, 798–803.

#### **Marine Polychaetes as an Untapped Source of Chemical Diversity: Case Study of the Irish Intertidal Terebellid *Eupolymnia nebulosa*** 

CalabroKevinLasserrePerrineJenningsLaurenceThomasOlivier P.Marine Biodiscovery, School of Chemistry and Ryan Institute, National University of Ireland Galway, University Road, H91 TK33 Galway, Ireland

Marine invertebrates have been recognized as an outstanding source of diverse and bioactive specialized metabolites. Sponges and tunicates are the most important producers of natural products and continue to be the main targets of worldwide bioprospecting studies, leaving aside other important groups of organisms such as annelids. As part of a national project aiming at the description of Irish marine invertebrates for biodiscovery (NMBLI), some intertidal terebellids have been collected. A first chemical screening along with a dereplication process using the online platform GNPS, led to the selection of the polychaete *Eupolymnia nebulosa* for further chemical investigation. 

Herein, we report the isolation and structure elucidation of nebulosins, cysteine derivatives containing an unprecedented highly branched thiolane ring (Figure 9). The relative configurations were deduced from the NOESY spectrum and *J*-based configurational analysis (JBCA) while the absolute configuration was established by comparison between theoretical and experimental ECD spectra. We also propose a plausible mechanism for the biosynthesis of these unique metabolites. The evaluation of the antioxidant activities of the sixteen nebulosins are ongoing.

### ***Theme 3. Drug Discovery and Development*** 

#### **Pheno-Target Screening: Combining Cell Assays and Target Identification** 

QuinnRonald J.Griffith Institute for Drug Discovery, Griffith University, Brisbane 4111, Australia

There is considerable risk in advancing a compound into development without identifying its molecular target. For many years, target-based drug discovery and associated high throughput screening was a dominant strategy in drug discovery, however there is a switch to cell-based phenotypic screening. Cell-based phenotypic screening can be target agnostic, requiring downstream identification of the molecular level mechanism. 

To address target deconvolution following cell-based phenotypic screening, we introduce a concept termed *‘pheno-target screening’*.

Native mass spectrometry using a Magnetic Resonance Mass Spectrometer (MRMS) can detect protein in its native folded state, it can detect and distinguish non-covalent and covalent protein-ligand complexes, it has high sensitivity, low sample consumption, it does not require derivatization of the compound or labeling of the protein target, and provides a direct visualization of all species present in solution under binding conditions. 

We have harnessed the chemical diversity of natural products for fragment-based drug screening. We have reported 96 low molecular weight natural products identified as binding partners of 32 putative malarial targets. Seventy-nine (79) fragments have direct growth inhibition on *Plasmodium falciparum* at concentrations that are promising for development of fragment hits against these protein targets. This adds a fragment library to the published HTS active libraries in the public domain. 

We illustrate the concept of *pheno-target screening* (Figure 10) using a cell-based phenotypic assay against *Mycobacterium tuberculosis* coupled to target identification using native mass spectrometry.

#### **NP^3^ Platform: Combined Protein Crystallography and MS-Based Metabolomics to Empower Natural Product Drug Discovery** 

de FelicioRafael^1^BazaanoCristina F.^1^,^2^AlvesLuiz G.^1^InfanteGina P. Polo^1^CunhaMarcos G.^1^AmadorIanka B.^1^Velasco-AlzateKaren^3^FurtadoLuciana C.^3^GubianiJuliana R.^4^FerreiraEverton L. F.^4^VieiraBruna D.^1^FerreiraRaquel O.^1^NascimentoAndrey F.^5^ZeriAna C. M.^5^TellesGuilherme^2^BerlinckRoberto G. S.^4^Costa-LotufoLeticia V.^3^TrivellaDaniela B. B.^1^1Brazilian Biosciences National Laboratory (LNBio), Brazilian Center for Research in Energy and Materials (CNPEM), Campinas 13083-970, Sao Paulo, Brazil2Institute of Computer Sciences, University of Campinas, Campinas 13083-852, Sao Paulo, Brazil3Department of Pharmacology, University of Sao Paulo, Sao Paulo 05508-900, Brazil4Chemistry Institute of Sao Carlos, University of Sao Paulo, Sao Carlos 13566-590, Sao Paulo, Brazil5Brazilian Synchrotron Light Laboratory (LNLS), Brazilian Center for Research in Energy and Materials (CNPEM), Campinas 13083-970, Sao Paulo, Brazil

Natural substances provide new chemical scaffolds for drug discovery and can probe novel enzyme binding sites and inhibition mechanisms. However, the identification of bioactive natural products and their enzyme binding mechanisms is challenging, sample and time-consuming. We have developed an integrated approach to overcome these gaps, based on high throughput screening of pre-fractionated NP libraries, X-ray protein crystallography and mass spectrometry techniques, all assisted by designed computer algorithms for identifying bioactive natural products and their enzyme binding sites, in the very early stages of natural product based drug discovery. This approach was named the NP^3^ platform and works as follows: crystallographic fishing of the bioactive natural product from non-purified and unknown complex chemical samples with crystals of the target protein reveals the active natural product binding site, its mechanism of interaction with the enzyme, and provides initial clues on its chemical structure. LC-MS/MS-based metabolomics is then employed for filtering candidate *m/z* (compounds) in the unknown mixture and, by an iterative process of crystal electron density and MS/MS spectra interpretation, it is possible to reveal the chemical identity of the bioactive natural product. This iterative approach proved successful even when using low resolution protein crystals and active natural products present in trace amounts in complex chemical samples. The process can be performed in miniaturized scales, in which each step is compatible with high throughput techniques. The NP^3^ platform is empowering natural product drug discovery in our pipeline, as it will be exemplified by marine fungi and bacterial extracts screened and deconvoluted with the NP^3^ platform.

##### **Acknowledgments** 

Serrrapilheira Institute 3659, FAPESP 15/17177-6 and 13/50228-8.

#### **Antiparasitic Metabolites Isolated from the Antarctic Algae-Associated Fungus: *Penicillium echinulatum*** 

TeixeiraThaiz R.^1^FilhoPéricles G. Abreu^1^YatsudaAna P.^1^ClementinoLeandro C.^2^GraminhaMárcia A. S.^2^JordãoLaís G.^3^PohlitAdrian M.^3^ColepicoloPio^4^DebonsiHosana M.^1^1School of Pharmaceuticals Sciences of Ribeirão Preto—University of São Paulo, Ribeirão Preto, SP 14040-903, Brazil2School of Pharmaceuticals Sciences of São Paulo State University, Araquaquara, SP 14800-903, Brazil3National Institute of Amazonian Research—INPA, Manaus, AM 69067-375, Brazil4Institute of Chemistry—University of São Paulo, São Paulo, SP 05508-000, Brazil

The neglected diseases such as leishmaniasis, malaria and neosporosis affect millions of people and animals around the world. Currently there are few therapeutic options available, which present several drawbacks, such as high toxicity, turning the development of new drugs urgent. Herein, the antiparasitic activity (IC_50_) of crude extract, fractions and isolated substances from *Adenocystis utricularis* endophytic fungus *Penicillium echinulatum* were evaluated against the etiologic agents *Leishmania amazonensis*, *Plasmodium falciparum* and *Neospora caninum*, whilst its cytotoxic potential (CC_50_), in murine peritoneal macrophages. For this, after determine the antileishmanial activity of the crude extract (IC_50-PRO_ = 21.2 ± 1.0 μg/mL), we performed extract fractionation and the nine obtained fractions, LCV-A to LCV-I, were further characterized regarding their antileishmanial properties. LCV-E (IC_50-PRO_ = 25.7 ± 1.4 μg/mL) was the most bioactive fraction and was further explored in order to identify its antileishmanial compound(s). Indeed, among the isolated compounds, viridicatin presented an outstanding antileishmanial activity against amastigotes (IC_50-AMA_ = 4.1 ± 0.6 μg/mL; SI > 125) of *L. amazonensis*, the most relevant parasite form responsible for cutaneous leishmaniasis development. Moreover, viridicatin is safer than the reference drug amphotericin-B (IC_50-AMA_ = 5.4 ± 0.7 μg/mL; SI = 3.9), considering its selectivity index (SI = CC_50_/IC_50_), the compound is 30-times more selective to the parasite rather than to the host cell, indicating its low cytotoxic potential and, consequently, its safeness to be further considered in the pipeline of drug discovery process for leishmaniasis. Besides, viridicatin also showed mild anti-*Plasmodium* (IC_50_ = 18.5 ± 4.3 μg/mL) and excellent anti-*Neospora* (IC_50_ = 2.2 ± 0.1 μg/mL, SI > 227) activity, indicating viridicatin as promising hit for development of a wide spectrum drug to fight this neglected parasitic diseases.

**Figure:**
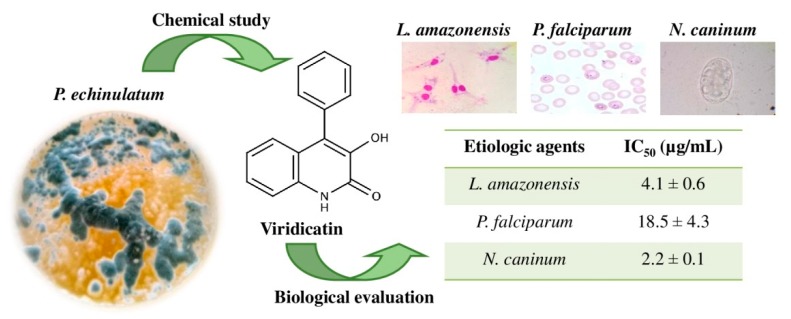
**Graphical Abstract**

#### **Amentadione, a Meroditerpenoide from Brown Algae with Capacity of Modulating Osteoarthritic Responses, in a Close-to-In-Vivo OA Model** 

AraújoNuna^1^ViegasCarla^1^,^2^PerrolasInês^1^CostaRúben^1^MarreirosCatarina^1^MagalhãesJoana^3^BlancoFrancisco^3^RamosAcácio^4^CarvalhoMaria Miguel^4^SousaJoão Paulo^4^VermeerCees^5^ZúbiaEva^6^SimesDina^1^,^2^1Centre of Marine Sciences (CCMAR), University of Algarve, 8005-139 Faro, Portugal2GenoGla Diagnostics, CCMAR, University of Algarve, 8005-139 Faro, Portugal3Grupo de Bioingeniería Tisular y Terapia Celular, Instituto de Investigación Biomédica de A Coruña, Complejo Hospitalario Universitario de A Coruña, Sergas Universidad de A Coruña, 15006 A Coruña, Spain4Department of Orthopedics and Traumatology, Hospital Particular do Algarve, 8005-226 Gambelas-Faro, Portugal5VitaK, Maastricht, 6229 EV Maastricht, The Netherlands6Department of Organic Chemistry, Faculty of Marine and Environmental Sciences, University of Cadiz, 11510 Cádiz, Spain

Osteoarthritis (OA) is a degenerative and chronic disease, prevalent in the middle-aged and older individuals affecting 10–15% of all adults aged over 60. OA is a complex and multifactorial joint disease characterized by loss of articular cartilage, subchondral bone remodelling and synovial inflammation. Current osteoarthritis treatment is still limited and mainly focused on symptoms management and relif, prompting the need for the identification of new therapeutic targets and the development of novel disease-modifying agents. Macroalgae represent a wide source of bioactive compounds with potential preventive and therapeutic applications for several inflammatory diseases including osteoarthritis. In this study, we developed an experimental pipeline, including a OA primary culture system followed by a more complex OA human cartilage co-culture, mimicking an early stage of the disease. The in vitro and ex-vivo OA models were stimulated with interleukin-1β (IL-1β) and hydroxyapatite (HAP), to provide the closest approximation of in vivo conditions, were mineralization interplays with inflammation. To investigate the anti-osteoarthritic potential of amentadione (YP), a meroditerpenoid isolated from the brown algae Cystoseira usneoides, the inflammatory responses of the OA models treated with YP, were assessed by measuring the expression of specific OA genes, and the accumulation of OA markers. The results revealed that YP downregulated the expression of inflammatory/degradation genes, and diminished the production of inflammatory agents, under inflammatory/mineralizing conditions. In conclusion, these results suggest that YP has a protective role in OA development and progression and should be considered of potential value for the treatment of osteoarthritis.

##### **Acknowledgments** 

Nuna Araújo is the recipient of the Portuguese Science and Technology Foundation (FCT) fellowship SFRH/BD/111824/2015, and. Catarina Marreiros is the recipient of a 0055 ALGARED+5E fellowship. This work was financed by FCT through the transitional provision DL57/2016/CP1361/CT0006 and project UID/Multi/04326/2019.

#### **Minimizing Taxonomic and Natural Product Redundancy in Microbial Libraries using MALDI-TOF MS and the Bioinformatics Pipeline IDBac** 

CostaMaria S.^1^,^2^ClarkChase M.^2^OmarsdottirSesselja^1^SanchezLaura M.^2^MurphyBrian T.^2^1Faculty of Pharmaceutical Sciences, University of Iceland, Hagi, Hofsvallagata 53, IS-107 Reykjavík, Iceland2Department of Pharmaceutical Sciences, College of Pharmacy, University of Illinois at Chicago, 833 South Wood Street (MC 781), Room 539, Chicago, IL 60607, USA

Libraries of microorganisms have been a cornerstone of drug discovery efforts in the past century, however the high degree of strain duplication in these libraries has resulted in unwanted natural product redundancy. In the current study, we implemented a workflow that maximizes natural product diversity while minimizing the total number of bacteria in a library. Using a collection expedition to Iceland as an example, we purified all distinguishable bacterial colonies (1616 total) off of isolation plates derived from 86 environmental samples. We employed our mass spectrometry (MS) based-IDBac workflow on these isolates to form groups of taxa (based on protein MS fingerprints; 3–15 kDa), and further distinguished taxa subgroups based on their degree of overlap within corresponding natural product spectra (0.2–2 kDa). This informed the decision to create a library of 301 isolates spanning 54 genera. This process required only 25 h of data acquisition and 2 h of analysis. In a separate experiment, we reduced the size of an existing library (from 833 to 233 isolates, a 72.0% reduction) without sacrificing natural product diversity. Overall, our pipeline minimizes library entries and costs associated with library generation, and maximizes chemical space entering into downstream biological screening efforts. This workflow represents a significant advance toward frontend discovery efforts by minimizing the serendipity that has hampered library generation. 

**Figure:**
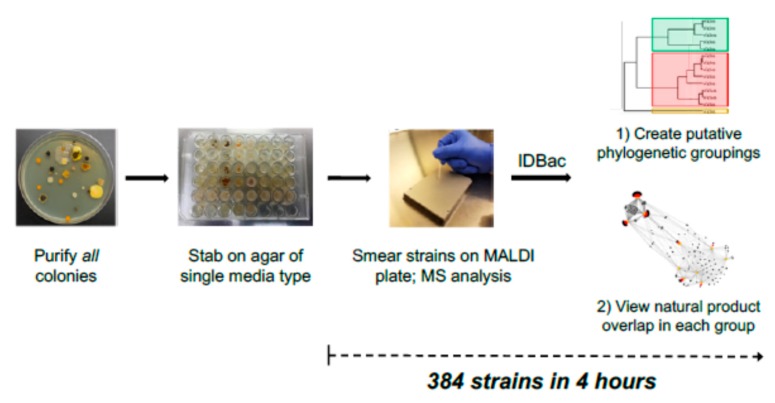
**Graphical Abstract**

#### **A Target-Oriented Approach for Bioprospecting IAP Modulators in Extracts from Marine Bacteria** 

BarbosaGabriel H.^1^BrancoPaola C.^2^Reis-SilvaCatarina S. M.^2^BauermeisterAnelize^2^,^3^LopesNorberto P.^3^Costa-LotufoLeticia V.^2^JimenezPaula C.^1^1Instituto do Mar, Universidade Federal de São Paulo, Baixada Santista 11070-100, Brazil2Instituto de Ciências Biomédicas, Universidade de São Paulo, São Paulo 05508-000, Brazil3Faculdade de Ciências Farmacêuticas, Universidade de São Paulo, Ribeirão Preto 14040-903, Brazil

The Inhibitor of Apoptosis Proteins (IAPs) are relevant targets in cancer research, as members of this class are found overexpressed in several tumor types and almost completely absent in normal cells. Thus, this work aimed to prospect, through a target-guided approach, natural products from marine actinobacteria that modulate IAPs, further validating their bioactivity in in vitro assay models. Three proteins from the IAP family (survivin, livin and XIAP) obtained by heterologous expression in *E. coli* vector were attached to a resin support and incubated with marine actinobacteria extracts using a bioaffinity protocol named functional chromatography. Molecules retained in the resin were eluted and analyzed by HPLC-MS. From 19 extracts tested, one from a *Streptomyces* sp. strain (BRA-214) recovered from the sediments from St. Peter and St. Paul’s Archipelago returned 6 hits, four of which (*m*/*z* 613, 704, 718 and 752 [M + H]^+^) were isolated by semi-preparative HPLC. Although their structures are not yet completely solved, fragmentation patterns are indicative of unknown non-ribosomal peptides. For biological validation, SKMEL-19 (human melanoma) cell line, which had shown highest expression of survivin from the cell panel assessed, showed to be more sensitive to the isolated compounds, while the compound with *m*/*z* 704 induced a lower expression of survivin, while increasing apoptosis makers. Therefore, the functional chromatography bioaffinity protocol showed to be a valid and applicable technique to prospect for IAP ligands, allowing the identification and recovery of unprecedented secondary metabolites that seem to interact and modulate this family of proteins. 

##### **Acknowledgments** 

CAPES: INCT BioNat (CNPq/INCT #465637/2014-0); FAPESP (#2015/17177-6; 2017/17648-4); PPG Bioprodutos e Bioprocessos (UNIFESP).

### ***Theme 4. Biosynthesis of Marine Natural Products*** 

#### **Comparative Genomics Unravels Cryptic Diversity in the Cyanobactin Biosynthetic Pathway** 

MattilaAnttiJokelaJouniWahlstenMattiSivonenKaarinaFewerDavid P.Department of Microbiology, University of Helsinki, Biocenter 1, Viikinkaari 9, FI-00014 Helsinki, Finland

Cyanobactins are a rapidly expanding class of natural products produced through the post-translational modification of short precursor proteins [1–5]. Here we traced the distribution of cyanobactin biosynthetic pathways across the bacterial domain. This analysis demonstrates that the capacity to produce cyanobactins is spread throughout the bacterial domain and many of the newly identified cyanobactin gene clusters encode potent antimicrobial peptides. We report novel cyanobactin post-translational modifications including prenylation, phosphorylation and disulfide bridge formation. Partial reconstitution and heterologous expression of cyanobactin biosynthetic pathways encode rapidly evolving enzymes that catalyze the regiospecific and stereospecific modification of amino acids in macrocyclic and linear peptides. We reconstructed the evolutionary history of the cyanobactin biosynthetic pathways and examined the roles of common ancestry, convergent evolution, horizontal gene transfer, and recombination in promoting the chemical diversity of these natural products. These findings broaden the structural diversity of the cyanobactin family to include highly modified linear and macrocyclic peptides with rare post-translational modifications.

References

1. Dittmann, E.; Fewer, D.P.; Neilan, B.A. Cyanobacterial toxins: biosynthetic routes and evolutionary roots. *FEMS Microbiol. Rev*. **2013**, *37*, 23–43.
2. Dalponte, L.; Parajuli, A.; Younger, E.; Mattila, A.; Jokela, J.; Wahlsten, M.; Leikoski, N.; Sivonen, K.; Jarmusch, S.A.; Houssen, W.E.; et al. N-Prenylation of tryptophan by an aromatic prenyltransferase from the cyanobactin biosynthetic pathway. *Biochemistry*
**2018**, *57*, 6860–6867.
3. Alexandru-Crivac, C.N.; Umeobika, C.; Leikoski, N.; Jokela, J.; Rickaby, K.A.; Grilo, A.M.; Sjö, P.; Plowright, A.T.; Idress, M.; Siebs, E.; et al. Cyclic peptide production using a macrocyclase with enhanced substrate promiscuity and relaxed recognition determinants. *Chem. Commun.*
**2017**, *53*, 10656–10659.
4. Parajuli, A.; Kwak, D.H.; Dalponte, L.; Leikoski, N.; Galica, T.; Umeobika, U.; Trembleau, L.; Bent, A.; Sivonen, K.; et al. A unique tryptophan C-prenyltransferase from the kawaguchipeptin biosynthetic pathway. *Angew. Chem. Int. Ed. Engl*. **2016**, *55*, 3596–3599.
5. Leikoski, N.; Liu, L.; Jokela, J.; Wahlsten, M.; Gugger, M.; Calteau, A.; Permi, P.; Kerfeld, C.A.; Sivonen, K.; Fewer, D.P. Genome mining expands the chemical diversity of the cyanobactin family to include highly modified linear peptides. *Chem. Biol.*
**2013**, *20*, 1033–1043.

#### **Single-Bacterial Genomics, Raman Spectroscopy, and Biochemical Studies Identify Metabolic Functions of Uncultivated Marine Microbiota** 

HemmerlingFranziska^1^KogawaMasato^2^HosokawaMasahito^2^AndoMasahiro^2^MoriTetsushi^3^Maciejewska-RodriguesHanna^1^CahnJackson K. B.^1^TakeyamaHaruko^2^PielJörn^1^1Institute of Microbiology, Swiss Federal Institute of Technology (ETH), 8093 Zurich, Switzerland2Department of Life Science and Medical Bioscience, Waseda University, Tokyo 169-8050, Japan3Division of Biotechnology and Life Sciences, Tokyo University of Agriculture and Technology, Tokyo 183-8538, Japan

Uncultivated bacteria represent an underexplored source for the production of bioactive metabolites such as antibiotics. The marine sponge *Theonella swinhoei* Yellow (TSY), has previously been described as being inhabited by the bacterial genus Ca. ‘Entotheonella’, a ‘talented producer’ of various natural products (NPs). In previous work, biosynthetic gene clusters (BGCs) for all NP families isolated from TSY were assigned in ‘*Entotheonella*’ spp., with the exception of the aurantosides, which are responsible for the strikingly yellow phenotype of the sponge. To target this gap, a fraction of uncultivated filamentous microbiota from TSY was encapsulated with a microfluidic device, and single cells were isolated with a micropipette. We identified the aurantoside producer by Raman microscopy, and sequenced single cell genomes by in-droplet multiple displacement amplification. The sequencing data revealed a putative *ats* BGC, which we linked to aurantoside production via reconstitution of a biosynthetic transformation. Phylogenetic analysis of the 16S rRNA gene revealed a new chloroflexi candidate genus ‘*Poriflexus*’ with the aurantoside producer Ca. ‘*Poriflexus aureus*’ as its only representative. We further analyzed the ‘*P. aureus*’ draft genome and encountered an impressive number and diversity of putative BGCs, and biosynthetic domains from multiple NP families. Our data suggest that TSY harbors not only ‘*Entotheonella*’ spp., but also Ca. ‘*P. aureus*’ as talented NP producers, and shows the potential to discover further compounds in this marine source in the future.

#### **Nuclear Magnetic Resonance, Mass Spectrometry and Chemical Probes in Natural Product Biosynthesis** 

CrumpMatthewSchool of Chemistry, University of Bristol, Cantock’s Close, Bristol BS8 1TS, UK

Polyketide natural products (produced by polyketide synthases (PKSs) currently serve as a vast source of high value compounds with applications spanning agrochemicals, antibiotics, anti-cancer and human and veterinary medicine as well as tool compounds for use in biotechnological research. Enormous strides have been made in understanding the chemistry, biochemistry and structural biology of the PKSs that are complex collections of enzymes with a diverse array of architectures. 

Our work focuses on the biosynthesis of key structural features of several naturalproducts including the the anti-MRSA antibiotic kalimantacin, marine bacterium derived thiomarinol, and mupirocin (a mixture of pseudomonic acids) [1,2]. We have recently elucidated a number of important mechanistic steps in the assembly of these metabolites and this presentation will focus on the role of structural biology [3], state-of-the-art Nuclear Magnetic Resonance, Mass Spectrometry and ACP bound chemical probes in the analysis of kalimantacin. 

**Figure:**
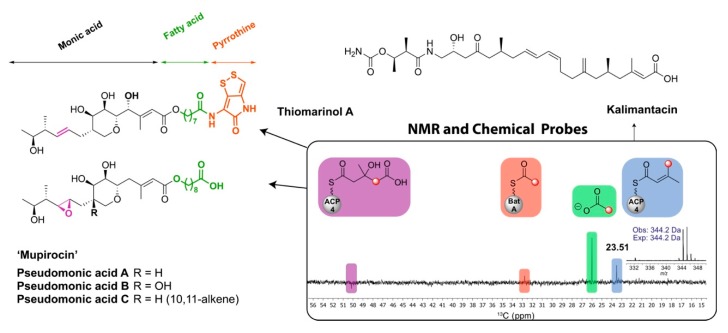
**Graphical Abstract**

References

1. Gao, S.-S.; Wang, L.; Song, Z.; Hothersall, J.; Stevens, E.R.; Connolly, J.; Winn, P.J.; Cox, R.J.; Crump, M.P.; Race, P.; et al. Selected Mutations Reveal New Intermediates in the Biosynthesis of Mupirocin and the Thiomarinol Antibiotics. *Angew. Chem. Int. Ed.*
**2017**, *56*, 3930–3934.
2. Thistlethwaite, I.R.G.; Bull, F.M.; Cui, C.; Walker, P.D.; Gao, S.-S.; Wang, L.; Song, Z.; Masschelein, J.; Lavigne, R.; Crump, M.P.; et al. Elucidation of the relative and absolute stereochemistry of the kalimantacin/batumin antibiotics. *Chem. Sci.*
**2017**, *8*, 6196–6201.
3. Haines, A.S.; Dong, X.; Song, Z.; Farmer, R.; Williams, C.; Hothersall, J.; Ploskon, E.; Wattana-Amorn, P.; Stephens, E.R.; Yamada, E.; et al. A conserved motif flags acyl carrier proteins for b-branching in polyketide synthesis. *Nat. Chem. Biol.*
**2013**, *9*, 685–692.

#### **A Symbiosis of Synthesis and Synthetic Biology in the Elucidation of Polyketide Biosynthesis** 

WillisChristine L.School of Chemistry, University of Bristol, Cantock’s Close, Bristol BS8 1TS, UK

Polyketide-derived natural products isolated from bacteria exhibit a range of important biological activities making them attractive leads for the development of therapeutics and agrochemicals. They are assembled in the host organism via sophisticated multiple enzyme architectures—polyketide synthases. Our overall aim is to fully understand how polyketides are produced and thus enable rational engineering of the complex biosynthetic machinery to deliver novel bioactive compounds cleanly and efficiently in scalable amounts. 

This lecture will focus on the combination of synthetic biology and organic synthesis to investigate polyketide biosynthesis enabling the generation of new bioactive targets and biocatalysts of potential widespread value. Examples will be taken from our recent work on marine natural products such as the thiomarinols [1,2] (and structurally related mupirocin) and abyssomicin C (Figure 11) [3,4].

References

1. Wang, L.; Parnell, A.E.; Williams, C.; Baker, N.A.; Challand, M.R.; van der Kamp, M.; Simpson, T.J.; Race, P.R.; Crump, M.P.; Willis, C.L. A Rieske oxygenase/epoxide hydrolase-catalysed reaction cascade creates oxygen heterocycles in mupirocin biosynthesis. *Nat. Catal.*
**2018**, *1*, 968.
2. Gao, S.-S.; Wang, L.; Song, Z.; Hothersall, J.; Stevens, E.R.; Connolly, J.; Winn, P.J.; Cox, R.J.; Crump, M.P.; Race; Thomas, C.M.; Simpson, T.J.; Willis, C.L. Selected mutations reveal new intermediates in the biosynthesis of mupirocin and thiomarinol antibiotics. *Angew. Chem. Int. Ed.*
**2017**, *56*, 3930.
3. Byrne, M.J.; Lees, N.R.; Han, L.-C.; van der Kamp, M.; Mulholland, A.J.; Stach, J.E.M.; Willis, C.L.; Race, P.R. The catalytic mechanism of a natural Diels-Alderase revealed in molecular detail. *J. Am. Chem. Soc.*
**2016**, *138*, 6095.
4. Lees, N.R.; Han, L.C.; Byrne, M.J.; Davies, J.A.; Parnell, A.E.; Moreland, P.E.J.; Stach, J.E.M.; van der Kamp, M.W.; Willis, C.L.; Race, P.R. An esterase-like lyase catalyzes acetate elimination in spirotetronate/spirotetramate biosynthesis. *Angew. Chem. Int. Ed.*
**2019**, *58*, 2305.

#### **Structural and Functional Dissection of an Enzymatic Natural Product Assembly Line** 

MaschioL.^1^ParnellA. E.^1^MbathaS.^2^van der KampM. W.^1^HayS.^3^PudneyC. R.^4^TangT. D.^5^BurstonS. G.^1^WillisC. L.^2^RaceP. R.^1^1Faculty of Life Sciences, School of Biochemistry, University of Bristol, Bristol BS8 1TD, UK2Faculty of Science, School of Chemistry, University of Bristol, Bristol BS8 1TS, UK3Department of Biology and Biochemistry, Faculty of Science, University of Bath, Bath, BA2 7AX, UK4Manchester Institute of Biotechnology and School of Chemistry, Faculty of Science and Engineering, The University of Manchester, Manchester, M1 7DN, UK5Max-Planck Institute for Molecular Cell Biology and Genetics, Pfotenhauerstraße 108, 01307 Dresden, Germany

Abyssomicin C is a broad-spectrum antibacterial natural product that was first isolated from the marine bacterium *Verrucosispora maris*. This compound exhibits potent bioactivity against methicillin resistant *Staphylococcus aureus* (MRSA) and shows potential for development as a clinically useful antibiotic. Abyssomicin C is the simplest of the spirotetronate polyketides, and studies of the biosynthetic pathway to this compound have revealed a multitude of enzymatic peculiarities. Here I outline progress across three strands of activity: (i) structural and functional analysis of the intact type I polyketide synthase AbyB3, using a combination of *cryo*-electron microscopy and X-ray crystallography. (ii) Fundamental studies of the *aby* pathway enzyme AbyU, a *bona fide* natural Diels-Alderase, exploring substrate selectivity and the ability of this enzyme to generate ‘non-natural’ spirotetronates, as well as an in-depth mechanistic study, revealing a model by which Diels-Alderases and other b-barrel cytosolic enzymes act in stereo- and regio-selective manners to catalyse key industrial reactions. This information has been used to guide the expression of a suite of mutants with improved biocatalytic abilities. (iii) Studies revealing AbyU to be a highly stable, piezophilic enzyme, well adapted to pressures seen both in a marine environment and in industrial processes.

#### **Avant-Garde Assembly-Line Biosynthesis Expands Diversity of Cyclic Lipodepsipeptide Products** 

ZhangJia Jia^1^TangXiaoyu^1^HuanTao^2^RossAvena C.^3^MooreBradley S.^1^,^4^1Center for Marine Biotechnology and Biomedicine, Scripps Institution of Oceanography, University of California at San Diego, La Jolla, CA 92037, USA2Department of Chemistry, University of British Columbia, Vancouver, BC V6T 1Z1, Canada3Department of Chemistry, Queen’s University, Kingston, ON K7L 3N6, Canada4Skaggs School of Pharmacy and Pharmaceutical Sciences, University of California at San Diego, La Jolla, CA 92037, USA

Modular nonribosomal peptide synthetase (NRPS) and polyketide synthase (PKS) enzymatic assembly lines are large and dynamic protein machines that generally undergo a linear progression of catalytic cycles via a series of enzymatic domains organized into independent modules. Here we report the heterologous reconstitution and comprehensive characterization of two hybrid NRPS-PKS assembly lines from marine Alphaproteobacteria that defy many standard rules of assembly-line biosynthesis to generate a large combinatorial library of cyclic lipodepsipeptide protease inhibitors called thalassospiramides. We generate a series of precise domain-inactivating mutations in thalassospiramide assembly lines and present compelling evidence for an unprecedented biosynthetic model that invokes inter-module substrate activation and tailoring, module skipping, and pass-back chain extension, whereby the ability to pass the growing chain back to a preceding module is flexible and substrate-driven. Expanding bidirectional inter-module domain interactions could represent a viable mechanism for generating chemical diversity without increasing the size of biosynthetic assembly lines and raises new questions regarding our understanding of the structural features of multi-modular megaenzymes.

### ***Theme 5. Synthesis of Marine Natural Products and Medicinal Chemistry*** 

#### **Biomimetic Photooxidation of the Macrocyclic Core of Salarin C from the Sponge *Fascaplysinopsis* sp.** 

SchäckermannJan-NiklasLindelThomasInstitute of Organic Chemistry, TU Braunschweig, 38106 Braunschweig, Germany

The salarins from the marine sponge *Fascaplysinopsis* sp. (Madagascar) constitute a unique group of 17-membered lactones [1,2]. Salarin C (**1**, Figure 12) showed promising cytotoxicity against human leukemia cells by inducing mitotic cycle arrest, apoptosis, and DNA damage [3]. Interestingly, Kashman and coworkers observed the transformation of the oxazole unit of salarin C (**1**) to the trisacylamine moiety of the less cytotoxic salarin A, when storing **1** under light and oxygen.

We were intrigued by the question on whether such a Wasserman-type rearrangement could be induced in the reaction flask. Having synthesized the trisubstituted oxazole via halogen dance reaction, we could show that an open-chain model compound indeed underwent conversion to the trisacylamine when treated with photochemically generated singlet oxygen [4]. In this communication, we describe the first synthesis of the carbon skeleton of the salarin C macrocycle that is to be extended further towards the total synthesis of that marine natural product. Conformational analysis of the macrocycle was already possible, as was the oxidation of macrocycle (**2**) to trisacylamine (**3**) in high yield [5].

References

1. Bishara, A.; Rudi, A.; Aknin, M.; Neumann, D.; Ben-Califa, N.; Kashman, Y. Salarin C, a new cytotoxic sponge-derived nitrogenous macrolide. *Tetrahedron Lett*. **2008**, *49*, 4355‒4358.
2. Bishara, A.; Rudi, A.; Aknin, M.; Neumann, D.; Ben-Califa, N.; Kashman, Y. Salarins D–J, seven new nitrogenous macrolides from the madagascar sponge *Fascaplysinopsis* sp. *Tetrahedron*
**2010**, *66*, 4339‒4345.
3. Del Poggetto, E.; Tanturli, M.; Ben-Califa, N.; Gozzini, A.; Tusa, I.; Cheloni, G.; Marzi, I.; Cipolleschi, M.G.; Kashman, Y.; Neumann, D.; et al. Salarin C inhibits maintenance of hypoxia-selected Chronic Myeloid Leukemia progenitor cells. *Cell Cycle*
**2015**, *14*, 3146‒3154.
4. Schäckermann, J.-N.; Lindel, T. Synthesis and photooxidation of the trisubstituted oxazole fragment of the marine natural product salarin C. *Org. Lett.*
**2017**, *19*, 2306‒2309.
5. Schäckermann, J.-N.; Lindel, T. Macrocyclic core of salarin C: synthesis and oxidation. *Org. Lett.*
**2018**, *20*, 6948‒6951.

#### **Antiproliferative Effect of Prenylated Diketopiperazine and Barbituric Acid Derivatives against Triple Negative Breast Cancer** 

AdegokeYusuf A.^1^de la MareJo-Anne^2^EdkinsAdrienne L.^2^BeukesDenzil R.^1^1School of Pharmacy, University of the Western Cape, Robert Sobukwe Road, Bellville 7535, South Africa2Biomedical Biotechnology Research Unit, Department of Biochemistry, Microbiology and Biotechnology, Rhodes University, Grahamstown 6140, South Africa

Triple negative breast cancers (TNBCs) lack estrogen (ER), progesterone (PR) and human epidermal growth factor (HER-2) receptors which renders many anticancer agents, targeting these receptors ineffective. New cancer chemotherapeutics targeting TNBCs are thus needed. Sargaquinoic acid (**2**), a marine natural product produced by several marine algae of the genus *Sargassum*, displayed an IC_50_ of 14.71 μM against MDA-MB-231 cells. This promising activity provided an opportunity to design analogs with improved activity. In this study we designed and synthesised a series of diketopiperazine (**1**) and barbituric acid (**3**) derivatives as analogs of sargaquinoic acid (Figure 13). 

A series of diketopiperazine and barbituric acid derivatives were synthesised in good yield, via Aldol condensation with various aldehydes. All compounds were fully characterized by NMR, HRMS and IR spectroscopy. 

A preliminary assessment of cytotoxicity of the compounds was evaluated at 25 μM concentration against HCC70 cells. The most active compounds were further evaluated against HeLa, MDA-MB-231, HCC70 and MCF12A cells. These compounds showed no improvement over SQA in these assays. 

#### **The Advanced Floating Chirality Distance Geometry Approach—How Anisotropic NMR Parameters Can Support the Determination of the Relative Configuration of Natural Products** 

KöckMatthias^1^,^2^ImmelStefan^3^ReggelinMichael^3^1Alfred-Wegener-Institut für Polar- und Meeresforschung in der Helmholtz-Gemeinschaft (AWI), Am Handelshafen 12, 27570 Bremerhaven, Germany2Helmholtz-Zentrum für Infektionsforschung (HZI), Inhoffenstr. 7, 38124 Braunschweig, Germany3Clemens-Schöpf-Institut für Organische Chemie und Biochemie, Technische Universität Darmstadt, Alarich-Weiss-Straße 4, 64287 Darmstadt, Germany

The determination of the relative and absolute configuration of natural products is essential to understand their interactions in the biological field and to allow their procurement through total synthesis. But so far, there is no general NMR method for a secure assignment of the relative or even the absolute configuration of non-crystallizable natural products. The method of choice for small molecules with several stereogenic centers is the combination of distance geometry (DG) and distance bounds driven dynamics (DDD) calculations using NOE/ROE-derived distance restraints (r). The most important aspect of the NOE/ROE-restrained DG/DDD method (fc-rDG/DDD) is to run the sampling of structures in four-dimensional space including the possibility for the configurations to change during the simulation (floating chirality, fc) and therefore to determine the conformation AND the relative configuration of small organic molecules simultaneously. A general application of this approach to all different kind of natural products was hindered by the fact that NOEs/ROEs cover only short-range interactions (up to 400 pm for small molecules), and is hampered or even impossible for proton-deficient structures. This can be overcome by the use of anisotropic NMR parameters (RDCs, RQCs, and RCSA) in the structure under investigation. In contrast to NOEs/ROEs, these anisotropic parameters are global in nature and independent from the distances between nuclei. All these NMR observables can now be used within the fc-rDG/DDD method using the recently developed software ConArch+ [1,2].

References

1. Immel, S.; Köck, M.; Reggelin, M. Configurational analysis by residual dipolar coupling driven floating chirality distance geometry calculations. *Chem. Eur. J.*
**2018**, *24*, 13918–13930
2. Immel, S.; Köck, M.; Reggelin, M. Configurational analysis by residual dipolar couplings: A critical assessment of diastereomeric differentiabilities. *Chirality*
**2019**, *31*, 384–400.

#### **Designing New Agents for the Peloruside Binding Site** 

WoollyEthanTeesdale-SpittelPaulHarveyJoanneSchool of Chemical and Physical Sciences, also Centre of Biodiscovery, Maurice Wilkins Centre for Molecular Biodiscovery, Victoria University of Wellington, Wellington 5043, New Zealand

The marine natural product peloruside A (Figure 14) has potential for anticancer application due to its potent cytotoxicity and its ability to kill cancer cells through disruption of microtubules dynamics. However, due to the low natural abundance and its structural complexity its application has been limited. Therefore, it is desirable to produce analogues of peloruside A that are more synthetically accessible while retaining the potency of the parent compound. Pharmacophores of peloruside A that have been identified to interact with the microtubules will be linked via scaffolding regions. These analogues will be tested for their biological activity and this will then guide the development of second generation analogues. This presentation will highlight the research undergone so far.

#### **Bioactive Metabolites from an Actinobacterial Strain *Streptomyces smyrnaeus*** 

HarizaniMaria^1^MacedoSara Costa^2^SantaremNuno^2^Cordeiro-da-SilvaAnabela^2^,^3^RoussisVassilios^1^IoannouEfstathia^1^1Section of Pharmacognosy and Chemistry of Natural Products, Department of Pharmacy, National and Kapodistrian University of Athens, 15771 Athens, Greece2I3S—Instituto de Investigação e Inovação em Saúde, Universidade do Porto, 4200-135 Porto, Portugal3Department of Biological Sciences, Faculty of Pharmacy, University of Porto, 4050-313 Porto, Portugal

Marine-derived microorganisms receive constantly increasing attention as sources of new bioactive natural products. In the framework of our investigations on the chemistry of marine microorganisms, a rich microbank containing several hundred bacterial strains isolated from marine sediments and macroorganisms collected in the Eastern Mediterranean Sea has been created. The actinobacterial strain BI0918, isolated from a marine sediment collected at Kefalonia island and identified as *Streptomyces smyrnaeus*, was selected for further investigation due to its interesting chemical profile. Its large-scale organic extract was submitted to a series of chromatographic separations, leading to the isolation of a large number of secondary metabolites. The structures of the isolated compounds, among which several are new natural products, were determined on the basis of extensive analysis of their 1D and 2D NMR and MS data. Some of the isolated compounds were evaluated for their in vitro antiparasitic activity against bloodstream forms of *Trypanosoma brucei* and intracellular amastigotes of *Leishmania infantum*, as well as cytotoxicity against THP-1 macrophages. The results of the bioactivity evaluation were promising, as one of the metabolites exhibited higher activity levels and selectivity index compared to the reference drug. 

##### **Acknowledgments** 

The authors thank Special Account for Research Grants and National and Kapodistrian University of Athens for funding to attend the meeting. 

##### **Discovery, Synthesis, and Biological Activity of New Cyanobacterial Natural Products from the South China Sea** 

FangFang^1^LiTe^1^YuYiyi^2^CaoZhengyu^2^ZhangWeiyan^1^ZhangBin^1^YuanYe^1^DingLijian^1^HeShan^1^NamanC. Benjamin^1^,^3^1Li Dak Sum Marine Biopharmaceutical Research Center, College of Marine Sciences, Ningbo University, Ningbo 315211, China2Department of Traditional Chinese Medicine Pharmacology, School of Chinese Traditional Pharmacy, China Pharmaceutical University, Nanjing 210029, China3Scripps Institution of Oceanography, University of California, La Jolla, CA 92093, USA

A marine bioprospecting sample collection trip was conducted around the Paracel Islands in the South China Sea during early 2017 by several of the coauthors, with the permission of the local Chinese government and military entities. This island chain is under tight military restriction on who can visit or utilize the maritime resources, so the marine natural products that result from investigations of this niche ecosystem are accordingly considered to be very precious. The biomasses of 31 collected samples were extracted and then analyzed by LC-MS and LC-MS/MS with untargeted dependent scans. The data for each were analyzed by Global Natural Products Social Molecular Networking (GNPS). The resulting sets of node clusters and molecular families suggested that some groups likely contain new and potentially unique molecules, and this provided the sample prioritization and testable hypothesis for target-driven discovery research. One extract tentatively identified as a cyanobacterium of genus *Oscillatoria* was accordingly selected for further study. Targeted isolation efforts yielded the new natural products paracelamide A (**1**) and B (**2**). The characterization of these compounds will be reported, as well as the total synthesis and biological activity testing results for each, and ongoing research efforts from other samples collected around the Paracel Islands.

### ***Theme 6. Marine Natural Products Chemical Biology and Biotechnology*** 

#### **Defining Patterns in Chemical and Genomic Space to Prioritise Antibiotic Discovery from Actinobacteria** 

SoldatouSylvia^1^EldjarnGrimur Hjorleifsson^2^Huerta-UribeAlejandro^3^van der HooftJustin J. J.^4^RogersSimon^2^DuncanKatherine R.^3^1Department of Chemistry, University of Aberdeen, Aberdeen AB24 3EU, UK2School of Computer Science, University of Glasgow, Glasgow G12 8RZ, UK3Strathclyde Institute of Pharmacy and Biomedical Sciences, University of Strathclyde, Glasgow G4 0RE, UK4Department of Bioinformatics, Wageningen University, 6700 HB Wageningen, The Netherlands

Microbial drug discovery in the ‘omics era relies on three key datasets, biosynthetic, chemical and biological (activity). Yet, to integrate and interrogate these large and complex datasets remains a challenge and results in the low-throughput prioritization of only a few strains based on observed antibiotic activity. Despite this wealth of genomic and metabolomic data, linking metabolites to the BGC responsible for their production and to observed bioactivity is limited, slow (manual) and challenging. Furthermore, our current discovery is reliant on existing biosynthetic, chemical and antibiotic knowledge, thus overlooking unidentified parent ions (metabolites) or hypothetical proteins (BGCs) which is the exact chemical and biosynthetic space which should be prioritized to identify novel antibiotics. Here, we have generated two datasets of Actinobacterial strains to combine bacterial genomes (and their predicted BGCs) their chemical profiles (via strain fermentation and comparative metabolomics by molecular networking of the metabolite extracts) and antibiotic screening (against a panel of clinically relevant pathogens). The first dataset consists of 25 Polar rare-actinomycete genomes with almost 200 BGCs, combined with 100 metabolite profiles (each strain under four fermentation conditions). The second dataset consists of 10 *Streptomyces* genomes and four rare-actinomycete genomes with almost 500 BGCs, combined with 80 metabolite profiles (each strain under five fermentation conditions). Both datasets will be discussed in relation to the machine learning tools we have developed to establish patterns across strains and learn relationships between BGC, spectral features and bioactivity. 

#### **The Evolutionary Profile of a Superproducer Sponge Symbiont, *Candidatus* “Entheonella”** 

CahnJackson K.B.^1^PetersEike E.^1^LottiAlessandro^1^FieldChristopher^1^RückertChristian^2^KalinowskiJörn^2^SunagawaShinichi^1^TakeyamaHaruko^3^PielJörn^1^1Institute of Microbiology, Eidgenössische Technische Hochschule (ETH), 8093 Zürich, Switzerland2Center for Biotechnology, Universität Bielefeld, 33615 Bielefeld, Germany3Center for Advanced Biomedical Sciences, Waseda University, Tokyo 162-8480, Japan

Sponges and other marine invertebrates, long known as valuable sources of bioactive natural products, often rely on microbial symbionts for the biosynthesis of these molecules. The co-evolution of these ancient host animals with their microbiomes influences the distribution and diversity of biosynthetic systems, and is therefore an important consideration in the search for chemical novelty in nature. In some sponges, biosynthetic potential is divided between numerous symbionts, but in the Theonellidae sponge family nearly all known natural products have been attributed to a handful of members of a single uncultivated bacterial genus, *Candidatus* “Entotheonella”. Using a combination of single-cell sequencing and metagenomics, we have obtained the genomes of more than a dozen “Entotheonella” from a taxonomically and geographically diverse set of sponges. All possess rich and distinct complements of biosynthetic gene clusters, providing snapshots of the rapid divergent evolution of secondary metabolism. Unlike in other recently characterized producers of sponge-associated compounds we see no evidence of adaptive genome reduction, nor of metabolic niche differentiation between co-symbionts. Additionally, while we observe a general trend of co-evolution between host and symbiont, exceptions exist which suggest instances of horizontal symbiont transfer. A better understanding of the global and phylogenetic distribution of symbiont-produced specialized metabolites will assist in the mining of this valuable resource; in this context, these findings provide a detailed case study of the evolution of a biosynthetically talented microbial superproducer genus. 

#### **Marine Fungi from Sponges: Biodiversity, Chemodiversity and Biotechnological Applications** 

BovioElena^1^PoliAnna^1^GarzoliLaura^1^LuganiniAnna^2^VillaPietro^3^MusumeciRosario^3^FauchonMarilyne^4^CocuzzaClementina Elvezia^3^GribaudoGiorgio^2^HellioClaire^4^MehiriMohamed^5^VareseGiovanna Cristina^1^1Mycotheca Universitatis Taurinensis—DBIOS, University of Turin, 10125 Turin, Italy2Laboratory of Microbiology and Virology—DBIOS, University of Turin, 10123 Turin, Italy3Laboratory of Clinical Microbiology and Virology—Department of Medicine, University of Milano-Bicocca, 20900 Monza, Italy4Biodimar/LEMAR, 29200 Brest, France5University Nice Côte d’Azur, CNRS, ICN, UMR 7272, Marine Natural Products Team, 06108 Nice, France

Marine fungi represent one of the most understudied groups of microorganisms. Special attention deserve fungi associated with sponges: it is becoming more and more evident that they interact with sponges by producing secondary metabolites that could be involved in the host defence against predators, pathogens and fouling organisms. From a biotechnological point of view, the molecules isolated from these fungi showed interesting pharmaceutical properties and environmental applications. Thus, the aim of this work was (i) to isolate and identify the fungal community associated with four Atlantic sponges; (ii) to stimulate the production of secondary metabolites in fungi, using the OSMAC approach (One Strain—Many Compounds); (iii) to evaluate the biotechnological applications of the fungal molecules. 

Sponges revealed an astonishing biodiversity: 97 fungal taxa were isolated. 

The fungal community (20 taxa) associated with the sponge *Grantia compressa* was further studied for the production of secondary metabolites. Ten pure compounds were isolated from the fungus *Eurotium chevalieri* MUT 2316. Three molecules were alternatively able to completely inhibit the replication of Herpes simplex 1 virus and Influenza A virus; Six out of ten compounds showed antibacterial activity also against emergent pathogens. 

The molecules produced by *E. chevalieri* were investigated also for the antifouling properties. The tested compounds were able to inhibit both the growth and adhesion of several marine bacteria and microalgae. 

Fungi inhabiting sponges confirmed to be an outstanding source of interesting compounds that can have a direct impact on human life or contribute to environmental preservation. 

#### **Marine Derived Molecules and Their Potential Use in Cosmeceutical and Cosmetic Products** 

LetsiouSophiaGardikisKonstantinosEU-H2020 TASCMAR ConsortiumDepartment of Research and Development, APIVITA SA, Industrial Park of Markopoulo, 19003 Markopoulo Mesogaias, Greece

The oceans host a huge biodiversity, with more 250,000 species described, up to 8.5 million species still to be discovered as well as a microbial diversity which is largely unknown. Recently, the exploration of the oceans has allowed the discovery of a horde of previously unknown habitats living under extreme conditions. These environments host a variety of organisms adapted to these conditions and producing a wide range of active biomolecules. More than 25,000 new biologically active compounds have been identified in the past fifty years, with an increment of 5% per year and 1378 new molecules identified in 2014 alone. Among marine organisms, microorganisms, including fungi and bacteria, have attracted great attention as potential leading compound producers. 

On the other hand, nowadays, there is a huge interest on natural products obtained from marine organisms that can promote the state of health and well-being for humans. The global market for cosmetics and cosmeceutical products is forecasted to grow at a rate of 4.3% by 2022 with a value of USD 43 billion. Photo-protective, skin-care, and hair care products drive this trend of increasing demand. 

Here we present an overview of in vitro effects of marine-based molecules on primary normal human dermal fibroblasts (NHDF), as source of photo-protective, moisturizing, anti-wrinkle, skin-whitening products as well as other commonly active ingredients included in the synthesis of cosmeceuticals.

#### **In Vitro Bioactive Potential and Identification of Molecules Isolated from the Muscle of Farmed (*Litopenaeus vannamei*) and Wild (*Litopenaeus stylirostris*) Shrimp: Anti-Proliferative/Pro-apoptosis Activity and Protection against Oxidative Cellular Damage/Injury** 

García-RomoJoel Said^1^Hernandez-ZazuetaMartin Samuel^1^Galvez-IriquiAlma Carolina^1^Cruz-RamirezSusana Gabriela^1^Plascencia-JatomeaMaribel^1^Burboa-ZazuetaMaría Guadalupe^2^Sandoval-PetrisEdgar^2^Robles-SánchezRosario Maribel^1^Júarez-OnofreJosué Elías^3^López-SaizCarmen María^1^Hernández-MartínezJavier^4^Santacruz-OrtegaHisila^5^Burgos-HernándezArmando^1^1Department of Research and Postgraduate in Food, University of Sonora, Sonora 83000, Mexico2Department of Scientific and Technological Research, University of Sonora, Sonora 83000, Mexico3Department of Physics, University of Sonora, Sonora 83000, Mexico4Support Services Unit in Analytical Resolution, Universidad Veracruzana, Veracruz 91190, Mexico5Department of Research in Polymers and Materials, University of Sonora, Sonora 83000, Mexico

Cancer is one of the main causes of mortality worldwide and one way to stop it is through anti-cancer drugs. Seventy percent of all approved drugs have been isolated of natural source and it’s estimated that 1% of marine organism show anti-cancer properties. These data become even more interesting when these drugs are found in marine organisms used as food. Such is the case of shrimp, popular seafood worldwide. The aim of the present study was obtained from the muscle of farmed (*Litepenaeus vannamei*) and wild (*L. stylirostris*) shrimp, compounds with anti-proliferative/pro-apoptotic activity and potential of protecting cells against oxidative damage/injury. The isolation of bioactive compounds was carried out using chromatographic (open column, TLC) and their characterization using spectrophotometric (UV-Vis, fluorescence, FT-IR, ESI-MS, ^1^H and ^13^C RMN) techniques. Biological assays were complemented with colorimetric (spectrophotometer) and fluorometric (microscopy and flow cytometry) techniques. Results suggest that a single molecule present in a fraction obtained from both types of shrimp showed the highest values for anti-proliferative/pro-apoptosis activity against prostate cancer cell and evidence damage to DNA and F-actin polymerization, suggesting cellular collapse. This fraction also exhibited high antioxidant indicator activity was assayed by the ABTS, DPPH, and FRAP techniques. In addition, it showed protective effects against red blood cell-hemolysis induced by AAPH free radical and retinal pigmented epithelium cell, suggesting capability of protecting cell against H_2_O_2_-induced death, underlying protective mechanism against H_2_O_2_-induced injury. These suggest that this compound might be proposed for further investigation as possible chemotherapeutical and chemoprotection agent. 

#### **Radiosensitization of Maligant Cancer Cells by Polysaccharides from Brown Seaweed *Fucus evanescens*** 

MalyarenkoOlesyaSilchenkoArtemErmakovaSvetlanaRussian Academy of Sciences, Far Eastern Branch, G.B. Elyakov Pacific Institute of Bioorganic Chemistry, 690022 Vladivostok, Russia

Oncological diseases are of huge significance to human health. Radiation therapy is widely used to treat almost all types of cancers. One of the limitations of radiotherapy is the presence of radio-resistance of tumors and radio-toxicity to normal cells and systems. Therefore, the problem of increasing the effectiveness of radiotherapy acquires an important social and economic importance and requires constant attention and finding the best ways to solve it.

Numerous in vitro and in vivo studies have shown that natural compounds, especially of marine origin, could be perspective candidates for chemoprevention and therapy of different malignancies. Water-soluble polysaccharides of brown seaweeds, laminarans and fucoidans, are of the greatest interest due to their anticancer, anticoagulant, immunostimulatory, antivirus, radioprotective, and antioxidant activities without toxicity for organisms.

Our data provided evidence that laminaran and fucoidan isolated from brown seaweed *F. evanescens* exhibit significant anticancer activity against human melanoma, colon and breast cancer cells. The fucoidan was able to selectively sensitize cancer cells to X-ray irradiation, resulting in significant inhibition of cell proliferation, colony formation, and cancer cells’ migration. The molecular mechanism of this action was associated with the suppression of the phosphorylation of the mitogen activated protein kinases and the induction of apoptosis by activating the initiator and effector caspases, down-regulation of the anti-apoptotic protein expression, followed by the enhancing of DNA fragmentation. Moreover both investigated polysaccharides were found to possess radioprotective effect in vitro and in vivo. 

##### **Acknowledgments** 

This work was funded by the RFBR Grant # 18-34-20013.

## Poster Session 1: Chemical Ecology and Ecosystem Functioning, and Isolation and Structure Elucidation of Marine Natural Products 

### ***Theme 1. Chemical Ecology and Ecosystem Functioning*** 

#### **Comparative Studies of the Two Sea Cucumber Genera *Bohadschia* and *Actinopyga*: Biological Activities and Structural–Activity Differences** 

KamyabElham^1^KellermannMatthias^1^RohdeSven^1^SteinmannEike^2^KöckMatthias^3^MohrKathrin^3^SchuppPeter J.^1^,^4^1Institute for Chemistry and Biology of the Marine Environment, Carl-von-Ossietzky University Oldenburg, Schleusenstraße 1, 26382 Wilhelmshaven, Germany2Institute of Experimental Virology Group Virus Transmission (TWINCORE), Centre for Experimental and Clinical Infection Research, 30625 Hannover, Germany3Hemholtz-Zentrum für Infektionsforschung GmbH (HZI), Inhoffenstraße 7, 38124 Braunschweig, Germany4Helmholtz Institute for Functional Marine Biodiversity at the University of Oldenburg (HIFMB), Ammerländer Heerstrasse 231, D-26129 Oldenburg, Germany

Marine organisms provide a large diversity of Natural Products (NPs) with unique structures and functions that increase organisms’ survival and fitness. Sea cucumbers are slow moving organisms that do not have significant escape behaviour. Although some morphological defence mechanisms have been reported, they rely mainly on their chemical defences to deter predators or competitors. 

In this study we analysed the chemical defense of 5 holothurian in two genera, Bohadschia (i.e., *Bohadschia argus*, *Bohadschia vitiensis, Bohadschia* sp.) and Actinopyga (i.e., *Actinopyga echinites*, *Actinopyga mauritiana*). We used an activity-guided fractionation in *B. argus* to elucidate the structural-activity relationships of saponins. Crude organic extracts of different genera of family Holothuriidae showed that Actinopyga and Bohadschia contained high concentrations of saponins. However, while all species deterred feeding of the puffer fish Canthigaster solandri, Bohadschia extracts demonstrated significantly higher feeding deterrence compared to Actinopyga and also had higher anti-bacterial activities against (non) pathogenic bacteria. Furthermore, Bohadschia extracts (mainly from *B. argus*) showed higher cytotoxicity based on a brine shrimp assay, although the anti-fouling activities of both holothurid genera against the benthic diatom *C. closterium* were similar. Separation of the crude extract of *B. argus* by column chromatography yielded a series of active fractions against the Hepatitis Virus C (HCV), the bacteria Rhodococcus glutinis, Staphylococcus aureus, and Mucor hiemalis and the fungus *Candida albicans*. To detect and describe the structure-activity relationships of saponins, purification and identification of the active and non-active fractions are currently ongoing using various methods of high performance liquid chromatography (HPLC), high resolution liquid chromatography mass spectrometry (LC/MS) and nuclear magnetic resonance (NMR). We emphasize that a combination of biological and chemical screenings are an effective approach for selecting bioactive organisms to discover pharmacologically active natural products.

#### **Location Explains Variation in the Metabolic Production of the Marine Sponge *Xestospongia* sp.** 

BayonaLina M.^1^LeeuwenGemma van^1^ErolÖzlem^1^SwiertsThomas^2^Van Der EntEsther^2^VoogdNicole de^2^,^3^ChoiYoung Hae^1^,^4^1Natural Products Laboratory, Institute of Biology, Leiden University, 2333 BE Leiden, The Netherlands2Marine Biodiversity, Naturalis Biodiversity Center, 2333 CR Leiden, The Netherlands3Global Change and Marine Ecosystems, Institute of Environmental Sciences, Leiden University, 2300 RA Leiden, The Netherlands4College of Pharmacy, Kyung Hee University, Seoul 02447, Korea

The giant barrel sponge (*Xestospongia* sp.) has been widely studied due to their biological and chemical importance. From an ecological perspective, their relatively large size allows them to play an essential role in providing resources and protection to other organisms in the reef. Additionally, the wide range of chemical compounds that have been isolated from the sponge, such as terpenoids, alkaloids, brominated fatty acids, and sterols, have attracted significant attention from the scientific community. 

Unlike other sponges, the giant barrel sponge can be found in a broad geographical range; they are situated from the red sea to the Indo-Pacific Ocean and Australia as *X. testudinaria* and in the Atlantic Ocean as the sister species *X. muta*. However, recent studies have shown the presence of cryptic species, and specimens found in the same location are not necessarily genetically related. 

In this study, to correlate geographical location and metabolic variation, 139 specimens of *Xestospongia* sp., collected in four different locations: Martinique, Curacao, Taiwan, and Tanzania were investigated from a holistic approach. Using a multiplatform metabolomics methodology (NMR, LC-MS, and HPTLC), we aimed to gain insight into the metabolome of samples collected in different locations. It was found that samples collected in each location displayed characteristic metabolites and based on their chemical fingerprint it was possible to group the samples according to their geographical location. This indicates that environmental factors are a driving force in the production of metabolites and they can be just as important as genetic factors. 

#### **A Chemical and Ecological Approach Sheds Light on the Urticating System of Marine Fireworms** 

RighiSara^1^,^2^SavioliMartina^2^FortiLuca^1^PrevedelliDaniela^1^SimoniniRoberto^1^1Department of Life Sciences, University of Modena and Reggio Emilia, 41125 Modena, Italy2Department of Chemical and Geological Sciences, University of Modena and Reggio Emilia, 41125 Modena, Italy

Marine fireworms (Annelida, Amphinomidae) hold stinging dorsal bristles (chaetae) that cause injuries to divers and bathers. *Hermodice carunculata* is the most notorious species and it has recently attracted interest as a potentially invasive fireworm with few predators and uncharacterized defensive capacities. To date, the only acute inflammation inducer isolated from an amphinomid is “complanine”, a trimethylammonium compound. The main goal of this study was to promote an ecological understanding of *H. carunculata* defences through a multidisciplinary approach. The occurrence of complanine within tissues and its mode of delivery were assessed combining chromatographic steps and high resolution LC-MS/MS. The exact mass and retention time of complanine were detected in the extracts of the main ectodermal (dorsal body wall, gills, dorsal chaetae, ventral chaetae) and endodermal (gut, pharynx) tissues of *H. carunculata*. The role of complanine in trophic interactions was assessed offering the ectodermal tissues towards a predator (the fish *Chromis viridis*) and two relevant prey species (the anemones *Anemonia viridis* and *Aiptasia diaphana*). Only the dorsal chaetae were effective against predators and prey: they strongly deterred fishes and induced paralysis in the anemone tentacles. Dorsal chaetae treated with organic solvents lost their deterrence against fish predators and an inner hollow cavity suitable to vehicle toxins could be viewed by ESEM. These findings support a synergy between the mechanical injury of dorsal chaetae penetration and the release of complanine. This unique feature could support the success of fireworms in marine benthic environments and significantly improves knowledge on the chemical ecology of amphinomids. 

#### **Can Trait Evolution and Phylogenetics Predict the Discovery of New Toxins from Marine Invertebrates?** 

MadeiraCarolina^1^,^2^GonçalvesCátia^1^LoboJorge^3^CostaPedro M.^1^1UCIBIO—Applied Molecular Biosciences Unit, Departamento de Ciências da Vida, Faculdade de Ciências e Tecnologia, Universidade NOVA de Lisboa, 2829-516 Caparica, Portugal2MARE—Marine and Environmental Sciences Centre, Departamento de Biologia Animal, Faculdade de Ciências da Universidade de Lisboa, 1749-016 Lisboa, Portugal3Instituto Português do Mar e da Atmosfera, I.P. (IPMA), Rua Alfredo Magalhães Ramalho, 6, 1495-006 Lisboa, Portugal

Due to their extraordinary biodiversity, marine invertebrates can become one of the most important sources of novel bioactive compounds (BCs), especially toxins. As recent incentives to blue biotechnology have led to a significant increase in bioprospecting efforts in the marine realm, there is a need to improve our ability to predict the distribution of such BCs along the marine animal tree-of-life. We envisage that evolutionary ecology offers solutions for this grand challenge by integrating systematics, life-history traits and the secretion of specialised chemical compounds into phylogenetic models. Using intertidal Polychaeta from the Portuguese coast as a case study, our research aimed at predicting BCs occurrence in these marine invertebrates. Using Bayesian inference-based phylogenetics we combined: (i) molecular systematics (through COI gene sequencing) with (ii) morphoanatomical features (presence vs absence of tentacles in the proboscis and/or of mandibulae and venom glands) and (iii) ecological characteristics (trophic level) to construct a phylogenetic tree to identify potentially venom-producing polychaetes. Results allowed us to map several families within Order Phyllodocida, such as Glyceridae, Phyllodocidae, Nephtyidae and Nereidae that have evolved to produce complex toxin mixtures as defence or predation strategies. Harvesting secretions from mucus and glands, plus toxicity testing with natural prey (e.g., mussels) assisted validation of findings by disclosing tissue and cell-level toxicopathological alterations like cell death and DNA damage. Altogether, frontline bioinformatics and multi-trait phylogenetics can be combined into powerful tools to locate new species of interest secreting BCs, therefore contributing to turn marine bioprospecting for biotechnological purposes from random to systematic. 

##### **Acknowledgments** 

“Fundação Para a Ciência e Tecnologia” (project WormALL—PTDC/BTA-BTA/28650/2017) and UCIBIO (strategic project—UID/Multi/04378/2019) for funding this research.

#### **Toxin Profile of *Ostreopsis* cf. *ovata* from Continental Portuguese Coast and Selvagens Islands (Madeira, Portugal)** 

SoliñoLucía^1^,^2^García-AltaresMaría^3^GodinhoLia^1^SilvaAlexandra^1^CostaPedro Reis^1^,^2^1IPMA—Instituto Português do Mar e da Atmosfera, Rua Alfredo Magalhães Ramalho, 6, 1495-006 Lisbon, Portugal2CCMAR—Centre of Marine Sciences, University of Algarve, Campus of Gambelas, 8005-139 Faro, Portugal3Department of Biomolecular Chemistry, Hans-Knöll-Institut (HKI), Adolf-Reichwein-Straße 23, 07745 Jena, Germany

The toxigenic dinoflagellate *Ostreopsis* cf. *ovata* is known to produce a range of palytoxin (PLTX)—related compounds named ovatoxins (OVTX). *O. cf ovata* presents a wide variability in toxin production and its toxic profile is strain-specific. Several OVTX, denominated from -a to -l have been reported from different strains of this benthic microalgae up to now, mainly in Mediterranean isolates. However, less is known about the toxin profile of the strains present in the Atlantic coasts of Europe. In this work, two strains of toxigenic *O. cf ovata* isolated from the South coast of Portugal mainland (Algarve) and Selvagens Island (Madeira, Portugal) were cultured and tested for toxicity by haemolytic assay. Toxin profiles were qualitatively elucidated by Liquid Chromatography-High Resolution Mass Spectrometry (LC-HRMS). The strain from Algarve presented lower toxic potency than the strain from Selvagens island (12.29 against 54.79 pg of PLTX equivalents per cell) showing in both cases the characteristic toxin profile of Mediterranean strains. The major component, OVTX-a, was concomitant with OVTX from -b to -g and isobaric PLTX. Regarding the morphological and molecular characteristics of both strains as well as their toxin fingerprint, it is likely that both strains are closely related to those from Mediterranean coasts. The present study reports for the first time the occurrence of several OVTX congeners and iso-PLTX in *O. cf ovata* from Portuguese waters. Further research on quantitative toxin production of these and newly isolated strains are currently ongoing, to characterize the risk of OVTXs- related outbreaks in Portugal. 

#### **Mesophotic Marine Habitats: Inspiring Understudied Biodiversity** 

BenayahuYehuda^1^ShohamErez^1^LibermanRonen^1^TamirShai^1^AlonsoCarolina^2^ÁlvarezPedro^2^ChavanichSuchana^3^BialeckiAnne^4^Le GoffGéraldine^5^OuazzaniJamal^5^1Tel Aviv University, 69978 Tel Aviv, Israel2iMare Natural, 18600 Granada, Spain3Chulalongkorn University, Bangkok10330, Thailand4Université de la Réunion, 97715, Ile de la Réunion, France5ICSN-CNRS, 91198 Gif sur Yvette, France

Studies have revealed the bewildering diversity on shallow reefs, comprising plethora of invertebrates. Until the past decade most biodiversity surveys have been restricted to the upper ~30 m. The mesophotic coral-reef ecosystem (MCE) has been defined as comprising the light-dependent communities (30 to <150 m) in tropical and subtropical regions. Remotely-operated vehicles (ROVs) and technical diving have now facilitated the investigation of MCEs. Consequently, they have become available for research with an increasing interest in their yet unexplored bio-resources, keeping in mind holobiont concept. The scarce data available on non-scleractinian MCE fauna in the Red Sea, Andaman Sea, Gulf of Thailand and the Western Mediterranean Sea intrigued us to conduct thorough surveys on the MCE fauna for bioprospecting purposes. The results revealed diverse species assemblages associated with a variety of micro-symbionts, including species new to science and new zoogeographical records. The findings highlight the possibility that MCEs host depth generalists, along with unique depth specialists uniquely found in MCEs. In addition, this ecosystem might include species also found below the deepest fringes of the MCEs. The evidence suggests that octocorals, sponges along with other invertebrates are the major benthic organisms in MCE, being far more diverse than has been envisioned. The results also raise pressing issues concerning the importance of conservation policies aiming at protecting the MCE biodiversity and its possible function as refugia for impoverished shallow reef habitats. 

##### **Acknowledgments** 

TASCMAR project (www.tascmar.eu) is funded by the European Union in the frame of H2020 (GA No 634674). 

#### **Strategic Defense Potential of a Sponge Associated with Sandy Bottom in the Western South Atlantic Ocean** 

FleuryBeatriz G.^1^,^2^SilvaAmanda G.^1^,^2^LopesAna Lea D.^1^AmorimCarolina G.^1^AraujoJuliana M.^1^,^2^BragaTamires S. R.^1^AmaralVictor R.^1^VançatoYollanda C. S. F.^1^,^2^1Departamento de Ecologia, IBRAG, Universidade do Estado do Rio de Janeiro, PHLC sala 220, Rua São Francisco Xavier 524, Maracanã, Rio de Janeiro 20550-900, Brazil2Programa de Pós-Graduação em Ecologia e Evolução, IBRAG, Universidade do Estado do Rio de Janeiro, Rio de Janeiro 20550-013, Brazil

Porifera evolved different strategies, physical and chemical, to defend themselves against threats from their environment. They produce biologically functional natural products, such allelopathic substances and other chemical defenses against predation, fouling and pathogens. However, few studies of the different sponge defense strategies have been carried out to date in the South Atlantic Ocean. The aim of this study was to investigate the chemical and physical defenses responses of the abundant *Iotrochota arenosa* (Demospongiae) from Ilha Grande Bay, RJ-Brazil. The interactions between *I. arenosa* and invasive *Tubastraea* corals was evaluated, in situ, with competition trials using physical barriers to separate chemical and physical effects. The antifouling activity of mucus and organic extracts were tested in the lab through substrate preference (treatment and filter paper control) in the ‘mussel test’. Anti-predation bioassays against the crab *Pachygrapsus transversus* used mucus, extracts and powdered sponge, added separately to artificial food based on agar+squid (treatments). The competition results indicated that *I. arenosa* uses physical and chemical mechanisms of defense, to surround and later override the invasive *Tubastraea*, probably causing choking. The antifouling results indicated that *I. arenosa* mucus was more efficient (*p* = 0.047) to inhibit the production of byssal threads. Deterrent activity was effective with powdered sponge, mucus and extract (*p* = 0.01; *n* = 23). This work is unprecedented in the defensive evaluation *of I. arenosa*, suggesting that the physical and chemical weapons may be driving the observed success of this sponge in the environment.

#### **Bioactive Metabolites from the Algal Garden of the Limpet *Scutellastra mexicana*** 

de los ReyesCarolina^1^YáñezBenjamín^2^CarballoJ. Luis^2^ZubíaEva^1^1Departamento de Química Orgánica, Facultad de Ciencias del Mar y Ambientales, Universidad de Cádiz, 11510-Puerto Real/Cádiz, Spain2Instituto de Ciencias del Mar y Limnología, Universidad Nacional Autónoma de México, Apdo. 811-Mazatlan, Sinaloa 82000, Mexico

The giant limpet *Scutellastra mexicana* is the only known true patellid living on tropical east Pacific coasts, from Mexico to Peru, and nowadays is among the most endangered marine invertebrates [1]. *S. mexicana* lives on rocky substrates in the shallow sublittoral, showing homing and algal gardener behaviour. Thus, each limpet creates a home scar on the rock where returns after feeding and around that scar grows an algal garden of about 5–10 cm length [2].

Although limpet foraging has been the topic of extensive scientific research [3], there are no data on the involvement of chemical compounds in the relationship between the limpet and the algae growing around. Interestingly, no other invertebrate seems to invade the algal garden around each limpet, which suggests a deterrent role of the algae. 

As a part of our ongoing multidisciplinary approach to the study of the threatened species *S. mexicana*, we have performed a chemical analysis of the algal garden of this limpet, aimed to determine the presence of bioactive metabolites. 

Samples of the algae were collected from rocks populated with *S. mexicana* at Maria Cleofas Island (Pacific Ocean, Mexico). The algal extract was subjected to different chromatographic separation steps, allowing the isolation of a series of compounds whose structures have been determined by NMR and MS analysis. From a biological/ecological point of view, the isolated compounds are endowed with osmoregulatory, antifeeding or antifouling properties. 

These results represent the first characterization of natural compounds produced by the algae thriving around *S. mexicana* that could have a key role in the chemical defence of this giant limpet. 

References

1. Esqueda, M.C.; Ríos-Jara, E.; Michel-Morfín, J.E.; Landa-Jaime, V. Vertical distribution and diversity of gastropods molluscs from intertidal hbitats of the Ratnagiri Coast Mahararashtra, India. *Rev. Biol. Trop*. **2000**, *48*, 765–775.
2. Espinosa, F.; Rivera-Ingraham, G.A. Biological Conservation of Giant Limpets: The Implications of Large Size. *Adv. Mar. Biol*. **2017**, *76*, 105–155.
3. Burgos-Rubio, V.; De la Rosa, J.; Altamirano, M.; Espinosa, F. The role of patellid limpets as omnivorous grazers: a new insight into intertidal ecology. *Mar. Biol*. **2015**, *162*, 2093–210.

#### **Benthic Cyanobacteria from Tropical Mangroves as Producers of Antimicrobials** 

DuperronSébastien^1^,^2^BeniddirMehdi A.^3^DurandSylvain^1^LongeonArlette^1^DuvalCharlotte^1^GrosOlivier^4^BernardCécile^1^Bourguet-KondrackiMarie-Lise^1^1Molécules de Communication et Adaptation des Microorganismes, UMR 7245 MCAM, Muséum National d’Histoire Naturelle, 57 rue Cuvier (CP 54), 75005 Paris, France2Institut Universitaire de France, 75005 Paris, France3Équipe “Pharmacognosie-Chimie des Substances Naturelles” BioCIS Univ. Paris-Sud, CNRS, Université Paris-Saclay 5 rue J.-B. Clément, 92290 Châtenay-Malabry, France4UMR 7205 ISYEB et Université des Antilles, Pointe à Pitre, 97157 Guadeloupe, France

Cyanobacteria have emerged as a target group of interest in the search for new types of bioactive compounds [1]. The benthic species, especially in tropical zones, that may form dense biofilms on various types of substrates are particularly interesting [2] but still poorly known, compared to pelagic species. In this context and in the framework of a French CNRS research project (X-Life CABMAN 2018–2019) aiming to explore benthic cyanobacteria in mangrove ecosystems, a sampling program was conducted in two distinct areas, Guadeloupe (Caribbean) and Mayotte (Indian Ocean) that resulted in isolation of a collection of eighty new cyanobacterial strains. Our first investigations on Guadeloupe strains have combined phylogenetic, chemical and biological studies in order to better understand the taxonomic diversity as well as the chemical ecology of these cyanobacteria through the role of their specialized metabolites. Therefore, in addition to the description of new cyanobacteria species by polyphasic approach, chemical analyses using LC-MS/MS data allowed to set up a molecular network, which was enriched by antimicrobial activities evaluation against human pathogenic and environmental ichtyopathogenic bacterial strains. Results focused on the chemical biodiversity of cyanobacteria isolated from tropical mangrove in French overseas territories will be presented and discussed.

References

1. Shah, S.A.A.; Akhter, N.; Auckloo, B.N.; Khan, I.; Lu, Y.; Wang, K.; Wu, B.; Guo, Y.-W. Structural diversity, biological properties and applications of natural products from Cyanobacteria. A review. *Mar. Drugs*
**2017**, *15*, 354.
2. Alvarenga, D.O.; Rigonato, J.; Branco, L.H.Z.; Fiore, M.F. Cyanobacteria in mangrove ecosystems. Cyanobacteria in mangrove ecosystems. *Biodivers. Conserv.*
**2015**, *24*, 799–817.

#### **Metagenomic Studies of Microbial Sulphur Mats, an Unexplored Natural Product Resource** 

PadhiChandrashekhar^1^RuscheweyhHans-Joachim^1^SuarMrutyunjay^2^SunagawaShinichi^1^PielJörn^1^1Institute of Microbiology, ETH Zurich, 8093 Zurich, Switzerland2School of Biotechnology, KIIT University, Odisha 751024, India

Bacterial natural products are the basis of most known antibiotic families, but have traditionally been isolated from a taxonomically and ecologically limited range of prokaryotic life. Based on the hypothesis that chemical novelty will be found in poorly studied, high-diversity, interaction-rich microbiomes in oligotrophic habitats, we have studied one such community, sulphur mats from a brackish coastal lagoon. These algal mats follow an annual cycle of formation and degradation, and in the later stage of this cycle release the sulphur-based odorous compounds from which they earn their name. Unlike cyanobacterial mats, sulphur mats have not been investigated in context of their natural product potential. In this study, we used metagenomic methods to investigate the biosynthetic potential of the complex microbial mat community. To overcome the challenges of bacterial DNA being overwhelmed and masked by algal and other eukaryotic DNA in the community during metagenome sequencing, we applied an array of binning platforms. The result was high-quality prokaryotic bins that could be assigned to bacterial taxa with relatively high confidence. In-depth analysis of the biosynthetic gene clusters (BGCs) revealed a high abundance of putative ribosomally synthesized and post-translationally modified peptides (RiPPs) and terpenes; a finding that was consistent among spatially distant samples. In contrast, metagenomes from other lake ecosystems have shown a moderate number of polyketide and non-ribosomal peptide BGCs in addition to terpene genes. The high diversity of RiPP systems identified in the mat ecosystem provides a rich discovery resource for bioactive peptides with putative functions in chemical defense.

#### **Toxins from an Unsuspected Invertebrate: A Poisonous Cocktail from a Jawless Predatory Polychaete** 

RodrigoAna P.GrossoAnaBaptistaPedro V.FernandesAlexandra R.CostaPedro M.UCIBIO—Research Unit on Applied Molecular Biosciences, Departamento de Ciências da Vida, Faculdade de Ciências e Tecnologia da Universidade Nova de Lisboa, 2829-516 Caparica, Portugal

Animal venoms are complex mixtures of toxins and other substances with immense biotechnological potential due to their ability to interfere with specific biological pathways. The extraordinary biodiversity of marine invertebrates offers a wide range of novel toxins that are difficult to isolate and characterise in little-known animals with reduced genomic annotation. It is the case for *Eulalia viridis*, a predatory phyllodocid that is believed to secrete noxious substances to assist its predatorial behaviour. Combining RNAseq and microscopy techniques, our work disclosed that *Eulalia* possesses several transcripts that were annotated and found to code for peptides with sequence similarities from previously reported venom components of other species, being secreted in specialised cells located in the proboscis. Specifically, through homology search and phylogenetic analyses, several noxious substances, such as hyaluronidases and cysteine-rich peptides, were found to be closely related with those typically present in venomous animals, such as Hymenoptera, *Conus* and Serpentes, highlighting the diversity and complexity of this cocktail. Among the venomous cocktail of *Eulalia,* proteins with well-preserved domains, such as ankyrin motifs and EGF were also found, common in venom proteins of both invertebrates and vertebrates. These proteins in *Eulalia* venomous secretions can play various functions, such as tissue permeabilization and interference with neuronal calcium channels, as well as having anticoagulant properties. These fit *Eulalia*’s behaviour, which is a jawless worm, feeding by suction after immobilising prey. Altogether, the results indicate that *Eulalia* secretes toxins, rendering it from an apparently inoffensive worm to a fierce predator of the intertidal. 

##### **Acknowledgments** 

The authors acknowledge “Fundação para a Ciência e Tecnologia” for funding project GreenTech (PTDC/MAR-BIO/0113/2014) and the Ph.D. fellowship SFRH/BD/109462/2015 to A.P.R. This work was supported by UCIBIO, financed by national funds from FCT/MCTES (UID/Multi/04378/2019). 

#### **The Chemical Ecology of *Chromodoris* Nudibranchs** 

ChanWeili^1^GarsonMary J.^1^CheneyKaren L.^2^,^3^1School of Chemistry and Molecular Biosciences, University of Queensland, Brisbane 4072, Australia2School of Biological Sciences, University of Queensland, Brisbane 4072, Australia3Queensland Brain Institute, University of Queensland, Brisbane 4072,, Australia

**Figure 15:**
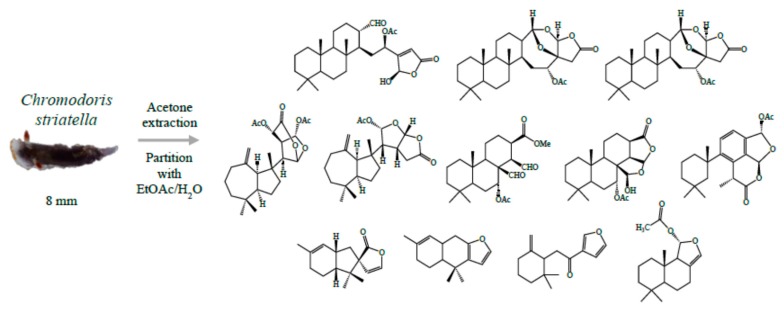
The compounds isolated from *Chromodoris striatella*.

Nudibranchs (Mollusca: Gastropoda: Opisthobranchia) that feed on marine sponges have evolved to acquire and re-purpose noxious sponge metabolites for their own defence. Many chemically protected nudibranchs also display bright colourations and patterns to advertise their unprofitability to predators, a phenomenon known as aposematism (apo: away; sema: sign). In particular, a few aposematic *Chromodoris* species had demonstrated a highly selective sequestration of a toxic macrolide, latrunculin A, in the mantle rim [1]. This contrasts other *Chromodoris* nudibranchs that utilize a complex mixture of compounds in the mantle border for defence. A recent study found that *Chromodoris* nudibranchs display flexible colour patterns. [2] suggesting that this group is involved in a Müllerian mimicry ring whereby conspicuously coloured species mimic one another to increase the efficacy of the aposematic signals to their common predators. Species within a mimicry group may possess different chemical profiles and therefore have unequal levels of chemical defence. Non-chemically protected species can also mimic the colour patterns of a well defended species (Batesian mimicry). This study explores the chemistry of the newly suggested *Chromodoris* mimics, as well as the different chemical strategies employed by chromodorid nudibranchs (Figure 15).

References

1. Cheney, K.L.; White, A.; Mudianta, I.W.; Winters, A.E.; Quezada, M.; Capon, R.J.; Mollo, E.; Garson, M.J. Choose Your Weaponry: Selective Storage of a Single Toxic Compound, Latrunculin A, by Closely Related Nudibranch Molluscs. *PLoS ONE*
**2016**, *11*, e0145134.
2. Layton, K.K.S.; Gosliner, T.M.; Wilson, N.G. Flexible colour patterns obscure identification and mimicry in Indo-Pacific Chromodoris nudibranchs (Gastropoda: Chromodorididae). *Mol. Phylogenet. Evol.*
**2018**, *124*, 27–36.

#### **Bugs from Slugs: Exploring the Bacterial Diversity of Nudibranchs by Metagenomics** 

Pimentel-ElardoSheila^1^CheneyKaren^2^GarsonMary^3^NodwellJustin^1^1Department of Biochemistry, University of Toronto, Toronto, ON M5G 1M1, Canada2School of Biological Sciences, University of Queensland, Brisbane 4072 QLD, Australia3School of Chemistry and Molecular Biosciences, University of Queensland, Brisbane 4072 QLD, Australia

Nudibranchs commonly called sea slugs are marine gastropod mollusks that shed their shells during the larval stage. Due to their lack of physical protection, nudibranchs have evolved different defense mechanisms to deter predators. Several species sequester chemical defenses from their diet and store different secondary metabolites from their prey to render their bodies distasteful to potential predators. In some species, these compounds are selectively localized in different body parts such as the rim as their primary line of defense. Although several defensive secondary metabolites identified in nudibranchs are suspected to be of microbial origin, no one has investigated these so far. 

This study aims to look at the microbiome of different nudibranch species and compare the bacterial composition of different body parts in selected species. We used 16S rRNA gene-based metagenomics to characterize the bacterial composition of 24 different sea slugs mostly nudibranchs, but also including some sacoglossans, sea hares and headshield slugs collected from the Great Barrier Reef, Australia. Analysis of the core microbiome revealed that *Mycoplasma*, *Ruegeria* and *Alteromonas* are the prevalent genera while Proteobacteria as the most abundant phylum. In a few nudibranchs, some bacterial species dominate the entire microbiome (>98%) while in selected species, the rim is dominated by a unique bacterial taxon not found in other parts of the animal. This is the first extensive study looking at the microbiome of diverse nudibranchs. With shotgun metagenomics underway, we hope to correlate the microbial producers of the defensive metabolites found in these remarkable animals.

#### **Potential of Metabolomics in the Integrative Systematics of Octocorals, Case Study in the Tropical Eastern Pacific** 

JaramilloKarla B.^1^,^2^BeniddirMehdi A.^3^AbadRubén^1^RodriguezJenny^1^SanchezJuan A.^4^McCormackGrace^2^ThomasOlivier P.^5^1Escuela Superior Politécnica del Litoral, ESPOL. Centro Nacional de Acuicultura e Investigaciones Marinas, CENAIM. Campus Gustavo Galindo Km. 30.5 Vía Perimetral, P.O. Box 09-01-5863 Guayaquil, Ecuador2Zoology, School of Natural Sciences and Ryan Institute, National University of Ireland Galway, University Road, H91 TK33 Galway, Ireland3Équipe “Pharmacognosie-Chimie des Substances Naturelles” BioCIS, Univ. Paris-Sud, CNRS, Université Paris-Saclay, 5 rue J.-B. Clément, 92290 Châtenay-Malabry, France4Universidad de los Andes, Departamento Ciencias Biológicas, Laboratorio de Biología Molecular Marina (BIOMAR), Cra. 1 #18a 12, Bogotá 111711, Colombia5Marine Biodiscovery, School of Chemistry and Ryan Institute, National University of Ireland Galway, University Road, H91 TK33 Galway, Ireland

Soft corals (Cnidaria, Anthozoa, Octocorallia) represent a benthic group of marine invertebrates that inhabit some ecosystems of the oceans and they are found highly abundant in the Tropical Eastern Pacific. In this marine ecoregion, Ecuador is well-recognized as a marine biodiversity hotspot and has attracted experts in the taxonomy of octocorals. The identification of octocorals in the Eastern Pacific has usually been supported by sclerite characterization and DNA barcoding but despite the valuable contributions of molecular data, many discrepancies remain especially at species level. 

As soft corals are also known to produce a diverse array of secondary metabolites, an integrative approach was conducted to better define the systematics of this group using three methods: morphological characterization of sclerites, phylogenetic analyses (based on two mitochondrial markers COI and MutS markers) and a metabolomic examination (combining LC-MS analyses and MS/MS molecular networking). The untargeted metabolomic approach using UHPLC-HRMS was proven to be useful as a complementary tool in the systematics of these species especially at the genus level. Interestingly, the MS/MS molecular networking revealed key biomarkers for an interspecific discrimination between the 5 different genera studied. Our first insight into the octocorals diversity at El Pelado Marine Protected Area—Ecuador led to the identification of eleven species. These preliminary results demonstrate the high potential of metabolomics for a more general application to the octocoral group.

### ***Theme 2. Isolation and Structure Elucidation of Marine Natural Products*** 

#### **Isolation of Fungi Using the Diffusion Chamber Device FIND** 

LiborBenjaminHarmsHenrikKehrausStefanEgerevaEkaterinaKönigGabriele M.Institute for Pharmaceutical Biology, University of Bonn, Nussallee 6, 53115 Bonn, Germany

**Figure 16:**
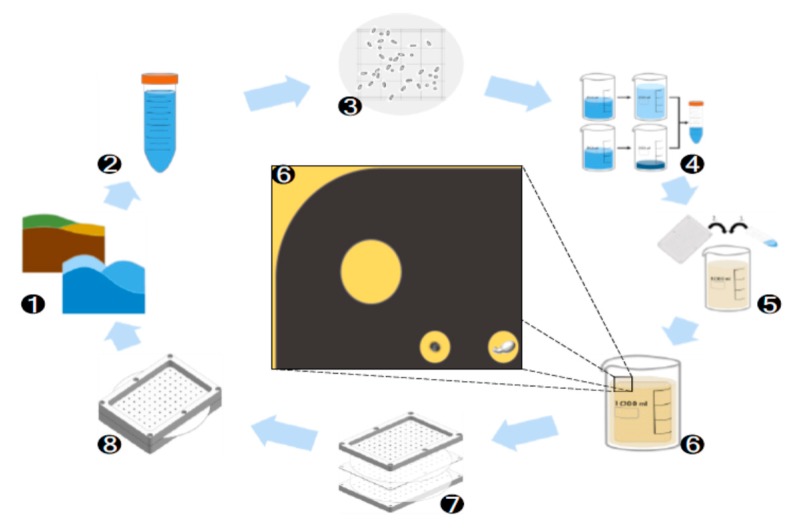
Scheme showing the FIND technology: **1**. Environmental sample, **2**. Sample preparations, **3**. Determination of cell abundance, **4**. Dilution or concentration of sample, **5**–**6**. Addition of sample to molten agar and loading of the central plate to obtain one fungal part in each growing chamber, **7**. Attachment of semipermeable membranes to central plate and sealing by upper and lower plates, **8**. The loaded and assembled device is put back into the original environment for cultivation.

A general problem in trying to obtain axenic cultures of environmental microorganisms is the “great plate count anomaly”, meaning that 99% of all microbes in environmental samples cannot be isolated. In the current study we aimed to improve the isolation of fungal strains with the help of the Fungal one-step IsolatioN Device (FIND) technology. In general, the FIND technology is a multi-chambered micro agar plate, where initially in each chamber only one fungal part is located. After inoculation the device is placed back into the original natural environment of sample collection, to ensure favourable growth conditions. Figure 16 displays the procedure of a FIND experiment. We carried out experiments with terrestrial soil and marine sediment, as well as sea water samples to test this method, and were able to obtain axenic cultures of 12 different filamentous fungal strains, one of them being the marine *Heydenia* cf. *alpina*. The latter yielded two new terpenoid structures, which are the first secondary metabolites from this genus. 

#### **Connecting Molecules to Gene and Back—Can Three Distinct Classes of Swinholide-Like Macrolides Be Produced by the Same Biosynthetic Gene Cluster of a *Symploca* sp.?** 

ReherRaphael^1^Ferreira-LeãoTiago^1^MossNathan A.^1^TekeBahar^1^ArevaloGary^1^NannengaBrent^2^GerwickLena^1^GerwickWilliam H.^1^,^3^1Center for Marine Biotechnology and Biomedicine, Scripps Institution of Oceanography, University of California, La Jolla, CA 92093, USA2Center for Applied Structural Discovery, The Biodesign Institute, Arizona State University, Tempe, AZ 85281, USA3Skaggs School of Pharmacy and Pharmaceutical Sciences, University of California, La Jolla, CA 92093, USA

Tropical marine filamentous cyanobacteria of the genus *Symploca* have been described as prolific producers of bioactive natural products such as the antineoplastic agent dolastatin 10 or the actin cytoskeleton disruptor swinholide A. An environmental collection of the American Samoan cyanobacterium *Symploca* sp. ASS-20JUL14-1 was fractionated guided by both MS^2^-based molecular networking and cytotoxicity against NCI-H460 human lung cancer cells and led to the detection of swinholide A-J as well as samholide A-I. The planar structures of the latter, occuring in nanomolar quantities, were proposed by manual MS^2^ analysis and remain to be confirmed by the cryoEM method MicroED, a novel cutting-edge technique for the structure elucidation of small molecules with drastically reduced compound and analysis time requirements. 

Further, we isolated and elucidated the structure of a new swinholide-like macrolide, symplocolide A (**1**), a structural hybrid of swinholide A and luminaolide B. This raised interest in the biosynthesis of **1**; after genome sequencing and assembly using the MiBIG reference biosynthetic gene cluster (BGC) “*swi*” and initial mining of the biosynthetic pathways, we located a 98 kB BGC, “*sym*” that putatively encodes for **1**. Preliminary biosynthetic analysis suggests that *sym* might not only be responsible for the production of **1**, but intriguingly, for the aforementioned swinholides and samholides as well. BiGSCAPE analysis of the *sym* BGC versus all cyanobacterial genomes from NCBI and the Gerwick lab, as well as use of the bioinformatic tool transATor, support this hypothesis, paving the way for decrypting the mechanism of biosynthesis of these divergent swinholide-like metabolites. 

**Figure:**
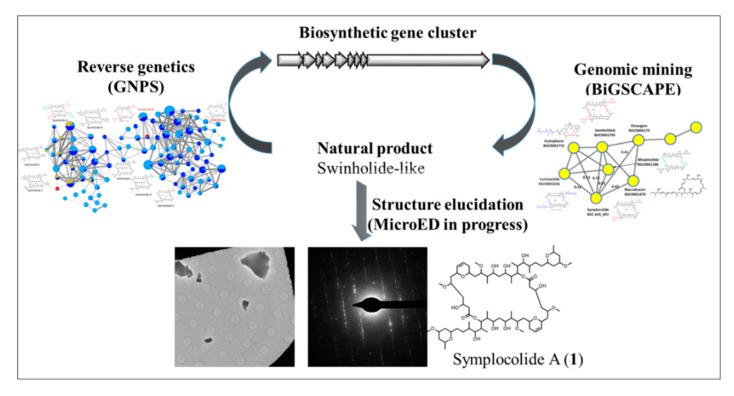
**Graphical Abstract**

#### **Bisindole Alkaloids Isolated from the Irish Coastal Sponge, *Spongosorites calcicola*, as Part of the National Marine Biodiscovery Project, NMBLI** 

JenningsLaurence K.^1^,^2^KaurNavdeep^1^,^2^RodriguesDaniel^1^,^2^FisherJeffrey^2^ThomasOlivier P.^1^1Marine Biodiscovery, School of Chemistry and Ryan Institute, National University of Ireland Galway (NUI Galway), University Road, H91 TK33 Co. Galway, Ireland2Marine Biodiscovery, Marine Institute, Rinville West, H91 R673 Co. Galway, Ireland

Over the last 60 years, marine natural products have been reported with a unique chemical diversity not observed in terrestrial natural products. However, there still remains significant areas of largely unexplored diverse marine habitats. The Beaufort Marine Biodiscovery project established the basis for marine biodiscovery in Ireland. Currently, the national marine biodiscovery laboratory in Ireland project (NMBLI) is continuing this marine natural products discovery campaign, with a primary goal to examine Irish coastal invertebrates to identify bioactive marine natural products. 

**Figure 17:**
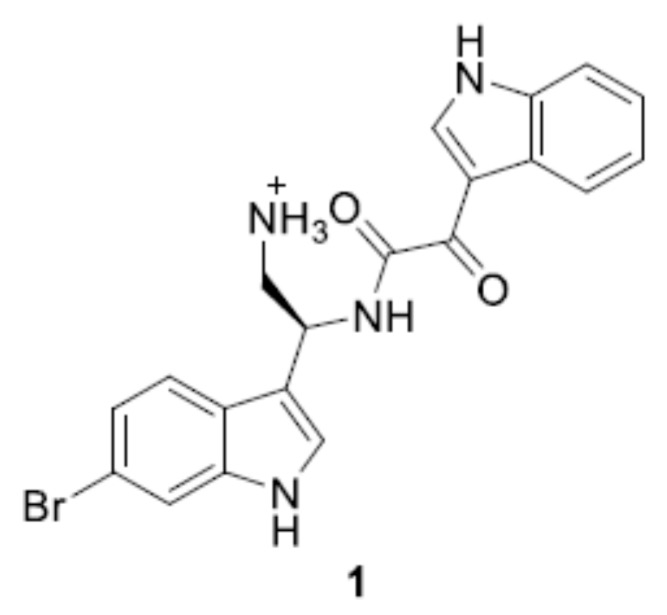
Structure of Calcicamide isolated from *S. calcicola.*

Following initial chemical and biological screening of Irish marine invertebrates, fractions of the sponge *Spongosorites calcicola* were found to exhibit strong anti-tumor activities and a large number of brominated metabolites. Herein, we describe the isolation, structure elucidation and novel biological evaluation of one new bisindole alkaloid, Calcicamide (**1**), from *S. calcicola* (Figure 17). Calcicamide is biosynthetically related to a number of other common marine natural products including the topsentins, spongotines and hamacanthins. We will also present the effects of Calcicamide as well as five other known bisindole analogues on the expression of the telomeric protein TRF2, which exhibits potent pro-oncogenic properties. 

#### **The Structure and Biological Activity of Heinamides from the Cyanobacterium *Nostoc* sp. UHCC0702** 

HeiniläL. M. P.JortikkaA.JokelaJ.WahlstenM.FewerD. P.SivonenK.Department of Microbiology, University of Helsinki, Helsinki 00014, Finland

**Figure 18:**
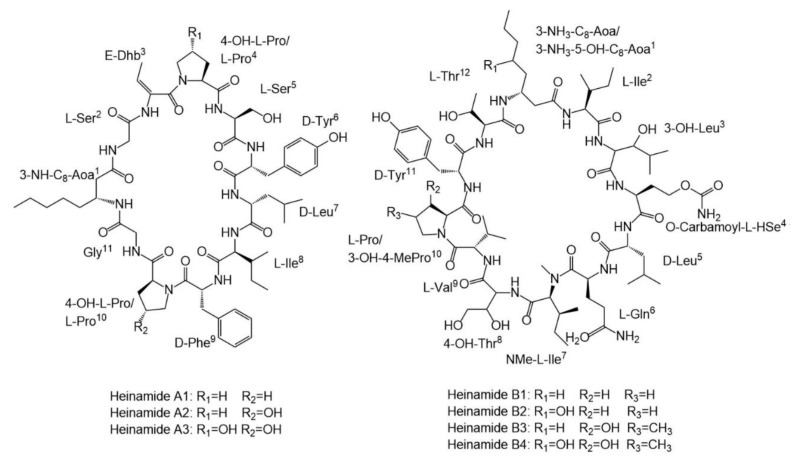
Heinamide structures.

Cyanobacteria are a rich source of natural products many of which have potent antifungal activity. Here we report the discovery of heinamides from the filamentous diazotrophic cyanobacterium *Nostoc* sp. UHCC 0702. Heinamides are new macrocyclic peptides belonging to two separate structural classes that both exhibitted antifungal activity against *Aspergillus flavus*. The chemical structures of the compounds were elucidated with UPLC-MS and NMR. The isolated compounds were named heinamides A1–A3 and B1–B4 (Figure 18) and exhibited structural similarity to the laxaphycin A (11 residue) and laxaphycin B (12 residue) families of macrocyclic peptides. The two classes of peptides exhibited synergistic antifungal effect when one compound from both groups was present. The antifungal acitivity was minimal when tested with individual compounds and with multiple compounds of the same size group. We obtained a genome sequence for *Nostoc* sp. UHCC 0702 and identified the biosynthetic genes for heinamide production. This study demonstrates that the production of antifungal laxaphycin A and B peptide pairs that act in synergy is widespread in cyanobacteria. 

#### **Isotopologue-Guided Identification of Halogenated Anilines from a Benthic Diatom** 

BaumeisterTim U. H.^1^StaudingerMona^2^PohnertGeorg^1^,^2^1Max Planck Institute for Chemical Ecology, Fellow Group Plankton Community Interaction, Hans-Knöll-Straße 8, 07745 Jena, Germany2Friedrich Schiller University Jena, Institute for Inorganic and Analytical Chemistry, Department of Bioorganic Analytics, Lessingstr. 8, 07743 Jena, Germany

The intriguing isotope pattern produced by brominated/chlorinated compounds is a useful property to identify those compounds in an extract by chromatography-coupled mass spectrometry. The introduction of high resolution accurate mass (HRAM) mass spectrometry further allowed for using the mass difference between light and heavier isotopes as a property for identification. In an integrated approach, DeltaMS, an open source R app, uses the mass difference and the isotope pattern to highlight potential candidate ions. As a proof of concept, extracts from the marine benthic diatom *Nitzschia* cf. *pellucida*, known for its possession of haloperoxidases, were obtained and analyzed on a HRAM GC-Orbitrap mass spectrometer. Several brominated compounds could be detected, whereby the detection of a tribrominated compound drew our attention. Structure elucidation by MS and a co-injection confirmed the presence of 2,4,6-tribromoaniline which was so far only known as an anthropogenic pollutant. But not only tribromoaniline, but also the trichloroaniline, and the mixed trihalogenated (Cl/Br) anilines could be identified. Stable isotope labeling, using Na^15^NO_3_, and NaH^13^CO_3_ as sole carbon and nitrogen source in the growth medium, confirmed the biosynthesis of the halogenated anilines by the diatom. Intra- and extracellular monitoring of the halogenated anilines at different time points during the algal growth showed an accumulation of those compounds. Intracellular amounts exceeded the extracellular by many times, indicating that those compounds could act as a deterrent against grazers. This is the first study that shows the presence of biosynthesized trihalogenated anilines.

#### **Spinochromes of Pacific Sea Urchins: Distribution and Bioactivity** 

VasilevaElena A.MishchenkoNatalia P.FedoreyevSergey A.G.B. Elyakov Pacific Institute of Bioorganic Chemistry, Far-Eastern Branch of the Russian Academy of Sciences, 690022 Vladivostok, Russia

Marine hydrobionts, such as sea urchins, and specifically their gonads, are a valuable renewable food resource. At the same time, they can serve as a unique source of various natural compounds, which can be the basis for the creation of various biomaterials, effective medicinal and parapharmaceutical preparations, as well as functional food products. After the removal of the gonads, large amounts of sea urchin shells are left as waste. This shell material is rich in bioactive quinonoid pigments, principally spinochromes. Although this class of compounds has been known for over 100 years, there is still not so much data on their chemical and biological properties. The most common spinochrome—echinochrome A is the active substance in the Russian antioxidant drug Histochrome^®^ that is being used successfully in cardiology and ophthalmology. Recent findings revealed new properties of echinochrome A—anti-diabetic, anti-allergic, gastroprotective, mitochondria-protective and other activities. Seeing echinochrome A to exhibit so many different effects it is interesting to study the distribution and biological activity of other spinochromes. 

Using validated HPLC-DAD-MS method we investigated spinochrome composition of 21 Pacific sea urchin species. Ten spinochromes containing different substituents in 1,4-naphthoquinone core were isolated for the structure-activity relationship studies. All these compounds were tested for in vitro antioxidant activity using several standard assays, in vitro cardioprotective activity on the model of doxorubicin-induced oxidative stress in human cardiomyocytes, and for their ability to prevent cisplatin-induced oxidative stress in mice kidneys. The most potent antioxidants appeared to be echinamine B, spinochromes D and E and 7,7’-anhydroethylidene-6,6’-bis(2,3,7-trihydroxynaphthazarin). 

#### **Marine Sponges from Indian Ocean: A Highly Promising Source for the Discovery of Novel Bioactive Compounds to Fight against Ageing and Age-Related Diseases** 

HassaneCharifat Saïd^1^CamposPierre-Eric^1^TintillierFlorent^1^ClercPatricia^1^BoyerJean-Bernard^1^VoogdNicole De^2^FokialakisNikolas^3^TrougakosIoannis P.^4^GardikisKonstantinos^5^WenzkowskiChristine^6^BignonJérôme^7^Le GoffGéraldine^7^MoriouCéline^7^Al-MourabitAli^7^DufosseLaurent^1^FouillaudMireille^1^OuazzaniJamal^6^BialeckiAnne^1^1LCSNSA, Université de la Réunion, Ile de la Réunion, 97744 Saint-Denis, France2Naturalis Biodiversity Center, Leiden, Darwinweg 2, 2333 CR Leiden, The Netherlands3Faculty of Pharmacy, National & Kapodistrian University of Athens, 15771 Athens, Greece4Faculty of Biology, National & Kapodistrian University of Athens, 15784 Athens, Greece5APIVITA SA, Research and Development Department, Industrial Park of Markopoulo, Markopoulo Mesogaias, 19003 Athens, Greece6Crelux GmbH, 82152 Martinsried, Germany7ICSN-CNRS, 91190 Gif sur Yvette, France

Ageing is commonly defined as the accumulation of diverse deleterious changes occurring in cells and tissues with advancing age that are responsible for the increased risk of pathologies such as Alzheimer’s disease, cardiovascular diseases, neurodegeneration or cancers. As the population of developed countries is ageing, the prevalence of a variety of age-related diseases is increasing. In order to counteract this major healthcare challenge, marine natural products represent an extraordinary reservoir of structurally diverse bioactive metabolites which may offer anti-ageing properties with pharmaceutical, cosmeceutical and nutraceutical applications. 

Taking into consideration the aforementioned issues, the H2020 European project TASCMAR explores marine invertebrates and symbionts from under-investigated marine biodiversity hotspots and develops innovative approaches for the discovery and production of compounds with anti-ageing activity. The Chemistry Laboratory of Natural Substances and Food Sciences (LCSNSA, University of La Reunion) located at Reunion island is involved in this ambitious research program and this communication will therefore provide an outline of the contribution made by the LCSNSA to TASCMAR. The laboratory has collected a total of 112 samples of sponges from Mayotte and Rodrigues (Indian Ocean). The samples were extracted and the crude extracts obtained were submitted to a biological evaluation against a wide range of different targets involved in ageing or age-related diseases. These targets include catalase, sirtuin 1, CDK7, proteasome, Fyn kinase, tyrosinase and elastase. Twenty-nine (29) crude extracts have shown promising results. The chemical investigation of these 29 extracts for the discovery of molecules with anti-ageing effects will be discussed. 

#### **MS Dereplication for Rapid Discovery of Structurally New or Novel Natural Products** 

TabudravuJioji N.^1^,^2^PellissierLéonie^2^SmithAlan James^2^KidRichard^3^MiltonEdward J.^4^DengHai^2^EbelRainer^2^GissiCarmela^5^,^6^MilneBruce F.^7^CimpanGabriela^4^JasparsMarcel^2^1School of Forensic and Applied Sciences, Faculty of Science & Technology, University of Central Lancashire, Preston, Lancashire PR1 2HE, UK2Marine Biodiscovery Centre, Department of Chemistry, University of Aberdeen, Scotland AB24 3UE, UK3Publisher, Data & Databases, Royal Society of Chemistry, Thomas Graham House, Science, Park, Milton Road, Cambridge CB4 0WF, UK4Advanced Chemistry Development, UK Ltd. Venture House, Arlington Square, Downshire Way, Bracknell, Berks RG12 1WA, UK5Department of Biosciences, Biotechnologies and Biopharmaceutics, University of Bari “A. Moro”, Via Orabona 4, 70125 Bari, Italy6IBIOM, Istituto di Biomembrane, Bioenergetica e Biotecnologie Molecolari, CNR, Via Amendola 165/A, 70126 Bari, Italy7CFisUC, Department of Physics, University of Coimbra, Rua Larga, 3004-516 Coimbra, Portugal

In order to accelerate the isolation and characterisation of structurally new or novel natural products, it is crucial to develop efficient strategies that prioritise samples with greatest promise early in the workflow so that resources can be utilised in a more efficient and cost-effective manner. Two complementary approaches have been developed: One is based on targeted identification of known compounds held in a database based on high resolution MS and predicted LC retention time data [1]. The second is an MS metrics-based approach where the software algorithm calculates metrics for sample novelty, complexity, and diversity after interrogating databases of known compounds, and contaminants. These metrics are then used to prioritise samples for isolation and structure elucidation work [2]. Both dereplication approaches have been validated using natural product extracts resulting in the isolation and characterization of new or novel natural products. 

References

1. Chervin, J.; Stierhof, M.; Tong, M.H.; Peace, D.; Hansen, K.Ø.; Urgast, D.S.; Andersen, J.H.; Yu, Y.; Ebel, R.; Kyeremeh, K.; et al. Targeted Dereplication of Microbial Natural Products by High-Resolution MS and Predicted LC Retention Time. *J. Nat. Prod.*
**2017**, *80*, 1370–1377, doi:10.1021/acs.jnatprod.6b01035.
2. Tabudravu, J.N.; Pellissier, L.; Smith, A.J.; Subko, K.; Autréau, C.; Feussner, K.; Hardy, D.; Butler, D.; Kidd, R.; Milton, E.J.; et al. LC-HRMS-Database Screening Metrics for Rapid Prioritization of Samples to Accelerate the Discovery of Structurally New Natural Products. *J. Nat. Prod.*
**2019**, *82*, 211–220, doi:10.1021/acs.jnatprod.8b00575.

#### **Cone Snails Natural Products: Isolation and Characterization of Toxins** 

NevesJorge L. B.^1^,^2^ImperialJulita S.^3^LinZhenjian^3^MorgensternDavid^4^UeberheideBeatrix^4^GajewiakJoanna^3^RobinsonSamuel D.^3^EspinoSamuel^3^WatkinsMaren^3^AntunesAgostinho^1^,^5^SchmidtEric W.^3^VasconcelosVitor^1^,^5^OliveraBaldomero M.^3^1CIIMAR/CIMAR, University of Porto, Terminal de Cruzeiros do Porto de Leixões, Avenida General Norton de Matos, S/N, 4450-208 Matosinhos, Portugal2Faculty of Engineering and Marine Science, University of Cabo Verde, Mindelo CP 163, Cabo Verde3Departments of Medicinal Chemistry and Biology, University of Utah, Salt Lake City, UT 84112, USA4Langone Medical Center, Department of Biochemistry and Molecular Pharmacology, New York University, New York, NY 10016, USA5Faculty of Sciences, University of Porto, Rua do Campo Alegre, 4169-007 Porto, Portugal

The natural products from Cone Snails (*Conus* spp.) venoms have received much attention over the last decades due to the biological activity, extraordinary diversity, and molecular studies that opened a window for biomedicine research [1]. Venomous *Conus* are highly specialized venomous predators that may produce up to 100,000 small compounds and it is estimated over 2,000,000 natural products to be present in the venoms of venomous marine snails. Conotoxins, are currently being developed as analgesics for the treatment of neuropathic pain. In December 2004, the synthetic version of the peptide ω-conotoxin MVIIA (commercial name Prialt^®^) from *C. magus* has been approved by the US-FDA to treat chronic pain in humans [2,3]. 

Our research has been focused on Cabo Verde venomous marine snails particularly those that belong to the genus *Conus*. This project includes the isolation and structure elucidation of compounds from the venom of two species, one endemic—*Conus ateralbus*, and one non-endemic *Conus genuanus*; using MALDI-TOF, LC-MSMS, and NMR data. We described the isolation and characterization of the first bioactive peptide from the venom of *C. ateralbus*. The 30Amino Acid (AA) venom peptide is named δ-conotoxin AtVIA. An excitatory activity was manifested by the peptide on a majority of mouse lumbar dorsal root ganglion neurons and homology which include conserved sequence elements with δ-conotoxins (pharmacology family) from another worm and fish-hunters [4]. On the *C. genuanus* we did the chemical characterization of a novel small molecule, a guanine derivative with unprecedented features; we named it genuanine. Genuanine was neuroactive when injected into mice having paralytic activity [5].

References

1. Terlau, H.; Olivera, B.M. Conus venoms: a rich source of novel ion channel-targeted peptides. *Physiol. Rev.*
**2004**, *84*, 41–68.
2. Jimenez, E.C.; Shetty, R.P.; Lirazan, M.; Rivier, J.; Walker, C.; Abogadie, F.C.; Yoshikami, D.; Cruz, L.J.; Olivera, B.M. Novel excitatory Conus peptides define a new conotoxin superfamily. *Nurochem*
**2003**, *85*, 610.
3. Olivera, B.M.; Russell, W. *Molecular Interventions*; University of Utah: Salt Lake City, UT, USA, 2007; pp. 251–260.
4. Neves, J.L.B.; Lin, Z.; Imperial, J.S.; Antunes, A.; Vasconcelos, V.; Olivera, B.M.; Schmidt, E.W. Small Molecules in the Cone Snail Arsenal. *Org. Lett.*
**2015**, *17*, 4933–4935.
5. Neves, J.L.B.; Imperial, J.S.; Morgenstern, D.; Ueberheide, B.; Gajewiak, J.; Antunes, A.; Robinson, S.D.; Espino, S.; Watkins, M.; Vasconcelos, V.; et al. Characterization of the First Conotoxin from *Conus ateralbus*, a Vermivorous Cone Snail from the Cabo Verde Archipelago. *Mar. Drugs*
**2019**, *17*, 432.

#### **The Prospects of Microbial Natural Products as a Source of Possible Drug Prototypes for the Neglected African Disease: Ghana, a Case Study** 

KyeremehKwaku^1^,^2^DengHai^2^JasparsMarcel^2^1Marine and Plant Research Laboratory of Ghana, Department of Chemistry, School of Physical and Mathematical Sciences, University of Ghana, P.O. Box LG 56 Legon-Accra, Ghana2Marine Biodiscovery Centre, Department of Chemistry, University of Aberdeen, Old Aberdeen AB24 3UE, Scotland, UK

The sub-Saharan Africa (SSA) region is burdened with a high incidence of infections, schistosomiasis, trypanosomiasis, leishmaniasis and cancer. There is current rapid widespread development of resistance to the available treatments for these diseases. More striking a fact is that; for the parasitic diseases, the available number of drugs for treatment is exceptionally low and each of these have been under prescription for periods not less than 30 years. In fact, an average of the prescription periods from time of discovery to-date for the top 10 antiparasitics (Praziquantel or oxamniquine, amphotericine, pentavalent antimonials, paromomycin, miltefosine, pentamidine, fluconazole/itraconazole, melarsoprol, eflornithine and nifurtimox) that is likely to be prescribed to you in the clinic today is 56 years. Interestingly, there is a rising expertise of drug research scientists in SSA who have received training from Europe and other parts of the world and this coupled with the huge natural product diversity of the region necessitates the discovery of new drug scaffolds. Microbial natural products provide the largest chemical and biological diversity in any drug discovery screening program compared to other natural sources like plants and invertebrates with the resupply problems mostly overcome with large scale fermentation and heterologous expression. In the last six years, many interesting molecules such as butremycin, butrepyrazinone, butrecitrinadin, paenidigyamycins, legonmycins, legonindolizidines, legonmaleimides and legonaridines and many interesting enzymes like the Talented Solo Legon C have been discovered from novel Ghanaian microbes. This presentation outlines what is indeed the first major attempt to discover new drug scaffolds from SSA microbes. 

#### **“Being *Penicillium ubiquetum*”: Metabolomics and Molecular Networking for the Discovery of Rare New Natural Products from a Marine-Sourced Fungus** 

HoangThi Phuong Thuy^1^,^2^RoullierCatherine^1^,^3^GallardJean-François^4^PouchusYves François^1^BeniddirMehdi A.^5^GrovelOlivier^1^,^3^1Nantes Université, Faculty of Pharmacy, MMS, 9 Rue Bias, 44035 Nantes, France2Phu Tho College of Pharmacy, Phu Tho 290000, Vietnam3Corsaire-ThalassOMICS Metabolomics Facility, Biogenouest, Université de Nantes, 44035 Nantes, France4Institut de Chimie des Substances Naturelles, CNRS UPR 2301, University of Paris-Saclay, 91198 Gif-sur-Yvette, France5BioCIS, Université Paris-Sud, CNRS, Université Paris-Saclay, 92290 Châtenay-Malabry, France

Marine microorganisms, including fungi, are the source of ever more original molecules that can be used therapeutically. Nevertheless, their discovery requires the use of elicitation strategies of cryptic biosynthetic pathways to reveal their metabolic potential. These methods, such as co-cultivation, OSMAC or epigenetic remodeling, sometimes lead to the production of new major metabolites. In a much more frequent way they induce an over-expression of many original molecules produced in very small quantities, therefore very difficult to detect. For this, metabolomic and dereplicative tools such as molecular networks allow an exhaustive investigation of metabolic capacities and their variations.

In this way, in the goal of the discovery of new marine fungi natural products, we have investigated various marine-sourced *Penicillium* sp. strains including a *P. ubiquetum*, a species for which no chemical works have been reported so far. The study of variations of its metabolic expression in response to the cultural conditions has been carried out by LC-HRMS/MS. By combining metabolomics analyses, dereplication and molecular networking, we highlighted the ability of *P. ubiquetum* to direct its metabolism—under specific conditions and in the presence of seawater—toward the production of an abundant series of minor complex meroterpenoids and some unusual C25-steroids. MS-guided purification led to the discovery of new isobaric analogs in these two rare chemical families, showing the power of new bioinformatics tools for natural products discovery.

#### **Egyptian Fungal Antibiotic Metabolites—From Pharaohs to Modern Textiles** 

SoldatouSylvia^1^QaderM. Mallique^2^PavesiCoralie^1^MirandaKevin Jace^1^DiyaolouOluwatofunmilay A.^1^HamedAhmed A.^3^RatebMostafa E.^2^EbelRainer^1^1Marine Biodiscovery Centre, Department of Chemistry, University of Aberdeen, Aberdeen AB24 3UE, Scotland, UK2School of Computing, Engineering & Physical Sciences, University of the West of Scotland, Paisley PA1 2BE, UK3Microbial Chemistry Department, National Research Center, 33 El-Buhouth Street, P.O. Box 12622, Dokki, Giza 12622, Egypt

Since the discovery of penicillin, fungi have been in the spotlight as a prolific source of bioactive agents with many examples in the literature emphasising their importance in drug discovery. In our efforts towards the discovery of new bioactive fungal metabolites from niche ecosystems, several fungal strains were isolated from marine organisms and plants collected from Hurghada (Red Sea) and Wadi El Natrun valley, respectively. Based on the antimicrobial screening against a panel of pathogenic microorganisms, fifteen endophytic or invertebrate-associated fungal isolates were prioritised for further analysis. Chemical investigation was carried out for organic extracts obtained from small-scale fermentations. Specifically, analysis of the MS^2^ data through the GNPS platform revealed several known compounds which clustered with unidentified parent ions, suggesting the presence of new secondary metabolites. Fungal fermentation on rice afforded sufficient biomass for fractionation and separation which led to the isolation of a suite of new and known compounds belonging to various classes. In particular, itaconic acid and kojic acid derivatives were isolated from a *Cladosporium* sp., whereas a new cyclo-peptide and a new morpholine-2,5-dione were isolated from an *Epicoccum* sp. and *Alternaria* sp., respectively. Moreover, potentially new analogues of emericellamide A were identified in the molecular network of the crude extract of a marine *Aspergillus* sp. An addtional *Aspergillus* strain produced a suite of butyrolactone derivatives. The pure metabolites were screened for antibiotic and cytotoxic activity. The ultimate goal of this interdisciplinary project is to incorporate the new antimicrobial compounds onto textile substrates to enhance their functionality**.**

#### **Targeted Co-Cultivation of Baltic Marine fungi with Phytopathogens for Discovery of Novel Natural Agrochemicals** 

Oppong-DanquahErnestBlümelMartinaTasdemirDenizGEOMAR Centre for Marine Biotechnology, Research Unit Marine Natural Product Chemistry, GEOMAR Helmholtz Centre for Ocean Research Kiel, Am Kiel-Kanal 44, 24106 Kiel, Germany

**Figure 19:**
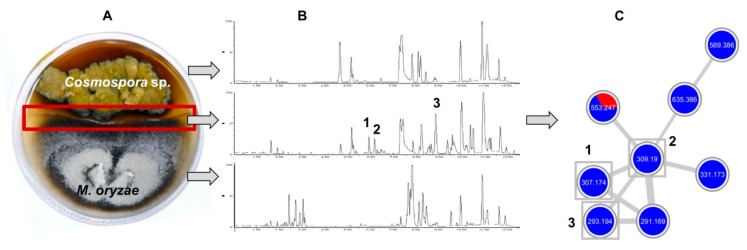
(**A**) Co-culture of *Cosmospora* sp. (top) and *M. oryzae* (bottom) including a marked inhibition zone in the middle (framed in red). (**B**) UPLC-MS spectra of the respective cultivation types (*Cosmospora* sp. top; *M. oryzae* bottom; inhibition zone of co-culture middle with three induced peak ions (**1**–**3**). (**C**) Molecular network showing a cluster containing the novel compounds (**1**–**3**) in the same molecular family.

Resistance of phytopathogens to pesticides threatens global food security, necessitating discovery of novel, ecofriendly crop-protection agents. Fungi have been prolific sources of natural agrochemicals. However, genomic studies point out a discrepancy between the often high number of biosynthetic gene clusters (BGCs) and low number of molecules obtained from fungi, because many BGCs remain silent in monoculture conditions. Co-cultivation of two microorganisms is a highly efficient method to awaken cryptic BGCs and enhance chemical diversity. Herein we adopted a unique co-cultivation approach involving marine fungi and plant pathogens to induce the targeted production of natural antibiotics against phytopathogens. Towards this aim, 123 marine-adapted fungi from the Baltic Sea were isolated and identified. 21 selected isolates were co-cultivated with economically relevant bacterial (*Pseudomonas syringae*, *Ralstonia solanacearum*) and fungal (*Magnaporthe oryzae*, *Botrytis cinerea*) phytopathogens. UPLC-QToF-MS/MS-based untargeted metabolomics approach using molecular networking (GNPS) was performed for comparative metabolome analyses of mono- and co-cultures. Co-cultivation of the marine fungus *Cosmospora* sp. and the phytopathogen *M. oryzae* led to production of 3 novel coumarans in the inhibition zone (Figure 19) that were absent in the monocultures. These compounds, plus several known naphtho-γ-pyrones and isochromans were purified by HPLC and characterized by spectroscopic means. The new and known compounds showed antifungal activities (up to IC_50_ 0.8 μg/mL against *M. oryzae*). These results render phytopathogens useful elicitors for inducing novel chemistry in fungal co-culture studies for discovery of crop protection agents. 

#### **Dereplication of Natural Products Using Diffusion Ordered Spectroscopy (DOSY)** 

KleksGuy^1^,^2^AveryVicky M.^2^CarrollAnthony R.^1^,^2^1Environmental Futures Research Institute, Griffith University, Gold Coast QLD 4222, Australia2Griffith Institute for Drug Discovery, Griffith University, Brisbane QLD 4111, Australia

**Figure 20:**
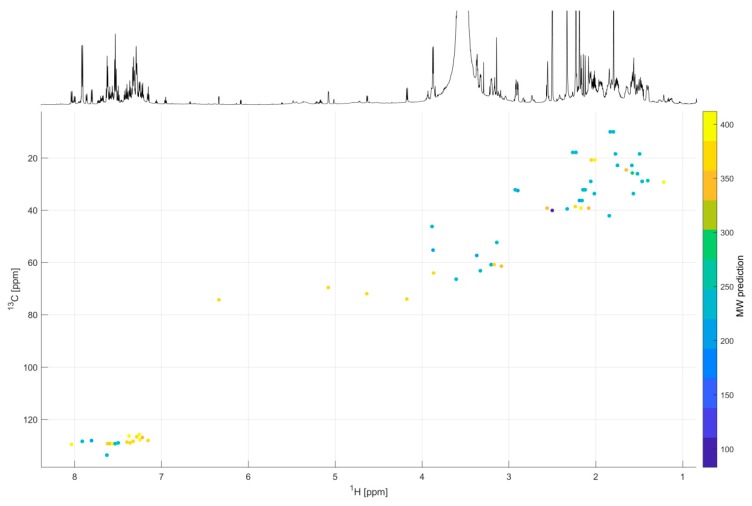
DOSY-HSQC of a crude extract with molecular weight prediction representation as a colour scale.

One of the challenges in natural product discovery is the re-isolation of known compounds. Dereplication is the process used to quickly identify these known compounds, thus saving time and effort directed at their re-isolation and structure determination. MS and/or NMR methods have been used in dereplication and each technique generates its own unique set of data (either mass or functional group based). Diffusion-ordered spectroscopy (DOSY) is a powerful tool that allows for the spectroscopic separation of components of a mixture by their diffusion coefficient. This separation is determined by the size and shape of the molecule in solution and thus provides an opporuntiy to generate molecular weight data directly from NMR spectra. We have generated DOSY diffusion coefficents for a library of known natural products and developed a correlation matrix to predict molecular weights by NMR (Figure 20). This approach have been successfully applied to identify known natural products in complex mixtures and to predict the molecular weight of unknown compounds in a mixture. The DOSY approach therefore provides a useful dereplication tool that bridges the gap between hyphenated MS/NMR dereplication methods. The biggest disadvatange of 2D DOSY is signal overlap, which leads to inaccurate estimation of diffusion coefficients resulting in large errors in the molecular weight prediction. Since signal overlap is unavoidable for a complex mixture such as an extract, we also utilzed 3D DOSY experiments. Here we demonstrate the dereplication of natural products using 2D and 3D DOSY. 

#### **Halogenated Tyrosine Derivatives from the Pacific Zoantharian *Antipathozoanthus hickmani*** 

GuillenPaul O.^1^,^2^JaramilloKarla B.^1^,^3^JenningsLaurence^2^Genta-JouveGrégory^4^,^5^de la CruzMercedes^6^CautainBastien^6^ReyesFernando^6^RodríguezJenny^1^ThomasOlivier P.^2^1ESPOL Escuela Superior Politécnica del Litoral, ESPOL, Centro Nacional de Acuacultura e Investigaciones Marinas, Campus Gustavo Galindo km. 30.5 vía Perimetral, P.O. Box 09-01-5863 Guayaquil, Ecuador2Marine Biodiscovery, School of Chemistry and Ryan Institute, National University of Ireland Galway (NUI Galway), University Road, H91 TK33 Galway, Ireland3Zoology, School of Natural Sciences and Ryan Institute, National University of Ireland Galway (NUI Galway), University Road, H91 TK33 Galway, Ireland4Équipe C-TAC, UMR CNRS 8038 CiTCoM—Université Paris Descartes, 4 Avenue de l’Observatoire, 75006 Paris, France5Unité Molécules de Communication et Adaptation des Micro-Organismes (UMR 7245), Sorbonne Universités, Muséum National d’Histoire Naturelle, CNRS, 75231 Paris, France6Fundación MEDINA, Centro de Excelencia en Investigación de Medicamentos Innovadores en Andalucía, Avda. del Conocimiento 34, Parque Tecnológico de Ciencias de la Salud, E-18016 Armilla, Granada, Spain

Zoantharians (Cnidaria: Hexacorallia) are sessile invertebrates commonly found in all marine ecosystems. Despite their wide distribution, chemical investigation on these organisms has been generally overlooked and they are limited to species belonging to the genera *Zoanthus* and *Palythoa.* Most reports of their chemical diversity deal with species collected from the Indo-Pacific and Atlantic Ocean, while species from the Tropical Eastern Pacific have been poorly investigated. *Antipathozoanthus hickmani* is one of the most representative zoantharians from this ecosystem, and it was first reported from the Galapagos Islands where it overgrows the black coral *Anthipathes galapagensis*. 

Herein, we report the isolation and structure elucidation of four halogenated dipeptides named Valdiviamides A-D isolated from *A. hickmani* collected in the Marine Protected Area El Pelado, Santa Elena-Ecuador. Valdiviamides A-D are halogenated tyrosine dipeptides characterized by the presence of bromine and iodine atoms on the phenol ring. Additionally, we propose halogenated tyrosine derivatives as chemical markers for species of the family Parazoanthidae. 

#### **Novel Zwitterionic Metabolites from Marine Diatoms** 

FeniziaSimona^1^,^2^PohnertGeorg^1^,^2^1Institute for Inorganic and Analytical Chemistry, Friedrich Schiller University, Lessingstrasse 8, 07743 Jena, Germany2Max Planck Institute for Chemical Ecology, Hans-Knöll-Straße 8, 07745 Jena, Germany

Zwitterions are characterized by the presence of both a positive and a negative charge within one molecule. They play an important role in the environment, since they are involved in the modulation of the global sulphur cycle in the atmosphere and since they mediated many interactions among marine species. 

The identification and classification of zwitterionic metabolites has been problematic until our development of novel chromatographic and mass spectrometric methods. These analytical methods show the presence of many zwitterions in microalgae that have not been recognized or characterized previously. In this talk, I will present how liquid chromatography coupled with mass spectrometry is utilized to assign novel hitherto unknown components in the “zwittermetabolome” of diatoms. The physiological functions of novel key metabolites is introduced and ecological implications are discussed. I report studies on three diatom species, *Phaeodactylum tricornutum*, *Skeletonema costatum* and *Thalassiosira weissflogii* that have emerged as model organisms in phycological studies. 

#### **Quimioprospecting for *Streptomyces* from the South Pacific: Genomic and Metabolic Study of Novel Compounds** 

SernaN.ZamoranoN.CámaraB.Laboratorio de Microbiología Molecular y Biotecnología Ambiental, Departamento de Química & Centro de Biotecnología DAL, Universidad Técnica Federico Santa María, Avenida España 1680, Casilla 110-V, Valparaíso, Chile

The indiscriminate use of antibiotics has enhanced the favourable conditions for the selected microorganisms. The scientific community is focusing on the search for new antibiotic compounds, which implies the need to identify new bioactive molecules. Specifically, marine actinomycetes have aroused interest because they are emerging sources of antibiotic compounds. 

Our group has been working on the bioprospecting of Chilean marine actinomycetes, in order to investigate their biotechnological potential to produce active secondary metabolites against pathogenic strains model, in this exploration we obtained a collection of 30 genera of actinomycetes and a possible novel genera belonging to the *Nocardiopsaceae* family. 

Antimicrobial activity assay of extracts crude of our actinobacteria obtained for OSMAC strategy was tested against pathogenic strain models like *Staphylococcus aureus*, *Pseudomonas aeruginosa*, and *Saprolegnia parasitica*. The promissory active extracts were selected to determine chemical profile using liquid chromatography high resolution mass spectrometry (LC-HRMS) that allowed dereplicated secondary metabolite and builds a hierarchical cluster, which enabled to choose those strains with new chemical entities and where further purification and identification efforts are being achieved. In addition, the fermentation extracts analyzed showed that 37% of the metabolites do not coincide with the fragmentation patterns of known metabolites reported in the Natural Product Dictionary (NPD). As an auxiliary tool in the search and elucidation of the chemical structure of novel natural products, the analysis and study of the genomes of our actinobacteria has allowed the identification of interesting cluster of biosynthetic genes that would be associated with secondary metabolites not reported in NPD. 

#### **Bioactivity and Metabolome Profile of Marine Microorganisms Isolated from Arctic Deep-Sea Sediments** 

MagotFlorent^1^Van SoenGwendoline^1^BlümelMartina^1^SoltwedelThomas^2^TasdemirDeniz^1^,^3^1GEOMAR Centre for Marine Biotechnology (GEOMAR-Biotech), Research Unit Marine Natural Products Chemistry, GEOMAR Helmholtz Centre for Ocean Research Kiel, Am Kiel-Kanal 44, 24106 Kiel, Germany2Alfred-Wegener-Institute Helmholtz Center for Polar and Marine Research, Dept. Deep-Sea Ecology and Technology, Am Handelshafen 12, 27570 Bremerhaven, Germany3Kiel University, Christian-Albrechts-Platz 4, 24118 Kiel, Germany

The deepsea (>1000 m water depth) constitutes more than 60% of the ocean’s biosphere and harbors an unparalleled biodiversity. Because of the high pressure, darkness and low nutrient availability, the deepsea represents an extreme environment for organisms, requiring excellent adaptation capability. Microorganisms that have the ability to thrive in deep-sea environment are regarded promising for biodiscovery. Herein, we studied microorganisms obtained from Arctic deep-sea sediment as a source of new bioactive metabolites. 70 bacterial and 7 fungal strains were isolated and identified from sediment samples collected by a ROV in the Fram Strait, Arctic Ocean (2432 m water depth) during RV Polarstern expedition PS108 in 2017. In order to activate different biosynthetic gene clusters and enhance chemical diversity, the strains were cultivated in four different solid media. The cultures were extracted with EtOAc and the extracts were analyzed by UPLC-MS/MS. Molecular networking was used successfully for comparative metabolomics of microorganisms grown in different media. The crude extracts were screened against a panel of clinically relevant microbial pathogens and six cancer cell lines. Two fungi and two bacteria showed selective activity against the yeast *Candida albicans*, the Gram-negative bacterium *Pseudomonas aeruginosa* and the melanoma cancer cell line A375. Interestingly, for three of the strains, the bioactivity occurred only when grown in one specific media, showing the importance of culture conditions. This presentation will outline the metabolome and bioactivity analyses of the Artic deep-sea sediment microorganisms.

##### **Acknowledgments** 

Funding was provided in frame of EU-H2020 as part of the MSCA-ITN project “MarPipe”.

#### **Can Well Studied Geographic Locations Still Provide Opportunities for Natural Product Discovery?—An Australian Case Study** 

UrbanSylviaLeverJamesMarine and Terrestrial Natural Products Research Group, School of Science (Applied Chemistry and Environmental Science), RMIT University, GPO Box, Melbourne, VIC 2476, Australia

**Figure 21:**
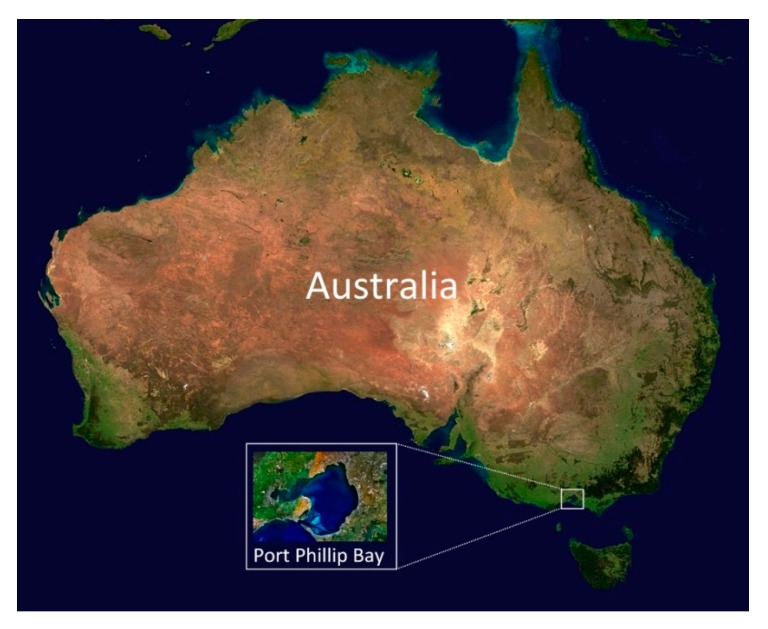
Location of Port Phillip Bay region in Victoria, Australia.

Southern Australian shores are home to more than 140 species of green algae, 240 species of brown algae and 800 species of red algae, making this region one of the most species diverse coastlines in the world [1]. Port Phillip Bay, located on the southern shore of Australia (Figure 21), has an area of approximately 2000 square kilometres and an average depth of 13 m. It has approximately 120 different species of macroalgae, represented by all three major phyla of brown, red and green algae. Since the first study to date 47% of these species, yielding an array of natural products, have been studied for phytochemical purposes. 

In the field of natural products discovery, sampling species from well studied geographical locations potentially decreases opportunity for the discovery of new bioactive secondary metabolites. Does this hypothesis hold true for Port Phillip Bay? A review of the discovery of natural products from marine algae studied in this region, region has been conducted [2]. Case studies demostrating the impact of new methodologies and technologies on the discovery rate will be presented to better understand what we might expect from this biodiverse region in the future. 

References

1. Edgar, G. *Australian Marine Life: The Plants and Animals of Temperate Waters*; Reed New Holland: Sydney, NSW, Australia, 2000.
2. Lever, J.; Brkljača, R.; Urban, S. Natural Products of the Common Marine Algae of Port Phillip Bay, Australia. (Manuscript in preparation).

#### **Stereochemical Study of Spongosoritin A by Vibrational Circular Dichroism and Quantum Chemical Calculations** 

BatistaAndrea N. L.^1^dos SantosFernando M.Jr.^1^BatistaJoao M.Jr.^2^ValverdeAlessandra L.^1^1Chemistry Institute, Fluminense Federal University, Niteroi, Rio de Janeiro 24020-141, Brazil2Institute of Science and Technology, Federal University of Sao Paulo, Sao Jose dos Campos, Sao Paulo 12231-280, Brazil

**Figure 22:**
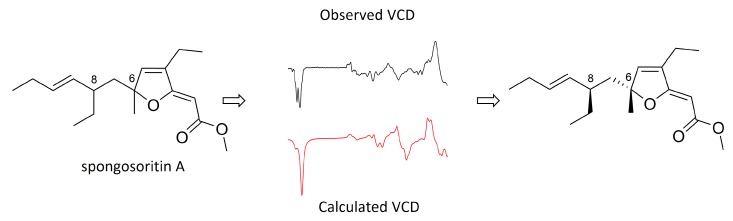
Comparison of experimental and calculated VCD of ( )-(6R,8R)-spongosoritin A.

The polyketide spongosoritin A (**1**) was first reported in 2005 from *Plakortis angulospiculatus* and *Spongosorites* sp. marine sponges [1,2]. This compound is moderately cytotoxic against colon cancer cells (HCT-116) with time-dependent activity [3]. It has been suggested that structural features, such as relative and absolute configurations, could affect the selectivity of the reported cytotoxicity by the activation of different pathways [3]. To date, the relative and absolute configuration of natural spongosoritin A has been suggested to be *syn*-(6*R*,8*R*) based on the comparison of its optical rotation values with that of others marine polyketides with furanylidene moiety as well as the synthetic **1** [4]. However, the determination of absolute configuration based on optical rotation values measured in single wavelengths may result in misassignments [5,6]. Thus, in this work we have applied a direct and efficient method for the determination of the absolute configuration of the natural spongosoritin A using vibrational circular dichroism, NMR and optical rotation, all associated with quantum chemical calculations (Figure 22).

References

1. Epifanio, R.A.; Pinheiro, L.S.; Alves, N.C. Polyketides from the marine sponge *Plakortis angulospiculatus*. *J. Braz. Chem. Soc.*
**2005**, *16*, 1367.
2. Capom, R.J.; Singh, S.; Ali, S.; Sotheeswaran, S. Spongosoritin A: A New Polyketide from a Fijian Marine Sponge, *Spongosorites* sp. *Aust. J. Chem.*
**2005**, *58*, 18.
3. Santos, E.A.; Quintela, A.L.; Ferreira, E.G.; Sousa, T.S.; Pinto, F.D.C.L.; Hajdu, E.; Carvalho, M.S.; Salani, S.; Rocha, D.D.; Wilke, D.V.; et al. Cytotoxic Plakortides from the Brazilian Marine Sponge Plakortis angulospiculatus. *J. Nat. Prod.*
**2015**, *78*, 996.
4. Alcock, L.J.; Norris, M.D.; Perkins, M.V. Total synthesis and structural elucidation of spongosoritin A. *Org. Biomol. Chem.*
**2018**, *16*, 1351.
5. Joyce, L.A.; Nawrat, C.C.; Sherer, E.C.; Biba, M.; Brunskill, A.; Martin, G.E.; Cohen, R.D.; Davies, I.W. Beyond optical rotation: what’s left is not always right in total synthesis**.**
*Chem. Sci.*
**2018**, *9*, 415.
6. Nakahashi, A.; Yaguchi, Y.; Miura, N.; Emura, M.; Monde, K. A Vibrational Circular Dichroism Approach to the Determination of the Absolute Configurations of Flavorous 5-Substituted-2(5*H*)-furanones. *J. Nat. Prod.*
**2011**, *74*, 707.

#### **Secondary Metabolites from Marine Antarctic Fungus *Arthrinium* sp. and their Photoprotective and Antioxidant Potential** 

JordãoA. C.^1^GasparL. R.^1^Marzocchi-MachadoC. M.^1^CanicobaN. C.^1^ColepicoloP.^2^DebonsiH. M.^1^1School of Pharmaceutical Sciences of Ribeirão Preto, University of São Paulo, Ribeirão Preto, São Paulo 14040-903, Brazil2Institute of Chemistry of University of São Paulo, São Paulo 05508-000, Brazil

**Figure 23:**
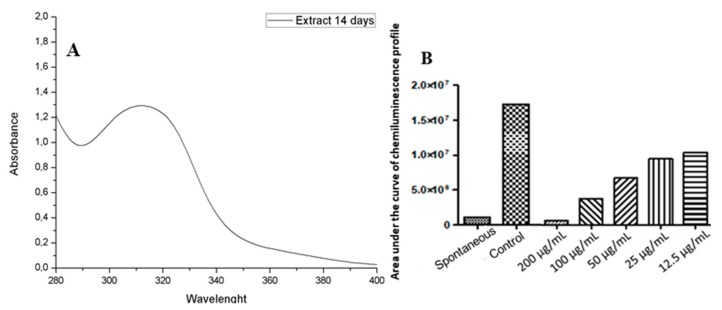
UV absorbance (**A**) and antioxidant activity (**B**) of the extract.

The conditions of the Antarctic continent are extreme; therefore, the organisms produce secondary metabolites necessary for survival in these severe conditions, which comprises in a rich source of new natural products. This study aimed to isolate and identify the secondary metabolites from the extract of the fungus *Arthrinium* sp., besides the biological potential evaluation. The fungus was cultivated in PDB medium and artificial sea water. The extract was obtained in AcOEt, and then submitted to vacuum chromatographic column, using a polarity gradient solvent system Hex: AcOEt. The procedures for the substances’ isolation were carried out using chromatographic techniques, such as TLC and HPLC. The metabolites identification was performed by NMR, spectroscopy in the Infrared and Ultraviolet regions, and mass spectrometry. The photoprotection and photodegradation of the material were determined by analyzing the absorption spectra in a spectrophotometer (range of 200–400 nm). The antioxidant activity was analyzed by the effect of the sample on the production of reactive oxygen species (ROS) by neutrophils, measured by chemiluminescence assay. The results can be found in Figure 23A,B. Among the isolated six substances, one fraction was identified as 2,3,4,6,8-pentahydroxy-1-methylxanthone. The extract and some fractions showed a potential photoprotective activity having good absoption in the UVB (280–320 nm), and UVA (320–400 nm) regions. The extract also showed antioxidant activities, in a concentration 100 μg/mL with a viability of 94.5%. The evaluation potential is being carrying out for the isolated substances. 

#### **Genome Mining and Chemical Analysis of Lipopeptides from a *Bacillus* Strain Isolated from a New *Spongia* Species** 

KyritsiAntigoni-Angeliki^1^TrindadeMarla^2^ReyesFernando^3^EbelRainer^1^JasparsMarcel^1^1Marine Biodiscovery Centre, Department of Chemistry, University of Aberdeen, Aberdeen AB24 3UE, UK2Institute for Microbial Biotechnology and Metagenomics (IMBM), Department of Biotechnology, University of the Western Cape, Bellville, Cape Town 7535, South Africa3Fundación MEDINA, Centro de Excelencia en Investigación de Medicamentos Innovadores en Andalucía, 18916 Granada, Spain

Natural products play a key role when it comes to the discovery of novel bioactive compounds. With terrestrial resources gradually becoming exhausted efforts have focused on expanding the isolation sources to the marine environment instead [1,2]. The world’s ocean covers 70% of the Earth’s surface and provides habitat to a variety of marine organisms that represent a vast untapped resource of novel bioactive natural products. Within them, *Bacillus* species has proven to be a prolific source of a distinguished class of cyclic lipopeptides (CLPs), which are known for their broad spectrum of antibacterial activity and low toxicity and can be mainly divided into three groups, surfactins, iturins and fengycins [3]. In the present work, *Bacillus* strain PE8-15 which was isolated from the sponge, *Spongia* sp. 001RSASPN sampled from low profile rocky reefs in Algoa bay White sands reef (South Africa) at depths of 23–25 metres, was originally subjected to PCR screening at Prof. Marla Trindade’s laboratory, revealing the presence of a putative NRP-PKS hybrid pathway, assumed to be responsible for the biosynthesis of a new lipopeptide. Fractionation by column chromatography of a crude extract obtained using HP-20 solid phase extraction, followed by HR-LCMS analysis of the produced fractions, indicated the presence of several lipopeptides. Further HPLC fractionation led to the isolation of several of them, among others two with m/z 1031.5146 and 1045.5301, corresponding to the molecular formulae C_48_H_74_N_6_O_19_, and C_49_H_76_N_6_O_19_, respectively, which indicates that they could represent bacillomycin D analogues. 2D NMR and LC-MS/MS experiments are currently being performed on them to determine their structures

References

1. Bernan, V.S.; Greenstein, M.; Maiese, W.M. Marine microorganisms as a source of new natural products. *Adv. Appl. Microbiol*. **1997**, *43*, 57–90.
2. Imhoff, J.F.; Labes, A.; Wiese, J. Bio-mining the microbial treasures of the ocean: New natural products. *Biotechnol. Adv*. **2011**, *29*, 468–482.
3. Xu, B.-H.; Lu, Y.-Q.; Ye, Z.-W.; Zheng, Q.-W.; Wei, T.; Lin, J.-F.; Guo, L.Q. Genomics-guided discovery and structure identification of cyclic lipopeptides from the *Bacillus siamensis* JFL15. *PLoS ONE*
**2018**, *13*, e0202893.

#### **Fractions Isolated from Octopus (*Paraoctopus vulgaris*): Identification and Chemopreventive Studies** 

Burgos-HernándezArmando^1^Cruz-RamírezSusana-Gabriela^1^López-SaizCarmen-María^1^García-RomoJoel-Said^1^Rosas-BurgosEma-Carina^1^Cinco-MoroyoquiFrancisco-Javier^1^VelázquezCarlos^2^HernándezJavier^3^1Departamento de Investigación y Posgrado en Alimentos, Universidad de Sonora, Hermosillo, Sonora 83000, Mexico2Departamento de Ciencias Químico-Biológicas, Universidad de Sonora, Hermosillo, Sonora 83000, Mexico3Unidad de Servicios de Apoyo en Resolución Analítica, Universidad Veracruzana, Xico, Veracruz 94294, Mexico

The chemopreventive potential of hexanic (HP) and methanolic (MP) phases from a hexane-soluble extract of *Paraoctopus vulgaris* was studied. The HP was found to be antimutagenic in the Ames assay using *Salmonella typhimurium* TA98 and TA100 tester strains and aflatoxin B1 as control mutagen. After silica gel-column chromatography, 7 fractions (F1–F7) resulted from HP, finding F6 and F7 to be antimutagenic in both tester strains. Since a high antimutagenic activity (85–97% reversion inhibition) was observed in TA98 strain at 3 μg/mL, F6 and F7 were analyzed using HPLC/photodiode array. A peak at 9.42 min was observed in both fractions suggesting a single compound in common. After ^1^H-NMR and ^13^C-NMR analysis, bis (2-ethylhexyl) phthalate (DEHP) was suggested as the potential bioactive compound. Based on the above, a chemopreventive bioassay using ENU-induced mammary tumor Sprague-Dawley female rats was performed using commercially acquired DEHP. Results suggested that DEHP might have a chemopreventive activity in the model used; however, further investigation must be carried out for full bioactivity assessment.

#### **Omics Integration for Bioguided Natural Product Isolation from *Serratia proteamaculans*** 

SimonBegrem^1^,^2^DelphinePasserini^2^ChristineDelbarre-Ladrat^2^OlivierGrovel^1^1Nantes Université, Faculty of Pharmacy, MMS, 9 Rue Bias, 44035 Nantes, France2Ifremer, Rue de l’Île d’Yeu, 44311 Nantes, France

Antimicrobial resistance has become a major health concern over the world, and new antimicrobial drugs with new mechanisms of action are urgently needed. Natural products from marine bacteria are a recognised and still promising source of bioactive compounds. One of the way to reach the discovery of such products consists in studying chemical interactions within complex microbial ecosystems such as the marine microbiome. In this way, following an initial screening of bacterial strains isolated from seafood products, we performed co-cultures of active bacteria against pathogens in order to induce the activation of cryptic biosynthetic gene clusters (BGCs) encoding antimicrobial compounds. 

A marine *Serratia proteamaculans* strain has been selected, which exhibited antimicrobial activity against both bacterial and fungal pathogens, such as *Staphylococcus epidermidis*, *Aerococcus salmonicidae*, *Aspergillus fumigatus* and *Candida albicans*. In order to detect putatively original bioactive natural products produced by this understudied species, we engaged a strategy combining genome mining and biological assays, together with metabolomics analysis of co-cultures of *S. proteamaculans* with target bacteria. In silico genome mining of *S. proteamaculans* allowed the detection of 9 BGCs among which 6 are undescribed, showing the high metabolic potential of the strain in terms of structural novelty. LC-HRMS metabolic profiles of *Serratia/Staphylococcus* co-cultures were compared to those of individual pure cultures of the two bacteria: metabolomics analyses exhibited an increased production of several metabolites but particularly the de-novo induction of 2 non-dereplicated compounds in co-culture samples. Further purification of these compounds and co-cultivation with other organisms are ongoing.

#### **New Scalarane-Type Sesterterpenoids from Marine Sponge *Lendenfeldia* sp.** 

PengBo-Rong^1^,^2^,^3^DuhChang-Yih^4^SungPing-Jyun^3^,^5^1Doctoral Degree Program in Marine Biotechnology, National Sun Yat-Sen University, Lien-Hai Road, Kaohsiung 804, Taiwan2Doctoral Degree Program in Marine Biotechnology, Academia Sinica, Academia Road, Taipei 115, Taiwan3National Museum of Marine Biology & Aquarium, Pingtung 944, Taiwan4Department of Marine Biotechnology and Resources, National Sun Yat-Sen University, Lien-Hai Road, Kaohsiung 804, Taiwan5Graduate Institute of Marine Biotechnology, National Dong Hwa University, Pingtung 944, Taiwan

**Figure 24:**
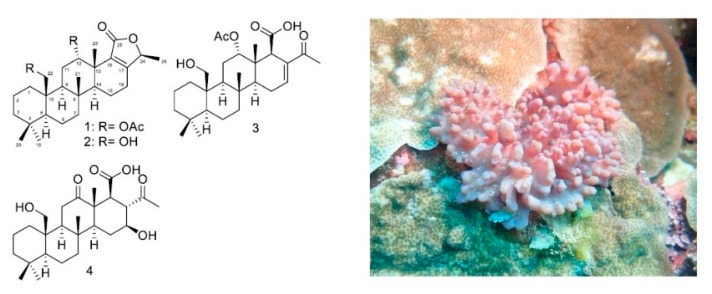
Structure of lendenfeldarane A–D (**1**–**4**), and a picture of marine sponge *Lendenfeldia* sp.

Marine sponge-derived natural products possess many classes of secondary metabolites. For instance, there were 18 identified chemical classes of the isolated new compounds during the last decade, including acid, alkaloid, ester, fatty acid, glycoside, ketone, lipid, macrolide, alcohol, peptide, peroxide, polyketide, quinone, steroid, sterol, terpene, terpenoid and unclassified. Among them, scalarane-type sesterterpenoids emerged as an interesting group of terpenoids isolated from marine sponges and shell-less mollusks. Scalarane sesterterpenoids demonstrated a wide spectrum of interesting biological properties, such as anti-inflammation, cytotoxicity, anti-feedant, anti-microbial, anti-fungal, ichthyotoxicity, anti-tubercular, anti-HIV, anti-fouling, inhibition of platelet-aggregation, inhibition of transactivation for the nuclear hormone receptor (FXR, farnesoid X-activated receptor), and stimulation of nerve growth factor synthesis. In the further study of metabolites from marine sponge *Lendenfeldia* sp, four new 24-homoscalarane analogues, lendenfeldarane A–D (**1**–**4**) (Figure 24) and eight known scalarane-type sesterterpenoids were isolated. The structures of scalaranes **1**–**12** were elucidated on the basis of spectroscopic analysis. Cytotoxicity of scalaranes **1**–**12** against the proliferation of a limited panel of tumor cell lines was evaluated. 

#### **Ubiquitin-Proteosome Modulating Dolabellanes from Formosan Marine Soft Corals** 

DuhChang-Yih^1^LingXue-Hua^1^WangShang-Kwei^2^1Department of Marine Biotechnology and Resources, National Sun Yat-Sen University, Kaohsiung 80441, Taiwan2Department of Microbiology and Immunology, Kaohsiung Medical University, Kaohsiung 80708, Taiwan

Marine soft corals have evolved unique characteristics in metabolic and physiological capabilities to produce secondary metabolites that may function in defense, food capture, interference competition. Soft coral-derived metabolites exhibit diverse biological activities such as cytotoxicity, inhibition of inflammatory reaction, anti-microbial and anti-viral activities. The ubiquitin-proteasome system (UPS) is a major intracellular, nonlysosomal proteolytic system that is involved in many important cellular pathways, including protein quality control, protein homeostasis, DNA repair, cell cycle progression, pathogen infection, transcriptional regulation, cellular differentiation, and immune modulation. Therapeutic drugs, Bortezomib, Carfilzomib and Ixazomib, target UPS have been licensed in treating multiple myeloma. Moreover, UPS inhibitors are demonstrated to attenuate the progression of neural degenerative disease. Therapeutic prospect of UPS inhibitor is promising and valuable. HCMV UL76 interacts with proteasome regulatory subunits of 26S proteasome. Fluorescence intensities and the phenotypic behaviors of UL76-aggresome are used as markers for proteasome inhibition. We have established a cell-based high-content drug screening assay direct-acting UPS and assessed dolabellanes isolated from Formosan soft corals. Four of them were shown to modulate EGFP-UL76 high-content profile in comparative to proteasome inhibitors MG132 and bortezomib.

#### **Antimicrobial Peptides and Peptidyl Analogues from Marine Microorganisms** 

LiawChih-Chuang^1^,^2^LienYa-Chu^1^HungMau-Xuan^1^1Department of Marine Biotechnology and Resources, National Sun Yat-sen University, Kaohsiung 80424, Taiwan2Doctoral Degree Program of Marine Biotechnology, National Sun Yat-sen University, Kaohsiung 80424, Taiwan

The indiscriminate worldwide overuse and misuse of antibiotics has led to high rates of microbial resistance so that the search of novel antimicrobial molecules to overcome the resistance phenomenon is urgently needed to human health. Based on the evolution view, antimicrobial peptides (AMPs) and their analogues are powerful weapons in either unicellular organism, such as bacteria, or multicellular organisms, such as fungi, plants, and animals to serve a protection role for themselves. Therefore, naturally-occurring AMPs and peptidyl analogues from marine microorganims could be considered as suitable templates for the development of alternatives to conventional antibiotics. By the global natural product social molecular networking (GNPS) analysis, we find a series of new AMPs, peptaibols, from the marine-derived fungus, *Trichoderma* sp. and peptidyl analogues, pesudopeptides, from *Vibrio* sp. All the structures of these peptide analogues are elucidated by 1D and 2D NMR, MS, and CD spectra. Their antimicrobial activity of these compounds is under investigation.

#### **Approaches for Accessing and Studying the Full Encoded Natural Product Potential of Marine Proteobacteria** 

RossAvena C.Department of Chemistry, Queen’s University, Kingston, ON K7L 3N6, Canada

Bacteria have long been an excellent source of bioactive natural product molecules that find application in therapeutics and drug development. Marine microbes in particular represent a promising and relatively untapped reservoir of natural products, however, there are still several challenges in the field of bacterial natural products discovery. Difficulties associated with cultivation of microbes in the artificial laboratory environment and also challenges gaining access to the full biosynthetic potential of bacteria in the lab remain bottlenecks in the effective and expedient exploitation of natural products as drug leads. We are currently developing ways to not only facilitate growth of marine bacteria but also to expand the range of compound classes and number of molecules they produce during lab cultivation. This presentation will discuss approaches we are taking to prioritize the most fruitful strains to study, cultivation based strategies to facilitate molecule production/discovery and genetic methods for inducing expression of silent biosynthetic gene clusters to allow access to the full scope of encoded metabolites. 

#### **Search for Bioactive Compounds from Marine-Derived Fungus *Emericellopsis maritima* through Osmac Techniques** 

Pinedo-RivillaCristinaNúñezMaría J.MillánCarlosAleuJosefinaDuránRosaColladoIsidro G.Department of Organic Chemistry, Facultad de Ciencias, Campus Universitario Río San Pedro s/n, Torre sur, 4º planta, Universidad de Cádiz, 11510 Puerto Real, Cádiz, Spain

The wide marine biodiversity and the relative lack of research on its resources have promoted in recent years the search for new compounds with biological activity, especially antibiotics due to the resistance acquired by several microorganisms to those already available in the market.

Marine derived fungi are proving to be an important source of compounds with interesting biologial activities. Furthermore, from the chemical point of view, they can be used as biocatalysts, producers of enzymes and bioremediators.

In this study, the secondary metabolism of the marine-derived fungus *Emericellopsis maritima*, isolated from sediments samples collected along an intertidal gradient of Bay of Cádiz, was studied as possible source of new compounds with pharmacological activity [1].

**Figure 25:**
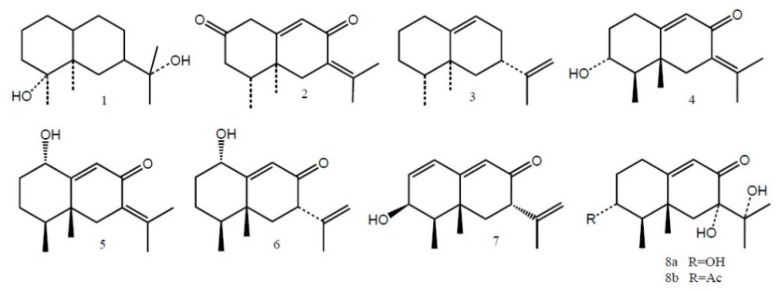
Isolated metabolites from *Emericellopsis maritima*.

A new approach using metabolomic analysis was incorporated to target the isolation of the bioactive compounds. Following the OSMAC (One Strain, Many Compounds) [2] approach, the fungus was grown using different cultivation conditions. The purification of the crude extracts afforded the sesquiterpenes with eremophilane skeleton **1**–**8** (Figure 25), which are known to show important biological activities [3,4].

References

1. Inostroza, A.; Lara, L.; Paz, C.; Perez, A.; Galleguillos, F.; Hernandez, V.; Becerra, J.; González Rocha, G.; Silva, M. Antibiotic activity of Emerimicin IV isolated from Emericellopsis minima from Talcahuano Bay, Chile. *Nat. Prod. Res.*
**2018**, *32*, 1361–1364.
2. Romano, S.; Jackson, S.A.; Patry, S.; Dobson, A.D.W. Extending the “One Strain Many Compounds” (OSMAC) Principle to Marine Microorganism. *Mar. Drugs*
**2018**, *16*, 244.
3. Daengrot, C.; Rukachaisirikul, V.; Tansakul, C.; Thongpanchang, T.; Phongpaichit, S.; Bowornwiriyapan, K.; Sakayaroj, J. Eremophilane Sesquiterpenes and Diphenyl Thioethers from the Soil Fungus Penicillium copticola PSU-RSPG138. *J. Nat. Prod.*
**2015**, *78*, 615–622.
4. Shi, X.; Zhang, S.; Wang, J.; Li, T.; Liu, J.; Hou, Y. Synthesis and Bioactivity of Natural Occurring Petasin-Like Derivatives as Antitumor Agents. *Open J. Med. Chem.*
**2015**, *5*, 23–31.

#### **GC- and UHPLC-MS Profiles as a Tool to Valorizate the Red Alga *Asparagopsis armata*** 

LesenfantsMarie L.^1^SecaAna M. L.^1^,^2^,^3^SilvaArtur M. S.^3^PintoDiana C. G. A.^3^1Faculty of Sciences and Technology, University of Azores, Rua Mãe de Deus, 9501-321 Ponta Delgada, Portugal2cE3c-Centre for Ecology, Evolution and Environmental Changes/Azorean Biodiversity Group, Rua Mãe de Deus, 9501-321 Ponta Delgada, Portugal3QOPNA & LAQV-REQUIMTE, Department of Chemistry, University of Aveiro, Campus de Santiago, 3810-193 Aveiro, Portugal

*Asparagopsis armata* is considered a biological invader and this red alga is in the last few years one of the worst nightmares for Azores coast biodiversity. So efforts to find an economically valuable application are welcome. In this context biological evaluations of its extracts, such as anti-aging, antioxidant and anticholinesterasic activities, were recently presented [1,2]. Naturally, the knowledge of this species chemical composition is utmost importance not only to find some valuable utilization but also to discovery its mechanisms of defence that can explain its invasive behaviour. In our effort to contribute to this problem solution we establish the GC-MS and UHPLC-MS profiles of both the non-polar and polar extracts. The main compounds in the lipophilic extract were palmitic acid and 1-monopalmitin and brominated compounds dominate both extracts. The detailed results will be presented and discussed in the presentation. 

##### **Acknowledgments** 

This study was financed by ASPAZOR project (DRCT: ACORES- 01-0145-FEDER-00060- ASPAZOR); Portuguese National Funds, through FCT –Fundação para a Ciência e a Tecnologia, and as applicable co-financed by the FEDER within the PT2020 Partnership Agreement by funding the Organic Chemistry Research Unit (QOPNA) (UID/QUI/00062/2013) and the cE3c centre (FCT Unit funding (Ref. UID/BIA/00329/2013, 2015–2018) and UID/BIA/00329/2019).

References

1. Lesenfants, M.L.; Rosa, G.P.; Barreto, C.; Seca, A.M.L. *Asparagopsis armata* dichloromethane extract: Biological activity and chemical composition. In Proceedings of the 1st Seaweed for Health Conference, Galway, Ireland, 24–27 June 2018; p. 13.
2. Fonseca, A.R.; Ponte, J.; Barreto, C.; Pinto, D.C.G.A.; Seca, A.M.L. Polar extracts of *Asparagopsis armata*: Anti-aging activity and chemical composition by LC-MS. In Proceedings of the 1st Seaweed for Health Conference, Galway, Ireland, 24–27 June 2018; p. 50.

#### **Polyketides and a Dihydroquinolone Alkaloid from a Marine-Derived Strain of the Fungus *Metarhizium marquandii*** 

El-KashefDina H.^1^,^2^DaletosGeorgios^1^PlenkerMalte^3^HartmannRudolf^3^MándiAttila^4^KurtánTibor^4^WeberHorst^5^LinWenhan^6^AncheevaElena^1^ProkschPeter^1^1Institut für Pharmazeutische Biologie und Biotechnologie, Heinrich-Heine-Universität Düsseldorf, Universitätsstrasse 1, 40225 Düsseldorf, Germany2Department of Pharmacognosy, Faculty of Pharmacy, Minia University, Minia 61519, Egypt3Institute of Complex Systems: Strukturbiochemie, Forschungszentrum Jülich GmbH, ICS-6, 52425 Jülich, Germany4Department of Organic Chemistry, University of Debrecen, POB 400, 4002 Debrecen, Hungary5Institut für Pharmazeutische und Medizinische Chemie, Heinrich-Heine-Universität Düsseldorf, Universitätsstrasse 1, 40225 Düsseldorf, Germany6State Key Laboratory of Natural and Biomimetic Drugs, Peking University, Beijing 100191, China

Three new natural products (**1–3**), including two butenolide derivatives (**1** and **2**) and one dihydroquinolone derivative (**3**), together with nine known natural products were isolated from a marine-derived strain of the fungus *Metarhizium marquandii.* The structures of the new compounds were unambiguously deduced by spectroscopic means including HRESIMS and 1D/2D NMR spectroscopy, ECD, VCD, OR measurements and calculations. The absolute configuration of marqualide (**1**) was determined by a combination of modified Mosher’s method with TDDFT-ECD calculations at different levels. The (3*R*,4*R*) absolute configuration of aflaquinolone I (**3**), determined by, O.R.; ECD and VCD calculations, was found to be opposite to the (3*S*,4*S*) absolute configuration of the related aflaquinolones A-G. The absolute configuration of the known natural product, terrestric acid hydrate (**4**), was likewise determined for the first time in this study. TDDFT-ECD calculations allowed determination of the absolute configuration of its chirality center remote from the stereogenic unsaturated γ-lactone chromophore. 

**Figure:**
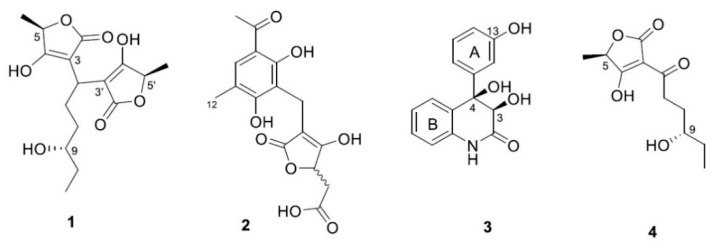
**Graphical Abstract**

##### **Acknowledgments** 

D.H.E.-K. gratefully acknowledges the Egyptian Government (Ministry of Higher Education) for awarding the doctoral scholarship. Financial support by the Manchot Foundation to P.P. is gratefully acknowledged.

#### **Diketopiperazines, Tetralones and Two Novel Valerolactam Derivatives from Endophytic Fungi Associated to the Marine Red Alga *Asparagopsis taxiformis*** 

SilvaDulce^1^MendonçaIatã^1^RufinoVictor^1^HonórioAlana^1^MedinaRebeca^1^YokoyaNair^2^1Institute of Chemistry, São Paulo State University—UNESP, Araraquara 14800-060, Brazil2Institute of Botany, SMA, Sao Paulo 0401-902, Brazil

Marine organisms from Brazilian coast and their endophytes have received growing attention during the past few decades. Our recent work on endophytic fungi from marine algae has confirmed their potential as interesting sources of bioactive compounds as well as their marked chemodiversity. Two fungal strains, *Hypoxylon investiens* and *Humicola fuscoatra*, were isolated from the marine red alga *Asparagopsis taxiformis*, collected at Fortaleza beach, in Ubatuba (SP, Brazil). After surface sterilization, fragments of the alga were spread on PDA or Czapek medium plates, and seawater or ultrapure water was used for fungal strains cultivation.

Subsequent micelia separation and partition of each extract, led to aqueous (AqFr), acetonitrile (MeCNFr) and hexane (HexFr) fractions, which were then assayed and showed moderate cytotoxic and anticholinesterase bioactivities. Fractionation of the *Humicola fuscoatra* extract over C-18 silica-gel CC, gradient mode, yielded 7 fractions, which were purified by HPLC-DAD-UV and afforded two isocoumarins, the diketopiperazine cycle (Phe-Pro), in addition to two novel valerolactam derivatives, with moderate cytotoxic activity. HRESIMS and detailed NMR spectral analyses, including ^1^H-^15^N HMBC experiments, indicated its molecular formulae, C_12_H_17_NO_4_, consistent with a novel dienone-valerolactam derivative. *Hypoxylon investiens* extract fractionation over Sephadex LH-20 and purification by HPLC afforded 5-methylmelein, nodulisporone, isosclerone, two diketopiperazines, in addition to a novel phenolic benzofuran derivative. Their structures were established from UV and detailed NMR spectral analyses, and confirmed from HRESI-TOF-MS data. Such results highlight the outstanding chemodiversity of marine-derived fungi, which may contribute for bioprospection efforts and sustainable exploration of Brazilian marine biodiversity.

#### **Bioactive Bromotyrosine Metabolites from the Marine Sponge *Aplysinella rhax*** 

OluwabusolaEmmanuel^1^TabudravuJioji^1^FeussnerKlaus^2^AnnangFreddie^3^ReyesFernando^3^Pérez-MorenoGuiomar^4^Ruiz-PérezLuis^4^González-PacanowskaDolores^4^EbelRainer^1^JasparsMarcel^1^1Marine Biodiscovery Centre, Department of Chemistry, University of Aberdeen, Aberdeen AB24 3UE, Scotland, UK2Institute of Applied Sciences, Faculty of Science, Technology and Environment, University of the South Pacific, Suva, Fiji3Fundación MEDINA, Parque Tecnológico de Ciencias de la Salud, Avenida del Conocimiento, 34.18016-Armilla, 18016 Granada, Spain4Instituto de Parasitología y Biomedicina “López-Neyra”, Consejo Superior de Investigaciones Científicas, Parque Tecnológico de Ciencias de la Salud, Avenida del Conocimiento, s/n, 18016-Armilla, 18016 Granada, Spain

**Figure 26:**
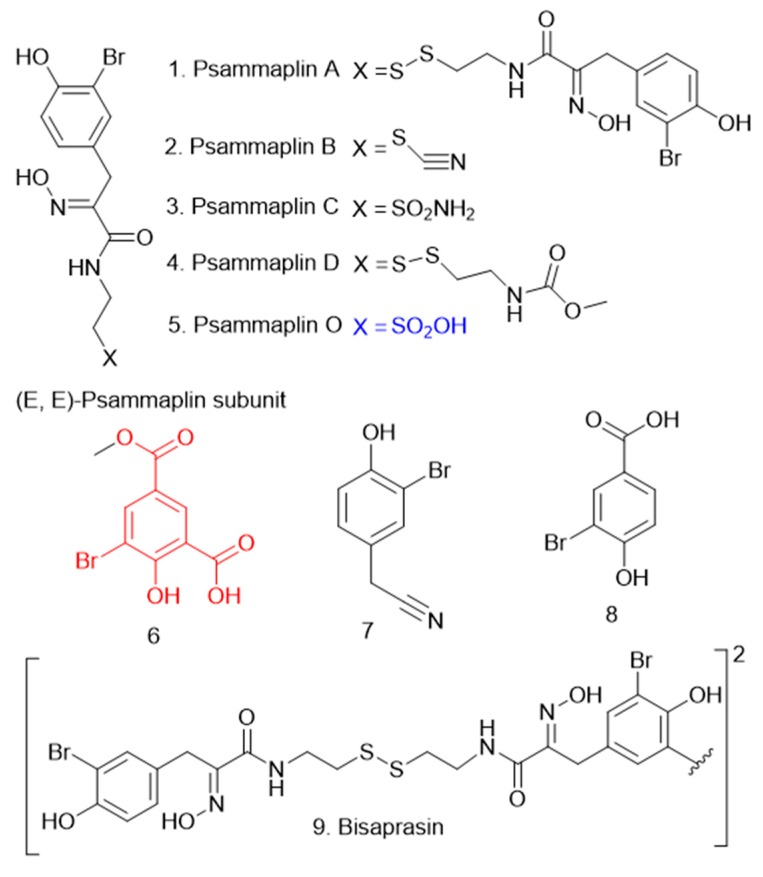
The structure of compounds **1**–**9**.

Marine sponges are highly diverse and capable of biosynthesizing a greater variety of natural bioactive products than other invertebrate phyla. Those belonging to the family Verongida are known producers of brominated secondary metabolites. The methanolic extract of the Fijian marine sponge *Aplysinella rhax* collection was fractionated and purified by reversed phase HPLC to yield 9 bromotyrosine derivatives (Figure 26) including two that have not been described in the literature before: psammaplin O (**5**) and 3-bromo-2-hydroxyl-5-(methoxycarbonyl)benzoic acid (**6**), alongside psammaplins A-D (**1–4**), bisaprasin (**9**), (3-bromo-4-hydroxyphenyl)-acetonitrile (**7**) and bromohydroxybenzoates (**8**). The structures of the new compounds were elucidated by HRMS, and 1D and 2D NMR spectroscopic analysis. The two new compounds exhibited no significant activity against *Trypanosoma cruzi* Tulahuen C4 parasites and *Plasmodium falciparum* 3D7. Psammaplin A and D showed moderate activity against both *T. cruzi* Tulahuen C4 and *P. falciparum* 3D7 with IC_50_ values of 30–43 μM and 60–67 μM respectively. Additionally, compounds **3** and **8** displayed weak activity against *P. falciparum* 3D7 with IC_50_ values of 131 and 115 μM respectively.

#### **Secondary Metabolites from Vietnamees Strain of Marine-Derived Fungus *Aspergillus flocculosus*** 

IvanetsElena V.^1^SmetaninaOlga F.^1^MenchinskayaEkaterina S.^1^PhanTrinh^2^,^3^YurchenkoAnton N.^1^1G.B. Elyakov Pacific Institute of Bioorganic Chemistry Far Eastern Branch of Russian Academy of Sciences, 690022 Vladivostok, Russia2Nhatrang Institute of Technology Research and Application, Vietnam Academy of Science and Technology, Nha Trang 650000, Vietnam3Graduate University of Science and Technology, Vietnam Academy of Science and Technology, Ha Noi 150000, Vietnam

The marine fungi are promising sources of bioactive compounds [1]. Marine habitat affects the biosynthetic processes in the organisms inhabited it, therefore, marine fungi can produce compounds unique in structure. In addition, the coast of Vietnam is an insufficiently studied area of South China Sea, since most researches are focus on the coast of China and Taiwan Strait. 

**Figure 27:**
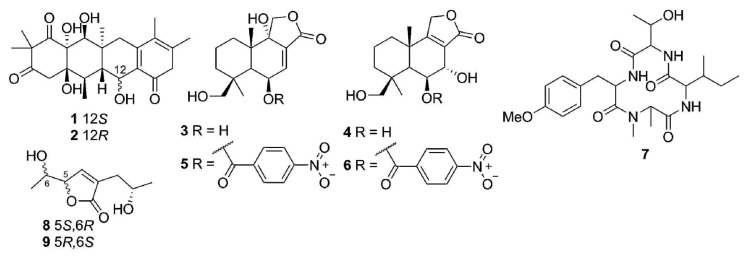
The structures of isolated compounds.

In this research work the metabolite composition of marine-derived fungus *Aspergillus flocculosus* (sediments, Nha Trang Bay, South China Sea) was investigated. As a result, a new merpterpenoid 12-*epi*-aspertetranone D (**1**) (Figure 27) together with its known epimer aspertetranone D (**2**) [2], two new sesquiterpenoids (**3**–**4**) and their known *p*-nitrobenzoate derivatives (**5**–**6**) [3,4], a new cyclic tetrapeptide (**7**), a new tetraketide aspilactonol G (**8**) and two its known isomers aspilactonol F (**9**) and dihydroaspyrone [5], as well as a known diketopiperazine mactanamide [6] were isolated. The structures of isolated compounds were established by combination of 1D and 2D ^1^H and ^13^C NMR and HRESIMS data. 

Compound **5** showed cytotoxic activity against mice neuroblastoma Neuro2A cells and human breast cancer cells MCF-7 with IC_50_ 4.9 and 59.6 μM, respectively, while compound **6** was inactive. Also compounds **3**, **5** and **7** showed cytotoxic activity against human prostate cancer cells 22Rv1 with IC_50_ 35.3, 3.0 and 38.7 μM, respectively. 

##### **Acknowledgments** 

The study was supported by Russian Foundation for Basic Research (Grants No 18-34-00621 for chemical study and 18-34-00737 for bioassays). 

References

1. Wang, K.W.; Ding, P. New Bioactive Metabolites from the Marine-derived Fungi *Aspergillus*. *Mini-Rev. Med. Chem.*
**2018**, *18*, 1072–1094.
2. Wang, J.; Wei, X.; Qin, X.; Tian, X.; Liao, L.; Li, K.; Zhou, X.; Yang, X.; Wang, F.; Zhang, T.; et al. Antiviral Merosesquiterpenoids Produced by the Antarctic Fungus *Aspergillus ochraceopetaliformis* SCSIO 05702. *J. Nat. Prod.*
**2016**, *79*, 59–65.
3. Belofsky, G.N.; Jensen, P.R.; Renner, M.K.; Fenical, W. New cytotoxic sesquiterpenoid nitrobenzoyl esters from a marine isolate of the fungus *Aspergillus versicolor*. *Tetrahedron*
**1998**, *54*, 1715–1724.
4. Fang, W.; Lin, X.; Zhou, X.; Wan, J.; Lu, X.; Yang, B.; Ai, W.; Lin, J.; Zhang, T.; Tu, Z.; et al. Cytotoxic and antiviral nitrobenzoyl sesquiterpenoids from the marine-derived fungus *Aspergillus ochraceus* Jcma1F17. *MedChemComm*
**2014**, *5*, 701–705.
5. Fuchser, J.; Zeeck, A. Secondary metabolites by chemical screening, 34: Aspinolides and aspinonene/aspyrone Co-metabolites, new pentaketides produced by *Aspergillus ochraceus*. *Liebigs Ann.*
**1997**, *1*, 87–95.
6. Lorenz, P.; Jensen, P.R.; Fenical, W. Mactanamide, a new fungistatic diketopiperazine produced by a marine *Aspergillus* sp. *Nat. Prod. Lett.*
**1998**, *12*, 55-60.

#### **Meroditerpenoids Isolated from the North-Eastern Atlantic Macroalga, *Halidrys siliquosa*** 

MurphyElliot^1^CalabroKevin^1^LasserrePerrine^1^ZanollaMarianela^2^StengelDagmar B.^2^ThomasOlivier P.^1^1Marine Biodiscovery, School of Chemistry and Ryan Institute, National University of Ireland Galway University Road, H91TK33 Galway, Ireland2Botany and Plant Science, School of Natural Sciences and Ryan Institute, National University of Ireland Galway University Road, H91TK33 Galway, Ireland

Macroalgae have evolved to produce various defence mechanisms including a chemical defence in the form of small metabolites. *Halidrys siliquosa*, a species of brown algae native to the Atlantic coasts across western Europe, has been shown to contain secondary metabolites exhibiting biological activities in combating various biofilms [1].

**Figure 28:**
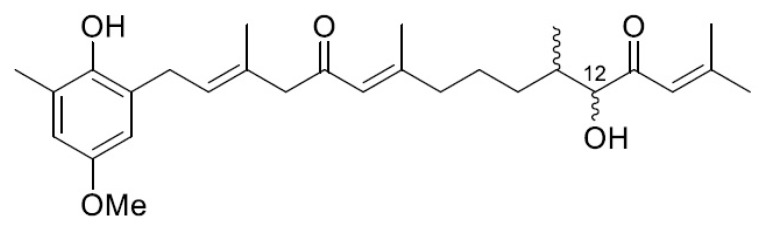
Example of meroditerpenoid isolated from *Halidrys siliquosa*.

The aim of our study was to isolate and characterise the structure of the chemical diversity produced by *H. siliquosa* using a set of analytical techniques. The chemical structures of an outstanding diversity of meroterpenoids present have been first elucidated with 1D and 2D NMR spectroscopic analysis (Figure 28). The question of the absolute configuration was addressed with circular dichroism. A molecular network built on GNPS has also been included in our study to better describe the algae’s metabolome. 

References

1. Culioli, G.; Ortalo-Magné, A.; Valls, R.; Hellio, C.; Clare, A.S.; Piovetti, L. Antifouling activity of meroditerpenoids from the marine brown alga *Halidrys siliquosa*. *J. Nat. Prod.*
**2008**, *71*, 1121–1126.

#### **Search for Marine Natural Products from the Mixtures of Marine Invertebrates in the Twilight Zone** 

NakamuraFumiaki^1^NakaoYoichi^1^,^2^1Graduate School of Advanced Science and Engineering, Waseda University, 3-4-1 Okubo, Shinjuku-ku, Tokyo 169-8555, Japan2Research Institute for Science and Engineering, Waseda University, 3-4-1 Okubo, Shinjuku-ku, Tokyo 169-8555, Japan

The twilight zone, also known as the mesopelagic zone between a depth of 100–1000 m, where sunlight hardly reaches [1]. The extreme environments different from the terrestrial were changed the metabolism in the inhabiting organisms. As the result, secondary metabolites from the twilight zone retain unique structures and bioactivities [2]. However, most of these precious samples are obtained as the crushed and fragmented small pieces that were difficult to be classified because of the collecting way by dredging. Therefore, we focused on the unclassified small fragment mixtures from the twilight zone and searched for new natural products from the extract of the mixture [3]. The fragment mixture (24.6 kg, wet wt.) collected by dredging at Yaku-shinsone (166–167 m), Kagoshima prefecture, Japan, was extracted and tested for the cytotoxicity using the MTT assay. Bioassay guided fractionation of the extracts identified several bioactive substances. On the other sides, metabolome analysis of the extracts from 99 classified dredging samples was performed using LC-MS that lead to the identification of the source organisms of the isolated substances.

References

1. Lee, C. Transformations in the “Twilight Zone” and beyond. *Mar. Chem.*
**2004**, *92*, 87–90.
2. Skropeta, D. Deep-sea natural products. *Nat. Prod. Rep.*
**2008**, *25*, 1131–1166.
3. Machida, K.; Abe, T.; Arai, D.; Okamoto, M.; Shimizu, I.; de Voogd, N.J.; Fusetani, N.; Nakao, Y. Cinanthrenol A, an estrogenic steroid containing phenanthrene nucleus, from a marine sponge *Cinachyrella* sp. *Org. Lett.*
**2014**, *16*, 1539–1541.

#### **Breakthrough Innovative Technologies in Marine Natural Products Investigation** 

Le GoffGéraldine^1^VlachouP.^2^AlonsoC.^3^ÁlvarezP. A.^3^GallardJ.-F.^1^FokialakisN.^2^FelezeuD.^4^TouronA.^4^AllegretbordonC.^4^OuazanniJ.^1^1Institut de Chimie des Substances Naturelles ICSN, Centre National de la Recherche Scientifique, Avenue de la Terrasse, 91198 Gif-sur-Yvette, France2Department of Pharmacognosy & Natural Products Chemistry, Faculty of Pharmacy, National and Kapodistrian University of Athens, 15771 Athens, Greece3iMARE Natural S.L. Avda. Habana, 10, 18600 Motril, Spain4Pierre Guerin Technologies, 79000 Niort, France

TASCMAR is an H2020 EU funded research project that investigates the chemical potential of the ocean’s mesophotic zone. The goal is to develop sustainable methods for discovering chemical compounds that can be used for application in diverse fields such as health/nutrition, depollution and nature-based cosmetics. In the context of TASCMAR, two breakthrough innovations were recently patented. These technologies are dedicated to the optimal production of microbial metabolites and the trapping of invertebrate metabolites in their natural habitat: UNIFERTEX (UNIversal FERmenTor EXpert) [1] offers a unique pilot-scale fermentor to carry out solid and liquid state fermentations in parallel, in a highly controlled environment with dedicated remote supervision and control software. SOMARTEX (Self Operating MARine Trapping EXtractor) [2] is the first technology to trap invertebrate/holobionts molecules in their natural habitat at any location and depth, without harvesting the organism or impacting their surrounding ecosystem. The proof of concept was established in-aquarium with XAD-amberlite solid-phase extraction. We reported the elicitation of guanidine alkaloids production of *Crambe crambe* in the presence of *Anemonia sulcata*, both collected from the Mediterranean Sea. Besides the previously reported crambescidin 359, and crambescidin acid, three new compounds were isolated; one carboxylated analog named crambescidin 401, and two analogs of crambescin B, crambescin B 281 and crambescin B 253 [3].

References

1. Ouazzani, J.; Le Goff, G.; Felezeu, D.; Touron, A.; Allegret-Bourdon, C. Unifertex, Universal Fermentor Expert, Device for Microbial Cultivation. CNRS/Pierre Guerin Technologies. PCT/EP2018/086882. 2018. Available online: https://youtu.be/sfXKRaAl-HU (accessed on May 27 2019).
2. Ouazzani, J.; Le Goff, G. Somartex, Self Operating MARine Trapping Extractor. CNRS, PCT/EP2019/051912. 2019. Available online: https://youtu.be/1Tmh8dFqZ34 (accessed on May 27 2019).
3. Vlachou, P.; Le Goff, G.; Alonso, C.; Álvarez, P.; Gallard, J.F.; Fokialakis, N.; Ouazzani, J. Innovative Approach to Sustainable Marine Invertebrate Chemistry and a Scale-Up Technology for Open Marine Ecosystems. *Mar. Drugs*
**2018**, *16*, 152.

#### **New Ophiobolins, Cytotoxic Sesterterpenes from the Marine Fungus *Aspergillus flocculosus*** 

ShinHee Jae^1^,^2^ChoiByeoung-kyu^1^,^2^KangJong Soon^3^1Department of Marine Biotechnology, University of Science and Technology (UST), 217 Gajungro, Yuseong-gu, Daejeon 34113, Korea2Marine Natural Products Chemistry Laboratory, Korea Institute of Ocean Science and Technology, 385 Haeyang-ro, Yeongdo-gu, Busan 49111, Korea3Laboratory Animal Resource Center, Korea Research Institute of Bioscience and Biotechnology, 30 Yeongudanjiro, Cheongju 28116, Korea

Five new sesterterpenes (1–5) together with four known ophiobolins (6–9) were isolated from the marine fungus *Aspergillus flocculosus* and their structures were elucidated by extensive NMR and MS data analysis and the comparison of chemical shift and optical rotation data with those reported in the literature. Among these ophiobolins, five new ophiobolins were first isolated as the ophiobolin derivatives consisting of three double bonds in the side chain and Z-conformation at C-14/C-15. Isolated ophiobolins were evaluated for cytotoxic activity against six cancer cell lines, HCT-15, NUGC-3, NCI-H23, ACHN, PC-3 and MDA-MB-231. All the compounds showed potent cytotoxicity with GI_50_ values ranging from 0.14~2.01 μM against all the cell lines.

#### **Cadiolide J–M, Antibacterial Polyphenyl Butenolides from the Korean Tunicate *Pseudodistoma antinboja*** 

WangWeihong^1^KimHiyoung^2^PatilRahul S.^1^GiriAwadut G.^1^WonDong Hwan^1^HahnDongyup^1^SungYoujung^1^LeeJusung^1^ChoiHyukjae^1^NamSang-Jip^1^KangHeonjoong^1^1Center for Marine Natural Products and Drug Discovery, School of Earth and Environmental Sciences, Seoul National University, Seoul 08826, Korea2Department of Biomedical Science & Engineering, Konkuk University, 120 Neungdong ro, Gwangjin gu, Seoul 05029, Korea

**Figure 29:**
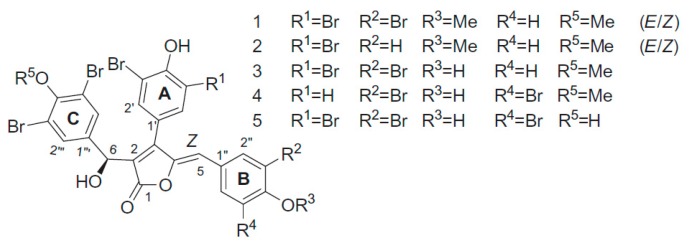
Chemical strucutres of cadiolides.

The tunicates (ascidians) of the genus *Pseudodistoma* (family *Pseudodistomidae*) comprise more than 20 species and are widely distributed in tropical as well as in temperate waters. Although previous investigations of the chemical constituents and bioactivity of tunicates of this genus have led to the discovery of a diverse array of nitrogenous secondary metabolites encompassing piperidine, alkyl amine, amino alcohol, β-carboline, and quinoline alkaloids, fewer examples of metabolites with interesting biological activities have been reported. Recently a chemical investigation conducted by our research group identified a series of antibacterial non-nitrogenous metabolites from the Korean tunicate *Pseudodistoma antinboja*. Activity-guided fractionations of the tunicate *Pseudodistoma antinboja* yielded four new compounds of the cadiolide class (cadiolides J–M, **1**, **3**–**5**) along with a known one (cadiolide H, **2**) (Figure 29). The structures were defined by spectroscopic methods including X-ray crystallographic analysis. These compounds were evaluated for their antibacterial activity and exhibited potent antibacterial activity against all of the drug resistant strains tested with MICs comparable to those of marketed drugs such as vancomycin and linezolid.

#### **Gas Chromatography and Mass Spectrometry Analyses of Volatiles from Marine Algae** 

JerkovićI.^1^MarijanovićZ.^1^KranjacM.^1^JokićS.^2^RojeM.^3^1Faculty of Chemistry and Technology, University of Split, R. Boškovića 35, Split 21000, Croatia2Josip Juraj Strossmayer University of Osijek, Faculty of Food Technology Osijek, Franje Kuhača 20, Osijek 31000, Croatia3Institure Rudjer Boskovic, Bijenicka Cesta 54, Zagreb 10000, Croatia

Marine secondary metabolites possess outstanding structural and functional diversity related to their different metabolic pathways. While the volatile organic compounds (VOCs) of terrestrial plants have attracted attention for a long time, the VOCs of marine algae have been much less investigated. The most common VOCs released by terrestrial plants are: “green leaf volatiles” (GLVs) which consist almost exclusively of C6 aldehydes and alcohols, other aliphatic compounds, monoterpenes, sesquiterpenes, phenylpropane derivatives, others. This is in remarkable contrast to marine VOCs that show much higher structural diversity: e.g., only among marine sesquiterpenes 54 different skeletal types of tricyclic sesquiterpenes were found. The best method for the VOCs analysis is gas chromatography coupled to mass spectrometry (GC-MS). However, before the analysis adequate preparative methods should be applied to marine algae samples such as headspace solid-phase microextraction (HS-SPME) or hydrodistillation (HD) that can be applied on the fresh or dried samples. Different examples of marine algae VOCs chemical profiles will be presented containing terpene compounds (mostly sesquiterpenes), sulphur containing compounds, non-isoprenoid C_11_-compounds, others. VOCs of marine organisms can have multiple functions in ecosystem in intraspecific (pheromones) and interspecific (kairomones) communication and in activated defences. In addition, these compounds (usually present in the extracts) may exhibit other biological activities, e.g., antibacterial, cytotoxic and antitumor.

#### **Bromophenolics from the Southern Australian Marine Alga *Polysiphonia decipiens*** 

LeverJamesBrkljačaRobertUrbanSylviaMarine and Terrestrial Natural Product Research Group, School of Science (Applied Chemistry & Environmental Science), RMIT University, GPO Box 2476, Melbourne, Vic 3001, Australia

**Figure 30:**
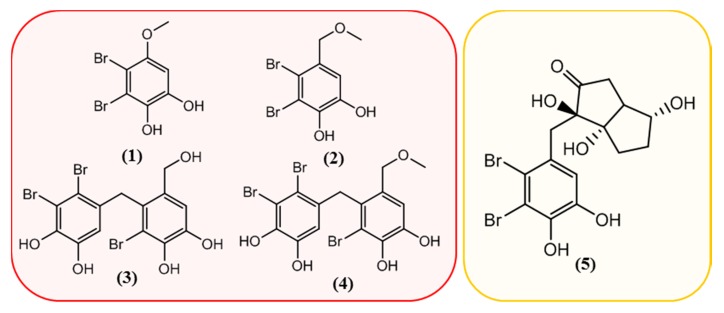
Marine natural products isolated from *P. decipiens*. Previously reported natural products (**red**) and determination of the relative configuration (**yellow**).

Marine red algae of the family Rhodomelaceae are known to produce bromophenolic type secondary metabolites that display antifeedant, antioxidant and anti-inflammatory properties [1–3]. Reported herein is the extraction, isolation and characterisation of four previously reported bromophenolic compounds (**1**–**4**) (Figure 30), from the red alga *Polysiphonia decipiens*, all of which displayed no appreciable antibacterial properties. Also reported is the isolation, characterisation and derivatisation of the known natural product rhodomelol (**5**) which was previously isolated from other members of the family Rhodomelaceae *Polysiphonia* lanosa and *Osmundaria colensoi.* Detailed here is the confirmation of the relative configuration of rhodomelol (**5**) which has been achieved for the first time. This involved chemical derivatisation followed by conducting single irradiation NOE NMR spectroscopy experiments. Finally, we were also able to secure the structure of an unprecedented structural derivative of rhodomelol via extensive 1D and 2D NMR spectroscopy. This work represents the first study of the phytochemical constituents of *P. decipiens*.

References

1. Kurata, K.K.; Taniguchii, K.; Takashima, K.; Hayashi, I.; Suzuki, M. Feeding deterrent bromophenols from *odonthalia corymbifera*. *Phytochemistry*
**1997**, *45*, 485–487.
2. Li, K.; Li, X.M.; Ji, N.Y.; Wang, B.G. Bromophenols from the Marine Red Alga *Polysiphonia urceolata* with DPPH Radiacal Scavenging Activity. *J. Nat. Prod.*
**2008**, *71*, 28–30.
3. Choi, Y.K.; Ye, B.-R.; kim, E.-A.; Kim, J.; Kim, M.-S.; Lee, W.W.; Ahn, G.-N.; Kang, N.; Jung, W.-K.; Heo, S.-J. Bis (3-bromo-4,5-dihydroxybenzyl) ether, a novel bromophenol from the marine red alga *Polysiphonia morrowii* that suppresses LPS-induced inflammatory response by inhibiting ROS-mediated ERK signaling pathway in RAW 264.7 macrophages. *Biomed. Pharmacother.*
**2018**, *103*, 1170–1177.

#### **Discovery of Two New Sorbicillinoids by Overexpression of the Global Regulator Laea in *Penicillium dipodomyis* YJ-11** 

YuJing^1^ZhangXianyan^1^MaChuanteng^1^SunChunxiao^1^CheQian^1^GuQianqun^1^ZhuTianjiao^1^,^2^ZhangGuojian^1^,^2^LiDehai^1^,^2^1Key Laboratory of Marine Drugs, Chinese Ministry of Education, School of Medicine and Pharmacy, Ocean University of China, Qingdao 266003, China2Laboratory for Marine Drugs and Bioproducts of Qingdao Pilot National Laboratory for Marine Science and Technology, Qingdao 266237, China

For decades, various secondary metabolites produced by filamentous fungi continued to be heard since they are important sources of bioactive natural products. As the sequencing technology developed, a great many biosynthetic genes were found not expressed under the ordinary laboratorial conditions, which were also referred to as “silent” or “cryptic” genes. To up-regulate these gene clusters in order to mine new compounds, a variety of approaches have been developed to affect the biosynthetic process. As a part of our ongoing work of searching diversified secondary metabolites from marine derived fungi, we recently isolated one filamentous fungi *Penicillium dipodomyis* YJ-11 from a marine sediment sample collected in Jiaozhou Bay of Qingdao. In order to activate the silent genes and obtain novel secondary metabolites, we overexpressed the native global regulator PdLaeA in *P. dipodomyis* YJ-11 and the mutant showed obvious changes both in morphologies and metabolic products in contrast to the wild type strain. Further chemical studies on mutant strain led to the isolation of two new compounds together with four known sorbicillin analogues. This is the first report on application of global regulator LaeA in *P. dipodomyis* with the purpose to tap up the silent genes and the result supported the truth that overexpression of the global regulator LaeA could be a feasible tool in activating silent biosynthetic gene clusters and producing new natural product structures.

#### **Nucleosides from the Pacific Bryozoan *Nelliella nelliiformis*** 

BracegirdleJoe^1^,^2^GordonDennis P^3^HarveyJoanne E.^1^,^2^KeyzersRobert A.^1^,^2^1School of Chemical and Physical Sciences, Victoria University of Wellington, 6012 Wellington, New Zealand2Centre for Biodiscovery, Victoria University of Wellington, 6012 Wellington, New Zealand3National Institute of Water & Atmospheric Research (NIWA), P.O. Box 893, Nelson, New Zealand

Marine organisms are a valuable source of bioactive natural products. Bryozoans, or sea mosses, are an understudied class of marine invertebrate, yet still have been the source of a variety of medicinally relevant compounds, such as the well-known bryostatins. Here, the secondary metabolites of the Pacific bryozoan *Nelliella nelliiformis* were investigated using an NMR-guided isolation procedure, leading to the purification of two new nucleosides termed nelliellosides A and B. The structures, including absolute configurations, were solved by spectroscopic and chromatographic techniques. Their total synthesis, along with a panel of four similar analogues, was also achieved, and a biological evaluation is reported herein.

**Figure:**
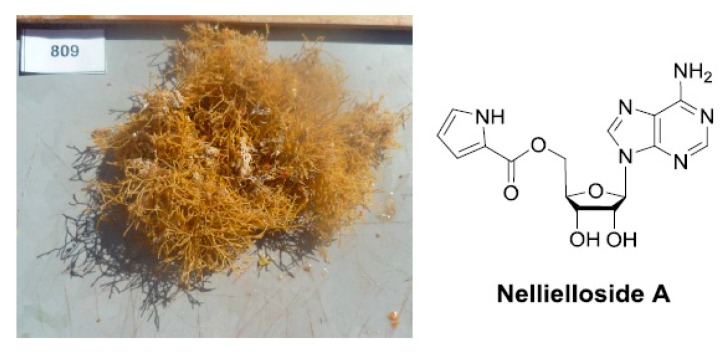
**Graphical Abstract**

#### **Discovery of New Anti-Inflammatory Cembranoids from a Formosan Soft Coral *Sarcophyton cherbonnieri*** 

PengChia-Chi^1^HuangChiung-Yao^1^AhmedAtallah F.^2^HwangTsong-Long^3^SheuJyh-Horng^1^,^4^1Department of Marine Biotechnology and Resources, National Sun Yat-sen University, Kaohsiung 804, Taiwan2Department of Pharmacognosy, College of Pharmacy, King Saud University, Riyadh 11451, Saudi Arabia3Graduate Institute of Natural Products, College of Medicine, Chang Gung University, Taoyuan 333, Taiwan4Frontier Center for Ocean Science and Technology, National Sun Yat-sen University, Kaohsiung 804, Taiwan

**Figure 31:**
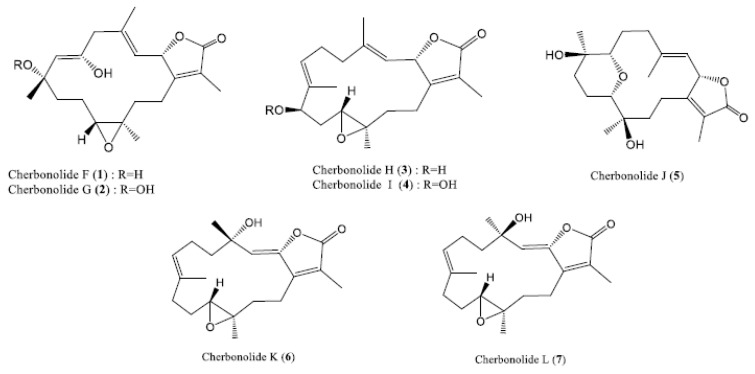
Chemical structures of cherbonolides F–L (**1**–**7**).

We have previously discovered seven cembranoids including an anti-inflammatory biscembrane peroxide from Formosan soft coral *Sarcophyton cherbonnieri* [1]. In order to detailedly investigate the secondary metabolites for understanding the biosynthetic capability and potential bioactivity of natural products from *S. cherbonnieri*, we continued our chemical investigation of this marine organism. This study led to the isolation of seven natural compounds, cherbonolides F–L (**1**–**7**) (Figure 31). The structures and relative configurations of these metabolites had been established by detailed spectroscopic analyses including 1D and 2D NMR (COSY, HSQC, HMBC and NOESY) data associated with MS experiments. The anti-inflammatory activities of compounds (**1**–**7**) on neutrophil pro-inflammatory responses were assayed by measuring their ability in suppressing fMLF/CB-induced superoxide anion generation and elastase release in human neutrophils. From the results, cherbonolide G (**2**) significantly exhibited elastase release (48.2 ± 6.2%) and cherbonolide H (**3**) showed stronger inhibition (44.5 ± 4.6%) toward superoxide anion generation at 30 µM, than other metabolites. 

References

1. Peng, C.-C.; Huang, C.-Y.; Ahmed, A.F.; Hwang, T.-L.; Dai, C.-F.; Sheu, J.-H. New Cembranoids and a Biscembranoid Peroxide from the Soft Coral Sarcophyton cherbonnieri. *Mar. Drugs*
**2018**, *16*, 276.

#### **Retrospective Metabolomics Using GNPS Molecular Networking for the Identification and Isolation of Bioactive Secondary Metabolites from Extreme Environment Microorganisms** 

MirandaKevin Jace A.^1^HamedAhmed A.^2^EbelRainer^1^JasparsMarcel^1^1Marine Biodiscovery Centre, Department of Chemistry, University of Aberdeen, Meston Building, Meston Walk, Aberdeen AB24 3UE, Scotland, UK2Microbial Chemistry Department, National Research Centre, 33 El-Buhouth Street, P.O. Box 12622, Dokki, Giza, Egypt

**Figure 32:**
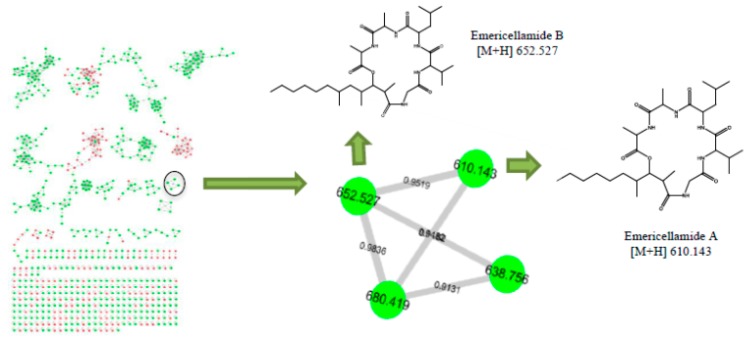
Molecular Network of the crude extract of *Aspergillus* sp. M113 revealed clusters of cyclic depsipeptides Emericellamide A and B and its potential new analogs.

The evolution of natural products research and drug discovery from bioassay guided isolation to genomic and statistical approaches like metabolomics has paved the way to the isolation and characterization of new and novel compounds. One such statistical approach that has gained increasing attention is Global Natural Product Social Molecular Networking (GNPS) molecular networking, relying on MS/MS fragmentation to evaluate similarities and differences in complex data sets. In this study, we employed what we term retrospective metabolomics to evaluate the secondary metabolites produced by microorganisms living in two different extreme environments. We analyzed the isolated compounds from two strains of bacteria, *Halomonas sulfadaestris* and *Pseudomonas* sp. from the South Shetland Trench, Antarctica and two strains of fungi, *Aspergillus* sp. M113 and *Penicillium* sp. 7S9 from Hurghada, Egypt. High resolution electrospray ionisation mass spectrometry data from each sample was enterered into GNPS, along with its internal database, to construct molecular networks evaluating known and unknown compounds. Prior to molecular networking, we isolated a cyclic depsipeptides such as Emericellamide A from the fungal sample and bacillomycin from the bacterial sample. Emericellamide A showed a distinct cluster in the molecular network that suggests several new analogs are present based on dereplication and MS/MS fragmentation patterns. These clusters identified from the molecular network will serve as a guide for further fractionation, isolation and structure elucidation. (Figure 32)

#### **Isolation and Antimycobacterial Activity of New Secondary Metabolites from a South African Marine *Streptomyces* Species MUIZ4Y** 

AcquahKojo S.^1^BeukesDenzil^2^WarnerDigby F.^3^,^4^,^5^MeyersPaul R.^6^SunasseeSuthananda N.^1^EbelRainer^7^DengHai^7^JasparsMarcel^7^GammonDavid W.^1^1Department of Chemistry, University of Cape Town, Rondebosch 7701, South Africa2School of Pharmacy, University of the Western Cape, Bellville 7535, South Africa3SAMRC/NHLS/UCT Molecular Mycobacteriology Research Unit & DST/NRF Centre of Excellence for Biomedical TB Research, Department of Pathology, Faculty of Health Sciences, University of Cape Town, Observatory 7925, South Africa4Institute of Infectious Disease and Molecular Medicine, Faculty of Health Sciences, University of Cape Town, Observatory 7925, South Africa5Wellcome Centre for Clinical Infectious Diseases Research in Africa, University of Cape Town, Rondebosch 7701, South Africa6Department of Molecular and Cell Biology, University of Cape Town, Rondebosch 7701, South Africa7Marine Biodiscovery Centre, Department of Chemistry, University of Aberdeen, Old Aberdeen AB24 3UE, Scotland, UK

**Figure 33:**
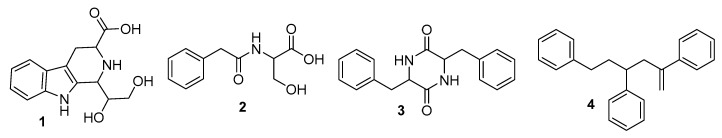
Compounds Isolated from Streptomyces species Muiz4Y.

Tuberculosis (TB) is the leading cause globally of mortality owing to an infectious disease. The rapid emergence of multidrug-resistant strains of the causative agent, Mycobacterium tuberculosis, coupled with the HIV-AIDS co-pandemic, necessitates the urgent discovery of potent anti-TB drugs with novel mechanisms of action. Natural products have historically proved a major source of drugs with microorganisms including actinobacteria serving as prolific producers of bioactive molecules. Here, we report the isolation of a *Streptomyces* strain Muiz4Y, from marine sediment collected along the False Bay coastline between Muizenberg and St. James in Cape Town, South Africa. The organic extract of a liquid culture of this strain exhibited antimycobacterial activity against *Mycobacterium aurum* A+ and *Mycobacterium tuberculosis* H37Rv. An activity guided isolation scheme including sequence of column chromatography, preparative TLC and reverse phase HPLC resulted in the isolation of four compounds comprising a β-carboline alkaloid (**1**), peptide (**2**) (Figure 33), diketopiperazine (**3**) and triphenyl hexane (**4**). Although **3** and **4** are known compounds **1** and **2** are new natural products. Only **4** exhibited antimycobacterial activity against *Mycobacterium tuberculosis* H37Rv with an MIC of 5.764 µg/mL. 

#### **Exploring Marine *Micromonospora* Species as Phocoenamicins Producers** 

KokkiniMariaPerez-BonillaMercedesOves-CostalesDanielde la CruzMercedesMartinJesúsVicenteFranciscaGenilloudOlgaReyesFernandoFundación MEDINA, Avda. del Conocimiento 34, Parque Tecnológico de Ciencias de la Salud, 18016 Granada, Spain

Marine actinomycetes are considered as a drug treasure house with respect to great potential regarding their secondary metabolites. Within the “rare actinomycetes” group, *Micromonospora* is the most prolific in producing metabolites, accounting for more than 700 compounds to date, including pharmaceutically important chemical classes [1]. Spirotetronates are a growing family of actinomyces-derived polyketides that possess complex structures and exhibit potent and unexplored bioactivities [2]. As part of the PharmaSea and MarPipe projects, phocoenamicins B and C, together with the known spirotetronate polyketide phocoenamicin, were isolated from cultures of *Micromonospora* sp. and demonstrated antimicrobial activity against methicillin-resistant *Staphylococcus aureus* (MRSA) and *Mycobacterium tuberculosis* H37Ra [3]. In this work, we cultivated *Micromonospora* sp. using an OSMAC approach, selected the most promising fermentation media to scale up their production and detected that EtOAc extracts produce several structurally related compounds not disclosed before, depending on the culture medium. Herein we report the diverse production, isolation and structural elucidation of new additional phocoenamicins isolated from culture broths of this actinomycete. Three strains belonging to the *Micromonospora* genus (CA-214671, CA-218877 and CA-214658), isolated from marine invertebrate and sediments collected in the Canary Islands were grown on carefully selected fermentation media (FR23-M, RAM2-P V2 and M-016). The Liquid-Liquid extraction of culture broths with EtOAc afforded organic crude extracts which were subjected to reversed-phase preparative HPLC and yielded several new minor analogs of the previously mentioned spirotetronates. Structural elucidation of the compounds was based on 1D and 2D NMR and High Resolution Mass Spectrometry (ESI-TOF). 

References

1. Igarashi, Y.; Ogura, H.; Furihata, K.; Oku, N.; Indananda, C.; Thamchaipenet, A. Maklamicin, an antibacterial polyketide from an endophytic *Micromonospora* sp. *J. Nat. Prod.*
**2011**, *74*, 670–674.
2. Braddock, A.A.; Theodorakis, E.A. Marine Spirotetronates: Biosynthetic Edifices That Inspire Drug Discovery. *Mar. Drugs*
**2019**, *17*, 232.
3. Pérez-Bonilla, M.; Oves-Costales, D.; de la Cruz, M.; Kokkini, M.; Martín, J.; Vicente, F.; Genilloud, O.; Reyes, F. Phocoenamicins B and, C.; New Antibacterial Spirotetronates Isolated from a Marine *Micromonospora* sp. *Mar. Drugs*
**2018**, *16*, 95.

#### **Isolation and Structure Elucidation of Ficiformylenes Isolated from the Italian Marine Sponges of *Petrosia ficiformis*** 

OyadomariYasumoto^1^MachidaKoshi^2^BeckingLeontine E.^3^,^4^BavestrelloGiorgio^5^D’AuriaMaria Valeria^6^ZampellaAngela^7^FusetaniNobuhiro^8^NakaoYoichi^1^,^2^1Graduate School of Advanced Science and Engineering, Waseda University, Shinjuku-ku, Tokyo 169-8555, Japan2Research Institute for Science and Engineering, Waseda University, Shinjuku-ku, Tokyo 169-8555, Japan3Wageningen Marine Research, Wageningen University & Research Centre, 1781 AG Den Helder, The Netherlands4Marine Animal Ecology, Wageningen University & Research Centre, Wageningen, The Netherlands5DiSTAV (Department of Earth, Environmental and Life Sciences), University of Genoa, Corso Europa 26, 16132 Genova, Italy6Department of Pharmacy, University of Naples “Federico II”, 1-80131 Naples, Italy7Department of Pharmacy, University of Naples “Federico II”, Via D. Montesano 49, 80131 Naples, Italy8Fisheries and Oceans Hakodate, Hakodate, Hokkaido 041-8611, Japan

Marine sponges are a rich source of secondary metabolites containing unique structures and bioactivities. Sponges are very fertile host animals for diverse symbiotic microorganisms which are believed to produce most of the sponge secondary metabolites. However, there are only a limited number of studies on the difference in sponge metabolites from the different habitats. The marine sponges of the genus *Petrosia* have been reported from various parts in the world and are known to contain a variety of polyacetylene compounds. During our study to compare sponge secondary metabolites of the same species, we found that the extracts of three marine sponge specimens of *Petrosia ficiformis* collected at the different sites in Italy exhibited different compositions of the secondary metabolites. As the result of the LC/MS-guided fractionation of the extracts prepared from *P. ficiformis* collected at Paraggi and Ischia, a new polyacetylene, ficiformylene A (**1**), was isolated as the characteristic compound of the collection at Paraggi. The pseudomolecular ion peak of **1** was detected only in the extract of *P. ficiformis* collected at Paraggi, while the related ficiformylene B (**2**) was detected in all the extracts of *P*. *ficiformis* collections. The structures of ficiformylens were elucidated on the basis of NMR and MS analyses. Ficiformylene A (**1**) indicated cytotoxicity against P388 cells with an IC_50_ value of 11.2 μM, while ficiformylene B (**2**) exhibited no cytotoxicity.

#### **The Chemical Constituents and Biological Activities of *Cladosiphon okamuranus*** 

ChengKun-Ching^1^,^2^WuTian-Shung^3^1Taiwan Sugar Research Institute, Tainan 701, Taiwan2Department of Chemistry, National Cheng Kung University, Tainan 701, Taiwan3School of Pharmacy, National Cheng Kung University, Tainan 701, Taiwan

*C. okamuranus* is a filamentous brown alga that mainly grows in Okinawa, Japan and the sub-tropical reef area of the Ryukyu islands. Recently, many experiments have confirmed that since *C. okamuranus* is rich in bio-active substances such as alginic acid and sulfated polysaccharides (fucoidan). They have a wide spectrum of activity in biological systems. Besides their well-known anticoagulant, anti-tumor, anti-hyperlipidemia and anti-thrombotic activity, fucoidans modulate inflammation, possess anti-proliferative and anti-adhesive effects on cells, protect cells from viral infection, and interfere with mammalian fertilization. *C. okamuranus* are now used as raw material for development of drugs and are also widely used as a health-promoting food component. In the present study, we aimed to purify anti-inflammatory consitiuents from *C. okamuranus* and further to develop anti-inflammatory food supplement.

#### **Isolation and Absolute Configuration of the Cyanobacterial Secondary Metabolite Nocuolin A** 

PretoMarco A. C.^1^SousaM. Lígia^1^,^2^UrbatzkaRalph^1^AlmeidaJoana R.^1^LeãoPedro N.^1^VasconcelosVitor^1^,^2^1CIIMAR—Interdisciplinary Centre of Marine and Environmental Research, Terminal de Cruzeiros de Leixões, Av. General Norton de Matos s/n, 4450-208 Matosinhos, Portugal2FCUP—Faculty of Sciences, University of Porto, Rua do Campo Alegre 1021/1055, 4169-007 Porto, Portugal

Cyanobacteria are ancient organisms that have been evolving surviving strategies for more than 3500 million of years. These diverse survival strategies translate into chemical diversity, originating compounds with wide variety of activities and very diverse chemical templates. As such, cyanobacteria have been proving to be a valuable resource for new chemical entities, with unique structural features. A recently discovered secondary metabolite, nocuolin A [1], was found to also be produced by a strain from LEGE-CC collection [2]. In this work, the isolation of nocuolin A was carried out using a bioassay guided procedure. The planar structure of the isolated metabolite was confirmed via 1D and 2D NMR and LC-MS/MS experiments. The determination of the absolute configuration of nocuolin A’s single stereogenic centre was performed by using ECD spectroscopy coupled with computational simulations. In addition, clues regarding the intriguing biosynthesis of this compound were collected from feeding experiments employing ^13^C labelled sodium acetate. The biological activity of nocuolin A has also been explored [3].

##### **Acknowledgments** 

Support for this work was provided by the Strategic Funding UID/Multi/04423/2013 through national funds provided by the Foundation for Science and Technology (FCT) and the European Regional Development Fund (ERDF), in the framework of the programme PT2020 and funding was provided by the project NASCEM PTDC/BTA-BTA/31422/2017 (POCI-01-0145-FEDER-031422), financed by the FCT, COMPETE2020 and PORTUGAL2020.

References

1. Voráčová, K.; Hájek, J.; Mareš, J.; Urajová, P.; Kuzma, M.; Cheel, J.; Villunger, A.; Villunger, A.; Kapuscik, A.; Bally, M.; et al. The cyanobacterial metabolite nocuolin a is a natural oxadiazine that triggers apoptosis in human cancer cells. *PLoS ONE*
**2017**, *12*, e0172850, doi:10.1371/journal.pone.0172850.
2. Ramos, V.; Morais, J.; Castelo-Branco, R.; Pinheiro, Â.; Martins, J.; Regueiras, A.; Pereira, A.L.; Lopes, V.R.; Frazão, B.; Gomes, D.; et al. Cyanobacterial diversity held in microbial biological resource centers as a biotechnological asset: the case study of the newly established LEGE culture colection. *J. Appl. Phycol.*
**2018**, doi:10.1007/s10811-017-1369-y.
3. Sousa, M.L.; Preto, M.; Vasconcelos, V.; Linder, S.; Urbatzka, R. Antiproliferative Effects of the Natural Oxadiazine Nocuolin A Are Associated With Impairment of Mitochondrial Oxidative Phosphorylation*. Front. Oncol.*
**2019**, *9*, doi:10.3389/fonc.2019.00224.

#### ***Stylissa* aff. Carteri from Futuna Islands as a Source of Pyrrole-Imidazole Alkaloids** 

Miguel-GordoMaria^1^CalabroKevin^1^JenningsLaurence^1^MorrowChristine^2^ThomasOlivier P.^1^1Marine Biodiscovery, School of Chemistry and Ryan Institute, National University of Ireland Galway (NUI Galway), University Road, H91 TK33 Galway, Ireland2Department of Natural Sciences, National Museums Northern Ireland, 153 Bangor Road, BT18 0EU Cultra, Northern Ireland

**Figure 34:**
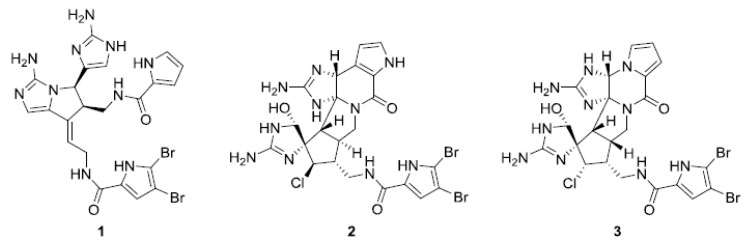
Structures of new PIAs from the sponge Stylissa aff. carteri.

The Tara Pacific expedition (2016–2018) explored the Pacific Ocean with the aim of conducting an integrated study of the marine biodiversity of coral reefs facing climate and demographic changes. In this context, an inventory of marine sponges was undertaken for the first time around the Futuna Islands situated in the Central Indo-Pacific Ocean with the objective of describing their associated chemical diversity. A priority list of the collected sponges was created based on the taxonomy, biomass available, chemical profiles and bioassay results. The sponge *Stylissa* aff. *carteri* was selected for further investigation leading to the isolation of 8 brominated pyrrole-imidazole alkaloids (PIAs). Three new oroidin derivatives were identified, a new bicyclic dimer with a novel pyrrolo[1,2-*c*]imidazole core (**1**) and two new hexacyclic dimers, a dibrominated carteramine (**2**) and a dibrominated konbu’acidin (**3**) analogues (Figure 34). The 2D structures of **1**–**3** were elucidated from extensive analysis of NMR and HRMS spectroscopic data. The absolute configuration of the new molecules was assigned through computational and ECD experiments. Further, investigation through molecular networking applied to the fractions unveiled a large diversity of analogues.

#### **Antiproliferative and Anti-inflammatory Potential of Isolated Compounds from Octopus Ink (*Octopus vulgaris*)** 

Hernandez-ZazuetaMartin Samuel^1^Carbonell-BarrachinaÁngel Antonio^2^Taboada-AnteloPablo^3^Rosas-BurgosEma Carina^1^Ezquerra-BauerJosafat Marina^1^Garcia-RomoJoel Said^1^Campos-VegaRocío^4^Sandoval-PetrisEdgar^5^Burboa-ZazuetaMaría Guadalupe^5^Hernández-MartínezJavier^6^Burgos-HernándezArmando^1^1Department of Research and Postgraduate in Food, University of Sonora, Sonora 83000, Mexico2Department of Agrifood Technology, University of Miguel Hernandez de Elche, 03202 Alicante, Spain3Department of Particle Physics, University of Santiago de Compostela, 15703 Santiago de Compostela, Spain4Department of Chemistry, The Autonomous University of Querétaro. Santiago de Querétaro, Querétaro 76010, Mexico5Department of Scientific and Technological Research, University of Sonora, Sonora 83000, Mexico6Support Services Unit in Analytical Resolution, Universidad Veracruzana, Veracruz 91190, Mexico

The purpose of this study was to take advantage of the by-products of fishing, where compounds have been identified as a product of the secondary metabolism of marine organisms with potential pharmacological application, especially against chronic degenerative diseases such as cancer. In turn, persistent chronic inflammation processes have been linked to carcinogenesis and the development of the disease. Therefore, we used freeze-dried octopus ink (*Octopus vulgaris*) for the search, isolation, and characterization of the main compounds present in the ink, with anti-inflammatory and anti-proliferative potential. The results exhibited a compounds present in fraction eluted with Hex/AcOEt (open column) with IC_50_ of 47 μg/mL on nitric oxide production in stimulated macrophage cells with LPS from *E. coli*; also, the evaluation of inflammatory proteins modulation (Mouse Cytokine Array Panel A, of Proteome Profiler, Antibody Array, R&D Systems), suggest a regulation of different cytokine implicated in the pro-inflammatory response (IL-2, IL-7, IL-16, IL-23 and CCL-17). It should be noted that, this interaction with membrane proteins (Il-23 receptors) is linked to was observed (on the basis of microscopic immunofluorescence) when colon (GI_50_ of 52 μg/mL) and prostate (GI_50_ of 27 μg/mL) cancer cells were treated with the same compounds, exhibiting morphological membrane changes and apoptotic bodies, suggesting death by apoptosis. On the other hand, ^1^H and ^13^C NMR analysis suggested the presence of different sterol base, pyrrole, and indole compounds. Consequently, it is proposed to investigate the mechanism of action for the use of this type of compounds as possible drugs in the treatment of this disease.

#### **Bacterial-Derived Extracellular Electron Shuttle** 

MeversEmily^1^SuLin^2^PishchanyGleb^1^,^3^CornejoJose^2^BaruchMoshe^2^Ajo-FranklinCaroline^2^ClardyJon^1^1Department of Biological Chemistry & Molecular Pharmacology, Harvard Medical School, Boston, MA 02115, USA2Molecular Foundry, Lawrence Berkeley National Laboratory, Berkeley, CA 94720, USA3Department of Microbiology and Immunobiology, Harvard Medical School, Boston, MA 02115, USA

*Shewanella oneidensis* MR-1 is a facultative anaerobic γ-proteobacterium that has the ability to utilize a diverse suite of terminal electron acceptors, including insoluble solid metal oxides. The mechanisms underlying how MR-1 indirectly shuttles electrons to these solid substrates are poorly understood. In 2000 chemical analyses of MR-1’s spent supernatant revealed that MR-1 excretes a small labile molecule that has the ability to recover anaerobic respiration of mutants on solid substrates, but the active metabolite was never identified. Revisiting this lack of identity with specialized resin, HR-LCMS analysis, and total synthesis, led to its identification as 2-amino-3-carboxy-naphthoquinone (ACNQ). ACNQ is derived from DHNA (1,4-dihydroxy-2-naphthoic acid) in a non-enzymatic process that frustrated genetic approaches to identify the shuttle. Both ACNQ and DHNA robustly restore reduction of AQDS under anaerobic growth in menaquinone-deficient mutants. Electrochemistry analyses reveal that ACNQ is contributing to the extracellular electron transfer (EET) as an electron shuttle, without altering any menaquinone generation or EET related cytochrome *c* expression.

#### **Anti-Bacterial Biofilm Activity and Fatty Acids Composition from Saudi Red Sea Organisms** 

HawasUsama W.ShaherFekriGhandourahMohamedEl-KassemLamia T. AbouSatheeshSathianesonAl-SofyaniAbdul Mohsin A.Marine Chemistry Department, Faculty of Marine Sciences, King Abdulaziz University, Jeddah 21589, Saudi Arabia

Biofilm formation is the primary step in biofouling growth on substrates submerged in the sea. This study aimed to evaluate the antibiofilm activity of metabolites of some Red Sea organisms against three biofilm-bacterial strains isolated from Jeddah coast. Free fatty acids (FAs) and other lipoidal matters were extracted from the sea cucumber *Holothuria atra*, green alga *Avrainvillea amadelpha*, and costal plant *Salicornia fruticosa*. The composition of lipoidal fractions were analyzed by GC-MS which showed *A. amadelpha* is rich by saturated FAs with 74%, while sea cucumber *H. atra* revealed high content of unsaturated FAs with 60%. Palmetic acid is the major FAs composition in all species ranged from 14.5% to 26.7%. Phytol, sterols and hydrocarbons (C8–C29) were represented in the alga *A. amadelpha* as high contents with values 25.8%, 21.9% and 18.5%, respectively. The metabolites of extracts and lipoidal contents showed biofilm inhibitory activity against the isolated bacterial strains, where the lipoidal (unsaponified) fraction of *S. fruticosa* exhibited higher inhibitory activity against *Planomicrobium* sp. at a concentration of 200 μg/mL. Concentration dependent activity was not uniform among the extracts or the fractions.

#### **Polyoxygenated Steroids from the Soft Coral *Sinularia polydactyla* Collected in the Egyptian Red Sea** 

TammamMohamed^1^,^2^MahdyAldoushy^3^IoannouEfstathia^1^RoussisVassilios^1^1Section of Pharmacognosy and Chemistry of Natural Products, Department of Pharmacy, National and Kapodistrian University of Athens, 15771 Athens, Greece2Biochemistry Department, Faculty of Agriculture, Fayoum University, Fayoum 63514, Egypt3Department of Zoology, Faculty of Science, Al-Azhar University (Assiut Branch), Assiut 71524, Egypt

Steroidal molecules comprise a very significant class of natural product owing to their capability to cross the lipophilic membrane of the cell, and after binding to the steroid receptors, articulate their detailed physiological functions. Soft corals are considered a prolific source of often structurally complex steroids, showing diversified nuclei or functionalized side chains. Steroids of marine origin and especially polyoxygenated sterols have exhibited a diverse array of pharmacological activities, such as antimicrobial, cytotoxic, antifouling, ichthyotoxic and antiinflammatory. In the course of our investigations on the chemistry of marine organisms, specimens of the gorgonian *Sinularia polydactyla* were collected by SCUBA diving from Hurghada in the Red Sea (Egypt). The soft coral tissues were extracted with mixtures of CH_2_Cl_2_/MeOH and the organic residue was submitted to a series of chromatographic separations, leading to the isolation of 24 steroids, among which 6 are new natural products. The structures and the relative configurations of the isolated natural products were established mainly on the basis of extensive analysis of 1D and 2D NMR and MS data.

##### **Acknowledgments** 

Financial support to M.T. from the non-profit organization.

#### **A Novel Chlorophyll Derivative, 13^2^-Hydroxy-Pheofarnesin A, from a Marine Cyanobacteria with Lipid Reducing Activity** 

SilvaNatália Gonçalves^1^SousaMaria Lígia^1^ReisMariana Alves^1^VasconcelosVítor^1^,^2^UrbatzkaRalph^1^1Interdisciplinary Center of Marine and Environmental Research (CIIMAR/CIMAR), University of Porto, Terminal de Cruzeiros de Leixões, Av. General Norton de Matos s/n, 4450-208 Matosinhos, Portugal2Faculty of Sciences, University of Porto, Rua do Campo Alegre 1021/1055, 4169-007 Porto, Portugal

Research has focused on natural product discovery for health treatments, such as obesity and related co-morbidities, and cyanobacteria are regarded as a prolific source of biologically active natural compounds. In this work, a bioassay-guided isolation approach was used to discover secondary metabolites from the Blue Biotechnology and Ecotoxicology Culture Collection (LEGEcc) with lipid reducing activity. Cyanobacterial strain *Nodosilinea* sp. LEGE 06001 was cultured and harvested. The freeze-dried biomass was extracted by repeated percolation with mixtures of DCM/MeOH. The resultant organic crude extract was fractionated by normal-phase vacuum liquid chromatography, and after several rounds of fractionation a novel chlorophyll derivative was successfully isolated. The structure elucidation of 13^2^-hydroxy-pheofarnesin a (hfa) was established based on one- and two-dimensional NMR spectroscopy and mass spectrometry. Hfa showed significant neutral lipid-reducing activity in the zebrafish Nile red fat metabolism assay after 48 h of exposure with an EC_50_ value of 15.5 ± 1.3 μM and did not cause any general toxicity or malformations. In an attempt to establish the structure-activity relationships, chlorophylls a and b were analysed using the same assay. Since did not show any effect, these results highlight the importance of the structural differences of hfa for the lipid reducing activity. This bioactivity was additionally confirmed in differentiated 3T3-L1 multicellular spheroids of murine preadipocytes. Hfa may be developed as a nutraceutical with lipid reduction activity, however future studies are needed to clarify its mechanisms of action.

##### **Acknowledgments** 

This work was supported by the European ERA-NET Marine Biotechnology project CYANOBESITY (ERA-MBT/0001/2015), financed by national funds through FCT (Foundation for Science and Technology, Portugal).

#### **A National Marine Biodiscovery Laboratory in Ireland** 

KaurNavdeep^1^,^2^JenningsLaurence K.^1^,^2^RodriguesDaniel^1^,^2^FisherJeffrey^2^FitzGeraldDick^3^DobsonAlan^4^StengelDagmar B.^5^ZanollaMarianela^5^McCormackGrace^6^ThomasOlivier P.^1^1Marine Biodiscovery, School of Chemistry and Ryan Institute, National University of Ireland Galway (NUI Galway), University Road, H91 TK33 Co. Galway, Ireland2Marine Biodiscovery, Marine Institute, Rinville West, H91 R673 Co. Galway, Ireland3Department of Biological Sciences, Faculty of Science & Engineering, University of Limerick, V94 T9PX Co. Limerick, Ireland4Environmental Research Institute, University College Cork, College Road, T12 K8AF Co. Cork, Ireland5Botany and Plant Science, School of Natural Sciences and Ryan Institute, National University of Ireland Galway (NUI Galway), University Road, H91 TK33 Co. Galway, Ireland6Zoology, School of Natural Sciences and Ryan Institute, National University of Ireland Galway (NUI Galway), University Road, H91 TK33 Co. Galway, Ireland

The marine environment is a source of an underexplored diversity of macro and microorganisms which are known to produce unique specialized metabolites for communication, defence, and competition. Some marine ecoregions of the oceans have not been inventoried and only sparsely chemically studied like the coasts of the island of Ireland in the North Eastern Atlantic Ocean.

The NMBLI aims to promote marine biodiscovery in Ireland and to bring Ireland to the forefront of global marine research in Europe. Therefore, the NMBLI has developed an Irish marine repository containing freeze-dried biomass, voucher specimens, and fractionated extracts from Irish marine invertebrates. Our repository is organized around a state-of-the-art web-database for storage of data and dereplication of known natural products. The fractionated extracts are chemically screened with UHPLC-HRMS/MS and biologically screened against a broad panel of microbial pathogens and tumour cell lines. These data profiles are uploaded on our web-database and our linked study on the open-access EMBL-EBI’s ‘Metabolights’. This process quickly leads us to the identification of marine organisms for an in-depth chemical investigation. Herein, we present the first results from the dereplication of marine natural products from our marine repository as well as some in-depth chemical investigations that have been performed on promising Irish marine invertebrates.

#### **Insights into *Eudistoma vannamei* Holobiome: Microbial and Chemical Diversities** 

BauermeisterAnelize^1^,^2^FurtadoLuciana Costa^1^FerreiraElthon G.^3^JimenezPaula Christine^4^AraújoWelington Luiz^1^OlchnheskiLuiz Ricardo^1^da Cruz LotufoTito Monteiro^5^Costa-LotufoLeticia Veras^1^LopesNorberto Peporine^2^1Instituto de Ciências Biomédicas, Universidade de São Paulo, São Paulo 05508-000, Brazil2Departamento de Física e Química, Faculdade de Ciências Farmacêuticas de Ribeirão Preto, Universidade de São Paulo, Ribeirão Preto, São Paulo14040-903, Brazil3Departamento de Química Orgânica e Inorgânica, Universidade Federal do Ceará, CE, 60451-970 Fortaleza, Brazil4Departamento de Ciências do Mar, Universidade Federal de São Paulo, Santos, SP 11.070-100, Brazil5Instituto Oceanográfico, Universidade de São Paulo, São Paulo 05508-120, Brazil

*Eudistoma vannamei* is a colonial ascidian endemic to the Brazilian northeastern coast considered a prolific source of secondary metabolites. This ascidian also hosts bioactive compounds-producing actinomycetes. To understand the relationship between the associated-microbiota and the ascidian, herein, *E. vannamei* was studied as a holobiont model using MS-based metabolomics and 16S rRNA gene diversity analyses. Gene sequencing analysis of *E. vannamei* showed that most of the detected OTUs belong to bacterial phyla, in which Proteobacteria was the most abundant (>50%), followed by Planctomycetes, Actinobacteria, Bacteroidetes, among others. Interestingly, the three analyzed samples shared about 50% of the observed OTUs, but the *Metacoder* analysis showed that, while there is specimens’ uniqueness concerning the microbial content, especially related to Actinomycetes and Bacteroidetes, groups belonging to the core community are undoubtably more abundant. The analyses by molecular network indicated the presence of staurosporines, a well-known chemical class present in the genus *Eudistoma*, but recognized as microbial metabolites. Aminoacids and phospholipids were also observed. Bacteria were also recovered from the ascidian tissue, and cultured in liquid and solid culture media to evaluate their metabolic potential to produce different chemical structures. The protocol employed herein led to the isolation of bacteria mostly from the Actinobacteria phylum, including *Streptomyces* and *Micomonospora*. The liquid culture of these strains provided a greater number of metabolites than the solid cultures, and the molecular network analysis allowed the rapid identification of compounds from the desferrioxamine, erythromycin, and marineosin chemical classes, which are interesting compounds from a biotechnological point of view.

##### **Acknowledgments** 

Financial support from FAPESP 2015/17177-6 and2017/17648-4.

#### **First Insights into Health Issues Related to Blooms of the Cyanobacterium *Lyngbya* Cf. *majuscula* in New Caledonia** 

ThomasOlivier P.^1^SolankiHiren^1^ZubiaMayalen^2^GegundeSandra^3^CalabroKevin^1^KakueGeorges^4^AlonsoAmparo^3^BotanaLuis M.^3^PayriClaude^5^1Marine Biodiscovery, School of Chemistry and Ryan Institute, National University of Ireland (NUI Galway), University Road, H91 TK33 Galway, Ireland2UMR EIO 241, Labex CORAIL, University of French Polynesia, BP6570, 98702 Faa’a Tahiti, French Polynesia, France3Departamento de Farmacología, Facultad de Veterinaria, Universidade de Santiago de Compostela, 27002 Lugo, Spain4Direction du Développement Durable et de la Recherche Appliquée—Province des îles Loyauté, Qaneno, Drehu, New Caledonia, France5UMR ENTROPIE (IRD, UR, CNRS), Institut de Recherche pour le Développement, B.P. A5, 98848 Nouméa CEDEX, New-Caledonia, France

Cyanobacterial blooms are becoming more recurrent in the Pacific Ocean leading to health but also economic concerns among the local communities. Recently, closures of beaches have resulted from the yearly occurrence of blooms of a cyanobacterium related to *Lyngbya majuscula*. The project CYCLADES was recently supported by the Pacific Fund to give some insights into recent events of dermatitis associated with these blooms in the Drehu island of New Caledonia.

We report the first results on the chemical diversity identified in specimens overgrowing corals in the affected beach. New linear and cyclic modified peptides were isolated as major metabolites together with the known dolastatin 3. Additionally, some modified fatty acids are reported for the first time and all metabolites were biologically tested.

#### **Marine-Derived Microorganisms as Potential Sources of Neurotrophin Mimetics for the Treatment of Neurodegeneration and Neuroinflammation** 

GiaccioPaolo^1^DestroMarco^2^ShawPamela J.^2^FerraiuoloLaura^2^RoussisVassilios^1^IoannouEfstathia^1^1Section of Pharmacognosy and Chemistry of Natural Products, Department of Pharmacy, National and Kapodistrian University of Athens, 15771 Athens, Greece2Sheffield Institute for Translational Neuroscience, University of Sheffield, Sheffield S10 2HQ, UK

Among neurodegenerative diseases, Amyotrophic Lateral Sclerosis (ALS) is a rare fatal heterogeneous disorder characterized by the progressive degeneration of motoneurones. ALS is a non-cell-autonomous disorder in which also astrocytes are affected and contribute to the motoneuronal death. ALS patients experience rapid deterioration in muscle function, with an average lifespan of 2–3 years after diagnosis. Currently, the most effective therapy extends lifespan only by a few months, thus highlighting the need for new and improved therapies.

In the framework of EuroNeurotrophin, marine microorganisms from the East Mediterranean basin will be investigated aiming at the discovery of new potential neurotrophin mimetics to treat neurodegenerative diseases. To this end, the organic extracts of 85 bacterial and 15 fungal marine-derived strains were submitted to a High-Throughput Screening (HTS) for the preliminary bioactivity evaluation and prioritization of extracts capable of inhibiting the cell death of cultured motoneurones. HTS was performed using the in vitro models of human astrocytes derived from a panel of patient biosamples carrying different ALS-associated mutations and neuronal co-cultures with end point neuroprotection of motor neurons. According to the results of the preliminary evaluation, three bacterial extracts exhibited significantly higher activity than that of the positive control. The results on the isolation and structure elucidation of the metabolites of the currently under chemical investigation large-scale extracts of the most promising strains will be presented.

##### **Acknowledgments** 

This project has received funding from the European Union’s Horizon 2020 research and innovation programme under the Marie Skłodowska-Curie grant agreement No 765704 (www.euroneurotrophin.eu).

#### **New Antiinflammatory Secoeunicellins from Octocoral *Cladiella* sp.** 

ZhangZhi-Jun^1^,^2^HwangTsong-Long^3^,^4^SungPing-Jyun^1^,^2^1National Museum of Marine Biology and Aquarium, Pingtung 944, Taiwan2Graduate Institute of Marine Biology, National Dong Hwa University, Pingtung 944, Taiwan3Research Center for Chinese Herbal Medicine, Research Center for Food and Cosmetic Safety, Graduate Institute of Healthy Industry Technology, College of Human Ecology, Chang Gung University of Science and Technology, Taoyuan 333, Taiwan4Graduate Institute of Natural Products, College of Medicine, Chang Gung University, Taoyuan 333, Taiwan

**Figure 35:**
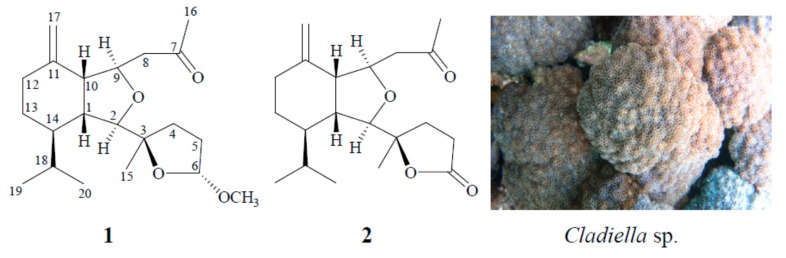
Structures of cladieunicellins W (**1**), X (**2**), and a picture of octocoral *Cladiella* sp.

Two novel 6,7-secoeunicellins, cladieunicellins W (**1**) and X (**2**) (Figure 35), were isolated from an octocoral identified as *Cladiella* sp. The structures of secoeunicellins **1** and **2** were established by spectroscopic methods. Eunicellin **1** is the first secoeunicellin possessing two tetrahydrofuran moieties and **2** represents the first 6,7-secoeunicellin possessing a γ-lactone ring. In anti-inflammatory testing, secoeunicellin 1 dispalyed inhibitory effects on the release of elastase (IC_50_ = 7.83 ± 0.83 µM) and the generation of superoxide anions (IC_50_ = 7.18 ± 1.20 µM). These results implied that the methoxy group at C-6 in 1 played important role in determining the activity of the compounds.

#### **Fucoidans from Brown Algae: Diversity of Structure and Anticancer Activity in Vitro** 

UsoltsevaRozaShevchenkoNataliaSuritsValeriyMalyarenkoOlesyaErmakovaSvetlanaG.B. Elyakov Pacific Institute of Bioorganic Chemistry, Far Eastern Branch of the Russian Academy of Sciences P. 104, 690022, Vladivostok, Russia

Brown algae produce valuable biologically active sulfated polysaccharides—fucoidans possessing different kinds of biological activity, for example, anticancer, antiviral, anticoagulant, immunomodulatory. These polysaccharides have a various structures: fucoidans can contain galactose, mannose, xylose, uronic acids residues, acetyl groups, branches; also the types of bonds between monosaccharide residues are different. The aim of the present work is comparison of structure of fucoidans from some kinds of brown algae and their anticancer activity in vitro. The structure of fucoidan fractions, isolated from *Laminaria longipes*, *Saccharina cichorioides* (Laminariaceae, Laminariales), *Sargassum duplicatum* and *Sargassum feldmannii* (Sargassaceae, Fucales), *Dictyota dichotoma* and *Padina boryana* (Dictyotaceae, Dictyotales) were investigated by chemical and instrumental methods. It was shown that the fucoidans from *L. longipes* and *S. cichorioides* were alfa-L-fucans, from *S. duplicatum* and *S. feldmannii*—galactofucans, from *Dictyota dichotoma* and *Padina boryana*—heteropolysaccharides with complex structure. All obtained fucoidans were differed significantly in details of structure. So, fucoidan from *S. cichorioides* was predominantly 1,3-linked alfa-L-fucan with small amount of branches at C2, while fucan from *L. longipes* mainly contained alternating 1,3-, 1,4- and 1,2-linked fucose residues. The both galactofucans from *S. duplicatum* and *S. feldmannii* had the main chains from fucose and galactose, but the backbone of polysaccharide from *S. feldmannii* was 1,3-linked, from *S. duplicatum*—1,4-linked. The branches of galactofucans also were different. The investigations of anticancer activity of obtained fucoidans revealed that these polysaccharides inhibited formation of colonies and growth of colon cancer cells in different manner.

##### **Acknowledgments** 

This work was supported by the RFBR Grant 19-54-54005.

#### **Implementing Metabolomics Tools to Optimise the Production of Anti-Biofilm Metabolites of Endophytic Fungi from Scottish Seaweeds** 

JaberSaif Aldeen^1^,^2^YoungLouise^1^BlackKirsty^3^TateRothwelle^1^Edrada-EbelRuAngelie^1^1Strathclyde Institution of Pharmacy and Biomedical Sciences, University of Strathclyde, 161 Cathedral Street, Glasgow G4 0RE, UK2Middle East University, Amman 11610, Jordan3Marine Biopolymers Ltd., Unit 54, Heathfield Industrial Park, Boundary Road, Ayr KA8 9DJ, UK

An increasing number of compounds with new structures and various bioactivities are recently being isolated from marine fungal sources. A metabolomics approach was used to aid media selection for the growth algal endophytic fungi and optimum production of their anti-biofilm metabolites. Anti-biofim activity has played an important role in multi-resistance of pathogenic bacteria like those of *Staphylococcus aureus*, *Klebsiella pneumoniae*, and *Pseudomonas aeruginosa.* The endophytic fungus *Dendryphilla salina* obtained from marine Scottish seaweed *Laminaria hyperborea*, was grown on nine different media namely: malt extract, rice, oat, and Wickerham media either incorporated with or without sea salt and in marine broth as well. Incubation periods were accomplished at three different growth phases. Fungal extracts were then prepared from the different fermentation conditions and their chemical profiles were analysed through nuclear magnetic resonance (NMR) spectroscopy and HPLC-coupled high-resolution mass spectrometry (HPLC-HRMS). The spectral data were processed and dereplicated using the Dictionary of Natural Products (DNP) database. The processed data were also subjected to multivariate analysis to target the bioactive metabolites and establish the optimum conditions for their production. The extracts were tested for anti-biofilm activity by Alamar Blue and planktonic solution bioassays. Three fungal extracts obtained from malt media both with and without sea salt as well as the oat media with sea salt were found active affording 100% inhibition of biofilm formation at concentration of 100 μg/mL. From the multivariate data analysis, three new metabolites were targeted for isolation work.

#### **Chemodiversity of the Irish Deep-Sea Sponge *Characella pachastrelloides*** 

AfoulloussSam^1^,^2^CalabroKevin^1^MorrowChristine^2^AllcockLouise^2^ThomasOlivier P.^1^1Marine Biodiscovery, School of Chemistry and Ryan Institute, National University of Ireland Galway University Road, H91TK33 Galway, Ireland2Zoology, School of Natural Sciences and Ryan Institute, NUI Galway, University Road, H91 TK33 Galway, Ireland

As part of a program aimed at characterizing hotspots of biological and chemical diversity in the deep sea of the North Eastern Atlantic, samples of marine sponges were collected from biogenic and geogenic reefs from a depth between 1000–3000 m. The extreme physical conditions of the deep sea force these organisms to develop unique specialized metabolites with original chemical structures and characteristics. Based on a preliminary chemical screening process, we decided to focus on the Tetractinellida sponge *Characella pachastrelloides* (Carter, 1876). We report herein the isolation and structure elucidation of four novel glycolipopeptides named characellides. These compounds (867 Da) consist of three separate moieties: a tripeptide (O-Me-Tyr, Asp, Thr), a nine/ten-member alkyl chain capped with a dimethyl substituted cyclic tetrahydropyran bound to the threonine and a 2-α-aminopyranuronamide sugar connected to the tripeptide via a *O*-glycosidic linkage with the threonine also. Characellides A and B are epimers, which only differ in their sugar moieties where the 2-α-glucuronamide replaces a 2-α-galacturonamide, with a ten member alkyl chain, while characellides C and D are epimers with a nine member alkyl chain. Additionally, 6 methylhercynine, two new poecillastroside saponins and cyanocobalamin were also isolated from this sponge.

#### **GNPS-Guided Isolation of Cyanobacterial Secondary Metabolites from the LEGE Culture Collection** 

FreitasSara R.^1^LeãoPedro N.^1^VasconcelosVitor^1^,^2^1Interdisciplinary Centre of Marine and Environmental Research (CIIMAR/CIMAR), University of Porto, Avenida General Norton de Matos, s/n, 4450-208 Matosinhos, Portugal2FCUP, Faculty of Science, Department of Biology, University of Porto, Rua do Campo Alegre, 4169-007 Porto, Portugal

Cyanobacteria are a highly diversified group of photosynthetic ancestral bacteria that have adapted to virtually all environments on Earth where light is available. Their biological and lifestyle diversity is associated with the production of a plethora of secondary metabolites, many with potent bioactivities. From genome data, we know that only a small fraction of cyanobacterial natural products has been isolated so far, therefore there are ample opportunities for discovery within this bacterial phylum.

In this work we focused on the isolation of new natural products from a selected group of cyanobacteria isolated from the Atlantic Portuguese coast and maintained in the culture collection LEGE cc, from CIIMAR research centre. The cyanobacterial strains were selected based on the perceived potential for being secondary metabolite-rich, which in turn was inferred from their phylogenetic positioning. In parallel, a bioactivity screening (anticancer and antimicrobial) was conducted to prioritize the cyanobacterial strains for new secondary metabolite isolation, complemented with GNPS-based molecular networking analysis. Fractions derived from an organic extract of a *Desmonostoc muscorum* strain revealed antibacterial activity against *Bacillus subtilis* and, in addition, showed a conspicuous molecular network cluster that was not found in other cyanobacteria extracts. These masses were not represented in the natural products databases, therefore the major compound in the cluster was selected for isolation and structure elucidation, the details of which are presented herein.

#### **Intra-Clade Molecular Networking to Target the Isolation of Anti-Proteasome Metabolites from *Salinispora* sp. from the Madeira Archipelago** 

BauermaisterAnelize^1^DiasTiago^2^ValentãoPatrícia^3^AndradePaula^3^LopesNorberto P.^1^PereiraDavid^3^GaudêncioSusana P.^2^1Faculdade de Ciências Farmacêuticas, Universidade de São Paulo, Núcleo de Pesquisa em Produtos Naturais e Sintéticos (NPPNS), Departamento de Física e Química, Ribeirão Preto, São Paulo 14040-903, Brazil2UCIBIO, Department of Chemistry, Blue Biotechnology and Biomedicine Lab, Faculdade de Ciências e Tecnologia da Universidade NOVA de Lisboa, 2829-516 Caparica, Portugal3REQUIMTE/LAQV, Laboratório de Farmacognosia, Departamento de Química, Faculdade de Farmácia, Universidade do Porto, 4050-313 Porto, Portugal

Ocean environments constitute a major source of biodiversity, harboring marine life forms capable of producing a variety of molecules with unique features, unmatched biochemical diversity and structural complexity. Thirty-four marine obligate actinomycetes strains *Salinispora* sp. (33 *S. pacifica*, 1 *S. arenicola*), isolated from sediments collected off the Madeira Archipelago [1], were screened for anti-proteasome activity. Their metabolites were dereplicated using MS/MS Molecular Networking [2–4], and the data available in literature for the genus *Salinispora*. The 26S proteasome is a catalytic complex that constitutes the main non-lysosomal pathway of protein degradation in eukaryotic cells. This complex plays a key role in cellular protease as it is involved in the degradation of poorly bounded proteins. Since degradation of these proteins is critical to the survival of cancer cells, inhibition of the proteasome is currently a prominent therapeutic strategy. The potent anti-proteasome Salinosporamide A (Marizomib) discovered in 2003 from *S. tropica*, which in Phase III of clinical trials for the treatment of multiple myeloma highlights the importance of *Salinispora* genus as a source of anti-cancer agents.

Molecular Networking LC-MS/MS metabolomics profiling unveiled the absence of previously isolated anti-proteasome metabolites from MAR1. Three *S. pacifica* strains exhibited high anti-proteasome activity (inhibition > 90%). Based on the findings of this study, the Northeast Atlantic marine-derived MAR1 *Salinispora* sp. are revealed as a potential source of novel anti-proteasome agents.

##### **Acknowledgments** 

This work was supported by the Applied Molecular Biosciences Unit—UCIBIO which is financed by national funds from FCT/MCTES (UID/Multi/04378/2019). FCT/MCTES through grants PTDC/QUIQUI/119116/2010 and IF/00700/2014.

References

1. Prieto-Davo, A.; Dias, T.; Gomes, S.E.; Rodrigues, S.; Parera-Valadez, Y.; Borralho, P.M.; Pereira, F.; Rodrigues, C.M.P.; Santos-Sanches, I.; Gaudêncio, S.P. The Madeira Archipelago As a Significant Source of Marine-Derived Actinomycete Diversity with Anticancer and Antimicrobial Potential. *Front. Microbiol.*
**2016**, *7*, 1594, doi:10.3389/fmicb.2016.01594.
2. Gaudêncio, S.P.; Pereira, F. Dereplication: racing to speed up the natural products discovery process. *Nat. Prod. Rep.*
**2015**, *32*, 779–810.
3. Wang, M.; Carver, J.J.; Phelan, V.V.; Sanchez, L.M.; Garg, N.; Peng, Y.; Nguyen, D.D.; Watrous, J.; Kapono, C.A.; Luzzatto-Knaan, T.; et al. Sharing and community curation of mass spectrometry data with Global Natural Products Social Molecular Networking. *Nat. Biotechnol.*
**2016**, 34, 828–837.
4. Bauermeister, A.; Velasco-Alzate, K.; Dias, T.; Macedo, H.; Ferreira, E.G.; Jimenez, P.C.; Lotufo; T.M.C.; Lopes, N.P.; Gaudêncio, S.P.; Costa-Lotufo, L.V. Metabolomic Fingerprinting of Salinispora From Atlantic Oceanic Islands. *Front. Microbiol.*
**2019**, 9, 3021, doi:10.3389/fmicb.2018.03021.

#### **Antibacterial Polyketides from Antarctic Sponge Derived Fungus *Penicillium citrinum* HDN151010** 

SunChunxiaoSunZichaoCheQianZhangGuojianGuQianqunLiDehaiZhuTianjiaoKey Laboratory of Marine Drugs, Chinese Ministry of Education, School of Medicine and Pharmacy, Ocean University of China, Qingdao 266003, China

The sponge-associated microorganisms have been an attractive resource for novel structural bioactive compounds. In our on-going search for bioactive secondary metabolites from Antarctic sponge-associated microorganisms, two new polyketides, named demethyldihydrocitrinone (**1**) and trimethylphenylacetate A (**2**), together with five biogenetically related known citrinin derivatives (**3**–**7**), were isolated from the crude extract of the fungus *Penicillium citrinum* HDN151010, which was isolated from an unidentified sponge collected in the Prydz bay (depth 624 m), Antarctic. Their structures, including absolute configurations, were established on basis of, M.S.; NMR, and ECD analysis. As a well-known class of polyketides, citrinins possess regular skeletons and substituted groups. Distinguished from the previously reported citrinin derivatives, compound **1** was the first case with the C-3 methyl group lost, indicating that unique geographical features of Antarctica may promote the unique metabolic pathways of the microorganisms that living in it. Compound **1** showed promising activities against *Escherichia coli*, *Vibrio Parahemolyticus*, and *Proteus vulgaris* with MIC values of 0.8, 3.1 and 6.3 μM, respectively, while compounds **3**–**7** were found to inhibit the growth of *Mycobacterium phlei* for the first time, indicating anti-tuberculousis potential.

#### **New Starfish Glycosides: Structure and Anticancer Activity** 

MalyarenkoTimofey V.IvanchinaNatalia V.KichaAlla A.StonikValentin A.G.B. Elyakov Pacific Institute of Bioorganic Chemistry, Far Eastern Branch of the Russian Academy of Sciences, Pr. 100-let Vladivostoku 159, 690022 Vladivostok, Russia

The studies of polar steroidal compounds from starfish by our research group led to the isolation of new substances with anticancer activity. For example, six new steroidal biglycosides, cariniferosides A–F, were isolated from the starfish *Asteropsis carinifera*. Sulfated compounds demonstrated a significant inhibition of colony formation of human melanoma RPMI-7951 and breast cancer T-47D cells. A new monoglycoside, leptaochotensoside A, isolated from the Far Eastern starfish *Leptasterias ochotensis*, were found to prevent EGF-induced neoplastic cell transformation of mouse epidermal JB6 Cl41 cells and inhibit the colony formation of human breast cancer cells T-47D in a soft agar. The molecular mechanism of action of leptaochotensoside A was realized through regulation of MAPK signaling pathway. The new steroidal glycosides, anthenosides V–X, and seven previously known anthenosides were isolated from the extract of the tropical starfish *Anthenea aspera*. The mixture of known anthenosides J and K was shown to possess significant anticancer activity by inducing the apoptosis of colon cancer cells HT-29.

Recently eight new triterpene glycosides, pacificusosides A H, and one previously known triterpene glycoside, cucumarioside D, were obtained from the alcoholic extract of the Far Eastern starfish *Solaster pacificus*. Triterpene glycosides are not typical metabolites of starfishes but specific for sea cucumbers. It was established, that these glycosides effectively suppressed the EGF- and TPA-induced neoplastic cell transformation of JB6 Cl41 cells.

##### **Acknowledgments** 

The study was supported by Grant No. 18-74-10028 from the RSF (Russian Science Foundation).

#### **Isolation and Structure Determination of Trikoramide A from the Marine Cyanobacterium *Symploca* sp.** 

MaYadanar Phyo^1^,^2^DingChi Ying Gary^1^OngJi Fa Marshall^1^TanLik Tong^1^1Natural Sciences and Science Education, National Institute of Education, Nanyang Technological University, 1 Nanyang Walk, Singapore 637616, Singapore2School of Biological Sciences, Nanyang Technological University, Singapore 637551, Singapore

Marine cyanobacteria are a treasure trove of unique bioactive secondary metabolites for drug discovery and development efforts. A majority of these compounds are nitrogen-containing and they belong to the hybrid polyketide-polypeptide structural class of molecules. Moreover, a number of these molecules possess potent biological activities, ranging from anticancer to anti-inflammatory property. In this study, a novel cyclic depsipeptide, trikoramide A, has been isolated from samples of the marine cyanobacterium, *Symploca* sp., collected from east coast of Bintan Island. Its planar structure was deduced by extensive 1D and 2D NMR spectroscopy in conjunction with mass spectrometry. The initial analysis using mass spectral data revealed the partial structure of trikoramide A to consist of proline, leucine, valine and phenylalanine units. In addition, trikoramide A possessed in vitro cytotoxicity towards MOLT4 human leukemic cancer cell line with IC50 value of 5.75 μM.

##### **Acknowledgments** 

This research is supported by the National Research Foundation, Prime Minister’s Office, Singapore under its Marine Science Research and Development programme (Award Nos. MSRDP-P15 and MSRDP-P34).

#### **Fragilides M-O, New 2,9,14-Triacetoxybriaranes from *Junceella fragilis*** 

LeeChieh-Yu^1^,^2^ChenYou-Ying^1^,^3^WenZhi-Hong^3^SungPing-Jyun^1^,^2^1National Museum of Marine Biology and Aquarium, Pingtung 944, Taiwan2Graduate Institute of Marine Biology, National Dong Hwa University, Pingtung 944, Taiwan3Department of Marine Biotechnology and Resources, National Sun Yat-sen University, Kaohsiung 804, Taiwan

**Figure 36:**
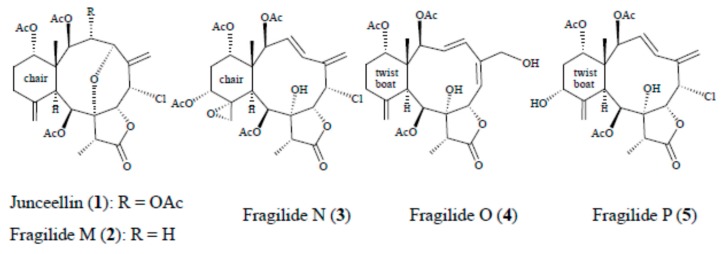
Structures of Junceellin (1) and fragilides M–P (2–5).

Chemical investigation of the EtOAc-soluble fraction from the MeOH/DCM extract of a sea whip gorgonian coral *Junceella fragilis* afforded four polyacetoxybriaranes, including a known metabolite, junceellin (**1**), along with four new analogues, fragilides M–P (**2**–**5**). The absolute configuration of **1** was determined by X-ray analysis and the structures of **2**–**5** were elucidated on the basis of spectroscopic methods (Figure 36) The proton chemical shifts of briarane-type natural products containing an 11,20-exocyclic carbon-carbon double bond were summarized and the difference between the two olefin protons (H-20a/b) was smaller than 0.2 ppm, whereas the methylidenecyclohexane rings exhibited a twisted boat conformation. Owing to the chemical shifts of the C-20 methylene protons, the configurations of the methylidenecyclohexane rings in briaranes **4** and **5** were concluded to be a twisted boat conformation and this finding was further supported by the NOESY correlations between H-10/H-20b; and H-12/H3-15 in the NOESY spectra of **4** and **5**. Anti-inflammatory activity of briaranes **1**–**5** in terms of reducing the expressions of elastase and superoxide anions by human neutrophils were described.

#### **Domhainone: A New Oxidized Steroid from the Irish Deep-Water Coral *Jasonisis* sp.** 

YoungRyan M.^1^NguyenTrang^2^SunXingmin^2^JohnsonMark P.^1^BakerBill J.^3^AllcockA. Louise^1^1Ryan Institute, National University of Ireland, Galway, H91 CF50, Ireland2Molecular Medicine, College of Medicine, University of South Florida, Tampa, FL 33620, USA3Department of Chemistry, College of Arts & Science, University of South Florida, Tampa, FL 33620, USA

**Figure 37:**
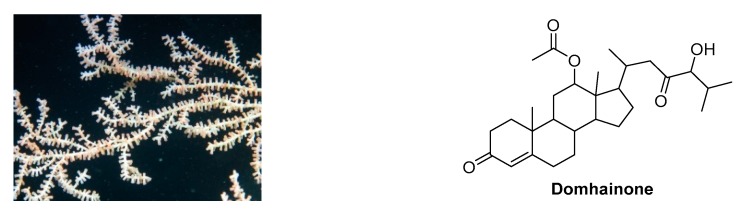
*C. difficile* and Structure of domhinone.

Submarine canyons are often biodiversity and biomass hotspots and the Whittard Canyon, located approximately 300 km off the Irish mainland, with steep cliffs inhabited by a plethora of corals and sponges is no exception. One species which populates these waters is a bamboo coral of the genus *Jasonisis*. In a high-throughput screening effort, the fractions originating from a chemical extract of this coral were identified as active against the pathogenic bacterium *Clostridioides difficile*. Infections of *C. difficile* have emerged as a significant public health threat worldwide. In the United States, *C. difficile* caused an estimated half a million infections and 29,000 deaths in 2012. The DCM extract of the bamboo coral was fractionated on C18 via VLC. The active fraction was subsequently purified via HPLC yielding the major metabolite domhainone (Figure 37). The final structure was elucidated using 1D/2D NMR spectroscopy and HRMS.

#### **(α-d-Glucopyranosyl-(1→2)-β-d-fructofuranoside 3)-l-Tryptophan Ester** 

KyeremehKwaku^1^KwainSamuel^1^TeteviGilbert Mawuli^1^CamasAnil Sazak^2^CamasMustafa^2^DofuorAboagye Kwarteng^3^DengHai^4^JasparsMarcel^4^1Marine and Plant Research Laboratory of Ghana, Department of Chemistry, School of Physical and Mathematical Sciences, University of Ghana, P.O. Box LG 56 Legon-Accra, Ghana2Department of Bioengineering, Munzur University, 62000 Tunceli, Turkey3Department of Biochemistry, Cell and Molecular Biology, University of Ghana, Legon, Ghana4Marine Biodiscovery Centre, Department of Chemistry, University of Aberdeen, Old Aberdeen AB24 3UE, UK

The *Mycobacterium* sp. BRS2A-AR2 is an endophyte of the mangrove plant *Rhizophora racemosa*, which grows along the banks of the River Butre, in the Western Region of Ghana. Spectroscopy and spectrometry aided chemical profiling of fermentation extracts produced by the strain led to the isolation of the new compound, (α-d-Glucopyranosyl-(1→2)-β-d-fructofuranoside)-l-tryptophan ester (**1**). Compound **1** is an aminoglycoside consisting of a tryptophan moiety esterified to a disaccharide made up of β-d-fructofuranose and α-d-glucopyranose sugars. The full structure of **1** was determined using, U.V.; IR, 1D, 2D-NMR and HRESI-LC-MS data. When tested against *Trypanosoma brucei brucei*, 1 (IC_50_ 11.25 μM) was just as effective as Coptis japonica (IC_50_ 8.20 μM). It is possible that, compound **1** interferes with the normal uptake and metabolism of tryptophan in the *Trypanosoma brucei brucei* parasite.

#### **Metabolomics at the Service of Natural Product Drug Discovery** 

RoullierCatherineUniversity of Nantes, EA 2160—MMS, 9 rue Bias, 44035 Nantes, France

In natural product drug discovery, several strategies have emerged to highlight the most promising compounds within complex mixtures (fractions or crude extracts) using tools from the metabolomics field. With the huge advances in analytical techniques and the development of bioinformatics, new horizons for natural products drug discovery open. Indeed, a great deal of interest has raised among the scientific community towards the use of metabolomics approaches to highlight, very early in the process, the most interesting compounds to circumvent the drawbacks of bioguided fractionation. In this presentation, the recent developments made by our lab in this area will be presented. In summary, based on LC-HRMS profiles, a complete workflow has been developed combining different freely-available and in-house bioinformatics tools to prioritize subsequent purification. It then allows to highlight from complex mixtures the most valuable molecules because of either:^1^ their potential novelty (based on an automated annotation process) [1, 2] the presence of the interesting pharmacophores Cl or Br (based on the R MeHaloCoA package) [2,3] a high score of importance in the bioactivity observed (based on the recently developed R FiBiCo script) [3]. These recent advances in the field will be presented together with examples of application to marine fungal extracts.

References

1. Bertrand, S.; Guitton, Y.; Roullier, C. Successes and pitfalls in automated dereplication strategy using liquid chromatography coupled to mass spectrometry data: A CASMI 2016 experience *Phytochem. Lett.*
**2017**, *21*, 297–305, doi:10.1016/j.phytol.2016.12.025.
2. Roullier, C.; Guitton, Y.; Valery, M.; Amand, S.; Prado, S.; Robiou du Pont T.; Grovel O.; Pouchus Y.F. Automated Detection of Natural Halogenated Compounds from LC-MS Profiles-Application to the Isolation of Bioactive Chlorinated Compounds from Marine-Derived Fungi. *Anal. Chem.*
**2016**, *88*, 9143–9150, doi:10.1021/acs.analchem.6b02128.
3. Ory, L.; Nazih, E.H.; Daoud, S.; Mocquard, J.; Bourjot, M.; Margueritte, L.; Delsuc, M.A.; Bard, J.M.; Pouchus, Y.F.; Bertrand, S.; et al. Targeting bioactive compounds in natural extracts—Development of a comprehensive workflow combining chemical and biological data. *Anal. Chim. Acta*
**2019**, *1070*, 29–42, doi:10.1016/j.aca.2019.04.038.

#### **Chemical Investigation of Two Sponges from Rodrigues Island (Indian Ocean) for the Discovery of Molecules with Healthy Ageing Promoting Activity** 

CamposPierre-Eric^1^BollaroFlavia^1^ClercPatricia^1^BoyerJean-Bernard^1^VoodgNicole De^2^FokialakisNikolas^3^TrougakousIoannis P.^4^GardikisKonstantinos^5^BignonJérôme^6^Le GoffGéraldine^6^MoriouCéline^6^Al-MourabitAli^6^OuazzaniJamal^6^BialeckiAnne^1^1LCSNSA, Université de la Réunion, Ile de la Réunion, 97744 Saint-Denis, France2Naturalis Biodiversity Center, Darwinweg 2, 2333 CR Leiden, The Netherlands3Faculty of Pharmacy, National & Kapodistrian University of Athens, 15771 Athens, Greece4Faculty of Biology, National & Kapodistrian University of Athens, 15784 Athens, Greece5APIVITA SA, Research and Development Department, Industrial Park of Markopoulo, Markopoulo Mesogaias, 19003 Athens, Greece6ICSN-CNRS, Gif sur Yvette, 91190 Paris, France

As the population of developed countries is ageing, the prevalence of a variety of age-related diseases, such as Alzheimer’s disease, cardiovascular diseases, neurodegeneration or cancers is increasing. In order to counteract this major healthcare challenge, marine natural products represent an extraordinary reservoir of structurally diverse bioactive metabolites. In this regard, TASCMAR, a EU-funded Horizon 2020 project, aspires to develop new tools and strategies on discovering novel marine derived compounds with healthy ageing promoting activity. The LCSNSA laboratory, located at Reunion Island and involved in TASCMAR project, has explored marine invertebrates and symbionts from the South West part of Indian Ocean.

**Figure 38:**
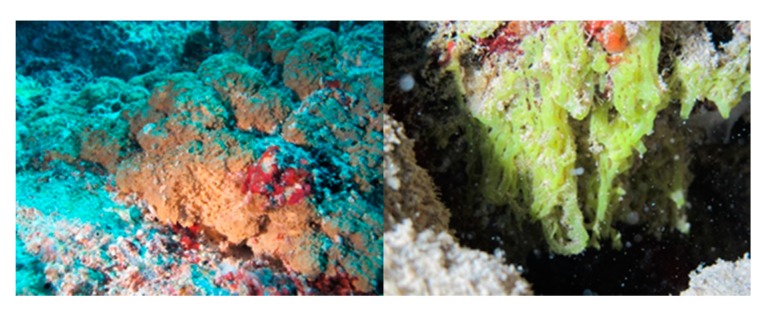
In situ photographies of Agelas sp. (**left**) and Ernstia naturalis (**right**).

Thus, two sponges, *Agelas* sp. and *Ernstia naturalis* (Figure 38), from Rodrigues have been investigated. The genus Agelas, already well studied and known to produce bioactive compounds, can be considered as a promising source of metabolites with anti-ageing activity. Sponges of the genus Ernstia on their side have never been studied for their chemical composition. The crude extract of *Agelas* sp. was found to inhibit FYN kinase (alzheimer’s disease), CDK7 (cancer) and tyrosinase (skin ageing). The crude extract of Ernstia naturalis was found to inhibit proteasome (oncogenesis) and tyrosinase.

This communication will provide an outline of the methodology used for the chemical investigation of these two sponges. The study of *Agelas* sp. led to the isolation of five bromopyrrole alkaloids and investigation of Ernstia naturalis yielded to the isolation of four imidazole alkaloids. These nine compounds were characterized by HRMS and NMR. Bioassays on the pure compounds are in progress.

#### **Isolation of Minutissamide Derivatives from a Marine Cyanobacteria Collected in Palau** 

PérezMartaCoelloLauraFernándezRogelioCuevasCarmenR&D, PharmaMar, S.A., Avda. de los Reyes 1, Pol. Ind. La Mina, 28770 Colmenar Viejo, Madrid, Spain

Blue-green algae, the most photosynthetic prokaryotes in nature, have been shown to be prolific producers of bioactive secondary metabolites, and a considerable number of these toxins have been developed as anticancer agents [1]. A major class of secondary metabolites from cyanobacteria are polyketide-conjugated nonribosomal peptides, commonly known as lipopeptides. A representative group of these structures are the minutissamides [2], which have been reported to exhibit antiproliferative activity against the MDA-MB-435 human melanoma cancer cell line. This study led to the identification of four new minutissamide analogs from an extract of a cyanobacteria collected in the north of Palau, where different ecosystems such as mangroves, channels and coral reefs coexist. The sample was collected under the Research Agreement of Palau International Coral Reef Center (PICRC)/Republic of Palau (ROP) and PharmaMar. The planar structures were determined using various spectroscopic techniques including HRESIMS and 1D and 2D NMR experiments. The absolute configurations of the amino acid residues were assigned using Marfey’s method after acid hydrolysis, while the relative stereochemistry of some centres was determined through JBC/ROESY analysis. Details of the isolation and spectroscopic data leading to the structure elucidation of the compounds, as well as their biological activities will be presented. The existence of numerous lipopeptides produced by cyanobacteria raises the question of their ecological relevance.

##### **Acknowledgments** 

We gratefully acknowledge the help of E. Gómez for their valuable technical assistance. The present research was financed in part by Grants from Ministerio de Ciencia, Innovación y Universidades of Spain (AGL2015-63740-C2-2-R and RTC-2016 4611-1, Inmunotop project), cofunded by the FEDER Programme from the European Union.

References

1. Nunnery, J.K.; Mevers, E.; Gerwick, W.H. Biologically active secondary metabolites from marine cyanobacteria. *Curr. Opin. Biothecnol.*
**2010**, *21*, 787–793.
2. Kahk, H.S.; Sturdy, M.; Krunic, A.; Kim, H.; Shen, Q.; Swanson, S.; Orjala, *J.* Minutissamides E–L, antiproliferative cyclic lipodecapeptides from the cultured freshwater cyanobacterium cf. *Anabaena* sp. *Bioorg. Med. Chem.*
**2012**, *20*, 6134–6143.

#### **Isolation of Serrawettin Analogs from a Marine Bacteria *Vibrio harveyi* with the Assistance of Molecular Networking** 

CruzPatriciaGonzálezJuan JoséRodríguezPilarZuñigaPazAltaresRaquelPérezMartade la CalleFernandoCuevasCarmenR&D, PharmaMar, S.A., Avda. de los Reyes 1, Pol. Ind. La Mina, 28770 Colmenar Viejo, Madrid, Spain

Serrawettins are non-ionic biosurfactant lipopeptides produced by the proteobacteria *Serratia marcescens*. Four molecular species, serrawettins W2, W4, W5 and W6 have also been reported from *Serratias* associated with roadkill mammals [1]. As part of our continuous efforts to find new secondary metabolites with anticancer properties, a bioactive extract of a cultured *Vibrio harveyi* (HEL-17) isolated from a bryozoan collected in the Pacific Ocean was selected for further scale up. The sample was collected under the Research Agreement of Palau International Coral Reef Center (PICRC)/Republic of Palau (ROP) and PharmaMar. A literature search indicated that there are only two reports concerning the composition of secondary metabolites from *Vibrio harveyi*. This fact encouraged us to characterize the compounds present in this extract using LC-MS/MS analysis combined with molecular networking and NMR. This study led to the isolation of six new serrawettin analogs together with other known families of glycolipids and fatty acids, showing the importance of combining different analytical methods. Details of the producer microorganism, isolation, and spectroscopic data leading to the structure elucidation of the new compounds, as well as their biological activity will be described in this communication.

##### **Acknowledgments** 

The present research was financed in part by Grants from Ministerio de Ciencia, Innovación y Universidades of Spain (RTC-20164892-1, DESPOL project).

References

1. Motley, J.; Stamps, B.W.; Mitchell, C.A.; Thompson, A.T.; Cross, J.; You, J.; Powell, D.R.; Stevenson, B.S.; Cichewicz, R.H. Opportunistic Sampling of Roadkill as an Entry Point to Accessing Natural Products Assembled by Bacteria Associated with Non-anthropoidal Mammalian Microbiomes. *J. Nat. Prod*. **2017**, *80*, 598–608.

#### **New Peptides Isolated from the Indonesian Sponge *Theonella* sp.** 

FernándezRogelioPérezMartaBuenoSantiagoCuevasCarmenR&D, PharmaMar, S.A., Avda. de los Reyes 1, Pol. Ind. La Mina, 28770 Colmenar Viejo, Madrid, Spain

Sponges of the genus *Theonella* sp. have been found to contain a vast variety of bioactive metabolites, including macrolides, cyclic and linear peptides, steroids and alkaloids. Most of them have exhibited significant cytotoxic activity [1].

In the course of our screening program to isolate novel compounds with antitumor properties from marine sources, we have isolated two new linear peptides from a *Theonella* specimen, which was collected off the coast of Seram in Indonesia under collaboration with Andalas University.

These compounds were obtained by bioassay-guided fractionation of an organic extract of the organism, using VLC RP-18 chromatography and reverse phase semi-preparative HPLC. Structure elucidation of these new metabolites was carried out by spectroscopic methods including MS, ^1^H, ^13^C and ^2^D-NMR. The stereochemistry of the amino acids was determined by hydrolysis followed by derivatization with Marfey’s reagent and comparison with commercial standards by HPLC-MS. The two new linear peptides isolated contain an unprecedented tribromopyrrol unit in their structure. This finding highlights *Theonella* and its microbiota as an endless source of novel structures.

##### **Acknowledgments** 

We gratefully acknowledge the help of E. Gómez for their valuable technical assistance. The present research was financed in part by Grants from Ministerio de Ciencia, Innovación y Universidades of Spain (AGL2015-63740-C2-2-R and RTC-2016 4611-1, Inmunotop project), cofunded by the FEDER Programme from the European Union.

References

1. Micheal, C.; Wilson, M.C.; Mori, T.; Rückert, C.; Uria, A.R.; Helf, M.J.; Takada, K.; Gernert, C.; Steffens, U.A.E.; Heycke, N.; et al. An environmental bacterial taxon with a large and distinct metabolic repertoire. *Nature*
**2014**, *506*, 58–62.

## Poster Session 2: Drug Discovery and Development, and Biosynthesis of Marine Natural Products

### ***Theme 3. Drug Discovery and Development*** 

#### **Chromomycins from *Streptomyces* sp. from the Zoanthid *Palythoa caribaeorum* Induce Immunogenic Cell Death in Metastatic Melanoma** 

WilkeDiego V.^1^FlorêncioKatharine G. D.^1^PintoFrancisco C. L.^2^SahmBianca Del B.^3^EdsonEvelline A.^1^BauermeisterAnelize^3^,^4^Costa-LotufoLetícia V.^3^PessoaOtília D. L.^2^1Drug Research and Development Center, Department of Physiology and Pharmacology, Federal University of Ceara, Fortaleza, CE 60430-275, Brazil2Department of Organic and Inorganic Chemistry, Federal University of Ceara, Fortaleza, CE 60021-970, Brazil3Department of Pharmacology, Institute of Biomedical Sciences, University of Sao Paulo, São Paulo 05508-900, Brazil4Department of Physics and Chemistry, School of Phaceteutical Sciences of Ribeirao Preto, University of Sao Paulo, Ribeirao Preto, São Paulo 14040-903, Brazil

Advanced metastatic melanoma (AMM) is considered incurable. Some antitumor chemotherapics, e.g., anthraciclines and oxaliplatin, induce activation of the immune system through a complex spatiotemporal process called immunogenic cell death (ICD). Tumor cells undergoing ICD function as vaccine, releasing “danger signals” (DAMPs), which act as adjuvants, and neoantigens of the tumor recognized as antigens. Discovery of ICD inducers against AMM is interesting for discovery of leads with a higher translational significance. Megabiodiversity of Brazilian Exclusive Economic Zone (BEEZ) is an invaluable source of bioactive compounds. As part of a broader prospection program, we made a library of 1500 marine strains recovered from invertebrates and sediment from BEEZ, and 15% of the extracts depicted cytotoxicity. This work aimed to identify ICD inducers on AMM model. First we isolated four highly cytotoxic dextrorotatory chromomycins (CA5, CA6, CA7, CA8), against a mini-panel of tumor cells with IC50 values ranging from 200 pM to 800 nM (MTT assay), from *Streptomyces* sp. BRA-384 strain (Chagas et al., JBCS, 2019, accepted). Then we performed in vitro assays with metastatic melanoma (B16-F10) to evaluate ICD signals and a vaccination assay. Chromomycins A5–A8 inhibited completely colony formation in nanomolar range. Furthermore, we observed, by flow cytometry, cell stress such as increased granularity, acidic vesicles organelles formation, calreticulin externalization and cell death as pyknosis and plasmatic membrane disruption. C57Bl/6 mice vaccinated with CA5 treated-B16-F10 cells control the tumor growth when compared with both, negative (saline) and positive (doxorubicin treated-B16-F10 cells) controls. Studies are ongoing to evaluate more ICD markers in vitro and the immune system involvement in vivo.

##### **Acknowledgments** 

CNPq_#421124/2016-4, CNPq/INCTBionat_#465637/2014-0.

#### ***Desmarestia antarctica* (Desmarestiales, Phaeophyceae): An Antarctic Derived Source of Bioactive Compounds against Protozoan Diseases** 

TeixeiraThaiz R.^1^SantosGustavo S.^1^RangelKaren C.^1^GasparLorena R.^1^FilhoPéricles G. Abreu^1^PereiraLuís M.^1^YatsudaAna P.^1^ClementinoLeandro C.^2^GraminhaMárcia A. S.^2^JordãoLaís G.^3^,^4^PohlitAdrian M.^5^VilelaLeonardo Z.^5^ColepicoloPio^5^DebonsiHosana M.^1^1School of Pharmaceuticals Sciences of Ribeirão Preto—University of São Paulo, Ribeirão Preto, SP 14040-903, Brazil2School of Pharmaceuticals Sciences of São Paulo State University Júlio de Mesquita Filho—UNESP, Araraquara, SP 14800-903, Brazil3National Institute of Amazonian Research—INPA, Manaus, AM 69067375, Brazil4Federal University of Amazonas—UFAM, Manaus, AM 69080900, Brazil5Institute of Chemistry—University of São Paulo, São Paulo, SP 05508-000, Brazil

Leishmaniasis and malaria are infectious diseases affecting millions of people worldwide. *Neospora caninum* is related to reproductive problems in cattle leading to relevant economic losses. The lack of specific treatments against neosporosis, the side effects of available drugs to treat leishmaniasis and the *Plasmodium* spp. resistance to antimalarial drugs leads to the search of new anti-protozoan molecules, especially from underexplored sources as the Antarctic marine environment. Herein, the chemical profile of volatile of *Desmarestia antarctica* was analyzed using GC-MS and the crude extract and fractions were evaluated regarding to their antiparasitic potential. The bioactivity was evaluated against promastigotes of *Leishmania amazonensis* (IC_50-leishmania_), tachyzoites of *Neospora caninum* Lac-Z-strain (IC_50-neospora_) and trophozoites of multi-drug-resistant *Plasmodium falciparum* K1 (IC_50-plasmodium_) as well as their cytotoxicity (CC_50_) against 3T3 BALB/c fibroblasts, to determine the selective index (CC_50/IC50_). Fractions D.A-FD (IC_50_-leishmania = 55.1 ± 0.1 μg/mL; SI 2.1) and DA-FH (IC_50_-leishmania = 57.1 ± 2.0 μg/mL; SI 2.4) showed good activity and were two-fold more selective to *L. amazonensis*, rather than to the host cells. The active fractions in the *N. caninum* proliferation assay were D.AFD (IC_50_-neospora = 1.2 ± 1.6 μg/mL) D.A-FF (IC_50_ = 3.1 ± 2.1 μg/mL) and D.A-FH (IC_50_ = 3.1 ± 2.0 μg/mL). The most active fraction (D.A-FD) in both *Leishmania amazonensis* and *Neospora caninum* assays was also screened against Plasmodium falciparum and presented weak activity (IC_50_-plasmodium = 19.1 ± 3.9 μg/mL). The major volatile compounds identified by GC-MS analysis of DA-FD were n-tetradecanoic acid, hexadecanoic acid and elaidic acid methyl ester. Next steps: LC-MS/MS dereplication acquisition data and isolation of molecules from the most active fractions.

#### **In Silico Cell-Based Models *en route* to the Discovery of Lead-Like Anticancer and Antibacterial Drugs** 

CruzSara^1^DiasTiago^1^,^2^GomesSofia E.^3^BorralhoPedro M.^3^RodriguesCecília M. P.^3^GaudêncioSusana P.^1^,^2^PereiraFlorbela^1^1LAQV-REQUIMTE, Department of Chemistry, Faculty of Science and Technology, Universidade NOVA de Lisboa, 2829-516 Caparica, Portugal2UCIBIO-REQUIMTE, Department of Chemistry and Department of Life Sciences, Faculty of Science and Technology, Universidade NOVA de Lisboa, 2829-516 Caparica, Portugal3Research Institute for Medicines (iMed.ULisboa), Faculty of Pharmacy, Universidade de Lisboa, 1964-003 Lisboa, Portugal

**Figure 39:**
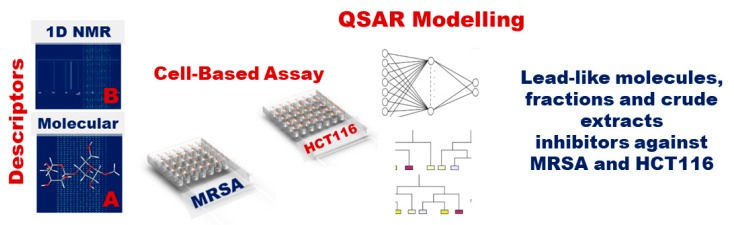
QSAR approaches **A** and **B**.

In recent years, there has been a growing interest in NP-like scaffolds mainly due to the decreasing number of new molecular entities in drug development pipelines and to the higher success rate of MNPs when compared with the synthetic industry average. Computer-aided drug design methods have emerged as powerful tools in the development of therapeutically important small molecules with higher hit rates than those obtained from more conventional approaches such as high throughput screening approaches. Herein, two quantitative structure–activity relationship (QSAR) approaches (A and B) built with machine learning techniques were presented for the prediction of antibacterial activity against the pathogen MRSA [1] and anticancer activity against the human colon carcinoma cell line HCT116 [2] (Figure 39).

The NMR QSAR classification models were built using 1D NMR data as descriptors, from crude extracts, fractions, and pure compounds obtain from actinobacteria isolated from marine sediments collected off the Madeira Archipelago [3]. A new NP drug hit discovery strategy was developed, by frontloading these samples with 1D NMR descriptors (approach B), contributing to alleviate NP discovery drawbacks of time consumption and biological activity screening-associated costs [1,2].

##### **Acknowledgments** 

This work was supported by the Associate Laboratory for Green Chemistry-LAQV which is financed by national funds from FCT/MCTES (UID/QUI/50006/2019) and by the Applied Molecular Biosciences Unit—UCIBIO which is financed by national funds from FCT/MCTES (UID/Multi/04378/2019). FCT/MCTES through grants PTDC/QUIQUI/119116/2010 and IF/00700/2014. FP also thanks to FCT/MCTES for the Norma transitória DL 57/2016 Program Contract.

References

1. Dias, T.; Gaudêncio, S.P.; Pereira, F. A Computer-Driven Approach to Discover Natural Product Leads for Methicillin-Resistant *Staphylococcus aureus* Infection Therapy. *Mar. Drugs*
**2019**, *17*, 16, doi:10.3390/md17010016.
2. Cruz, S.; Gomes, S.E.; Borralho, P.M.; Rodrigues, C.M.P.; Gaudêncio, S.P.; Pereira, F. *In Silico* HCT116 Human Colon Cancer Cell-Based Models En Route to the Discovery of Lead-Like Anticancer Drugs. *Biomolecules*
**2018**, *8*, 56, doi:10.3390/biom8030056
3. Prieto-Davo, A.; Dias, T.; Gomes, S.E.; Rodrigues, S.; Parera-Valadez, Y.; Borralho, P.M.; Pereira, F.; Rodrigues, C.M.P.; Santos-Sanches, I.; Gaudêncio, S.P. The Madeira Archipelago As a Significant Source of Marine-Derived Actinomycete Diversity with Anticancer and Antimicrobial Potential. *Front. Microbiol.*
**2016**, *7*, doi:10.3389/fmicb.2016.01594.

#### **Computer-Aided Discovery of New Anti-Leukemic Epigenetic Drugs Based on Free-Access Marine Compound Libraries** 

KamikiJessikaRoqueAna C. A.BarbosaArménio J. MouraUCIBIO, Departamento de Química, Faculdade de Ciências e Tecnologia, Universidade Nova de Lisboa, 2829-516 Caparica, Portugal

**Figure 40:**
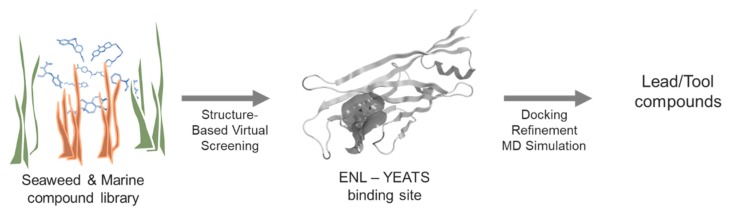
Depiction of the Drug Discovery protocol for new marine based ENL ligands. About 1000 seaweed metabolites and 1000 free access marine compounds (180 > MW > 500 g/mol) were collected and curated in a molecular library for Structure-based Virtual Screening. A docking screening using MOE was performed. The top hits were refined and further studied with Molecular Dynamics Simulations.

In Acute Myeloid Leukemia (AML) innovative drug therapies are crucial to improve its poor prognostic. Recently, the ENL-YEATS 3-D structure was unveiled1, an epigenetic reader protein required for the maintenance of AML which depletion has anti-leukemic effects in vitro and in vivo [1,2]. A structure-based drug discovery protocol was employed to search for new inhibitors for this novel anti-AML target (Figure 40). A library of marine compounds based on free-access databases was assembled and curated for Virtual Screening. The ENL binding site was selected to perform docking-based virtual screenings using MOE with induced-fit docking protocol [3]. A set of lead compounds with higher docking scores than the native acetylated lysine was selected. Their binding stability and interactions were assessed through MD Simulations. The most promising ligands will be tested with ENL-YEATS domain to validate the computational protocol and assess experimental affinity values.

References

1. Wan, L.; Wen, H.; Li, Y.; Lyu, J.; Xi, Y.; Hoshii, T.; Joseph, J.K.; Wang, X.; Loh, Y.E.; Erb, M.A.; et al. ENL links histone acetylation to oncogenic gene expression in acute myeloid leukaemia.. *Nature*
**2017**, *543*, 265–269.
2. Erb, M.A.; Scott, T.G.; Li, B.E.; Xie, H.; Paulk, J.; Seo, H.S.; Souza, A.; Roberts, J.M.; Dastjerdi, S.; Buckley, D.L.; et al. Transcription control by the ENL YEATS domain in acute leukaemia. *Nature*
**2017**, *543*, 270–274.
3. *Molecular Operating Environment (MOE)*; Chemical Computing Group ULC: Montreal, QC, Canada, 2018.

#### **Anti-Inflammatory Activity of Microalgae-Derived Bioactives with Potential Dermoprotective Apllications** 

MarreirosCatarina^1^IsmaelKovan^1^ViegasCarla^1^,^2^AraújoNuna^1^AndradeMiguel^3^RosaBruno^3^LimaRui^3^FernandesVictor Santos^3^BarreiraLuísa^1^VarelaJoão^1^SimesDina^1^,^2^1Centre of Marine Sciences (CCMAR), University of Algarve, 8005-139 Faro, Portugal2GenoGla Diagnostics, CCMAR, University of Algarve, 8005-139 Faro, Portugal3Centro Hospitalar Universitário Lisboa Norte, EPE, 1600-190 Lisboa, Portugal

Low-grade chronic inflammation, or inflammaging, is a pathological process associated with age-related diseases such as diabetes, atherosclerosis and skin aging. Chronic inflammation in the skin can be associated with skin aging, but also with severe pathological conditions such as cancer and psoriasis. New and innovative drugs able to dampen inflammation are urgently needed.

Marine species, known to represent a highly diverse and cost-effective source of bioactive compounds, are of great interest due to its potential health-promoting effects. In particular, microalgae have been recognized as a promising source of novel bioactive compounds with potential preventive and therapeutic applications for several inflammatory skin diseases.

In this work, we aimed to search for novel microalgae-derived extracts/characterized fractions with anti-inflammatory properties, relevant for dermoprotective applications. Ethanolic and aqueous extracts from 4 microalgae were screened for their anti-inflammatory activities in human macrophage-differentiated THP-1 cells (Mac-THP-1). Extracts were further fractionated by liquid-liquid extraction, and the resulting fractions were re-screened for anti-inflammatory activity in Mac-THP-1 cells. Selected extracts/fractions were tested for their anti-inflammatory properties in skin ex-vivo fragments, by measuring cytokine levels, histological alterations and gene expression of specific marker genes. Our results showed that a selection of microalgae extracts/fractions were able to decrease the inflammatory reaction in a closer-to-in-vivo human skin model, indicating that these microalgae-derived bioactive formulations have great potential as therapeutics for inflammatory-related skin diseases. This skin model is also a valuable tool to exploit the mechanism of action, the efficiency and toxicity of promising bioactives.

##### **Acknowledgments** 

Catarina Marreiros is the recipient of a 0055 ALGARED+5E fellowship. Nuna Araújo is the recipient of the Portuguese Science and Technology Foundation (FCT) fellowship SFRH/BD/111824/2015. This research was funded by the Project 0055 ALGARED+ 5E—INTERREG V-A España-Portugal project and by the Portuguese national funds from FCT—Foundation for Science and Technology through project UID/Multi/04326/2019.

#### **The Marine Mesophotic Zone as a Source for the Discovery of Novel Bioactive Molecules** 

TrougakosIoannis P.^1^FokialakisNikolas^2^SklirouAimilia^1^PapanagnouEleni-Dimitra^1^CheimonidiChristina^1^BairaEirini^2^Le GoffGéraldine^3^GianniouDespoina D.^1^VlachouPinelopi^2^BialeckiAnne^4^BenayahuYehuda^5^JerabekMoran^6^OuazzaniJamal^3^1Faculty of Biology, National & Kapodistrian University of Athens, 15784 Athens, Greece2Faculty of Pharmacy, National & Kapodistrian University of Athens, 15771 Athens, Greece3ICSN-CNRS, 91198 Gif sur Yvette, France4Université de la Réunion, 97715 Ile de la Réunion, France5Tel Aviv University, 69978 Tel Aviv, Israel6Crelux GmbH, 82152, Martinsried, Germany

Metazoans respond to harmful challenges by mounting anti-stress responses; this adaptation along with the evolvement of metabolic networks, were fundamental forces during evolution. Central to anti-stress responses are a number of short-lived transcription factors that by functioning as stress sensors mobilize cytoprotective genomic responses aiming to eliminate stressors and restore tissue homeodynamics. We have found that increased expression these cytoprotective pathways can enhance stress tolerance and longevity. Given that natural products are likely the only feasible mean for increasing healthy ageing in humans we employed the TASCMAR platform as a unique source for the discovery of novel bioactive small molecules with healthy ageing promoting activity. Specifically, in the frame of TASCMAR along with the existing collection (180) of invertebrates (MACLIB library), 179 marine invertebrates species (TARMAC library) were collected from the under-investigated mesophotic zone (between 30 and 100 m depth) of the Indian ocean, the Red sea and the Mediterranean sea. Furthermore, more than 300 (MICLIB library) and 312 (TARMIC library) associated microorganisms of MACLIB and TARMAC libraries respectively, were isolated. The samples were extracted and libraries of extracts, microfractions or pure molecules (following dereplication) were sent for biological evaluation against a wide range of different targets involved in ageing or age-related diseases (e.g., cancer or neurodegeneration). These targets include catalase, sirtuin 1, CDK7, proteasome, fyn kinase, tyrosinase and elastase. The rationale behind the selection of these targets along with our findings exemplifying the identification of numerous novel bioactive molecules from all libraries will be presented.

##### **Acknowledgments** 

TASCMAR project (www.tascmar.eu) is funded by the European Union in the frame of H2020 (GA No 634674).

#### **First Insights into the Chemical Composition of the Mucus of the Sponge *Haliclona viscosa* Isolated from Irish Marine Waters** 

KeekanKishor Kumar^1^PanditAbhay^2^ThomasOlivier P.^1^1Marine biodiscovery, School of Chemistry and Ryan Institute, National University of Ireland Galway, University Road, H91 TK33 Galway, Ireland2Centre for Research in Medical Devices (CÚRAM), Biomedical Sciences, National University of Ireland Galway, H91 W2TY Galway, Ireland

Mucus secretions of marine invertebrates, such as sponges, corals, worms perform important functions in their lifetime due to their unique *biochemical and physical properties.* Mucus *generally consists of* (glyco) proteins (mucins) and polysaccharides. The viscoelastic, adhesive, antimicrobial and other properties of the mucus may have potential biomedical applications. Among the several sponges screened for mucus production from Irish waters, *Haliclona viscosa* was one of the promising candidate identified in the study. Earlier reports of *H. viscosa* revealed more than thirty structurally different polymeric 3-alkyl pyridinium salts (poly-APS/3-APS) with a wide range of biological activities. Most of the previous studies employed organic solvents and or the precipitation of aqueous extract using organic solvents for the isolation of poly-APS. However, due to its high water solubility and also to characterize other water-soluble macromolecules (proteins/glycoproteins), the present study was undertaken. The aqueous extract (hand squeezed, after centrifugation at 10,000 rpm for 1 hr at 4 °C) of *H. viscosa* was subjected to preparative ultracentrifugation followed by Polyacrylamide Gel Electrophoresis (PAGE) and Nuclear Magnetic Resonance (NMR) spectroscopic studies. Ultracentrifugation yielded four fractions at various densities (1.00, 1.07, 1.13 and 1.20 g/mL). Based on NMR, fraction 1 and 2 confirms the presence of 3-APS and most of the proteins were concentrated in the fraction three according to electrophoretic separation. Fraction four was devoid of 3-APS and contained traces of proteins. Further work is under progress to characterize the poly-APS, proteins/glycoproteins by Dynamic Light Scattering (DLS), Liquid chromatography-mass spectrometry (LC-MS) based proteomics.

#### **Investigations of the Anticancer Activity and Mechanism of Action of the Derivatives of Marine Alkaloid Ascididemine** 

DyshlovoySergey A.^1^,^2^,^3^KauneMoritz^1^HauschildJessica^1^PelageevDmitry N.^2^RohlfingTina^1^BokemeyerCarsten^1^StonikValentin A.^2^von AmsbergGunhild^1^1University Medical Center Hamburg-Eppendorf, 20251 Hamburg, Germany2G.B. Elyakov Pacific Institute of Bioorganic Chemistry, School of Natural Sciences, Far Eastern Federal University, 690022 Vladivostok, Russia

Marine natural compound Ascididemine, a pentacyclic aromatic alkaloid isolated from ascidiae *Didemnum* sp., exhibits promising activity in different models of human cancer in vitro. Thus, we have synthesized 24 derivatives of Ascididemine in order to improve its anticancer properties. The consequent screening revealed two most promising derivatives to be more active in human drug-resistant prostate cancer (PCa) cells in comparison with non-cancer cells in vitro. The further investigation of these derivatives revealed its primary ability to target mitochondria of cancer cells, to induce ROS production as well as mitochondrial membrane depolarization, followed by caspase-9 and -3 mediated apoptosis. When applied in combination with the PARP-inhibitor Olaparib both investigated Ascididemine derivatives exhibited strong synergism in BRCA1-deficient prostate cancer cells. Analysis of the kinome and proteome suggested an up-regulation in katalytic acitivity of several kinases, specifically involved in the PI3K-Akt-pathway as well as the PIM1 kinase. In line with it the combinational treatment with specific Akt-inhibitors showed highly synergistic effect. Moreover, a moderate to high synergism in drug-resistant PCa cells has been shown for the investigated substances when combined with such well-established anticancer drugs as Abiraterone, Enzalutamide, Cisplatin, Carboplatin and Docetaxel.

In conclusion, we were able to identify two Ascididemine derivatives with potent anticancer activity alone as well as in combination with established drugs. The suggested mechanism of action includes mitochondria targeting, ROS induction and caspase-dependent apoptosis. Further in vitro and in vivo examinations are ongoing.

#### **Phlorotannins from *Fucus vesiculosus* and Their Potential Benefits towards the Gastrointestinal Tract** 

CatarinoMarcelo D.^1^FernandesIva^2^SilvaArtur M. S.^1^MateusNuno^2^CardosoSusana M.^1^1QOPNA & LAQV-REQUIMTE, Department of Chemistry, University of Aveiro, 3810-193 Aveiro, Portugal2REQUIMTE/LAQV, Department of Chemistry and Biochemistry, Faculty of Sciences, University of Porto, 4169-007 Porto, Portugal

According to WHO, worldwide obesity has nearly tripled since 1975 and diabetes almost quadrupled from 1980 to 2014 [1,2]. In addition, colon and stomach cancers were the 2nd and 3th most lethal causes of cancer deaths in 2018 [3]. In this context, phlorotannins, i.e., phenolic compounds characteristic from brown algae, have drawn much attention in the recent years due to their potential health benefits which include anti-obesity, anti-diabetes and antitumor effects [4]. In this work, the phlorotannin profile of a 70% acetone extract *F. vesiculosus* and its subsequent purified fractions were analyzed through UHPLC-DAD-ESI-MS^n^ analysis revealing a high variability in terms of polymerization degree (up to 22 units). Samples were further evaluated for their capacity to inhibit key enzymes from lipid or carbohydrate metabolism, showing good inhibitory activity against pancreatic lipase, α-amylase and especially α-glucosidase in which inhibitory effects were seen to be even pronounced than that of the pharmaceutical drug acarbose. Moreover, promising cytotoxic activity was exhibited against tumor cell lines from the intestinal tract, namely towards MKN-28 (stomach adenocarcinoma cells), HT-29 and Caco-2 (both cell lines of colon colorectal adenocarcinoma). Overall, this study provides evidence that *F. vesiculosus* phlorotannin rich extracts and subsequent purified fractions not only hold potential for weight and glycemia management, but also may prevent the proliferation certain cancerous cells in the intestinal tract, suggesting that the exploitation of these compounds for application with nutraceutical and even pharmaceutical purposes might be a viable approach towards the prevention of these problematics.

##### **Acknowledgments** 

Authors acknowledge the financial support of the project AlgaPhlor (PTDC/BAA-AGR/31015/2017), financed by National funds thought FCT. We also thank University of Aveiro, FCT/MEC for financial supporting QOPNA research Unit (FCT UID/QUI/00062/2019), through national funds and where applicable co-financed by the FEDER, within the PT2020 Partnership Agreement. Susana Cardoso thanks the research contract under the project AgroForWealth (CENTRO-01-0145-FEDER-000001), funded by Centro2020, through FEDER and PT2020. Marcelo D. Catarino acknowledges FCT for financial support (fellowship PD/BD/114577/2016).

References

1. WHO. Obesity and Overweight Fact Sheet. Available online: https://www.who.int/news-room/fact-sheets/detail/obesity-and-overweight (accessed on 26 May 2019).
2. WHO. Diabetes Fact Sheet. Available online: https://www.who.int/news-room/fact-sheets/detail/diabetes (accessed on 26 May 2019).
3. WHO. Cancer Fact Sheet. Available online: https://www.who.int/news-room/fact-sheets/detail/cancer (accessed on 26 May 2019).
4. Brown, E.M.; Allsopp, P.J.; Magee, P.J.; Gill, C.I.; Nitecki, S.; Strain, C.R.; Mcsorley, E.M. Seaweed and human healt. *Nutr. Rev.*
**2014**, *72*, 205–216.

#### **Portoamides A and B isolated from *Phormidium* sp. LEGE 05292 Induces Cytotoxicity on the Proliferative Cell Layer of In Vitro Microtumours** 

SousaM. Lígia^1^,^2^RibeiroTiago^1^,^2^VasconcelosVítor^1^,^2^LinderStig^3^,^4^UrbatzkaRalph^1^1CIIMAR—Interdisciplinary Centre of Marine and Environmental Research, 4450-208 Matosinhos, Portugal2FCUP—Faculty of Sciences of University of Porto, 4169-007 Porto, Portugal3Department of Oncology and Pathology, Cancer Centre Karolinska, Karolinska Institute, SE-171 76 Stockholm, Sweden4Department of Medical and Health Sciences, Linköping University, SE-581 83 Linköping, Sweden

Cyanobacteria are recognized as producer of natural products that in some case may have biotechnological applications. Often, these compounds are isolated from symbiotic or filamentous cyanobacteria. Portoamides A and B (**PAB**) were isolated from the strain *Phormidium* sp. LEGE 05929, occur naturally at the proportion of 3:1 and have allelopathic effects on the microalgae *Chlorella vulgaris* and cytotoxic effects against several cell lines. **PAB** were tested against the cancer cell line HCT116 cultured both in monolayer (2D) and as multicellular system (3D), and its penetration on the structure and consequential effects were analysed. The 2D approach revealed that **PAB** enters into cells on energy-independent mechanism, while fluorescent analyses on spheroids showed only cytotoxic effects against the outer, proliferative cell layers. Mitochondrial hyperpolarization was observed, a decrease of ATP content and disruption of OXPHOS with a significant decrease of maximal mitochondrial respiration. The overexpression of PINK1 additionally indicated that disruption of mitochondrial function is the underlying mechanism of cytoxicity. Targeting mitochondria was recently described as a particularly interesting strategy for cancer treatment, and **PAB** reveal specificity on this target, penetrating the outer layer of spheroids.

##### **Acknowledgments** 

This research was supported by the funding of RD units strategic plan UID/Multi/04423/2019 and the project CYANCAN (reference PTDC/MEDQUI/30944/2017) co-financed by NORTE 2020, Portugal 2020 and the European Union through the ERDF, and by FCT through national funds. R. Urbatzka was supported by the FCT postdoc grant SFRH/BPD/112287/2015 and M. L. Sousa by the FCT PhD grant SFRH/BD/108314/2015.

#### **Prodiginines as Antimelanoma Agents: Mechanism of Action, Chemoresistance Overcoming and Target Determination** 

BrancoPaola C.^1^AraújoCristine P.^1^Rezende-TeixeiraPaula^1^BauermeisterAnelize^1^AlvesDébora K.^2^Maria-EnglerSilvya S.^2^JimenezPaula C^3^Machado-NetoJoao A.^1^LopesNorberto P.^4^Costa-LotufoLeticia V.^1^1Department of Pharmacology, Institute of Biomedical Science, University of São Paulo, Av. Prof. Lineu Prestes, 1524, Sao Paulo, SP 05508-000, Brazil2School of Pharmacy, University of Sao Paulo, Sao Paulo 05508-00, Brazil3Federal University of Sao Paulo, Sao Paulo 11070-100, Brazil4School of Pharmaceutical Sciences of Ribeirão Preto, University of Sao Paulo, Sao Paulo 14040-903, Brazil

Prodiginines are family of red tripyrrole pigments with potent cytotoxic activity. We isolated prodigiosin (PG) and the derivatives cyclononylprodigiosine (CNP) and nonylprodigiosine (NP) from Brazilian marine bacteria. PG, CNP and NP were selective for melanoma, with IC_50_ values ranging from 0.02 μM for SKMel-19 and SKMel-28 (both with mutation BRAF^V600E^) to 0.52 μM for the SKMel-147 (mutation NRAS) lineage. Substances decreased cell viability, without affecting dead cells percentage after 24 h treatment, suggesting low doses promote cytostatic effects. In addition, a decrease in the clonogenicity of the SKMel-19 and SKMel-28 lines after treatment with PG and derivatives at sub-toxic doses was observed. Besides the induction of apoptosis as evidenced by increased cleaved caspase-3 and DNA damage induction as evidenced through increased histone H2AX in SKMel-19 cell line, treatment with PG in the SKMel-19 and SKMel-28 lines seems to negatively modulate the expression of survivin at the gene level. Additionally, PG and CNP could overcome chemoresistance to vemurafenibe in SKMel-19R but not in SKMel-28R, evidenced by IC_50_ of 0.06 μM for PG and 0.08 μM for CNP in SKMel-19R and decreased clonogenic capacity, similar to its naive counterpart. Additionally, PG treatment supressed mRNA survivin levels in SKMel-19R, but not in SKMel-28R. To investigate the role of survivin as the target, a bioaffinity chromatography was used and revealed that PG binds to survivin, what was confirmed by Microscale-Thermoforesis with a Kd of 2.2 µM. Altogether, our results demonstrated that PG and derivatives are selective to melanoma, inducing apoptosis, DNA damage and targeting and modulating survivin.

##### **Acknowledgments** 

FAPESP (2017/09022-8 and 2015/17177-6).

#### **Developing a Drug Discovery Platform Applied to Marine Natural Products** 

Moreiras-FiguerueloAlejandro^1^NuzzoGenoveffa^1^SansoneClementina^2^GalassoChristian^2^AndersenJeanette H.^3^FontanaAngelo^1^1Bio-Organic Chemistry Unit, Institute of Biomolec. Chemistry—CNR, Via Campi Flegrei 34, Pozzuoli, 80078 Naples, Italy2Marine Biotechnology Lab., Integrative Marine Ecology Dept., Stazione Zoologica Anton Dohrn, Villa Comunale, 80121 Naples, Italy3Marbio, Faculty of Biosciences, Fisheries and Economics, UiT—The Arctic University of Norway, NO-9037 Tromsø, Norway

The aim of this research project is to identify novel bioactive compounds from a variety of marine organisms (protists and invertebrates) by the development and use of an innovative platform of drug discovery. For both, finding novel bioactive compounds and testing the efficiency of the innovative platform, a drug screening is performed using about 55 different marine samples. The key element of the drug discovery platform consists in the fractionation of the crude extracts using a solid phase extraction procedure. The molecules are separated in five fractions of decreasing polarity in such way that the large salt quantities from marine organisms remain isolated from the rest of organic compounds. Moreover, the compounds in the other fractions become very concentrated and can be tested with higher sensitivity. The crude extracts together with their fractions are next tested for identifying biologic activities. The biologic activities of interest in this project are cytotoxicity (MTT assay after 48 h), antibiotic activity (growth inhibition after 20 h) and inhibition of the PTP1B enzyme, involved in Type 2 Diabetes, but multiple other tests can be easily added for detecting other interesting biologic activities. The data obtained from extractions and fractionations, together with the analysis of selected tests, will be presented in the poster of the conference. 

**Acknowledgements:** This research was funded by the European Commission H2020-MSCA-ITN-ETN MarPipe, grant agreement number 721421.

#### **Seriniquinone, Metabolite from a Marine Bacterium, and its Synthetic Derivative for Melanoma and Resistant Melanoma Cells Treatment** 

HirataAmanda S.^1^BrancoPaola C.^1^Rezende-TeixeiraPaula^1^JimenezPaula C.^2^La ClairJames J.^3^FenicalWilliam^3^Costa-LotufoLetícia V.^1^1Institute of Biomedical Sciences, University of Sao Paulo, São Paulo 05508-000, Brazil2Federal University of São Paulo, São Paulo 11015-020, Brazil3University of California, San Diego, CA 92093-0204, USA

Seriniquinone (SQ) is a secondary metabolite isolated from *Serinicoccus* sp. that regulates the expression of dermcidin gene (DCD) and induces cell death by autophagy selectively in melanoma cell lines. Dermicidin protein role in neoplasias remains unknown, but studies are showing it as a new oncogene candidate. LT406, a synthetic analog of SQ, was designed in order to optimize SQ properties. To characterize the toxicity and mechanism of action of SQ and LT406, SK-MEL-28, SK-MEL-147 and SK-MEL-28R melanoma cell lines (BRAFV600E mutant, NRAS mutant and BRAFV600E mutant resistant to vemurafenib drug, respectively) were used. LT406 was slightly more active than SQ after 24 and 48 h treatment as observed by IC_50_ values obtained through MTT assay. However, LT406 had similar toxicity in all lines after 72 h (0.09 µM and 0.04 µM in SK-MEL-28, 0.05 µM and 0.1 µM in SK-MEL-147 and 0.8 µM and 0.7 µM in SK-MEL-28R, for SQ and LT406, respectively). In the Trypan blue assay using SK-MEL-28 and SK-MEL-147, LT406 treatment had more non-viable cells, while SQ was more potent in the clonogenic assay. Continuing with these lines, DCD expression was modulated in SK-MEL-28 for both compounds but not in the SK-Mel-147. DCD and death proteins expressions were different between cell lines and treatments, suggesting death by autophagy for SQ and apoptosis for LT406. Considering all together, SQ seems to present a cytostatic effect in contrast to the cytotoxic effect from LT406. Analyzes of RNA and protein expressions reinforced that both compounds have different mechanisms of action.

##### **Acknowledgments** 

Financial support from FAPESP (2015/17177-6 and 2018/07661-6).

#### **Fishing Marine Natural Products Using TBX2 Trancription Factor as Bait** 

SahmBianca Del B.^1^SantosEvelyne A.^1^BrancoPaola C.^1^BauermeisterAnelize^2^MoreiraEduarda Antunes^2^JimenezPaula C.^3^LopesNorberto P.^2^PrinceSharon^4^Costa-LotufoLeticia V.^1^1Institute of Biomedical Sciences, University of São Paulo, São Paul, 05508-000, Brazil2School of Pharmaceutical Sciences, University of São Paulo, Ribeirão Preto, 14040-903 Brazil3Federal University of São Paulo, Santos 11070-100, Brazil4Division of Cell Biology, Department of Human Biology, University of Cape Town, Cape Town 7925, South Africa

TBX2 is a transcriptional factor with important roles in carcinogenesis and its modulation figure as a possible new anticancer therapy. Herein we aim the isolation and characterization of marine bacteria compounds with binding affinities to TBX2 recombinant protein. Using a reverse affinity procedure we were able to “fish” three classes of substances: staurosporins, surugamides and chromomycins. Chromomycin A5 (C-A5), a minor groove DNA binder molecule produced by Streptomyces bacteria, was chosen to proceed with studies. The binding affinity between C-A5 and TBX2 was assessed through microscale thermophoresis and compared with others DNA binding natural compounds. The results suggest a stronger binding affinity for C-A5 and TBX2 (KD: 41.3 μM). Biological activity assays were developed using the melanoma cell line 501mel, whereas TBX2 functions were previously characterized (Peres et al., 2010). C-A5 displayed high cytotoxicity (IC_50_ 0.9 nM) against 501mel and showed dose-dependent DNA-damaging properties. Interestingly, the investigation in the TBX2 signaling pathways revealed an alteration for TBX2 target molecules in cells treated with C-A5 at lower concentrations (0.5 and 1 nM).

Moreover, we checked for mRNA levels and found a significantly up-regulation for TBX2 as well as for its target genes. In this work we discuss the validation of TBX2 as “bait” for marine compounds as well as the borders between DNA-damage and transcriptional regulation possibly performed by C-A5.

##### **Acknowledgments** 

FAPESP (2015/17177-6 and 2014/50926-0), CNPQ (PROAFRICA: 440232/2015-5; PROSPECMAR: 458548/2013-8), CAPES (47/2017) and NRF.

#### **Use of Bioaffinity Chromatography in the Prospection of Anticancer Substances in Marine Actinomycetes: XIAP and STMN1 as Pharmacological Targets in Cancer** 

Reis e SilvaCatarina S. M.^1^BauermeisterAnelize^1^,^2^MoreiraEduarda A.^2^LopesNorberto P.^2^TangerinaMarcelo M. P.^3^FerreiraMarcelo J. P.^3^Machado-NetoJoão Agostinho^1^Costa-LotufoLeticia V.^1^1Institute of Biomedical Sciences, University of Sao Paulo, Sao Paulo 05508-900, Brazil2School of Pharmaceutical Sciences of Ribeirão Preto, University of Sao Paulo, Ribeirao Preto 14040-903, Brazil3Institute of Biosciences, University of Sao Paulo, Sao Paulo 05508-090, Brazil

Marine environment is an exceptional warehouse for new bioactive natural products, including those with anticancer properties. A major obstacle in pharmacology is the elucidation of the pharmacological target, which may be overcome by directed target screening in order to reduce costs and maximize time. XIAP and STMN1 proteins are interesting targets for anticancer therapy. XIAP is an Apoptosis Inhibitor Protein (IAPs), which are involved cell death prevention and resistance to current anticancer treatment by direct binding and inhibition of caspases. STMN1 is a microtubule dynamics regulator that promotes cell cycle progression and clonogenicity. Overexpression of STMN1 and XIAP are associated with a variety of cancers in humans. Bioaffinity chromatography is a novel technique and can be used to find biological affinity between proteins of interest and extracts which exhibit cytotoxicity. In the present study, we established the protocols for XIAP and STMN1 heterologous expression. For both proteins, overnight growth at 16 °C in *E. coli* BL21 (DE3) were suitable (XIAP: 0.3 mM IPTG and STMN1: 1 mM IPTG). After that, the Bioaffinity Assay (HPLC-MS) will be performed in a library of extracts of marine bacteria that presented cytotoxic activity. In this first assay 15 extracts of marine bacteria were grown in different culture media, three Hits were obtained which were classified as nonspecific because they were bound to both proteins, however it was demonstrated that the technique works and more tests are being performed. In summary, we standardized the expression of interesting anticancer targets to identify new pharmacological agents for cancer treatment.

##### **Acknowledgments** 

Financial Support from CAPES, CNPq and Fapesp (2018/06522-2; 2015/ 17177-6; 2017/16606-6; 2017/17648-4).

#### ***Llayta*: The Edible Cyanobacteria Colonies of *Nostoc* from Chile Significantly Reduce Neutral Lipid Content** 

LopesGraciliana^1^,^2^PretoMarco^2^GaletovicAlexandra^3^Gómez-SilvaBenito^3^MartinsJoana^1^,^2^UrbatzkaRalph^2^VasconcelosVitor^1^,^2^1FCUP, Faculty of Sciences, University of Porto, Rua do Campo Alegre, 4169-007 Porto, Portugal2CIIMAR/CIMAR, Interdisciplinary Centre of Marine and Environmental Research, Novo Edifício do Terminal de Cruzeiros do Porto de Leixões, Avenida General Norton de Matos, S/N, 4450-208 Matosinhos, Portugal3Laboratory of Biochemistry, Biomedical Department, Faculty of Health Sciences, and Centre for Biotechnology and Bioengineering, University of Antofagasta, CeBiB, Avenida Angamos 601, Antofagasta 1240000, Chile

**Figure 41:**
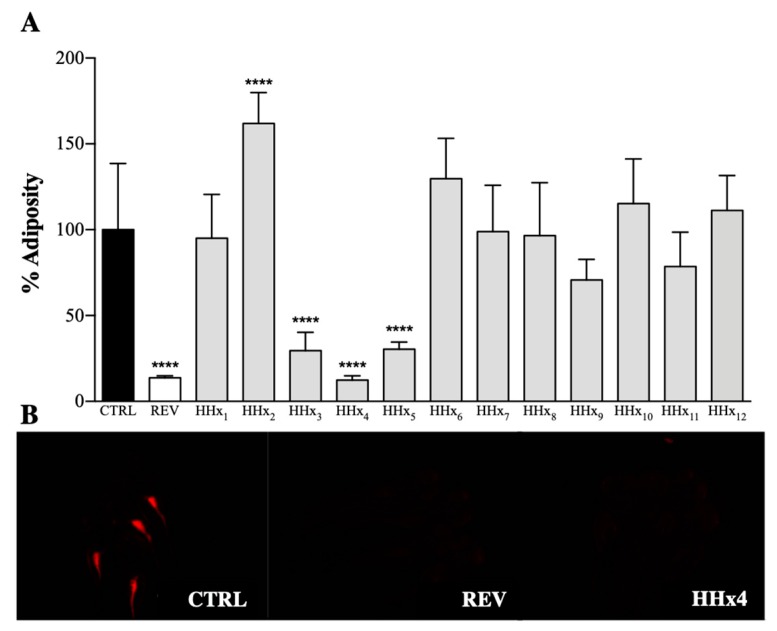
Percentage of Nile red fluorescence in zebrafish larvae after 48h exposure to Nostoc Llayta sub-fractions HHx1-HHx12. Resveratrol (REV) was used as positive control, and CTRL is the solvent control (0.1% DMSO) (**A**), and Zebrafish larvae view under fluorescence microscopy (**B**). Error bars represent the mean ± SD. Statistical significance at **** *p* < 0.0001 (One way ANOVA analysis of variance).

*Llayta* has been part of the Andean feeding practices of the rural communities of Perú and Chile since the pre-Columbian days. It consists in colonies of cyanobacteria of the genus *Nostoc*, which are harvested, sun-dried, and sold in the market as ingredient for human consumption. Although it is known for its substantial content in essential amino acids and polyunsaturated fatty acids, little is known about *Llayta*, with regard to its pharmacological and biotechnological potential. In order to uncover the pharmacological and nutraceutical potential of *Llayta*, we have explored its lipid-reducing capacity, through a bioassay-guided isolation, using the zebrafish Nile red fat metabolism assay. The dry biomass of *Llayta* was extracted by repeated percolation with a warm mixture of CH_2_Cl_2_/MeOH (2:1, *v/v*), yielding a crude extract of 0.58 g. The crude extract was then fractionated by normal-phase (Si gel 60, 0.015–0.040 mm) vacuum liquid chromatography with an increasing polarity grade, from 90% *n*-hexane to 100% ethyl acetate and 100% methanol, giving a total of 10 fractions. The most active fractions (H and Hx) were combined, due to their similarity, and further sub-fractioned into 12 new sub-fractions (HHx1-HHx12), by column chromatography, and monitored by thin layer chromatography. The most active fraction, HHx4 (Figure 41), reduced the neutral lipid content by approx. 80%, and is now being subjected to further chemical characterization, in order to find the compound responsible for the biological activity.

##### **Acknowledgments** 

This work was done in the framework of the project BLUEHUMAN–BLUE biotechnology as a road for innovation on HUMAN’s health aiming smart growth in Atlantic Area–EAPA_151/2016 of the Interreg Atlantic Area Programme funded by the European Regional Development Fund. This work was supported by the FCT Project UID/Multi/04423/2019.

#### **PM14: A New Antitumor Compound in Clinical Studies in Patients with Solid Tumors** 

RodríguezRaquelMartínezValentínFranceschAndrésGuillénMaría JoséMuntSimonAvilésPabloCuevasCarmenResearch and Development, PharmaMar, S.A.; Avda. de los Reyes 1, Pol. In. La Mina, 28770 Colmenar Viejo, Madrid, Spain

PM14, a new antitumor chemical entity from PharmaMar’s internal research activities, has demonstrated activity in solid tumors in animal models. Following preclinical development, a Phase I clinical study started in September 2017, with the primary objective of identifying the optimal dose for the administration of PM14 in patients with advanced solid tumors. Secondary objectives of this initial clinical trial are to assess the safety profile and to evaluate PM14 pharmacokinetics and pharmacogenomics in patients.

This Phase I clinical study with PM14 is expected to include approximately 50 patients with advanced solid tumors.

#### **Investigation of HDAC and 20S Proteasome Inhibitors from Cyanobacteria** 

MartinsJoana^1^,^2^CamposAlexandre^2^LeãoPedro N.^2^VasconcelosVitor^1^,^2^1Faculty of Sciences, University of Porto, Rua do Campo Alegre, 4169-007 Porto, Portugal2Interdisciplinary Center of Marine and Environmental Research (CIIMAR/CIMAR), University of Porto, Terminal de Cruzeiros do Porto de Leixões, Avenida General Norton de Matos, S/N, 4450-208 Matosinhos, Portugal

Cancer is one of the leading causes of dead worldwide and continuous efforts to find effective anti-cancer drugs are of crucial importance. Cyanobacteria are a rich source of bioactive molecules, with several reported activities including anti-cancer. The histone deacetylase enzymes (HDAC) and the 20S proteasome are two molecular targets that have attracted research attention for their potential, especially in cancer treatment. Our group maintains a large cyanobacterial culture collection—LEGE Culture Collection (LEGE CC)—with strains isolated from marine, estuarine and freshwater environments that are representatives of several cyanobacterial orders. In this study, crude extracts from 36 cyanobacterial strains from the LEGE CC were tested against the HDAC and the 20S proteasome activities. Several extracts possessed significant levels of HDAC and 20S proteasome inhibition, which prompted us to select one of the more active strains for large-scale growth with the aim of isolating their active(s) constituent(s). As a result, the bioassay-guided fractionation of the bioactive compounds from a *Nodosilinea nodulosa* strain lead us to identify two active fractions against the 20S proteasome chymotrypsin-, trypsin- and caspase-like activities. The chemical analysis of these fractions allowed us to identify that their main constituents are polyunsaturated fatty acids (PUFAs) but also contain a series of unknown halogenated molecules.

##### **Acknowledgments** 

FCT Project UID/Multi/04423/2019; INTERREG Atlantic Area European programme EnhanceMicroalgae project EAPA_338/2016.

#### **Antibacterial Activity of Organic Extracts of Gorgonian *Eunicella cavolini* from the Adriatic Sea in Croatia** 

BojanićKrunoslav^1^,^2^PavelićSandra Kraljević^2^,^3^MarkovićDean^2^,^3^MatuljaDario^2^,^3^GrbčićPetra^2^,^3^PopovićNatalija Topić^1^,^2^Strunjak-PerovićIvančica^1^,^2^Čož-RakovacRozelindra^1^,^2^1Laboratory for Biotechnology in Aquaculture, Division of Materials Chemistry, Ruđer Bošković Institute, Zagreb 10000, Croatia2Centre of Excellence for Marine Bioprospecting BioProCro, Zagreb 10000, Croatia3Department of Biotechnology, Centre for High-Throughput Technologies, University of Rijeka, Rijeka 51000, Croatia

*Eunicella cavolini* is an abundant gorgonian species in the Mediterranean Sea and previous studies in Greece showed its organic extracts to be rich in complex steroids (pregnanes, epidioxysterols, and secosterols) that showed antiproliferative activity against various human cancer cell lines. This study evaluated antibacterial activity against three human pathogens *Escherichia coli* (EC), *Pseudomonas aeruginosa* (PA), and *Staphylococcus aureus* (SA). Sampling was performed in May 2018 at 23 m depth. Specimens were extracted using CH_2_Cl_2_/MeOH (3:1) at room temperature, dried in vacuo (OE) with further 4 fractions obtained with c-hexane:EtOAc (2 fractions with 75:25 (F1) and 50:50 (F2) ratio, respectively), EtOAc (F3), and EtOAc:MeOH (80:20 (F4)), all dissolved in DMSO. The highest tested concentrations were 2500 μg/mL for all extracts and 1000 μg/mL for F1. The antibacterial activity was evaluated by broth microdilution assay according to the Clinical & Laboratory Standards Institute guidelines with results interpreted both visually and spectrophotometrically. The results showed partial inhibition of all three bacterial species by the OE with the highest activity against SA which was an order of magnitude higher than with EC and PA. Fractionated samples F1–F3 had higher antibacterial activity (in decreasing order F2, F3, F1) than the OE with SA being the most inhibited followed by EC, and PA. Minimum inhibitory concentration (MIC) was observed only for SA with F2 and F3 at the highest tested concentration. Cultivation of SA at MIC showed the fractions with bacteriostatic rather than bactericidal activity, with F2 having the strongest activity according to number of colonies isolated.

#### **Microbial Diversity and Biomedical Potential of Marine Bacteria Associated with the Neptune Cup Sponge, *Cliona patera*, using Culture Dependant and Culture Independant Methods** 

KatermeranNursheena Parveen^1^HoXin Yi^2^DeignanLindsey Kane^2^OngJi Fa Marshall^1^TunKarenne^3^TanLik Tong^1^1Natural Sciences and Science Education, National Institute of Education, Nanyang Technological University, 1 Nanyang Walk, Singapore 637616, Singapore2Singapore Centre for Environmental Life Sciences Engineering, Nanyang Technological University, 60 Nanyang Drive, Singapore 637551, Singapore3National Parks Board, 1 Cluny Road, Singapore Botanic Gardens, Singapore 259569, Singapore

Marine invertebrates, such as corals and marine sponges, are found to host a complex microbial consortium. Studies have shown that such microbes are essential to the health and resilience of these invertebrates. The Neptune cup sponge, *Cliona patera*, was recently rediscovered in Singapore waters after being last sighted in the 1870s. In this study, marine bacteria associated with *C. patera* were isolated and identified using culture dependent and culture independent methods. Marine bacterial strains, isolated using culture dependent methods based on five actinomycete-selective marine culture, were analyzed for their quorum sensing inhibitory activity using Pseudomonas aeruginosa reporter strains. In addition, selected bacterial strains were further screened for brine shrimp (*Artemia salina*) toxicity as well as anticancer activity based on the MOLT-4 cancer cell line. MS-based metabolomics approach based on the molecular networking platform was used for compound dereplication as well as detection of new metabolites in selected bacterial strains that showed significant activity. Furthermore, preliminary data on the bacterial diversity of the sponge based on amplicon pyrosequencing will be presented. The microbial data obtained from this study can be used as baseline data for future ecological studies on sponge resilience as well as assessing the biomedical potential of *C. patera*-associated microbes.

#### **Cytotoxic Activity of the Proteasome Inhibitors Produced from Marine *Streptomyces* sp. in Glioma Cells** 

FurtadoLuciana Costa^1^BauermeisterAnelize^1^JimenezPaula Christine^2^NetoJoão Agostinho Machado^1^FelícioRafael de^3^TrivellaDaniela Baretto Barbosa^3^LopesNorberto P.^4^LotufoLeticia Veras Costa^1^1Institute of Biomedical Sciences, University of São Paulo, São Paulo 05508-000, Brazil2Federal University of São Paulo, Santos 11070-100, Brazil3Brazilian Biosciences National Laboratory, National Center for Research in Energy and Material, Campinas 13083-970, Brazil4School of Pharmaceutical Sciences, University of São Paulo, Ribeirão Preto 14040-903, Brazil

Marine natural products emerge as a source of inestimable pharmacological potential. In this perspective, the sustainable supply of bioactive metabolites from the cultivation of marine microorganisms appears as an attractive solution for the rational exploration of these substances. A target assay showed high cytotoxic potential of the BRA-346 extract that inhibited the catalytic activity of the ChTL subunit of the proteasome. *Streptomyces* sp. (BRA-346) isolated from the Brazilian endemic ascidian, *Euherdmania* sp., was shown to be a promising source of cytotoxic substances. This strain produced an extract with significant cytotoxic activity, IC_50_ of 30 ng/mL in human colon carcinoma cells, HCT-116. The enzymatic assay using purified *S. cerevisiae* proteasome showed an inhibition with an average concentration of 0.42 μg/mL for the extract and 0.04 μg/mL for its enriched fraction (BRA-346ADC). The crystallographic assays detected the presence of a compound belonging to the epoxy-ketone class bounded to the catalytic subunit ChTL. Mass spectrometric analysis identified the TMC-86A ion and dihydroeponemycin in the fraction. Proteasome inhibitors are used clinically in the treatment of multiple myeloma, but they have been tested with promising results in other types of cancers, including glioblastomas. Tests with the enriched fraction BRA-346 exhibited high cytotoxicity in the cell lines HOG and T98G, IC_50_ = 25.4 and 37.3 ng/mL, respectively. Molecular analysis showed results related to the proteasome pathway and reticulum stress induction by the BRA-346ADC fraction. Together these results reinforce the importance of the compounds present in the BRA-346 strain in glioblastoma models.

##### **Acknowledgments** 

Financial support from FAPESP (2017/18235-5; 2015/17177-6), CNPq, CAPES.

#### **Antiviral Activity of *Laurencia* Metabolites against Chikungunya Virus** 

WankeTauana^1^PhilippusAna Claudia^1^LhullierCintia^1^PaixãoIzabel^2^FalkenbergMiriam^1^IoannouEfstathia^3^RoussisVassilios^3^1Postgraduation Program in Pharmacy, Federal University of Santa Catarina, Florianopolis, SC 88040-900, Brazil2Federal Fluminense University, Niteroi, RJ 24220-900, Brazil3Section of Pharmacognosy and Chemistry of Natural Products, Department of Pharmacy, National and Kapodistrian University of Athens, 15771 Athens, Greece

The mosquito-borne Chikungunya virus (CHIKV) that has emerged in Western countries in the recent years causes fever, besides the severe back and joint pain that may persist for months or years. No treatment or vaccine is available at the moment, so effective drugs are urgently needed. Since halogenated sesquiterpenes exhibit a wide spectrum biological activities we have decided to submit a panel of halogenated metabolites isolated from the red alga *Laurencia catarinensis* to evaluation of their antiviral activity against CHIKV. Specimens of *L. catarinensis* were collected from Xavier Island, Santa Catarina, Brazil, and following a series of chromatographic separations resulted in the isolation of a number of metabolites. Antiviral activity was evaluated with VERO cells infected with CHIKV. Among the tested halogenated metabolites, johnstonol and prepacifenol epoxide presented EC_50_ of 0.65 and 1.25 μM, respectively and are currently being submitted to further evaluations aiming to find an effective and safe treatment. To the best of our knowledge, this is the first report of marine compounds active against CHIKV.

#### **Preliminary Evidence of Bioactivity from Deep Sea Coral and Sponge Extracts: Influencing Human Stem Cell Growth and Differentiation** 

MarchesePietro^1^YoungRyan^2^O’ConnellEnda^3^BakerBill J.^4^JohnsonMark^2^FearnheadHoward^3^AllcockLouise^2^MurphyMary^1^1Regenerative Medicine Institute, National University of Ireland, H91W2TY Galway, Ireland2Ryan Institute, National University of Ireland, H91TK33 Galway, Ireland3Screening Core Facility, National University of Ireland, H91W2TY Galway, Ireland4Chemistry Department, University of South Florida, FL 33620, USA

Extensive areas of our Planet are covered by deep sea, constituting the major environment existing on Earth and a valuable reservoir of bioactive compounds. Musculoskeletal diseases such as osteoarthritis and osteoporosis could benefit from the discovery of new drugs to induce tissue regeneration through control of stem cell fate. In the sea, corals and sponges can build calcified structures with similarities to human bone and may have potential for the discovery of metabolites to use in regenerative medicine.

We investigated the ability of deep-sea corals and sponges extracts to induce osteogenic differentiation of Mesenchymal Stem Cells (MSCs) using high throughput screening technology. A library of 160 raw extracts was tested on MSCs seeded in 96-well plates and treated with 0.15 mg/mL extracts over 10 days. Cell differentiation was measured as calcium mineralized in the extracellular matrix after treatment while the cell number was obtained after nuclear staining. The screening was performed using Janus automated liquid handling and Operetta high content imaging systems (Perkin Elmer). From this screening, five extracts showed significant differentiation induction of MSCs compared to the control and other four extracts showed proliferative effect inducing an increase up to 30% of cell number. Nine negative hits were also detected as cytotoxic extracts killing up to 96% of the originally seeded cells.

To the best of our knowledge this is the first screening done on MSCs investigating prodifferentiation factors produced by deep sea benthic organisms. Further analyses on extract fractions and purified molecules are ongoing.

#### **Neuroprotective Effects of Two Fijian Compounds against Oxidative Stress** 

AlvariñoRebeca^1^AlonsoEva^1^,^2^TabudravuJioji^3^,^4^GegundeSandra^1^JasparsMarcel^4^AlfonsoAmparo^1^BotanaLuis M.^1^1Departamento de Farmacología, Facultad de Veterinaria, Universidad de Santiago de Compostela, 27003 Lugo, Spain2Fundación Instituto de Investigación Sanitario Santiago de Compostela (FIDIS), Hospital Universitario Lucus Augusti, 27003 Lugo, Spain3School of Forensic and Applied Sciences, Faculty of Science & Technology, University of Central Lancashire, Preston PR1 2HE, Lancashire, UK4Marine Biodiscovery Centre, Department of Chemistry, University of Aberdeen, Aberdeen AB24 3UE, Scotland, UK

The Fiji Islands, in the Central Pacific, harbour a great biodiversity. Because of this, Fiji’s marine flora and fauna have been subjected to an intense research in the field of natural products. More than 400 compounds have been isolated from Fijian organisms, being sponges, ascidians and corals the major sources of bioactive products. In this study, the neuroprotective abilities of two compounds obtained from Fijian organisms were evaluated. Tavarua deoxyriboside A was obtained from a tunicate collected in the island of Tavarua. On the other hand, the cyclic depsipeptide jasplakinolide was isolated from the sponge *Jaspis splendens*, collected from Suva Harbour, in Viti Levu Island. Tavarua deoxyriboside A biological activity has not been tested so far, whereas jasplakinolide has shown anticancer activity through the promotion of actin polymerization. The capacity of compounds to protect cells from oxidative stress was determined in the cell line SH-SY5Y. With this purpose, human neuroblastoma cells were co- treated for 6 h with 150 μM H_2_O_2_ and compounds at non-toxic concentrations. Both compounds improved cell survival, recovered mitochondrial membrane potential and produced a decrease in reactive oxygen species release. In addition, tavarua deoxyriboside A and jasplakinolide increased the amount of glutathione in cells. These neuroprotective effects were mediated by the ability of compounds to activate the transcription factor Nrf2. Its expression levels were determined by western blot and a significant increase was found in the nuclear fraction. In summary, our results suggest a Nrf2-mediated neuroprotective effect of both compounds.

#### **Modulation of Cyclophilins with *Spongionella*-Derived Compounds** 

GegundeSandra^1^AlfonsoAmparo^1^AlonsoEva^1^AlvariñoRebeca^1^JuanateyCarlos González^2^BotanaLuis M.^1^1Departamento Farmacología, Facultad Veterinaria, Universidad Santiago de Compostela, 27002 Lugo, Spain2Servicio de Cardiología, Hospital Universitario Lucus Augusti, 27004 Lugo, Spain

Natural gracilins, isolated from the sponge *Spongionella gracilis*, have shown antioxidant, neuroprotective, anti-inflammatory and immunosuppressive properties and some of them were reported as cyclophilins (Cyps) modulators. Cyps are a subgroup of proteins called immunophilins, with peptidyl prolyl *cis-trans* isomerase (PPIase) activity which possesses high affinity to the immunosuppressant cyclosporine A (CsA). These proteins have been involved in several cellular processes such as immune response, apoptosis and oxidative stress. Nevertheless, the physiological relevance of Cyps family remains unclear. From the gracilins group, gracilin A (GraA) was described to have neuroprotective and immunosuppressant activity by binding CypA and CypD. For this reason, natural GraA was used to synthesize small compounds by pharmacophoredirected retrosynthesis. These synthetic GraA-analogs showed specific immunosuppressive or anti-inflammatory ability by selectively joining CypA or CypD. In this context, the aim of this work was to study the modulation of Cyps profile in activated T lymphocytes by gracilin L (GraL) and two synthetic analogs, Mika (+) - 23a (23a) and Mika (-) - 23d (23d). For this purpose, human T lymphocytes were pre-treated with compounds for 2 h and then stimulated with Concanavalin A (ConA) for 48 h. After that, intra and extracellular Cyp, A.; B, C and D levels were determined. CsA was used as control of effect in all experiments. Our results show that natural and synthetic gracilins downregulate intracellular Cyps expression induced by ConA as well as the release of Cyps to the media culture.

#### **The Nagoya Protocol and Its Implications on the EU Atlantic Arc Countries Legislation** 

MartinsJoana^1^,^2^CruzDiogo^1^,^2^VasconcelosVitor^1^,^2^1Faculty of Sciences, University of Porto, Rua do Campo Alegre, 4169-007 Porto, Portugal2Interdisciplinary Center of Marine and Environmental Research (CIIMAR/CIMAR), University of Porto, Terminal de Cruzeiros do Porto de Leixões, Avenida General Norton de Matos, S/N, 4450-208 Matosinhos, Portugal

Research on Marine Natural Products frequently evolves the work with genetic resources (GR) and associated data. The Nagoya Protocol on Access to Genetic Resources and Fair and Equitable Sharing of Benefits Arising from their Utilization came into force in October 2014, and since then, it is crucial for researchers to ensure that they have legal clarity in how to use the GR.

In the European Union (EU), novel legislation had to be developed in order to apply the obligatory elements of the Protocol, namely Regulation (EU) No. 511/2014 and Implementing Regulation (EU) 2015/1866. As a consequence, EU Member-States had to develop their own legislation to implement the Nagoya Protocol (NP) and the EU regulations. One important fact that distinguishes the national legislation of the EU Member-States is that some countries choose to control access to the GR, while others don’t. This fact, among others, makes the use of GR within the EU a practical challenge. The EU Atlantic Arc countries, including Portugal, Spain, France, The United Kingdom and Northern Ireland and Ireland, share an attractive coastline to what concerns the potential of their GR. In this way, it is important for GR users to be informed about the existing regulations and the national differences that may occur within the EU countries. We this in mind, we have conducted a bibliographic research related to the origins and the main content of the NP. Its main implications in the national legislation of several countries form the Atlantic Arc region will be presented.

##### **Acknowledgments** 

This work was done in the framework of the INTERREG Atlantic Area European programme EnhanceMicroalgae project EAPA_338/2016. This work was supported by the FCT Project UID/Multi/04423/2019.

#### **Cyanobacteria as a Source of Anticancer Compounds** 

FerreiraLeonor^1^ScharfensteinHugo^1^KrempelTim^1^MoraisJoão^1^PretoMarco^1^VasconcelosVitor^1^,^2^UrbatzkaRalph^1^ReisMariana^1^1CIIMAR, Interdisciplinary Centre of Marine and Environmental Research, Terminal de Cruzeiros de Leixões, Av. General Norton de Matos s/n 4450-208 Matosinhos, Portugal2FCUP, Faculty of Sciences, University of Porto, Rua do Campo Alegre 1021/1055, 4169-007 Porto, Portugal

In the past years, cyanobacteria secondary metabolites have been largely studied for their potential as a source of natural products with pharmaceutical interest. As the rates of cancer incidence are increasing, it is important to improve the search for new therapeutics, by discovering new sources of medications. Cyanobacteria are promising producers of anticancer compounds, as the antibody-drug conjugate Brentuximab vedotin (Adcetris™), an FDA approved drug used in the treatment of lymphomas, is of cyanobacterial origin, and multiple other compounds originated from these microorganisms are currently on clinical trials. To assess the potential of cyanobacteria strains belonging to the Blue Biotechnology and Ecotoxicology Culture Collection (LEGE-CC) to produce new compounds, we established a method of fractionation of crude extracts, combined with metabolomic analysis, as well as an optimization of anticancer assays using a physiologically relevant model, 3D cell spheroids. To create a library of cyanobacterial fractions, multiple strains belonging to the Nostocales order were cultured and the crude, obtained from methanolic extraction of dried biomass, was further fractionated by HPLC. The library was tested on 3D cell spheroids from the HCT 116 cell line, after optimization of the most sensitive assays. HRLCMS/MS data of the crude extracts were submitted to Global Natural Products Social Molecular Networking for dereplication. In this work, we present the results from the optimization of the methodologies used in the screening of the LEGE-CC strains.

##### **Acknowledgments** 

This research was developed under CYANCAN project PTDC/MED-QUI/30944/2017, co-financed by NORTE 2020, Portugal 2020 and the European Union through the ERDF, and by FCT through national funds and was additionally supported by the FCT strategic fund UID/Multi/04423/2019.

#### ***Phaeurus antarcticus* and Associated Fungus: Photoprotective Potential and regulation of Human Neutrophil Function** 

SantosG. S.^1^RangelK. C.GasparL. R.^1^CanicobaN. C.^1^Marzocchi-MachadoC. M.^1^ColepicoloP.^2^DebonsiH. M.^1^1School of Pharmaceuticals Sciences of Ribeirão Preto—University of São Paulo, Ribeirão Preto, São Paulo 14040-903, Brazil2Institute of Chemistry—University of São Paulo, São Paulo 05508-000, Brazil

Antarctic macroalgae and their endophytic fungi are an underexplored source of bioactive molecules. Herein, chemical profile, photoprotective and immunomodulatory potential of *Phaeurus antarcticus* and its associated fungus *Penicillium purpurogenum* were evaluated*. P. antarcticus* n-hexane (Pa-HX) and ethyl acetate (Pa-EtOAc) extracts were analyzed by GC-MS. *P. purpurogenum* EtOAc crude extract was fractionated to yield 9 fractions (PpA-PpI) and submitted to different chromatographic techniques to obtain a pure compound. NMR-H1 analysis were taken to structure elucidation. Photoprotective potential was assessed by evaluating the UVA/UVB absorption, photostability, phototoxicity test (OEDC-TG-432) and UV-B photoprotection assay performed in immortalized human-keratinocytes (HaCaT) using cell viability after irradiation with a cytotoxic UV-B dose (300 mJ/cm^2^). Scavenger activity was evaluated using DPPH. Cytotoxicity to neutrophils was measured by Trypan-Blue exclusion assay and regulation effect of reactive oxygen species (ROS) production by luminol-dependent chemiluminescence. Aspergillumarin A (**Aa**) was isolated from PpE-fraction. The molecule exhibited UV-B (λmax, nm): 312, photostability and UV-B photoprotection effects increasing cell viability to 96% (200 μg/mL) and 64% (100 μg/mL) after UV-B irradiation when compared to the UV-B filter ethylhexyl metoxycinnamate (MTX) which enhanced cell viability only to 56% (200 μg/mL) (*p* < 0.05). P.a-HX, P.a-EtOAc, PpE-fraction and Aspergillumarin A presented down and up-regulation of ROS production respectively. None of the samples presented DPPH scavenger activity suggesting that this regulatory effect may result from other metabolic pathways. Results present *P. antarcticus* (first report of bioactivity) and *P. purpurogenum* as sources of molecules with photoprotective and immunomodulatory potential.

**Figure:**
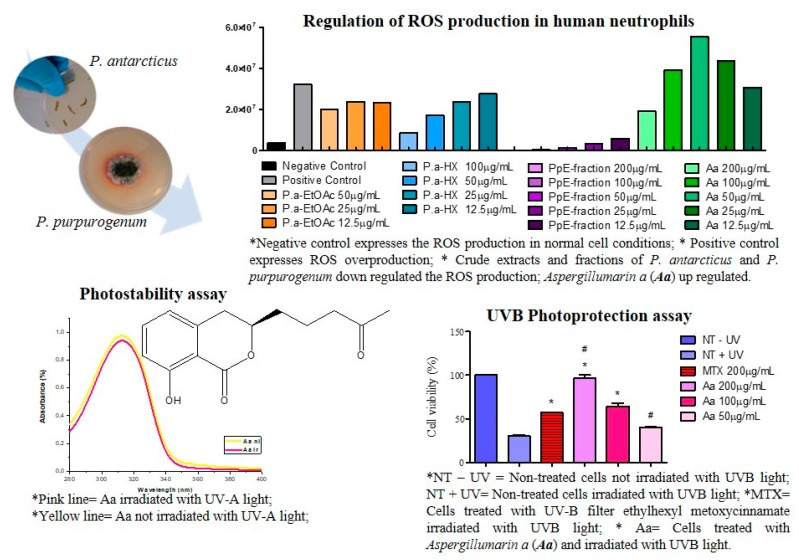
**Graphical Abstract**

#### **Anti-Prostate Cancer Mechanism of Lu01-M Extract from Marine Actinomycete, *Streptomyces* sp.** 

ChenYu-Cheng^1^LuMei-Chin^2^,^3^El-ShazlyMohamed^4^,^5^ShihShou-Ping^6^WuYang-Chang^7^,^8^1The Ph.D. Program for Cancer Biology and Drug Discovery, China Medical University and Academia, Sinica, Taichung 404, Taiwan2Graduate Institute of Marine Biotechnology, National Dong Hwa University, Pingtung 944, Taiwan3National Museum of Marine Biology & Aquarium, Pingtung 944, Taiwan4Department of Pharmacognosy and Natural Products Chemistry, Faculty of Pharmacy, Ain-Shams University, Organization of African Unity Street, Abassia, Cairo 11566, Egypt5Department of Pharmaceutical Biology, Faculty of Pharmacy and Biotechnology, German University in Cairo, Cairo 11432, Egypt6Doctoral Degree Program in Marine Biotechnology, National Sun Yat-Sen University, Lien-Hai Road, Kaohsiung 804, Taiwan7Chinese Medicine Research and Development Center, China Medical University Hospital, Taichung 404, Taiwan8Graduate Institute of Natural Products, College of Pharmacy, Kaohsiung Medical University, Kaohsiung 807, Taiwan

There are many sources of marine natural products, including corals, sponges, tunicates, mollusks, algae, and microorganisms. The biological activity of these marine lives can be used as a source of drug development. It is considered as an important pillar for the treatment of diseases, but more and more research shows that many of the active substances present in marine organisms are in fact produced by chemical interactions between symbiotic microorganisms such us bacteria, fungi, algae, etc. This article screened the marine actinomycetes *Streptomyces* sp., by using ethyl acetate to extract the secondary metabolite, Lu01-M. Lu01-M showed anti-prostate cancer effect and the most sensitive cancer cell line was PC3, with IC_50_ 2.45 ± 0.27 and 1.69 ± 0.21 μg/mL after 24 and 48 h treatment, respectively. Thus, PC3 cells were subjected to further investigation. Lu01-M also affecting cell cycle related protein, inhibited cells proliferation and migration. The use of increasing doses of Lu01-M (0 to 6.25 μg/mL) increased the percentage of disruption of mitochondrial membrane potential, induced the reactive oxygen species and endoplasmic reticulum stress, and inhibited the p-Akt signaling, leading to decrease the cell viability. We further expanded our investigation to evaluate the antitumor effect of Lu01-M in vivo xenograft animal models. Animal experiments showed that Lu01-M could inhibit tumor growth without affecting the body weight of mice and biochemical index. Taken together, these findings suggest that Lu01-M has a great potential in the development of anti-cancer drugs.

#### **Isolation of *Labrenzia alexandrii* Strains Producing a Pederin-Like Compound Using a Dilution to Extinction Methodology in 384 Microplate Format** 

BenítezXulioGonzálezElena G.ZuñigaPazRodriguezPilarCañedoLibradade la CalleFernandoCuevasCarmenR&D Area, PharmaMar, S.A., Avda. de los Reyes 1, Pol. Ind. La Mina, 28770 Colmenar Viejo, Madrid, Spain

Despite the vast efforts done to overcome “The great plate count anomaly”, most of the microbiological diversity remains unexplored due to the incapability of its cultivation. Several methods had been used to overcome this obstacle and help in the discovery of new bioactive compounds from microorganisms. Dilution-to-extinction method has been successfully used for the isolation of new marine bacteria during the last 30 years. A good example has been the isolation of SAR11 lineage-like groups and other oligotrophs in the past years.

We have used the concept of the dilution-to-extinction’s method in an attempt of isolation and drug discovery of marine bacteria. Using a 384-microplate format several strains belonging to *Labrenzia alexandrii* were isolated, screened and the chemical dereplication showed the production of pederin analogues. The first description of a pederin-like compound from the culture of a free-living bacteria was published by our research group in 2017 using a classical solid medium platting. Here we describe the cultivation, and the molecular characterization of the strains. As far as we know, this is the first time the dilution-to-extinction method has been used for antitumor compounds discovery.

### ***Theme 4. Biosynthesis of Marine Natural Products*** 

#### **Regioselective Prenylation of Peptide Termini during the Biosynthesis of the Natural Product Muscoride** 

MattilaAntti^1^AndstenRose-Marie^1^JumppanenMikael^2^AssanteMichele^2^JokelaJouni^1^WahlstenMatti^1^MikulaKornelia M.^3^GuggerMuriel^4^KoskelaHarri^5^SivonenKaarina^1^Yli-KauhaluomaJari^2^IwaïHideo^3^FewerDavid P.^1^1Department of Microbiology, P.O. Box 56, Viikki Biocenter, Viikinkaari 9, University of Helsinki, FI-00014 Helsinki, Finland2Drug Research Program, Division of Pharmaceutical Chemistry and Technology, Faculty of Pharmacy, Viikinkaari 5 E (P.O. Box 56), University of Helsinki, FI-00014 Helsinki, Finland3Research Program in Structural Biology and Biophysics, P.O. Box 65, Institute of Biotechnology, University of Helsinki, FI-00014 Helsinki, Finland4Collection des Cyanobactéries, Département de Microbiologie, Institut Pasteur, 28 Rue du Docteur Roux, 75724 CEDEX 15, 75015 Paris, France5VERIFIN, Department of Chemistry, University of Helsinki, P.O. Box 55, FI-00014 Helsinki, Finland

Prenylation is important in the biosynthesis of many natural products and plays an important role in increasing structural variety and biological activity. Cyanobactins are a growing family of ribosomally produced and post-translationally modified natural products. Muscoride A is an unusual linear peptide alkaloid that contains two contiguous oxazoles and is uniquely prenylated on both the amino and carboxy termini. Here we show that muscoride is synthesized through post-translational modifications of a short precursor protein. We identified the 12.7 kb muscoride (*mus*) biosynthetic pathway from *Nostoc* sp. PCC 7906 that is responsible for the elaboration of muscoride through post-translational modifications of the short MusE precursor protein. The *mus* biosynthetic gene cluster encodes nine cyanobactin biosynthetic genes including MusF1 and MusF2, two paralogous copies of the cyanobactin prenyltransferase. The predicted tetrapeptide substrate of MusF1 and MusF2 was synthesized through novel tandem cyclization route in eight steps. Biochemical assays demonstrated that MusF1 acts on the carboxy terminus while MusF2 acts on the amino terminus. The MusF1 from both strains prenylated in forward orientation. The two MusF2 differed notably as the MusF2 from *Nostoc* sp. PCC 7906 catalyzed reverse prenylation whereas MusF2 from *Nostoc* sp. UHCC 0398 catalyzed forward prenylation. We identified a new muscoride variant from *Nostoc* sp. UHCC 0398, which contains three contiguous oxazoles and both termini are protected by forward prenyl groups. This finding expands the regiospecific chemical functionality and genetic diversity of cyanobactin prenyltransferases.

#### ***Hyella patelloides*, a Marine Cyanobacterium with a Unique Biosynthetic Potential** 

BritoÂngela^1^,^2^VieiraJorge^1^,^2^VieiraCristina P.^1^,^2^ZhuTao^3^LeãoPedro^4^RamosVitor^4^MartinsTeresa^1^,^2^,^4^LuXuefeng^3^,^5^VasconcelosVitor M.^4^,^6^GuggerMuriel^7^TamagniniPaula^1^,^2^,^6^1i3S—Instituto de Investigação e Inovação em Saúde, Universidade do Porto, 4200-135 Porto, Portugal2IBMC—Instituto de Biologia Molecular e Celular, Universidade do Porto, 4200-135 Porto, Portugal3Key Laboratory of Biofuels, Shandong Provincial Key Laboratory of Synthetic Biology, Qingdao Institute of Bioenergy and Bioprocess Technology, Chinese Academy of Sciences, Qingdao 266101, China4Interdisciplinary Centre of Marine and Environmental Research (CIIMAR/CIMAR), University of Porto, 4450-208 Matosinhos, Portugal5Laboratory for Marine Biology and Biotechnology, Qingdao National Laboratory for Marine Science and Technology, Qingdao 266237, China6Faculdade de Ciências, Departamento de Biologia, Universidade do Porto, 4169-007 Porto, Portugal7Institut Pasteur, Collection des Cyanobactéries, 75015 Paris, France

Natural products (NPs) research has played a crucial role in drug discovery, disclosing a significant number of novel metabolites. However, there is still an urgent demand for new effective drugs, such as antibiotics, and marine cyanobacteria are an excellent source for natural-derived molecules. Previously, we isolated and characterized a new *Hyella* species—*H. patelloides*—from a rocky beach on the North of Portugal [1,2]. A preliminary metabolomic analysis revealed the potential of this strain to produce compounds related to naopopeptin and antanapeptin as well as novel ones [3]. Therefore, this species is being further investigated at genomic and metabolomic level. The *H. patelloides* genome is the first one reported for the *Hyella* genus, and a comparative genomic analysis with its closest cyanobacterial counterparts revealed its distinctiveness, in particular its unique biosynthetic potential. In *Hyella*’s genome it was possible to identify more than twenty natural products BGCs, most of them orphan. In addition, one BGC was identified as being putatively involved in the production of terminal olefin, and our results showed that *H*. *patelloides* produces hydrocarbon with C_15_ chain length, and synthesizes C_14_, C_16_ and C_18_ fatty acids exceeding 4% of the dry cell weight. Currently, a cultivation scale-up is ongoing to obtain enough biomass for the isolation/characterization of novel compounds.

References

1. Brito, Â.; Ramos, V.; Seabra, R.; Santos, A.; Santos, C.L.; Lopo, M.; Ferreira, S.; Martins, A.; Mota, R.; Frazão, B.; et al. Culture-dependent characterization of cyanobacterial diversity in the intertidal zones of the Portuguese coast: a polyphasic study. *Syst. Appl. Microbiol.*
**2012**, *35*, 110–119.
2. Brito, Â.; Ramos, V.; Mota, R.; Lima, S.; Santos, A.; Vieira, J.; Vieira, C.P.; Kaštovský, J.; Vasconcelos, V.M.; Tamagnini, P. Description of new genera and species of marine cyanobacteria from the Portuguese Atlantic coast. *Mol. Phylogenet. Evol.*
**2017**, *111*, 18–34.
3. Brito, Â.; Gaifem, J.; Ramos, V.; Glukhov, E.; Dorrestein, P.C.; Gerwick, W.H.; Vasconcelos, V.M.; Mendes, M.V.; Tamagnini, P. Bioprospecting Portuguese Atlantic coast cyanobacteria for bioactive secondary metabolites reveals untapped chemodiversity. *Algal. Res.*
**2015**, *9*, 218–226.

#### **Branching in the Biosynthesis of Kalimantacin Antibiotics** 

WeirAngusWalkerPaulCrumpMattWillisChrisSchool of Chemistry, University of Bristol, Cantock’s Close, Bristol BS8 1TS, UK

**Figure 42:**
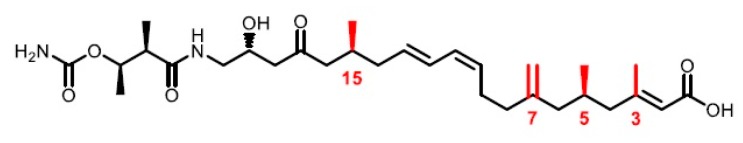
The chemical structure of Kalimantacin A.

Kalimantacin A (Figure 42) is a narrow spectrum polyketide antibiotic with strong, selective activity against *Staphylococci* including multidrug resistant strains (MRSA, VISA) [1,2]. Recently, the relative and absolute stereochemistry of kalimantacin A and the precursor 17,19-diol have been assigned using a combination of chemical degradation, fragment synthesis, NMR spectroscopy and bioinformatics [3]. 

**Scheme 1:**
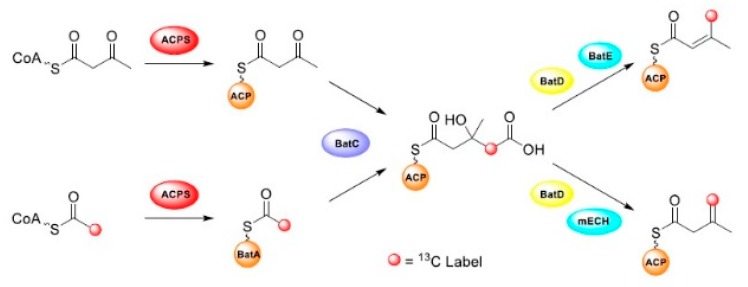
In vitro reconstitution of the β-branching pathway.

Particularly fascinating structural features are the presence of β-branches in kalimantacin. Although these are common in polyketides kalimantacin is, to date, the only known polyketide with three distinct classes of β-branch inserted sequentially by the same cassette (at C-3, C-5 and C-7). During in vitro studies on the reconstitution of the β-branching pathway we developed a powerful NMR technique that allowed the monitoring of a ^13^C signal on a single ACP or on multiple protein modules (Scheme 1). This enabled the formation of a range of β-methyl branches on a PKS assembly of 80 kDa to be detected. The results of these studies will be described.

References

1. Tokunaga, T.; Kamgiri, K.; Orita, M.; Nishikawa, T.; Shimizu, M.; Kaniwa, H. Kalimantacin A, B, and C, Novel Antibiotics Produced by *Alcaligenes* sp. YL-02632S. *J. Antibiot.*
**1996**, *49*, 140–144.
2. Mattheus, W.; Gao, L.J.; Herdewijn, P.; Landuyt, B.; Verhaegen, J.; Masschelein, J.; Volkhaert, G.; Lavigne, R. The Kalimantacin/Batumin Biosynthesis Operon Encodes a Self-Resistance Isoform of the FabI Bacterial Target. *Chem. Biol.*
**2010**, *17*, 149–159.
3. Thistlethwaite, I.R.G.; Bull, F.M.; Cui, C.; Walker, P.D.; Gao, S.S.; Wang, L.; Song, Z.; Masschelein, J.; Lavigne, R.; Crump, M.P.; et al. Elucidation of the relative and absolute stereochemistry of the kalimantacin/batumin antibiotics. *Chem. Sci.*
**2017**, *8*, 6196–6201.

#### **A Cyanobacterial PKS/NRPS Biosynthetic Pathway That Connects Polyhalogenated Fatty Acids to Alpha-Keto Acids** 

AbtKathleen^1^,^2^LeãoPedro^2^1PhD Program Biomedical Sciences, Institute of Biomedical Sciences Abel Salazar, University of Porto (ICBAS/UP), Rua de Jorge Viterbo Ferreira 228, 4050-313 Porto, Portugal2Interdisciplinary Centre of Marine and Environmental Research (CIIMAR/CIMAR), University of Porto, Avenida General Norton de Matos, s/n, 4450-208 Matosinhos, Portugal

Cyanobacteria are a rich source of bioactive secondary metabolites with potential for pharmacological and biotechnological applications. Many of these compounds derive from polyketide synthase (PKS) and non-ribosomal peptide synthetase (NRPS) assembly lines. Such pathways are typically fully encoded in biosynthetic gene clusters (BGCs). Despite the enormous progress in genome sequencing technologies and computational cluster detection tools, many compounds do not have an associated BGC and vice versa. Understanding the complex biosynthesis of secondary metabolites, however, provides valuable insight into the unique enzymatic machinery of cyanobacteria. This knowledge in turn can enable microbial production of industrially relevant compounds as well as reveal new biocatalysts to facilitate synthetic reactions. Here, we identify a biosynthetic pathway of a hybrid PKS/NRPS nature that is responsible for the biosynthesis of natural products consisting of a polychlorinated fatty acyl moiety and an α-keto acid. The putative BGC was detected by mining the genome of the producing *Sphaerospermopsis* sp. strain for halogenase homologs. Feeding studies with stable-isotope labelled fatty acids and α-keto acids followed by LC-HRMS analysis confirmed label incorporation of both building blocks into the hybrid compounds.

#### **Elucidation and Exploitation of Mupirocin and Thiomarinol Biosynthetic Pathways** 

WangLuoyi^1^SongZhongshu^1^CrumpMatthew P.^1^SimpsonThomas J.^1^RacePaul R.^2^WillisChristine L.^1^1School of Chemistry, Cantock’s Close, University of Bristol, Bristol BS8 1TS, UK2School of Biochemistry, University Walk, University of Bristol, Bristol BS8 1TD, UK

**Figure 43:**
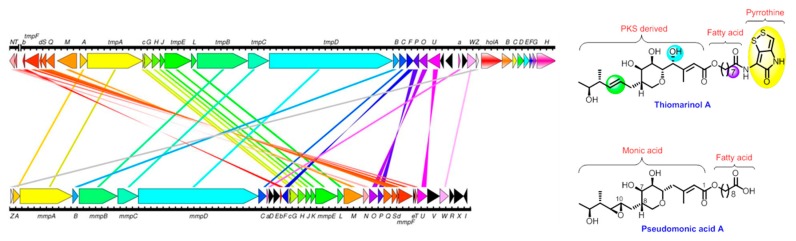
Comparison of the organisation of the thiomarinol biosynthesis gene cluster (upper line) with the mupirocin gene cluster (lower line), and structures of thiomarinol A and pseudomonic acid A.

Mupirocin is the active ingredient of the ointment Bactroban marketed by GSK. It is a mixture of pseudomonic acids, A.; B, and C produced by *Pseudomonas fluorescens* NCIMB 10586 via *trans*-AT polyketide synthases (PKSs). Thiomarinols are closely related to mupirocin, being produced by the marine bacterium *Pseudoalteromonas* spp. SANK 73390 via a very similar biosynthetic gene cluster (Figure 43), with the addition of a non-ribosomal peptide synthase (NRPS)-encoded pyrrothine moiety [1,2].

Our previous studies on detailed metabolic profiling of mutant strains generated by systematic inactivation of PKS and tailoring genes, along with re-feeding of isolated metabolites to mutant stains have provided us insights into mupirocin biosynthesis. These results suggested the presence of many noncanonical features, including the existence of parallel pathways, diverse oxygenation steps, noncolinear PKS gene order, etc. [3–5]. By rational genetic manipulation and heterologous expression of key enzymes from these biosynthetic pathways, we aim to fully understand how polyketides are generated via sophisticated multiple enzyme architectures, which may significantly aid the development of novel bioactive compounds efficiently in scalable amounts and of new biocatalysts of widespread value.

References

1. Thomas, C.M.; Hothersall, J.; Willis, C.L.; Simpson, T.J. Resistance to and Synthesis of the Antibiotic Mupirocin. *Nat. Rev. Microbiol.*
**2010**, *8*, 281–289.
2. Murphy, A.C.; Fukuda, D.; Song, Z.; Hothersall, J.; Cox, R.J.; Willis, C.L.; Thomas, C.M.; Simpson, T.J. Engineered Thiomarinol Antibiotics Active against MRSA Are Generated by Mutagenesis and Mutasynthesis of Pseudoalteromonas SANK73390. *Angew. Chem. Int. Ed.*
**2011**, *50*, 3271–3274.
3. Gao, S.S.; Hothersall, J.; Wu, J.; Murphy, A.C.; Song, Z.; Stephens, E.R.; Thomas, C.M.; Crump, M.P.; Cox, R.J.; Simpson, T.J.; et al. Biosynthesis of Mupirocin by Pseudomonas Fluorescens NCIMB 10586 Involves Parallel Pathways. *J. Am. Chem. Soc.*
**2014**, *136*, 5501–5507.
4. Gao, S.S.; Wang, L.; Song, Z.; Hothersall, J.; Stevens, E.R.; Connolly, J.; Winn, P.J.; Cox, R.J.; Crump, M.P.; Race, P.R.; et al. Selected Mutations Reveal New Intermediates in the Biosynthesis of Mupirocin and the Thiomarinol Antibiotics. *Angew. Chem. Int. Ed.*
**2017**, *56*, 3930–3934.
5. Wang, L.; Parnell, A.; Williams, C.; Bakar, N.A.; Challand, M.R.; van der Kamp, M.W.; Simpson, T.J.; Race, P.R.; Crump, M.P.; Willis, C.L. A Rieske Oxygenase/Epoxide Hydrolase-Catalysed Reaction Cascade Creates Oxygen Heterocycles in Mupirocin Biosynthesis. *Nat. Catal.*
**2018**, *1*, 968–976.

#### **Isotope-Guided Discovery of Cyanobacterial Secondary Metabolites** 

ReisMarianaLeãoPedroInterdisciplinary Center of Marine and Environmental Research (CIIMAR/CIMAR), University of Porto, Terminal de Cruzeiros de Leixões, Av. General Norton de Matos s/n, 4450-208 Matosinhos, Portugal

Cyanobacterial secondary metabolites are an interesting source of natural products for drug discovery, due to their unique and exotic structures. Most of these compounds were discovered through bioactivity-guided screening endeavors. However, recent advances in genomics are revolutionizing the field of microbial natural products discovery [1]. Genome-guided discovery has the advantage of linking compounds with the genes encoding their biosynthesis and vice versa. In this way, the genomisotopic approach aims to unveil the products of cryptic biosynthetic gene clusters (BGC) by using a combination of BGC genomic information and isotope-guided fractionation to expedite their isolation [2]. In this exploratory work, the genomisotopic approach will be applied for the discovery of novel compounds from cyanobacteria belonging to Nostocales order. Thus, selected cyanobacterial genomes (available from NCBI) were be submitted to antiSMASH for BGC analysis. According to the type of compound predicted by antiSMASH, an appropriate isotopic-labeled (^15^N) amino acid was fed to the cyanobacteria culture. The isotope incorporation was followed NMR (HMBC ^1^H-^15^N), which guided the isolation of the compounds through suitable chromatographic techniques (column chromatography and HPLC). The chemical structures of the pure compounds are being deduced from their physical and spectroscopic data (HRMS, 1D and 2D NMR).

##### **Acknowledgments** 

This research was funded by CYANCAN (PTDC/MED-QUI/30944/2017, co-financed by NORTE 2020, Portugal 2020 and the European Union through the ERDF, and by FCT) and by the FCT strategic fund UID/Multi/04423/2019.

References

1. Micallef, M.L.; D’Agostino, P.M.; Sharma, D.; Viswanathan, R.; Moffitt, M.C. Genome mining for natural product biosynthetic gene clusters in the Subsection V cyanobacteria. *BMC Genom.*
**2015**, *16*, 669.
2. Gross, H.; Stockwell, V.O.; Henkel, M.D.; Nowak-Thompson, B.; Loper, J.E.; Gerwick, W.H. The Genomisotopic Approach: A Systematic Method to Isolate Products of Orphan Biosynthetic Gene Clusters. *Chem. Biol.*
**2007**, *14*, 53–63.

#### **Understanding the Biosynthesis of the Fatty Acid Region of Mupirocin and Thiomarinol** 

RoweMatthewWinterAshleyAktarNahidaCrumpMatthewSchool of Chemistry, University of Bristol, Cantock’s Close, Bristol BS8 1TS, UK

**Figure 44:**
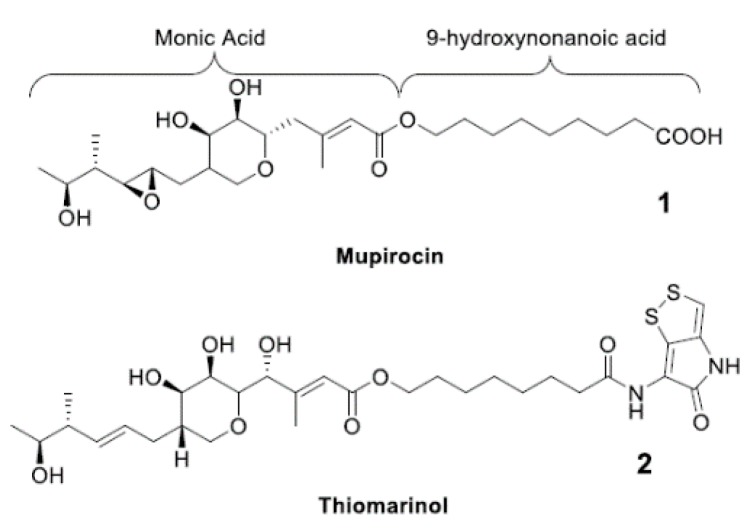
The structure of the main metabolites that make up mupirocin and thiomarinol.

Mupirocin and Thiomarinol are structurally related polyketide antibiotics which strong activity against Methicillin-resistant *Staphylococcus aureus* (Figure 44). The biosynthesis of these compounds from naturally occurring biomolecules is complex due to the action of many *trans*-acting enzymes.

**Figure 45:**
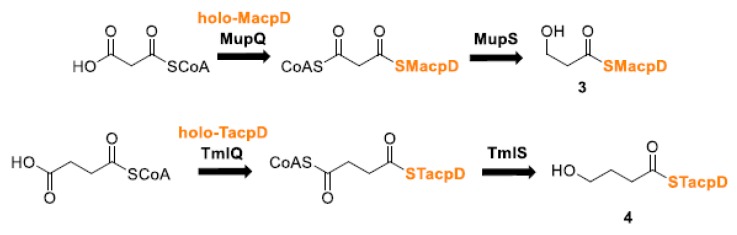
The first step in the formation of the fatty acid chains of mupirocin and thiomarinol.

The initial steps in the formation of the 9-hydroxynonanoic acid region in mupirocin (and 8-hydroxyoctanoic acid in thiomarinol) were shown to occur via the formation of a 3/4 carbon starter units **3** and **4** (Figure 45). These starter units are formed via the action of three enzymes (mAcpD/tAcpD, MupQ/TmlQ and MupS/TmlS) as confirmed by the conduction of in vitro assays with purified protein.

Further work has confirmed the identity of the esterase enzyme proposed to couple the polyketide derived moiety (monic acid) with the fatty acid chain; as confirmed with in vitro assays with purified protein. The selectivity of the activation of esterase enzyme was also tested by tethering the substrate to terminal thiol of various ACPs within the pathway. It is not currently known whether esterification occurs with incomplete (3-carbon) or complete (9-carbon) fatty acid chain so assays will be conducted to test this further.

#### **A Multi-Producer Microbiome Creates Chemical Diversity in the Marine Sponge *Mycale hentscheli*** 

RustMichael^1^HelfrichEric J. N.^1^FreemanMichael F.^1^,^2^NanudornPakjira^1^FieldChristopher^1^RückertChristian^3^PageMichael J.^4^WebbVictoria L.^5^KalinowskiJörn^3^SunagawaShinichi^1^PielJörn^1^1Institute of Microbiology, Eidgenössische Technische Hochschule (ETH) Zurich, Vladimir-Prelog-Weg 1-5/10, 8093 Zurich, Switzerland2Department of Biochemistry, Molecular Biology, and Biophysics and BioTechnology Institute, University of Minnesota–Twin Cities, St Paul, MN 55108, USA3Institute for Genome Research and Systems Biology, Center for Biotechnology, Universität Bielefeld, Universitätsstrasse 25, 33594 Bielefeld, Germany4National Institute of Water and Atmospheric Research Ltd. (NIWA), P.O. Box 893 Nelson, New Zealand5National Institute of Water and Atmospheric Research Ltd. (NIWA), Kilbirnie, P.O. Box 14 901 Wellington, New Zealand

Marine sponges are a rich source of bioactive natural products with considerable drug development potential. Many sponges harbor complex microbial communities that account for a significant portion of the host biomass. For an increasing number of cases, it has been shown that the producers of sponge-derived bioactive metabolites are symbiotic bacteria. The New Zealand sponge *Mycale hentscheli* contains three therapeutically relevant polyketides with potent bioactivities in the low nanomolar range. Here, we present an extreme case of natural product-based mutualism, in which multiple microbiome members contribute to the rich chemistry of their host sponge. We identified biosynthetic gene clusters for the polyketides mycalamide, pateamine and peloruside in three different symbiotic producers and show that all three compounds are produced by *trans*-acyltransferase polyketide synthases. Metagenome mining and binning revealed a large number of additional biosynthetic gene clusters among a broad phylogenetic range of bacteria. Among various predicted polyketides, peptides and other natural product types, we identified a gene cluster predicted to encode the production of a variant of the structurally unusual polytheonamide cytotoxins. The data provide a rationale for the chemical variability of *M. hentscheli* and support the concept that uncultivated bacteria harbor diverse producer taxa with as yet unrecognized drug discovery potential.

#### **Diversity and Distribution of CylC-Like Enzymes among Cyanobacteria** 

EusebioNadia^1^ReisJoão^1^CassellSamantha^2^NakamuraHitomi^2^GlasserNathaniel^2^BalskusEmily^2^LeãoPedro^1^1Interdisciplinary Centre of Marine and Environmental Research (CIIMAR/CIMAR), University of Porto, 4450-208 Matosinhos, Portugal2Department of Chemistry and Chemical Biology, Harvard University, Cambridge, MA 02138, USA

Natural products (NP) are a fruitful source of chemical diversity that has translated into many life-saving drugs. The rates of re-isolation of known NPs are increasing, and the majority of new NPs discovered in the past years are structurally similar to previously isolated compounds. The growing number of available bacterial genomes and the realization that a plethora of NP biosynthetic gene clusters (BGCs) with no associated NP (orphan BGCs) are found in such genomic data, has emphasized that we currently grasp only a small fraction of their (bio)chemical potential. Recent biosynthetic studies on cyanobacterial NPs revealed a novel dimetal halogenase (CylC) capable of selectively acting on unactivated carbon centers, a highly sought-after catalytic activity. These enzymes appear to be found exclusively in cyanobacteria, within orphan BGCs with different architectures. Here we provide insights into the diversity of *cylC* homologs that are found not only in publicly available cyanobacterial genomes but also in our in-house culture collection of cyanobacteria (LEGEcc). We designed different degenerate primers to screen over 300 cyanobacterial LEGEcc strains for *cylC* homologs. Using this strategy, we have identified and partially sequenced more than 50 different *cylC* homologs among different Cyanobacteria groups. In addition, we have searched sequenced genomes from 25 LEGEcc strains and retrieved additional homologs. Finally, we have performed phylogenetic analysis of the newly obtained sequences and all homologs available in the Genbank to provide a comprehensive overview of the diversity of these enzymes. This work will help guide future enzyme and natural product discovery efforts.

#### **Genome Mining for Discovery of New Post-Translationally Modified Peptides in Cyanobacteria from LEGE Culture Collection** 

BrancoRaquel Castelo^1^,^2^VasconcelosVitor^1^,^2^FewerDavid^3^LeãoPedro^1^1CIIMAR, Interdisciplinary Centre of Marine and Environmental Research, 4450-208 Matosinhos, Portugal2Faculty of Sciences, University of Porto, 4069-007 Porto, Portugal3Department of Microbiology, University of Helsinki, Biocenter 1, Viikinkaari 9, FI-00014 Helsinki, Finland

Natural products (NPs) are the basis of many modern medicines and are also important molecules for biotechnological applications. In the last years, NPs produced by cyanobacteria have attracted increasing scientific interest due to their potent bioactivities and structural uniqueness. One abundant group of bioactive NPs produced by cyanobacteria are the Ribosomally synthesized and Post-translationally modified Peptides (RiPPs). These can exhibit high chemical diversity through extensive post-translational modifications (PTMs) of a precursor peptide. As such, a wide range of activities are associated with RiPPs. Genome sequencing and the development of bioinformatics tools used in genome mining approaches has revealed that cyanobacteria carry the genetic potential to produce a variety of RiPPs. However, only a small portion of this diversity has been characterized to date. In this work, we mine the genomes of 30 phylogenetically distinct cyanobacterial strains belonging to Blue Biotechnology and Ecotoxicology Culture Collection (LEGEcc) in order to identify biosynthetic gene clusters encoding RiPPs. We report the abundance, diversity and distinct biosynthetic architecture of a number of different classes of RiPPs in LEGEcc strains, suggesting that these cyanobacteria potentially produce structurally novel secondary metabolites.

#### **Unprecedented Methylated Acetate Starter Unit Involved in the Biosynthesis of Novel Fungal Polyketides** 

MaddahFayrouz ElEgerevaEkaterinaKehrausStefanKönigGabriele M.Institute for Pharmaceutical Biology, University of Bonn, Nussallee 6, 53115 Bonn, Germany

Chemical investigation of the marine-derived fungus *Stachylidium* sp. 293K04 recently led to the isolation and characterization of marilones and marilines, polyketide-derived metabolites which are characterized by a phthalide and phthalimidine skeleton, respectively. Phthalides (isobenzofuranones) and phthalimidines (isoindolinones) both possess a bicylic nucleus derived from the fusion of a benzene ring and either a γ-lactone for phthalides or γ-lactam for phthalimidines. From a series of feeding experiments using ^13^C-labeled precursors we could outline the biogenetic origin of the basic carbon skeleton of marilone A and mariline B. So far, results from [1-^13^C]sodium propionate and l-[*Me*-^13^C]methionine feeding provide convincing evidence that a methylated acetate starter unit initiates the polyketide formation with chain extension by three acetate (malonyl-CoA) units to form a tetraketide intermediate. To the best of our knowledge, the involvement of a methylated acetate as a starter unit in the biosynthesis of marilone A (**1**) and mariline B (**5**) is unique amongst fungal metabolites.

#### **Structural and Mechanistic Studies of Mupirocin and Thiomarinol Biosynthetic Pathway Enzymes: The Putative Reverse Esterase MupB and TmlB** 

AkterNahidaWilliamsChristopherWinterAshleyCrumpMatthew P.School of Chemistry, Cantock’s Close, University of Bristol, Bristol BS8 1TS, UK

**Scheme 2:**
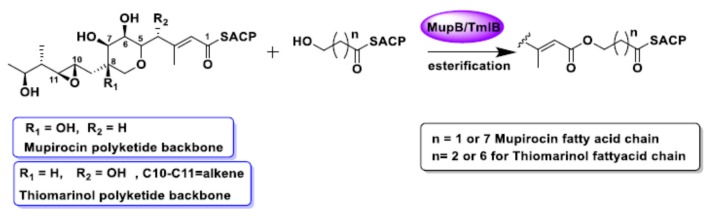
Putative esterification reaction of Mupirocin/Thiomarinol polyketide backbone with fatty acid chain catalyses by MupB/TmlB respectively.

Polyketides are a structurally diverse and pharmacologically interesting class of compounds produced by living systems such as bacteria and fungi. Mupirocin and thiomarinol are polyketide antibiotics produced by *Pseudomonas fluorescens* and *Pseudo-alteromonas sp. SANK73390* respectively. A key step in the biosynthesis of both antibiotics is esterification of a fatty acid sidechain to the main polyketide backbone but the enzyme that catalyses this reaction is unknown. MupB/TmlB are potential candidates for catalysing this reaction and share 37% sequence identity with each other. The aim of this work has been the functional and structural characterisation of both enzymes from these pathways. Cloning, expression, purification and crystallisation of MupB and TmlB allowed the determination of high-resolution crystal structures that have revealed a thiolase-fold common to the FabH like enzymes (KS-III) but containing an alternate catalytic triad that deviates from the typical KS-III and is comprised of Cis-His-Asp rather than Cis-His-Asn [1,2]. The orientation of these catalytic residues was non-canonical and matched a single example from the cervimycin pathway that also catalyses an esterification (CerJ) [3]. We demonstrate that Both MupB and TmlB self-load a simplified monic acid analogue enoyl-pantetheine, but interestingly MupB also loads other short chain acyl-CoAs and acyl-pantetheines. We also present a co-crystal structure of TmlB with enoyl-pantetheine substrate. These structural models allow us to identify the catalytic active site and as well as the putative mechanistic functions of these enzymes in their respective polyketide biosynthetic pathways (Scheme 2).

References

1. Pan, H.; Tsai, S.C.; Meadows, E.S.; Miercke, L.J.; Keatinge-Clay, A.T.; O’Connell, J.; Khosla, C.; Stroud, R.M. Crystal structure of the priming β-ketosynthase from the R1128 polyketide biosynthetic pathway. *Structure*
**2000**, *10*, 1559–1568.
2. Jackson, D.R.; Shakya, G.; Patel, A.B.; Mohammed, L.Y.; Vasilakis, K.; Wattana-Amorn, P.; Valentic, T.R.; Milligan, J.C.; Crump, M.P.; Crosby, J.; et al. Structural and functional studies of the Daunorubicin priming ketosynthase DpsC. *ACS Chem. Biol.*
**2018**, *13*, 141–151.
3. Bretschneider, T.; Zocher, G.; Unger, M.; Scherlach, K.; Stehle, T.; Hertweck, C. A ketosynthase homolog uses malonyl units to form esters in cervimycin biosynthesis. *Nat. Chem. Biol.*
**2012**, *8*, 154–161.

#### **A Novel Enzymatic Esterification Involved in the Biosynthesis of Branched Bartolosides** 

ReisJoão A.FigueiredoSandraLeãoPedro N.Interdisciplinary Centre of Marine and Environmental Research (CIIMAR/CIMAR), University of Porto, 4450-208 Matosinhos, Portugal

Esterification is one of the most important reactions in industrial and biological chemistry. In organic synthesis, carboxyester formation usually relies on the electrophilicity of the carbonyl carbon and the nucleophilicity of an alcohol, as typified by the Fisher esterification. Inside living cells, analogous reactions are performed by acyltransferases, but usually require prior carboxylic acid activation. Under certain conditions, carboxylates can also act as nucleophiles to form esters with primary alkyl halides. However, in biological catalysis, this feature has only been observed in the transition states of haloalkane dehalogenases reactions. Here, we present a novel enzyme, BrtB, that catalyses the esterification of free fatty acids with secondary alkyl halide moieties found in the bartolosides, a group of cyanobacterial chlorinated dialkylresorcinols. This reactivity, in which the carboxylate likely acts as a nucleophile, was confirmed using enzymatic assays with purified recombinant BrtB. Among a series of branched bartolosides formed by BrtB, the most abundant seems to be the ester of bartoloside A and palmitic acid, which we isolated and structurally elucidated. BrtB is the second enzyme of its family to be characterized after CylK, which catalyses a Friedel-Crafts alkylation.

#### **Exploring Fatty Acid and Lipid Metabolism in Cyanobacteria** 

FigueiredoSandra A. C.MartinsTeresa P.AbtKathleenLeãoPedro N.Interdisciplinary Centre of Marine and Environmental Research (CIIMAR/CIMAR), Terminal de Cruzeiros do Porto de Leixões, University of Porto, 4450-208 Matosinhos, Portugal

Cyanobacteria are photosynthetic microorganisms that are prolific producers of Natural Products (NPs). Fatty acid (FA) and lipid metabolism are many times intertwined with cyanobacterial NPs biosynthesis. Despite its relevance, FA metabolism in cyanobacteria has not yet been fully elucidated. In this communication we report some unexpected findings for lipid catabolism in these organisms. We compared the metabolism of *E. coli* expressing a cyanobacterial acyl-ACP synthetase (Aas) with that of phylogenetically diverse cyanobacterial strains. These organisms were used in feeding studies with several stable isotope-labelled FAs. Next, the lipid isotopologs were identified by the respective difference of mass (m/z) using LC-HRMS. These results showed the expected pattern of isotopologs in *E. coli* correspondent to the β-oxidation pathway, a metabolic process responsible for the degradation of the FAs. Although β-oxidation was thought to be ubiquitous in all organisms, it seems to be lacking in cyanobacteria. In fact, our results with cyanobacteria showed an unusual pattern of isotopologs that suggest an unprecedented mechanism of FA degradation. To complement this information, bioinformatics tools were used to determine if cyanobacteria have gene ontologies assigned for enzymes involved in the β-oxidation pathway. Accordingly, we present a phylogenetic analysis that helps to place these findings into an evolutionary context. Taken together, our results have important implications for understanding the biochemical and physiological aspects of lipid metabolism in cyanobacteria.

## Poster Session 3: Synthesis of Marine Natural Products and Medicinal Chemistry, and Marine Natural Products Chemical Biology and Biotechnology 

### ***Theme 5. Synthesis of Marine Natural Products and Medicinal Chemistry*** 

#### **Mi180021, a Novel ADC with a New Marine DNA Binder Payload, Shows Outstanding Activity against HER2 Expressing Tumors** 

LatorreAlfonsoMartínezValentínÁlvarezSusanaFranceschAndrésDomínguezJuan M.GuillénMaría JoséMuntSimonAvilésPabloCuevasCarmenResearch and Development, PharmaMar, S.A.; Avda. de los Reyes 1, Pol. In. La Mina, 28770 Colmenar Viejo, Madrid, Spain

After more than 30 years of research to develop antibody drug conjugates (ADCs) using microtubule inhibitors or DNA interacting agents as payloads, only four ADCs have been approved by the FDA and the discovery of new payloads remains an unmet need. PharmaMar is a biopharmaceutical company dedicated to the discovery and development of new antitumor compounds of marine origin. We recently described the remarkable in vivo activity of MI130004 [1], a novel ADC composed of Trastuzumab conjugated to a potent new marine-compound PM050489 which binds to tubulin at a novel binding site, that led to an exclusive licensing agreement with Seattle Genetics in February 2018. Herein we describe a new ADC, MI180021, from conjugation of Trastuzumab with another new marine-derived analog PM160057 via cysteine residues and a cleavable linker. PM160057 is a synthetic compound belonging to the same family as Yondelis^®^ (trabectedin) that is commercialized for the treatment of advanced soft tissue sarcomas and relapsed ovarian cancer, and Zepsyre (lurbinectedin, PM01183) currently in Phase III in small cell lung cancer (SCLC). The new ADC, MI180021 shows highly potent in vitro activity and selectivity for cell lines with high HER2 expression. In vivo evaluation in mice is ongoing.

References

1. Avilés, P.M.; Domínguez, J.M.; Guillén-Navarro, M.J.; Muñoz-Alonso, M.J.; Mateo, C.; Rodríguez-Acebes, R.; Molina-Guijarro, J.M.; Francesch, A.; Martínez-Leal, J.F.; Munt, S.; et al. MI130004, a novel antibody-drug conjugate combining trastuzumab with a molecule of marine origin, shows outstanding in iivo activity against HER2-expressing tumors. *Mol. Cancer Ther.*
**2018**, *17*, 786.

#### **Mi130110, a New ADC Combining an Anti-CD13 Antibody and a Payload of Marine Origin Shows Remarkable In Vivo Activity** 

MateoCristinaRodríguezRaquelMuñoz-AlonsoMaría JoséFranceschAndrésDomínguezJuan M.GuillénMaría JoséMuntSimonAvilésPabloCuevasCarmenResearch and Development, PharmaMar, S.A.; Avda. de los Reyes 1, Pol. In. La Mina, 28770 Colmenar Viejo, Madrid, Spain

In the search for novel antibody-drug conjugates (ADCs) that improve the therapeutic arsenal against cancer it is imperative to identify novel targets to direct the antibody moiety. CD13 (aminopeptidase-N) possesses several features that are typical of an attractive ADC target and data reported in the literature show that it is highly expressed in different tumor cells but not in the corresponding healthy tissues. We have prepared a novel ADC, MI130110, by conjugation through a non-cleavable linker of the marine compound PM050489, a tubulin binding molecule whose efficacy as an ADC payload has previously been demonstrated. The conjugation did not affect the binding or the internalization efficiency of the antibody and MI130110 showed remarkable activity and selectivity in vitro in CD13-expressing tumor cell lines causing growth arrest and cell death. When tested in vivo, this newADC exhibited excellent antitumor activity in a CD13-positive fibrosarcoma murine model, with total remissions in a significant number of the treated animals, but failed to show any activity in a myeloma model not expressing CD13, thereby corroborating the selectivity of the ADC. Mitotic catastrophes, typical of the payload mechanism of action, were observed in cells treated with MI130110 both in vitro and in vivo, confirming the correct intracellular processing of the ADC and showing that conjugation to the antibody did not hinder the antiproliferative properties of the marine drug. These results demonstrate the suitability of CD13 as a novel ADC target as well as the effectiveness of ADC MI130110 as a promising antitumor therapeutic agent.

#### **Fighting Multiparasitism, a Neglected Reality in Marginalised Community: New Tools from the Sea** 

ImperatoreConcetta^1^CasertanoMarcello^1^LucianoPaolo^1^AielloAnna^1^GimmelliRoberto^2^RubertiGiovina^2^ParapiniSilvia^3^BasilicoNicoletta^3^AvundukSibel^4^PersicoMarco^1^FattorussoCaterina^1^MennaMarialuisa^1^1Department of Pharmacy, University of Naples “Federico II”, Via D. Montesano 49, 80131 Napoli, Italy2Institute of Cell Biology and Neurobiology, National Research Council, Campus A. Buzzati-Traverso, Via E. Ramarini, 32, 00015 Monterotondo (Roma), Italy3Department of Biomedical, Surgical and Dental Sciences. University of Milan, Via Pascal 36, 20133 Milan, Italy4Department Vocational School of Medicinal Health Services, Mugla University, Mugla 48187, Turkey

Neglected tropical diseases (NTDs), caused by several parasitic agents (*Plasmodium* spp., *Schistosoma* spp., *Leishmania* spp.), are still characterized by a high morbidity percentage worldwide. The therapeutic alternatives for neglected pathologies are very limited, first due to the drug-resistance phenomena but also due to unfavourable toxicity profiles and the difficult administration procedures of the few available chemical entities. A further drawback is represented by multiparasitism, namely the concurrent infestation of a single host by more than one parasite species, which poses additional diagnostic and therapeutic challenges [1]. In this view, a research aimed to discover a new chemical entity active against several parasites is crucial and the marine environment may be important resource [2]. As part of our own continuing search for new leads in the treatment of NTDs [3–5] we have explored the anti infective properties of the sponge derived metabolites avarone and avarol, as well as those of the semisynthetic thiazoavarone, obtained by converting the quinone avarone in the relevant thiazinoquinone. All compounds were shown to be active against the D10 and W2 strains of *P. falciparum*, the juvenile and adult forms of the worm *S. mansoni*, and both the promastigote and amastigote stages of *L. infantum* and *L. tropica*.

References

1. Steinmann, B.; Utzinger, J.; Du, Z.W.; Zhou, X.N. Multiparasitism: A Neglected Reality on Global, Regional and Local Scale. *Adv. Parasit.*
**2010**, *73*, 21–50.
2. Mayer, A.M.S.; Rodríguez, A.D.; Taglialatela-Scafati, O.; Fusetani, N. Marine Pharmacology in 2012–2013: Marine Compounds with Antibacterial, Antidiabetic, Antifungal, Anti-Inflammatory, Antiprotozoal, Antituberculosis, and Antiviral Activities; Affecting the Immune and Nervous Systems, and Other Miscellaneous. *Mar. Drugs*
**2017**, *15*, 273.
3. Imperatore, C.; Persico, M.; Aiello, A.; Luciano, P.; Guiso, M.; Sanasi, M. F.; Taramelli, D.; Parapini, S.; Cebrián-Torrejón, G.; Doménech-Carbó, A.; Fattorusso, C.; Menna, M. Marine inspired antiplasmodial thiazinoquinones: Synthesis, computational studies and electrochemicalassays. *RSC Adv.*
**2015**, *5*, 70689–70702.
4. Imperatore, C.; Persico, M.; Senese, M.; Aiello, A.; Casertano, M.; Luciano, P.; Basilico, N., Parapini, S.; Paladino, A.; Fattorusso, C.; Menna, M. Exploring the antimalarial potential of the methoxy-thiazinoquinone scaffold: Identification of a new lead candidate. *Bioorg. Chem.*
**2019**, *85*, 240–252.
5. Casertano, M.; Casertano, M.; Imperatore, C.; Luciano, P.; Aiello, A.; Putra, M.Y.; Gimmelli, R.; Ruberti, R.; Menna, M. Chemical Investigation of the Indonesian Tunicate Polycarpa aurata and Evaluation of the Effects Against Schistosoma mansoni of the Novel Alkaloids Polyaurines A and B. *Mar. Drugs*
**2019**, *17*, 278–289.

#### **Pharmacophore-Directed Retrosynthesis: A Function-Oriented Approach to Natural Product Total Synthesis** 

RomoDanielXueHoaranTruaxNathanyalVanKhoiTaoYongfengDepartment of Chemistry & Biochemistry, Baylor University, Waco, TX 76798-7348, USA

**Figure 46:**
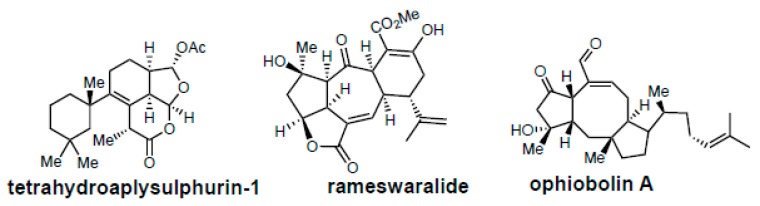
Selected natural products for pharmacophore-directed retrosynthesis.

The architecture and bioactivity of natural products frequently serves as an embarkation point for exploration of biologically-relevant chemical space. Total synthesis followed by derivative synthesis has historically enabled a deeper understanding of structure-activity relationships. However, synthetic strategies toward a natural product are not always guided by hypotheses regarding minimal structural features required for bioactivity i.e., the pharmacophore. We will present a new approach to natural product total synthesis that we term ‘pharmacophore-directed retrosynthesis’ [1]. In this strategy, a hypothesized, pharmacophore of a natural product is selected as an early synthetic target and this dictates the retrosynthetic analysis. In an ideal application, sequential increases in structural complexity of this minimal structure enables development of an SAR profile throughout the course of the total synthesis effort. This approach has the potential to identify simpler congeners retaining bioactivity at a much earlier stage of a synthetic effort. A few select natural products, like the ones depicted in Figure 46, that are currently being pursued in our group utilizing this strategy, along with caveats, will be described.

References

1. Abbasov, M.E.; Alvariño, R.; Chaheine, C.M.; Alonso, E.; Sánchez, J.A.; Conner, M.L.; Alfonso, A.; Jaspars, M.; Botana, L.M.; Romo, D. Simplified, Potent Immunosuppressive and Neuroprotective Agents Based on Gracilin A. *Nat. Chem.*
**2019**, *11*, 342–350.

#### **Photoreactive Hemiasterlin Derivatives Sharing a 2-Azidobenzimidazole Moiety** 

KanitzNils E.LindelThomasTU Braunschweig, Institute of Organic Chemistry, M247 38106 Braunschweig, Germany

**Figure 47:**
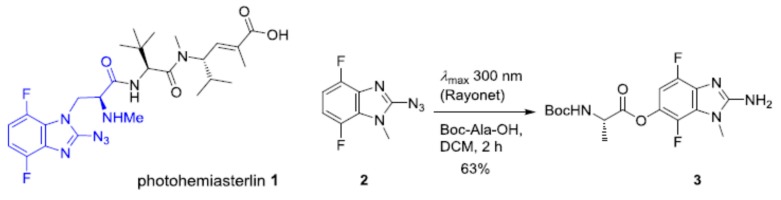
Photohemiasterlin and a typical irradiation experiment with 2-azido-1-methylbenzimidazole

The marine peptide hemiasterlin [1–4] has become a lead structure for drug development. The molecule binds to the tubulin interdimer interface, as determined for its close structural analogue HTI-286 [5]. Moreover, it is possible to alter the structure of the indole section without loosing biological activity. This makes hemiasterlin an ideal model compound for investigating the efficiency of new methods of photoaffinity labelling. Having synthesized hemiasterlin via an organocatalyzed α-hydrazination of a sterically congested aldehyde [6], we turned to photoreactive derivatives of hemiasterlin, such as **1** that contains a 2-azidobenzimidazole moiety instead of the indole unit. We had found that 2-azidobenzimidazole derivatives such as **2** undergo regioselective 6-oxygenation when irradiated in the presence of Brønsted acids [7,8]. An example is the reaction of **2** with Boc-protected alanine that afforded aryl ester **3** in 63% yield. We installed fluoro substituents at the benzimidazole ring for being able to detect photoproducts by ^19^F NMR [9]. It is our goal to covalently attach photohemiasterlin to tubulin and to analyse the fragments via MS^n^. Ideally, the molecule will bind as predicted, and, perhaps, there will be more targets than currently known. The poster communication describes the synthesis of all possible mono-, di-, and trifluorinated derivatives of 2-azido-1-methylbenzimidazole that are unsubstituted at C6. Irradiation experiments in the presence of amino acid-based reaction partners will be presented that provided photoarylation products. Finally, the synthesis and photoreactivity of photohemiasterlin **1** will be discussed (Figure 47).

References

1. Talpir, R.; Benayahu, Y.; Kashman, Y. Two new cytotoxic peptides from the marine sponge *Hemiasterella minor* (Kirkpatrick). *Tetrahedron Lett.*
**1994**, *35*, 4453–4456.
2. Crews, P.; Farias, J.J.; Emrich, R.; Keifer, P.A. Milnamide A, an unusual cytotoxic tripeptide from the marine sponge *Auletta* cf. *constricta*. *J. Org. Chem.*
**1994**, *59*, 2932–2934.
3. Coleman, J.E.; Dilip de Silva, E.; Kong, F.; Andersen, R.J. Cytotoxic peptides from the marine sponge *Cymbastela* sp. *Tetrahedron*
**1995**, *51*, 10653–10662.
4. Anderson, H.J.; Coleman, J.E.; Roberge, M.; Andersen, R.J. Cytotoxic peptides hemiasterlin, hemiasterlin A and hemiasterlin B induce mitotic arrest and abnormal spindle formation. *Cancer Chemother. Pharmacol.*
**1996**, *39*, 10653–10662.
5. Ravi, M.; Zask, A.; Rush, T.S., III. Structure-Based Identification of the Binding Site for the Hemiasterlin Analogue HTI-286 on Tubulin. *Biochemistry*
**2005**, *44*, 15781–15879.
6. Lang, J.H.; Jones, P.G.; Lindel, T. Total synthesis of the marine natural product hemiasterlin via organocatalyzed hydrazination. *Chem. Eur. J.*
**2017**, *23*, 12714–23717.
7. Sudakow, A.; Jones, P.G.; Lindel, T. Photochemical arylation of Brønsted acids with 2-azidobenzimidazole. *Eur. J. Org. Chem.*
**2012**, 681–684.
8. Sudakow, A.; Papke, U.; Lindel, T. Water-compatible photoarylation of amino acids and peptides *Chem. Eur. J.*
**2014**, *20*, 10223–10226.
9. Kanitz, N.E.; Lindel, T.*Z.* Photoreactivity of monofluorinated 2-azidobenzimidazoles towards carboxylic acids. *Naturforschung*
**2016**, *71*, 1287–1300.

#### **Studies towards the Total Synthesis of Dankastatin C** 

MendeSteffenLindelThomasTU Braunschweig, Institute of Organic Chemistry, 38126 Braunschweig, Germany

**Figure 48:**
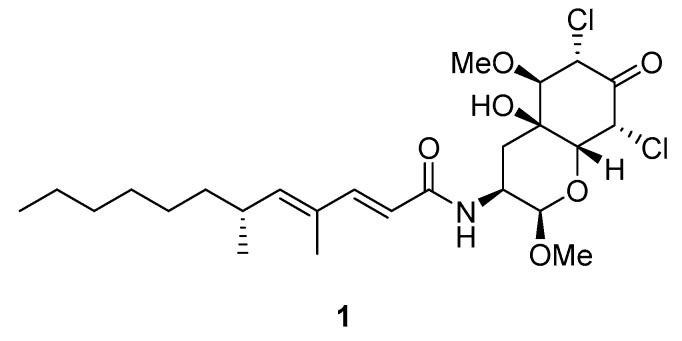
Polyketide derived tyrosine derivative dankastatin C.

The fungus *Gymnascella dankaliensis* was isolated from the sponge *Halichondria japonica*, collected in the Osaka Bay of Japan. Investigation of secondary metabolites produced by the fungus yielded dankastatin C (**1**) in 2013 (Figure 48). This polyketide tyrosine derivative exhibited an ED_50_ value of 57 ng/mL against the murine P388 lymphocytic leukemia cell line [1].

**Figure 49:**
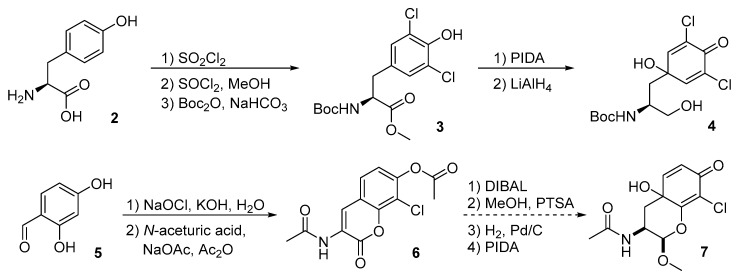
Assembly of a simplified coumarin system of dankastatin C.

Our approach towards dankastatin C (**1**) intends to connect the aliphatic chain with the bicyclus at the end of the synthesis. The synthesis of the side chain, which occurs also in the structures of gymnastatin A-H and aranorosin, is already known [2]. The challenging part will be the preparation of the dichlorinated bicyclus. One way is to begin with l-tyrosine (**2**) and to create a Michael system, followed by ring closure (Figure 49). Thereby, the formation of the chromane-derived framework will be in competition with the spiro compound. Another possibility is to construct coumarin **6** from aldehyde **5** followed by reduction, hydration, and PIDA oxidation.

References

1. Amagata, T.; Tanaka, M.; Yamada, T.; Chen, Y.-P.; Minoura, K.; Numata, A. Additional cytotoxic substances isolated from the sponge-derived *Gymnascella dankaliensis. Tetrahedron Lett.*
**2013**, *54*, 5960–5962.
2. Raffier, L.; Piva, O. Application of the diastereoselective photodeconjugation of α,β-unsaturated esters to the synthesis of gymnastatin H. *Beilstein J. Org. Chem.*
**2011**, 7, 151–155.

#### **Towards the Total Synthesis of Hemicalide: Synthesis of the C27–C46 Region** 

StockdaleTegan P.HanBing YuanLamNelson Y. S.GoodmanJonathan M.PatersonIanUniversity Chemical Laboratory, University of Cambridge, Lensfield Road, Cambridge CB2 1EW, UK

**Figure 50:**
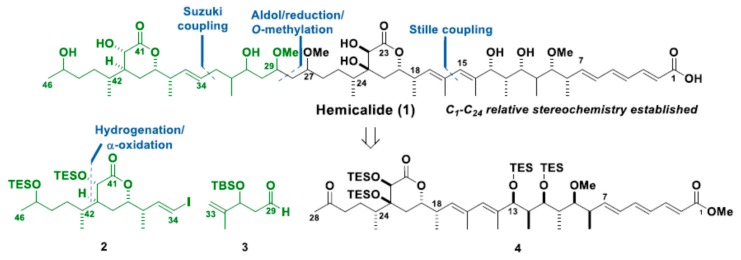
Hemicalide (**1**), with relative configuration and retrosynthesis overview.

The marine natural product hemicalide (**1**, Figure 50), isolated from the *Hemimycales* sp. sponge, displays impressively potent bioactivity, registering picomolar IC50 values against numerous cancer cell lines [1]. Preliminary biological evaluation indicated a novel antimitotic mechanism of action, involving microtubule destabilisation. While 1D- and 2D-NMR experiments established the planar 46-carbon structure, the low isolation yield (0.5 mg) precluded full stereochemical assignment and further biological studies. Previous synthetic and computational studies by our group, and the Ardisson and Cossy groups, have determined the relative configuration within the C1–C28 and C34–C46 stereoclusters [2–6]. Notably, we recently narrowed the possible configurations of the C1–C24 region to a single diastereomer (**4**) [7]. As part of our ongoing interest in the total synthesis-enabled stereochemical elucidation of hemicalide, this work presents progress on the synthesis of the C34–C46 (**2**) and C27–C33 (**3**) fragments. We devised a highly flexible approach to configure diastereomers of the, as yet, unknown stereochemical nature of the C29–C33 region (**3**) via a stereoselective aldol/reduction/*O*-methylation sequence. Construction of the C34–C46 hydroxylactone moiety proceeds via a series of diastereoselective aldol reactions, with asymmetric reduction of C39 and α-hydroxylation of C40 as key steps. Our highly diastereoselective approach enables rapid access to either enantiomers of **2** and **3**, important for ascertaining the relative configuration between each fragment.

References

1. Carletti, I.; Debitus, C.; Massiot, G. Molécules polykétides comme agents anticancéreux. *Pat. Appl. Pub.*
**2011**, *154*, 5130950.
2. Smith, S.G.; Goodman, J.M. Assigning stereochemistry to single diastereoisomers by GIAO NMR calculation: The DP4 probability. *J. Am. Chem. Soc.*
**2010**, *132*, 12946.
3. Fleury, E.; Lannou, M.-I.; Bistri, O.; Sautel, F.; Massiot, G.; Pancrazi, A.; Ardisson, J. Relative Stereochemical Determination and Synthesis of the C1–C17 Fragment of a New Natural Polyketide. *J. Org. Chem.*
**2009**, *74*, 7034.
4. MacGregor, C.I.; Han, B.Y.; Goodman, J.M.; Paterson, I. Toward the stereochemical assignment and synthesis of hemicalide: DP4f GIAO-NMR analysis and synthesis of a reassigned C16–C28 subunit. *Chem. Commun.*
**2016**, *52*, 4632.
5. Lecourt, C.; Boinapally, S.; Dhambri, S.; Boissonnat, G.; Meyer, C.; Cossy, J.; Sautel, F.; Massiot, G.; Ardisson, J.; Sorin, G.; Lannou, M.-I. Elaboration of Sterically Hindered δ-Lactones through Ring-Closing Metathesis: Application to the Synthesis of the C1–C27 Fragment of Hemicalide. *J. Org. Chem.*
**2016**, *81*, 12275.
6. Specklin, S.; Boissonnat, G.; Lecourt, C.; Sorin, G.; Lannou, M.-I.; Massiot, G.; Meyer, C.; Cossy, J. Synthetic Studies toward the C32–C46 Segment of Hemicalide. Assignment of the Relative Configuration of the C36–C42 Subunit. *Org. Lett.*
**2015**, *17*, 2446.
7. Han, B.Y.; Lam, N.Y.S.; MacGregor, C.I.; Goodman, J.M.; Paterson, I. A synthesis-enabled relative stereochemical assignment of the C1–C28 region of hemicalide. *Chem. Comm.*
**2018**, *54*, 3247.

#### **Preparation of Chemical Probes from Marine Natural Products** 

KamihiraRie^1^NakaoYoichi^1^,^2^1Waseda Research Institute for Science and Engineering, Waseda University, 3-4-1 Okubo, Shinjuku-ku, Tokyo 169-8555, Japan2Graduate School of Advanced Science & Engineering, Waseda University, 3-4-1 Okubo, Shinjuku-ku, Tokyo 169-8555, Japan

**Figure 51:**
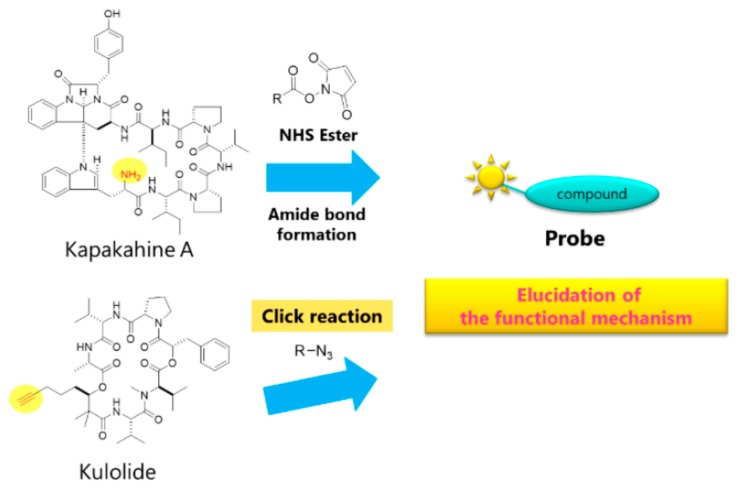
The schematic drawing of the study.

Marine organisms are known as the source of bioactive natural compounds. Numerous bioactive marine compounds have been isolated, but it is still difficult to disclose their modes of actions. To study the modes of actions, preparation of the chemical probes based on the natural products is the important process. Fluorescent probes are powerful tools to identify the localizing sites of the bioactive compounds in the cells. Also, affinity beads linked to the chemical probes are essential for targeting the binding proteins. To add more successful cases identifying the target proteins and the modes of actions, we turned to marine natural products including kapakahine A [1] and kulolide [2]. Kapakahine A contains an amino group which can react with the *N*-hydroxysuccinimide (NHS). Kulolide contains a terminal acetylene which can react with the azide group in the “click reaction”, which is the simple and efficient way to prepare the chemical probes (Figure 51). Preparation of chemical probes from marine natural products utilizing their functional groups as well as their application to the cell imaging will be presented in this poster.

References

1. Yeung, B.K.S.; Nakao, Y.; Kinnel, R.B.; Carney, J.R.; Yoshida, W.Y.; Scheuer, P.J. The Kapakahines, cyclic peptides from the marine sponge *Cribrochalina olemda.*
*J. Org. Chem*. **1996**, *61*, 7168–7173.
2. Reese, M.T.; Gulavita, N.K.; Nakao, Y.; Hamann, M.T.; Yoshida, W.Y.; Coval, S.J.; Scheuer, P.J. Kulolide:  a cytotoxic depsipeptide from a cephalaspidean mollusk, *Philinopsis speciosa.*
*J. Am. Chem. Soc.*
**1996**, *118*, 11081–11084.

### ***Theme 6. Marine Natural Products Chemical Biology and Biotechnology*** 

#### **Anticancer Potential of Chromomycins in Breast Cancer Cells: Modulation of T-Box2 Transcription Factor and DNA Damage Induction** 

Rezende-TeixeiraPaula^1^BerraCarolina M.^2^SahmBianca Del Bianco^1^BrancoPaola C.^1^AlmeidaLarissa Costa de^1^Machado-SantelliGláucia M.^3^Costa-LotufoLetícia V.^1^1Department of Pharmacology, Institute of Biomedical Sciences, University of Sao Paulo, Sao Paulo 005508-000, Brazil2Genomics and Molecular Biology Laboratory, International Research Center, A. C. Camargo Cancer Center, Sao Paulo 01525-001, Brazil3Department of Cell Biology and Development, Institute of Biomedical Sciences, University of Sao Paulo, Sao Paulo 05508-000, Brazil

Drug-resistance is the leading cause in the failure of cancer chemotherapy, stimulating the search for new drugs that overcome resistance. Chromomycins are compounds that selectively bind to dsDNA, altering replication and transcription processes. Preliminary results from bioaffinity chromatography and thermophoresis obtained at the Laboratory of Pharmacology of Marine Natural Products (LaFarMar-ICB/USP) suggest that chromomycin A5 (CA5) binds with high affinity to TBX-2. TBX-2 is a transcription factor described as a potent growth factor associated with embryonic development, however in many cancers are overexpressed, including the most aggressive forms of breast cancers. These results are quite interesting because they imply that CA5 may act as both an inducer of DNA damage and a TBX-2 ligand, making the availability of this transcription factor impaired, affecting cell proliferation. Thus, the main objective of this work is to study the activity of CA5 in breast tumor cells with different genotypes (MCF7, T47D, MDAMB231 and Hs578T) to verify if the treatment can alter the availability of T-box factors. The cytotoxic activity of CA5 showed IC_50_ values nM range for 72 h exposure. Treatment with IC_50_ and IC_50_/2 values decreased the TBX2 mRNA expression and increased of H2AX, evidencing the ability of CA5 to induce DNA damage. Morphological alterations and G0/G1 cell cycle arrest were induced by treatment with CA5. It is important to note that the most sensitive cells, MDAMB231 and MCF7, present mutations in genes involved in repair by homologous recombination and, apparently, these mutations sensitize cells more efficiently to CA5 effects.

##### **Funding** 

FAPESP (2015/17177-6), ArboControl Brasil Project (FNS/UnB TED74/2016 and TED42/2017).

#### **The Effects of Prodigiosin on TGF-β-Induced Cellular Responsiveness** 

ChungChih-Ling^1^SungPingJyun^2^,^3^WenZhi-Hong^4^ChenChun-Lin^1^1Department of Biological Sciences, National Sun Yat-sen University, Kaohsiung 80424, Taiwan2Graduate Institute of Marine Biology, National Dong Hwa University, Hualien 97401, Taiwan3National Museum of Marine Biology and Aquarium, Pingtung 944, Taiwan4Department of Marine Biotechnology and Resources, National Sun Yat-sen University, Kaohsiung 80424, Taiwan

Prodigiosin (PG), a group of red pigments first isolated from marine bacteria *Serratia marcescens*, has been showed to promote apoptosis in several human cancer cell line and display antimetastatic property. Those anticancer affects are approached by uncoupling vacuolar-type H+-ATPases (V-ATPases) through promoting H+/Cl− symport, eventually inhibiting lysosomal acidification. Transforming growth factor-β (TGF-β), a multiple cytokine, is known to play important roles in pathological process such as cancer development and tissue fibrosis. As TGF-β changes its role from tumor suppressor to promoter during tumorigenesis, exploring anticancer reagent targeting TGF-β is crucial. In this study, PG is firstly reported to induce type II TGF-β receptor (TβRII) expression. PG promotes TβRII accumulation in the perinuclear region in Mv1Lu cells at non-cytotoxic concentration. Our data show that PG trigger TβRII and Integrin β1 translocation to non-raft fractions. Interestingly, the increasing level of TβRII does not promote TGF-β/SMAD signaling transduction. Furthermore, we find that PG treatment enhance TGF-β-stimulated SMAD phosphorylation in mouse fibroblast (NIH-3T3 cells). Taken together, the results suggest further investigation for roles of PG in TGF-β signaling is warranted. The non-SMAD pathway should be analyzed in both Mv1Lu cells and NIH-3T3 cells, and the effects of PG on TGF-β-induced cell migration should be investigated as well.

#### **Molecular Mechanism of Resistance: Mutation of Actin in *Chromodoris* Sea Slugs Enable the Sequestration and Storage of Cytotoxic Latrunculin A** 

HertzerCora^1^UndapNani^2^,^3^AatzStefan^4^FrankenSebastian^4^HäberleinHanns^4^BhandariDhaka Ram^5^SpenglerBernhard^5^BöhringerNils^6^KehrausStefan^1^BaraRobert^3^IjongFrans Gruber^7^WägeleHeike^2^SchäberleTill F.^6^KönigGabriele M.^1^1Institute of Pharmaceutical Biology, Rheinische Friedrich-Wilhelms-University, 53113 Bonn, Germany2Zoological Research Museum Alexander Koenig, Leibniz Institute for Animal Biodiversity, Bonn, Leibniz Institute for Animal Biodiversity, 53113 Bonn, Germany3Faculty of Fisheries and Marine Science, Sam Ratulangi University, 95115 Manado, Indonesia4Institute of Biochemistry and Molecular Biology, Rheinische Friedrich-Wilhelms-University, 53113 Bonn, Germany5Institute of Inorganic and Analytical Chemistry, Justus-Liebig-University, 35390 Giessen, Germany6Institute for Insect Biotechnology, Justus-Liebig-University, 35390 Giessen, Germany7Politeknik Nusa Utara, Tahuna, Sulawesi Utara 95812, Indonesia

Chromodorid nudibranchs (Gastropoda, Mollusca) live and feed upon noxious sponges, from which they sequester defensive compounds to combat competition, pathogens, and predators.

Here we investigate secondary metabolites of six *Chromodoris* species (Chromodorididae, Doridoidea). Comparison of the individual extracts led to HPLC isolation of the cytotoxic 2-thiazolidinone macrolide Latrunculin A (LatA, 0.4 mg/specimen), identified by HR-ESI-MS, 1D and 2D NMR spectroscopy. LatA was the major metabolite in all examined *Chromodoris* species. Additionally, it was isolated from the associated sponge *Cacospongia mycofijiensis* (Thorectidae, Porifera), supporting a dietary origin of LatA. MALDI MS-Imaging revealed that LatA is stored specifically throughout the mantle tissue, mantle dermal formations (MDFs), and mucus glands. So far, only slugs of the genus *Chromodoris* are known to sequester LatA, indicating a unique resistance mechanism of this group.

LatA’s cytotoxicity is a direct result of its binding to globular actin-monomers, preventing their polymerization. We identified two crucial actin mutations causing amino acid substitutions D187G and R206T by genetic analysis of the *Chromodoris* actin gene sequences.

Based on these results, we hypothesize that the amino-acid substitutions D187G and R206T in *Chromodoris* actin are the underlying cause of their resistance to LatA. To examine this hypothesis, we currently conduct genome-editing and fluorescence microscopy experiments of HEK-293 cells.

The resistance to LatA, caused by D187G/R206T, would enable *Chromodoris* slugs to sequester the toxin from their prey *C. mycofijiensis*. Furthermore, this would allow them to store LatA in the mantle tissue and use it for their defence, without having to suffer from its cytotoxicity.

#### **Pentabromopseudilin, a Marine Antibiotic Inhbits Transforming Growth Factor-Beta (TGF-β) Activity by Accelerating the Receptor Turnover** 

ChungChih-Ling^1^KnölkerHans-Joachim^2^ChenChun-Lin^1^1Department of Biological Sciences, National Sun Yat-sen University, Kaohsiung 80424, Taiwan2Department of Chemistry, TU Dresden, 01069 Dresden, Germany

Pentabromopseudilin (PBrP) is a marine antibiotic isolated from the marine bacteria Pseudomonas bromoutilis and Alteromonas luteoviolaceus. PBrP exhibits antimicrobial, antitumor, and phytotoxic activities. Because PBrP selectively alters the motor activity of myosin-Va (MyoVa), it has strong potential for studying cell biological processes and potential therapeutic effects. In this study, we demonstrated that PBrP inhibited transforming growth factor-β (TGF-β)-stimulated luciferase activity with an EC50 value of 0.1–0.2 μM in cultured cells expressing Smad-dependent luciferase reporter. PBrP inhibited the TGF-β-induced expression of pSmad2 and PAI-1 proteins. Furthermore, PBrP ameliorated the TGF-β-mediated suppression of E-cadherin expression, induction of N-cadherin expression, and vimentin and fibronectin upregulation, thereby blocking TGF-β-induced epithelial–mesenchymal transition in A549 cells. PBrP reduced the cell-surface expression of type II TGF-β receptor (TβRII), followed by receptor degradation, as determined by cell-surface biotinylation/Western blot analysis. These findings suggest that intracellular accumulation promotes receptor degradation. Gene silencing approaches revealed that MyoVa may play a crucial role in PBrP-induced TβRII turnover and the subsequent reduction of TGF-β signaling. Because TGF-β signaling is crucial in the regulation of diverse biological processes such as cell plasticity and migration and cancer development and progression, PBrP should be further explored for its therapeutic role in treating fibrotic disease and cancer.

#### **A Marine Sponge Sesquiterpene Quinone Induces Apoptosis in Breast Cancer Cells** 

SuJui-Hsin^1^WengJing-Ru^2^1National Museum of Marine Biology and Aquarium, Pingtung 94450, Taiwan2Department of Marine Biotechnology and Resources, National Sun Yat-sen University, Kaohsiung 80424, Taiwan

Breast cancer is one of the top ten causes of death in Taiwan, and the mortality rate continued to increase in recent ten years. In our search for potential anti-tumor agonists, a marine sesquiteroene quinone (Ilimaquinone, IQ), isolated from sponge *Halichondria* sp. The cell viability of IQ was assessed by MTT assay, and the results showed that a concentration- and time-dependent manner in inhibiting breast cancer cell growth. Flow cytometric analysis demonstrated that IQ increased the percentages of apoptotic cells in breast cancer cells. In addition, IQ modulated the phosphorylation/expression of Bcl-xL, Bax, and Bcl-2. In conclusion, these findings provided the rationale for using this triterpenoid for the further development of breast cancer therapy.

#### **Potential of *Sphaerococcus coronopifolius* Terpenes as Therapeutic Agents for Infectious Diseases** 

FreitasRafaela^1^AlvesCelso^1^SilvaJoana^1^MartinsAlice^1^PinteusSusete^1^RibeiroJoana^1^DuarteAdriana^1^GasparHelena^2^PedrosaRui^1^1MARE—Marine and Environmental Sciences Centre, ESTM, Instituto Politécnico de Leiria, 2520-641 Peniche, Portugal2University of Lisbon, Faculty of Science, BioISI—Biosystems and Integrative Sciences Institute, 1749-016 Lisbon, Portugal

Nowadays, infectious diseases associated with resistance to antimicrobial agents are one of the major challenges for our society. It is estimated that around 700,000 people die each year due to this burden, however, this number could reach 10 million deaths by 2050. Therefore, the urgent need for a new generation of antimicrobial agents is of utmost importance. Marine environment has proven to be an interesting source of new structures, with unique characteristics not found in terrestrial sources that can be used for molecular modeling of new antibiotics. Thus, the aim of this study was to evaluate the antimicrobial potential of six terpenes isolated from the red alga *Sphaerococcus coronopifolius* against distinct human pathogenic microorganisms. Compounds (sphaerococcenol A, 12*S*-hydroxy-bromosphaerol, 12*R*-hydroxy-bromosphaerol, bromosphaerol, sphaerodactylomelol, alloaromadendrene) were isolated by HPLC and identified by NMR spectroscopy. The antimicrobial activities (200 μM) were evaluated against *Aspergillus fumigatus* (filamentous fungus), *Cryptococcus neoformans* (yeast), *Malassezia furfur* (yeast), *Cutibacterium acnes* (Gram +), *Staphylococcus epidermidis* (Gram +), *Klebsiella pneumoniae* subsp. *ozaenae* (Gram −) and *Salmonella enterica* (Gram −) through the readings of optical density at 600 nm. For the most potent compounds (microorganism growth inhibition > 50%), the IC_50_ was determined (0.1–200 μM).

Results revealed that 12*R*-hydroxy-bromosphaerol exhibited potent antimicrobial activity against several microorganisms, such as *A. fumigatus*, *C. neoformans* [IC_50_ = 18.52 μM (14.00–24.49)], *C. acnes* [IC_50_ = 8.75 μM (6.51–11.77)] and *S. epidermidis* [IC_50_ = 5.61 μM (4.18–7.53)], suggesting that this compound is the most promising one as an antimicrobial agent.

##### **Acknowledgments** 

This work was supported by the Portuguese Foundation for Science and Technology (FCT) through Strategic Projects UID/MAR/04292/2013 and UID/Multi/04046/2019 granted to MARE—Marine and Environmental Sciences Centre, and BioISI—BioSystems and Integrative Sciences Institute, respectively, through Red2Discovery Project (PTDC/MAR-BIO/6149/2014), co-financed by COMPETE (POCI-01-0145-FEDER-016791), through Oncologia de Precisão: Terapias e Tecnologias Inovadoras project (POINT4PAC) (SAICTPAC/0019/2015—LISBOA-01-0145-FEDER-016405) and through CrossAtlantic Project (PTDC/BIA-OUT/29250/2017), co-financed by COMPETE (POCI-01-0145-FEDER-029250).

#### **Interaction between Okadaic Acid and Neuropeptide Y: In Vivo Effects** 

CostasCelia^1^AbalPaula^1^LouzaoM. Carmen^1^VilariñoNatalia^1^VieytesMercedes R.^2^BotanaLuis M.^1^1Departamento de Farmacología, Facultad de Veterinaria, Universidad de Santiago de Compostela, 27002 Lugo, Spain2Departamento de Fisiología, Facultad de Veterinaria, Universidad de Santiago de Compostela, 27002 Lugo, Spain

Diarrheic shellfish poisoning (DSP) is one of the most common and extended intoxications caused by marine biotoxins in Europe. The symptomatology is characterised by affecting mainly the gastrointestinal tract, producing diarrhoea, vomiting, nausea and abdominal pain. Okadaic acid (OA) is the reference compound of its group of biotoxins and one of the responsible of DSPs. It is well described the inhibition of protein phosphatases PP1 and PP2A by OA and its analogues. Nevertheless, this mechanism of action doesn’t fully explain the symptomatology caused by the toxin, since other biotoxins potent inhibitors of PPs do not cause diarrhoea. On the other hand, mice treated with OA show not only digestive affections, but neurological signs, such as spasms. Previous in vitro studies of our group indicate a possible effect of the biotoxin over neuropeptide Y (NPY). To further elucidate this effect in vivo, mice were treated by oral gavage with OA and showed diarrhoea as the main symptom. Necropsy indicated that OA led to fluid accumulation in the intestines where changes in NPY were detected. Meanwhile those effects were partially reduced when mice were also treated with NPY. These preliminary results open the possibility that OA could affect NPY and consequently trigger diarrhoea.

#### **Marine Natural Products That Control a Specific Set of Histone Modifications** 

IkedaKotaro^1^AraiDaisuke^1^MachidaKoshi^2^Hayashi-TakanakaYoko^3^KimuraHiroshi^4^FusetaniNobuhiro^2^,^5^NakaoYoichi^1^,^2^1Department of Chemistry and Biochemistry, Graduate School of Advanced Science and Engineering, Waseda University Tokyo 169-8555, Japan2Research Institute for Science and Engineering, Waseda University, Tokyo 169-8555, Japan3Graduate School of Frontier Biosciences, Osaka University, Suita 565-0871, Japan4Cell Biology Center, Institute of Innovative Research, Tokyo Institute of Technology Yokohama 226-8503, Japan5Fisheries and Oceans Hakodate, Hakodate 041-8611, Japan

Genomic DNA of eukaryotes exists in the form of nucleosome, a structure consisting of DNA and core histone proteins. Several amino acid residues on *N*-terminal tail regions of histone proteins are targets of post-translational modification, including methylation and acetylation. Histone modifications control genomic functions in various ways, and thus are deeply involved in onset and/or progression of chronic diseases such as cancer, attracting attention as a therapeutic target.

We have explored marine natural products that change histone modifications in living cells. The activities were evaluated by immunofluorescense using specific monoclonal antibodies against each histone modification. As a result, we identified topsentin (**1**) [1] and bromotopsentin (**2**) [1] as active compounds. Compounds **1** and **2** share the same carbon skeletons and the only difference is one hydroxyl group in **1** is substituted to a bromo group in **2**. On the other side, **1** and **2** exerted specific effects on histone modifications in HeLa cells, respectively, indicating their different modes of action. We present activities of **1** and **2** on a total of 20 types of histone modifications and discuss potential of these compounds as epigenetic drugs.

References

1. Bartik, K.; Braekman, J.; Daloze, D.; Stoller, C.; Huysecom, J.; Vandevyver, G.; Ottinger, R. Topsentins, new toxic bis-indole alkaliods from the marine sponge *Topsentia genitrix*. *Canad. J. Chem*. **1987**, *65*, 2118–2121.

#### **Activity of *Fucus vesiculosus* (Bodelha) from the Tagus Estuary (Lisbon, Portugal) as Antiacetilcolinesterase and Antioxidant** 

NunesDiogo^1^,^2^RessaissiAsma^1^MeloRicardo^2^,^3^SerralheiroMaria Luísa M.^1^,^4^1BioISI, Faculdade de Ciências, Universidade de Lisboa, Campo Grande, 1749-016 Lisboa, Portugal2Departamento de Biologia Vegetal, Faculdade de Ciências, Universidade de Lisboa, Campo Grande, 1749-016 Lisboa, Portugal3MARE-Marine and Environmental Sciences Centre, Faculdade de Ciências, Universidade de Lisboa, Campo Grande, 1749-016 Lisboa, Portugal4Departamento de Química e Bioquímica, Faculdade de Ciências, Universidade de Lisboa, Campo Grande 1749-016 Lisboa, Portugal

*Fucus vesiculosus L*. (bladderwrack or bodelha) is a brown macroalga (Phaeophyceae, Fucaceae) distributed along the Portuguese coast. This common alga is reported to have several traditional uses with defined dosages [1]. The beneficial effects for health are attributed to different types of biological macromolecules. The less studied compounds are the phlorotannins, produced generally by the brown algae [2]. Phlorotannins are synthesized by polymerization of phloroglucinol produced via the shikimate pathway [3] and seem to have defense functions against ROS formed during photosynthesis [4]. In this study, samples from *F. vesiculosus* were taken from the Tagus river estuary (Lisbon, Portugal), and grouped by thallus size (indicator of age) and gender. Aqueous extracts from these samples were then prepared and analyzed for their phlorotannin concentration and antioxidant activity and correlated between classes. Phlorotannins were quantified by the Folin-Ciocalteu method and determined as an equivalent of gallic acid. In young thalli, males have a higher phenolic content whereas in adult stages the amount of phlorotannins is approximately the same. Antioxidant activity was determined as the capacity to scavenge free radicals like DPPH. Antioxidant activity was also higher in young male thalli, while achieving approximate values in adult stages. Enzymatic activity towards acetylcholinesterase was also carried out, analyzing samples that obtained the best results from the previous assays. Lastly, a High-Performance Liquid Chromatography was conducted to achieve a qualitative and quantitative analysis of the alga’s components.

References

1. EMA/HMPC/313675/2012. Available online: www.ema.europa.eu/en/medicines/herbal/fucus-vesiculosus-thallus (accessed on 26 May 2019).
2. Wells, M.L.; Potin, P.; Craigie, J.S.; Raven, J.A.; Merchant, S.S.; Helliwell, K.E.; Smith, A.G.; Camire, M.E.; Brawley, S.H. Algae as nutritional and functional food sources: revisiting our understanding. *J. Appl. Phycol.*
**2017**, *29*, 949–982.
3. Salminen, J.P.; Karonen, M. Chemical ecology of tannins and other phenolics: we need a change in approach. *Funct. Ecol.*
**2011**, *25*, 325–338.
4. Pangestuti, R.; Siahaan, E.A.; Kim S.-K.. Photoprotective Substances Derived from Marine Algae. *Mar. Drugs*
**2018**, *16*, 399.

#### ***Fucus spiralis* Extract Inhibits α-Amylase and α-Glucosidase In Vitro: A New Marine Product with Antidiabetic Potential?** 

DuarteA. M.^1^HortaA.^1^SilvaF.^2^BarrosoS.^1^GuarinoM. P.^3^MendesS.^1^GilM. M.^1^1MARE—Marine and Environmental Sciences Centre, Instituto Politécnico de Leiria, 2520-630 Peniche, Portugal2MARE—Marine and Environmental Sciences Centre, Faculdade de Ciências da Universidade de Lisboa, 1749-016 Lisboa, Portugal3ciTechCare—Center for Innovative Care and Health Technology, Instituto Politécnico de Leiria, 2410-197 Leiria, Portugal

Type 2 Diabetes Mellitus (T2DM) is a chronic disease that corresponds to 90% of the worldwide cases of diabetes. First line therapeutic approaches are based on lifestyle changes complemented with medication associated with several side effects and high costs. The scientific community has been working in drug discovery from natural sources that provide lower financial impact and fewer side effects. The inhibition of digestive enzymes by polyphenolic compounds has been highlighted as a therapeutic strategy, allowing postprandial hyperglycemia reduction by delaying digestion of carbohydrates. This study investigated whether a polyphenol-rich alga (*Fucus spiralis*) extract inhibits the digestive enzymes α-amylase and α-glucosidase. Extraction condition for obtaining the highest antioxidant activity and total phenolic content (TPC) of *Fucus spiralis* were optimized. Response surface methodology considering a central composite rotatable design was carried out to optimize the extraction conditions. The maximum antioxidant activity (DPPH IC_50_ 4.18; FRAP 547.03 mg AAE/g) and TPC, 80.83 mg GAE/g, was obtained with the following optimum extraction conditions: 26% of seaweed, 100% water and 300 s of homogenization. Additionally, optimized nonalcoholic extract at 1000 to 250 μg/mL in sodium phosphate/potassium phosphate buffer was screened for it α-amylase and α-glucosidase inhibitory activity through a spectrophotometric method, using colorimetric DNS and PNPG reagents, respectively and acarbose as positive. Maximal inhibition was achieved with seaweed extract with 250 μg/mL concentration, by 28.4 ± 0.04% (acarbose 76.0 ± 0.03%) for α-amylase and by 98.5 ± 0.002% (acarbose 55.0 ± 0.04%) for α-glucosidase. We concluded that *Fucus spiralis* extract inhibits α-amylase and α-glucosidase, two therapeutic targets in T2DM.

#### **Anti-Aging Activity of *Lobophora variegata* Ethanolic and Methanolic Extracts and Their Fractions** 

RosaG. P.^1^CostaA.^1^MedeirosD.^1^SecaA. M. L.^2^,^3^BarretoM. C.^2^1Faculty of Sciences and Technology, University of Azores, Rua Mãe de Deus, 9501-801 Ponta Delgada, Portugal2cE3c—Centre for Ecology, Evolution and Environmental Changes/Azorean Biodiversity Group & University of Azores, Rua Mãe de Deus, 9501-801 Ponta Delgada, Portugal3QOPNA & LAQV-REQUIMTE, University of Aveiro, 3810-193 Aveiro, Portugal

Seaweed have promising applications within food, cosmetic and health industries, which led to an increased interest in studying these organisms [1]. In several coastal areas, thousands of tons of macroalgae are cast on beaches and shorelines and it would be very interesting if this biomass could be managed, allowing the extraction of added-value compounds. In this context, polar extracts (methanol and ethanol) of a macroalgal beach cast sample mainly composed of *Lobophora variegata* were prepared and the anti-aging and antioxidant activities were evaluated. The preliminary results showed interesting results, and thus these crude extracts were then fractionated sequentially by their solubility in dichloromethane, acetone and ethyl acetate, resulting in 4 semi-pure fractions each, which were also tested. Fractions A1.1.1 and A1.2.3 were very good tyrosinase inhibitors (IC_50_ = 37.87 and 24.01 μg/mL, respectively) and fractions A1.1.2 and A1.2.2 presented very good inhibition of elastase (IC_50_ = 44.76 and 20.86 μg/mL, respectively). However, none of the fractions was active against collagenase. These results show that further purifications of these fractions can lead to the isolation of bioactive added-value compounds.

##### **Acknowledgments** 

Study and G. Rosa’s grant financed by: MACBIOBLUE project (MAC/1.1b/086, Interreg Mac 2014–2020 and DRCT); FCT—Fundação para a Ciência e a Tecnologia, the European Union, QREN, FEDER, COMPETE, by funding the cE3c centre (FCT Unit funding (Ref. UID/BIA/00329/2013, 2015–2018) and UID/BIA/00329/2019) and the QOPNA research unit (project FCT UID/QUI/00062/2019).

References

1. Loureiro, C.; Medema, M.H.J.; van der Oost, J.; Sipkem, D. Exploration and exploitation of the environment for novel specialized metabolites. *Curr. Opin. Biotechnol*. **2018**, *50*, 206–213.

#### **Toward the Discovery of New Antimicrobial Agents from Underexplored Marine-Derived Fungi in Egypt** 

HamedAhmed A.^1^,^2^SoldatouSylvia^2^QaderM. Mallique^3^PavesiCoralie^2^MirandaKevin Jace^2^IbrahimNabil A.^4^AbdelazizMohamed S.^1^EidBasma^4^GhareebMosaad A.^5^RatebMostafa E.^3^EbelRainer^2^1Microbial Chemistry Department, National Research Centre, 33 El-Buhouth Street, P.O. Box 12622, Dokki, Giza, Egypt2Marine Biodiscovery Centre, Department of Chemistry, University of Aberdeen, Aberdeen AB24 3UE, Scotland, UK3School of Computing, Engineering & Physical Sciences, University of the West of Scotland, Paisley PA1 2BE, UK4Textile Research Division, National Research Centre, Scopus Affiliation ID 60014618, 33 EL Buhouth St., Dokki, Giza, P.O. 12622, Egypt5Medicinal Chemistry Department, Theodor Bilharz Research Institute, Kornaish El Nile, Warrak El-Hadar, Imbaba (P.O. 30), Giza 12411, Egypt

Historically, natural products have been used for the treatment of many diseases since ancient times. One of the earliest records of medicinal use of natural products dating back to the ancient Egyptian civilization (pharaohs). As an Egyptian pharmaceutical record, the ancient Egyptian medical Ebers Papyrus (2900 B.C.), in which over 700 plant-based drugs were recorded for the treatment of many diseases. In our days, great attention has been given to endophytic fungi due to the chemical diversity of their secondary metabolites. Therefore, this study was undertaken to isolate and identify endophytic and associated fungi from plant and marine organisms collected from two locations in Egypt, Hurghada and Inland saline lakes of Wadi El Natrun. As a result 51 pure fungal strains were isolated, cultivated on different media and their extracts were screened biologically as antimicrobial against some pathogenic microorganism and as antioxidant activity using DPPH. Based on the bioassay data, 15 isolates were exhibited interesting antimicrobial and antioxidant activity. Morphological and genetic studies of the selected isolates have shown that, they are belonging to six genera (*Aspergillus*, *Epicoccum*, *Penicillium*, *Cladosporium*, *Paecilomyces*, *Alternaria*). Based on chemical screening and derelictions by analysis of the LC-MS/MS and H^1^ NMR data, the extracts with promising chemical profile were purified using different chromatographic techniques and pure compounds were evaluated as antimicrobial, antibiofilm against some clinical pathogens. Our main target is to use these bioactive compounds to functionalize the textile to improve comfort, performance and protection properties of fabric include antimicrobial, UV-Protection, self-cleaning and odor control.

#### **Growth Inducing Properties of the Marine Organisms Extracts on Selected Fungal Species** 

CikošAna-Marija^1^ŠarkanjBojan^2^JerkovićIgor^3^MolnarMaja^1^ŠubarićDrago^1^BabićJurislav^1^JokićStela^1^1Josip Juraj Strossmayer University of Osijek, Faculty of Food Technology Osijek, Franje Kuhača 20, Osijek 31000, Croatia2Department of Food Technology, University Center Koprivnica, University North, Trg dr. Žarka Dolinara 1, Koprivnica 48000, Croatia3Faculty of Chemistry and Technology, University of Split, R. Boškovića 35, Split 21000, Croatia

Even though marine organisms are known for their bioactive compounds having beneficial effect on human body, the compounds with opposite activity are also present in such organisms. Therefore, this study aimed to investigate the growth inducing effect of the marine organisms extracts on the tested fungi. Five different marine organisms including *Aplidium conicum*, *Codium bursa*, *Cystoseira* sp. and *Eunicella cavolinii*, all collected in April 2018 from the Adriatic Sea, were used for the extraction process. The extracts of marine organisms were obtained with ultrasound-assisted extraction (UAE) which was performed in the ultrasound bath with two different solvents, water and dimethylsulfoxide (DMSO). Polar compounds were extracted with water, while non-polar compounds were obtained with DMSO. The prepared extracts were used for the testing of growth inducing properties on the most problematic mycotoxigenic fungal species such as *Penicillium expansum*, *Fusarium verticilloides*, *Aspergillues flavus*, *Alternaria alternate*, *Aspergillus ochraceus* and *Fusarium gramniearum.* The standardized microdilution method used for the testing was in accordance with the NCCLS guidelines, document M38-A. According to the results, there is a significant difference in the growth inducing properties on tested fungal species depending on the used extract and their concentration. Some of the extracts showed growth inducing properties at low concentrations (5 and 50 μg/mL), while in some cases the high amount of DMSO or H_2_O extract (5000 μg/mL) promoted growth of the fungi.

#### **Bioactivity Screening of Marine and Freshwater Cyanobacteria for the Isolation of Novel Compounds for Diabetes Type II Using In Vivo and In Vitro Approaches** 

RibeiroTiago^1^RosaFilipa^1^AstrainBegoña Sanchez^1^VasconcelosVítor^1^,^2^ReisMariana^1^UrbatzkaRalph^1^1Interdisciplinary Centre of Marine and Environmental Research (CIIMAR), 4450-208 Matosinhos, Portugal2Faculdade de Ciências, Universidade do Porto, 4169-007 Porto, Portugal

Diabetes, and in particular diabetes type II (DMT2), shows an increase of incidence worldwide and different therapeutics have been studied. Cyanobacteria, known as blue-green algae, are recognized to produce many secondary metabolites including toxins with applications to the human health. The aim of this study is to investigate the effect of cyanobacteria from the CIIMAR in-house cyanobacterial culture collection (LEGEcc) on the increase of glucose uptake using in vivo and in vitro approaches. For the in vitro approach, hepatocarcinoma HepG2 cells were starved in Hank’s Balanced Salt Solution and then exposed to cyanobacterial fractions. For the in vivo approach, 3DPF *Danio rerio* (zebrafish) larvae were exposed to the cyanobacterial fractions. In both assays, the glucose uptake was quantified by the fluorescent dye 2-NBDG. Extraction and production of fractions of cyanobacterial biomass was performed with hexane, ethyl acetate and methanol, either in a polarity gradient extraction or in a sequential extraction. Both assays were optimized to quantify the increase of glucose uptake by the use of known model compounds. The screening of cyanobacteria resulted in the identification of cyanobacterial strains with promising bioactivities. Different results were observed for each approach with more positive results in the in vivo approach. In the ongoing work, we intend to isolate novel bioactive compounds and to elucidate their structures.

##### **Acknowledgments** 

This work was supported by the European ERA-NET Marine Biotechnology project CYANOBESITY (ERA-MBT/0001/2015) and FCT grant SFRH/BD/139131/2018 financed by national funds through FCT (Foundation for Science and Technology, Portugal).

#### **Cytotoxic Activity of Marine Fungal Crude Extracts Isolated from the Portuguese Western Coast** 

da Luz CaladoMaria^1^AlvesCelso^1^SilvaJoana^1^PinteusSusete^1^PedrosaRui^2^GasparHelena^2^,^3^CamposMaria Jorge^2^1MARE—Marine and Environmental Sciences Centre, Instituto Politécnico de Leiria, 2520-641 Peniche, Portugal2MARE—Marine and Environmental Sciences Centre, ESTM, Instituto Politécnico de Leiria, 2520-641 Peniche, Portugal3Universidade de Lisboa, Faculdade de Ciências, BioISI—Biosystems and Integrative Sciences Institute, 1749-016 Lisboa, Portugal

Marine fungi represent an emerging and promising source for the production of new bioactive natural products with biotechnological and pharmacological potential. Given this relevance, 20 marine fungi with distinct nutritional strategies (16 endophytic fungi associated with seaweeds, and 3 and 1 saprobic fungi occurring on driftwood and sea foam respectively), were isolated from four intertidal habitats of the Portuguese Western coast and bioprospected for the production of secondary metabolites with cytotoxic potential. The identification was based on the morphology of fruiting structures and/or sequences of ITS rDNA and revealed that all the fungi belonged to the Ascomycota; among these, three were exclusively marine, i.e., *Corollospora maritima*, *Lindra obtusa* and *Asteromyces cruciatus*. A small-scale fermentation assay was performed with all the isolated fungi, and the crude ethyl acetate extracts were obtained from both culture broth and mycelia. Cytotoxicity of the 20 fungal extracts were tested against MCF-7 cells derived from human breast adenocarcinoma following 24 h exposure, and the effects were revealed by the MTT assay. Extracts that exhibited higher antiproliferative activity, were found to decrease cell viability by 50% (IC_50_) at concentrations between 10.9 to 122.5 μg/mL. One of these crude extracts produced by the halotolerant fungus *Nigrospora oryzae* was selected for further analysis, given the fact that this bioactivity was not displayed in previous studies and this species was recorded in association with a seaweed for the first time. Gas chromatography-mass spectrometry analysis of this extract allowed the identification of its major volatile metabolites, linoleic acid, palmitic acid and erucamide.

##### **Acknowledgments** 

This study had the support of Fundação para a Ciência e Tecnologia (FCT), through the strategic project UID/MAR/04292/2013 granted to MARE and the grant awarded to Maria da Luz Calado, Smart Valorization of Endogenous Marine Biological Resources Under a Changing Climate (reference Centro-01-0145-FEDER- 000018).

#### **Antioxidant Properties of Phlorotannin-Enriched Extracts from the Brown Algae *Fucus spiralis*** 

BarrosoSónia^1^HortaAndré^1^,^2^,^3^,^4^DuarteAna Marta^1^GilMaria Manuel^1^1MARE—Marine and Environmental Sciences Centre, ESTM, Polytechnic Institute of Leiria, 2520-630 Peniche, Portugal2Division of Aquaculture and Upgrading, Portuguese Institute for the Sea and Atmosphere, 1749-077 Lisboa, Portugal3Research Institute for Medicines, Faculty of Pharmacy, Universidade de Lisboa, 1649-003 Lisboa, Portugal4Pharmacological Sciences Department, Faculty of Pharmacy, Universidade de Lisboa, 1649-033 Lisboa, Portugal

**Scheme 3:**
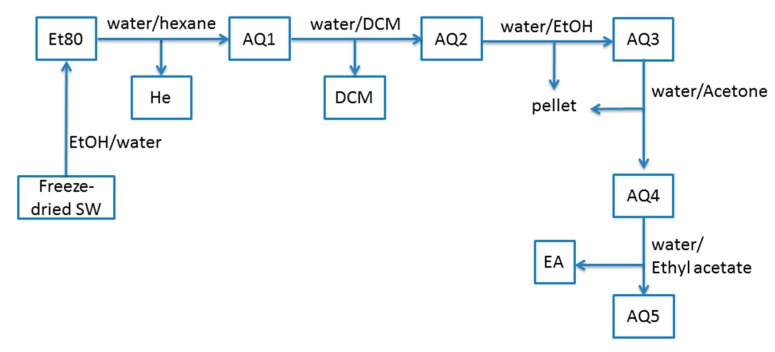
Extraction and fractionaction diagram.

The accumulation of harmful free radicals and ROS in the organism has been related to the occurrence of several diseases. Antioxidants have an important role in the prevention of such diseases. Algae, due to the constant exposition to extreme conditions, are rich in antioxidants along with several other nutritionally important compounds, being therefore a very promising source of bioactive functional ingredients. Brown algae, in particular, are rich in polyphenolic compounds, namely phlorotannins. In this study, crude ethanol-water (80:20) extracts (Et80) of the brown algae Fucus spiralis were fractionated using a liquid-liquid separation procedure based on polarity (Scheme 3) in order to obtain phlorotannin-enriched fractions. All the fractions were then evaluated for their antioxidant capacity (DPPH radical-scavenging and FRAP tests) and total phenolics (Folin-Ciocalteu method). Ethyl acetate fractions (EA) were the ones with higher content in phenols (389.24 ± 53.38 mg GAE/g extract), the best free radical-scavenging activity (IC_50_ = 9.44 μg/mL) and best results in the ferric-reducing antioxidant power (34.5424 ± 2.04 mg GAE/g extract). Preliminary characterization of the EA fractions by FTIR and HPLC revealed high content in phlorotannins.

##### **Acknowledgments** 

The authors thank FCT, Portugal, for financial support (UID/MAR/04292/2019). This work was also supported by projects SmartBioR (CENTRO-01-0145-FEDER-000018, Integrated Programme SR&TD co-funded by Centro 2020 program, Portugal 2020, European Union, through the European Regional Development Fund) and SeaWeedFeeds (16-02-01-FMP-84, Operational Programme MAR2020).

#### **Potential Use of Seaweed Extracts as Bioactive Molecules for Cyanobacterial Growth Regulation** 

ZerrifiSoukaina El Amrani^1^TazartZakaria^1^KhalloufiFatima El^1^,^2^OudraBrahim^1^CamposAlexandre^3^VasconcelosVitor^3^,^4^1Laboratory of Biology and Biotechnology of Microorganisms, Faculty of Sciences Semlalia Marrakech, Cadi Ayyad University, Av. Prince My Abdellah P.O. Box 2390, Marrakech 40000, Morocco2Polydisciplinary Faculty of Khouribga (FPK), Sultan Moulay Slimane University, Marrakech 40000, Morocco3CIIMAR, Interdisciplinary Centre of Marine and Environmental Research, Terminal de Cruzeiros do Porto de Leixões, Av. General Norton de Matos, s/n, 4450-208 Matosinhos, Portugal4Departament of Biology, Faculty of Sciences, University of Porto, Rua do Campo Alegre, 4169-007 Porto, Portugal

Cyanobacterial Harmful blooms became in last decades a serious problem for aquatic ecosystems as well for the human health. The most important freshwater bloom forming cyanobacteria are *Microcystis* spp. Due to their hazardous effects it is important to, control of those blooms. This work, evaluated the potential algicidal activities of fourteen species of seaweeds collected from the coast of Morocco, extracted with methanol, and screened in solid and liquid medium against the growth of the toxic cyanobacteria *Microcystis aeruginosa* and the microalgae *Chlorella* sp. The algicidal activity was tested for the first time by the agar diffusion technique in solid medium and the counting technique in liquid medium. The results in solid medium revealed that the algicidal activity was limited to *Microcystis aeruginosa*. The extract of *Bornetia secundiflora* showed the highest growth inhibition activity against *Microcystis*), whereas, the extracts of *Laminaria digitata, Halopytis incurvus, Ulva lactuca* and *Sargasum muticum* did not show any inhibition. In liquid medium, the results indicated that all methanolic extracts of different macroalgae tested had a significant inhibitory effect on *M. aeruginosa* compared to the positive control (copper sulphate). The maximum inhibition rates of *M. aeruginosa* was obtained by *Bifurcaria tuberculata*, *Codium elongatum* and *B. secundiflora* extracts. The potent inhibitory effects induced by some selected seaweed extracts could be explorer as a new ecological approach for cyanobacteria growth regulation in aquatic ecosystems.

#### **Antimicrobial and Antioxidant Activities of Sulphated Polysaccharides Extracted from Macroalgae Collected Off Peniche, Portugal** 

TeodoroFernando^1^MartinsAlice^1^AlvesCelso^1^SilvaJoana^1^PinteusSusete^1^AlvesJoana^1^FrancoMargarida^2^GasparHelena^3^PedrosaRui^1^1MARE—Marine and Environmental Sciences Centre, ESTM, Polytechnic Institute of Leiria, 2520-641 Peniche, Portugal2Centre for Rapid and Sustainable Product Development, Institute Polytechnic of Leiria, 2430-028 Marinha Grande, Portugal3University of Lisbon, Faculty of Science, BioISI—Biosystems and Integrative Sciences Institute, 1749-016 Lisbon, Portugal

Macroalgae are one of the most alluring sources of novel compounds, from which sulphated polysaccharides (SPs) have shown to possess promising bioactivities, that can be used in the development of state-of-the-art therapeutic approaches [1]. The aim of this research was to extract SPs from *Sargassum muticum*, *Bifurcaria bifurcata*, *Fucus spiralis*, *Asparagopsis armata*, *Plocamium cartilagineum*, *Sphaerococcus coronopifolius* and *Codium tomentosum*, collected from the Portuguese coast (Peniche), study their chemical features, and their antimicrobial and antioxidant activities.

SPs extraction was performed as described by Rodrigues [1] with slight modifications. SPs composition e.g., total carbohydrates, sulphate, and protein quantification were determined by standard methodologies. SPs extracts were also characterized through FTIR spectroscopy. Antioxidant capacity was evaluated by DPPH, FRAP, and ORAC assays. The antimicrobial activity was assessed against *Bacillus subtilis* (ATCC 6633), *Candida albicans* (ATCC 10231), *Escherichia coli* (ATCC 25922), *Pseudomonas aeruginosa* (ATCC 27853), *Salmonella enteritidis* (ATCC 13076) and *Staphylococcus aureus* (ATCC 25923), through the readings of optical density at 600 nm.

Although it was observed some variability in chemical composition, FTIR spectra evidenced characteristic bands of sulphated polysaccharides. Regarding the bioactivities, none of the crude SPs presented significant antioxidant properties. However, promising antimicrobial activities were evidenced by SPs from *S. muticum* (IC_50_ of 24.7, 28.0 and 9.3 μg/mL^−1^), and *S. coronopifolius* (IC_50_ of 42.0, 43.9 and 36.5 μg/mL^−1^) against *B. subtilis*, *P. aeruginosa*, and *S. aureus*, respectively, highlighting the potential of both seaweeds as a source of new antibiotics.

##### **Acknowledgments** 

This work was supported by the Portuguese Foundation for Science and Technology (FCT) through Strategic Projects UID/MAR/04292/2013 and UID/Multi/04046/2019 granted to MARE—Marine and Environmental Sciences Centre, and BioISI—BioSystems and Integrative Sciences Institute, respectively, through Red2Discovery Project (PTDC/MAR-BIO/6149/2014), co-financed by COMPETE (POCI-01-0145-FEDER-016791), through Oncologia de Precisão: Terapias e Tecnologias Inovadoras project (POINT4PAC) (SAICTPAC/0019/2015—LISBOA-01-0145-FEDER-016405) and through CrossAtlantic Project (PTDC/BIA-OUT/29250/2017), co-financed by COMPETE (POCI-01-0145-FEDER-029250).

References

1. Rodrigues, J.A.G.; Coura, C.O.; Benevides, N.M.B. Extração e caracterização físico-química dos polissacarídeos sulfatados da rodofícea Gracilaria cornea J. Agardh e avaliação de seus efeitos sobre coagulação in vitro/Extraction and physical-chemical characterization of sulfated polysaccharides from the rhdophyceae Gracilaria cornea J. Agardh and evaluation of their effects on coagulation in vitro. *Acta Fish. Aquat. Resour*. **2016**, *4*, 91–100.

#### **Bioactivity Screening of Marine Cyanobacteria for the Isolation of Novel Compounds for Hepatic Steatosis and Lipid Reducing Activity** 

AstrainBegoña^1^RosaFilipa^1^GestssonBaldvin^3^RibeiroTiago^1^SilvaNatália^1^VasconcelosVítor^1^,^2^EirikssonFinnur^3^ThorsteinsdottirMargrét^3^,^4^UrbatzkaRalph^1^1Interdisciplinary Center of Marine and Environmental Research (CIIMAR/CIMAR), University of Porto, Terminal de Cruzeiros de Leixões, Av. General Norton de Matos s/n, 4450-208 Matosinhos, Portugal2Faculty of Science, University of Porto, Rua do Campo Alegre 1021/1055, 4169-007 Porto, Portugal3ArcticMass, 101 Reykjavik, Iceland4Faculty of Pharmacy, University of Iceland, 107 Reykjavik, Iceland

Therapeutics for obesity and obesity-related co-morbidities, such as hepatic steatosis, have been widely studied due to the prevalence of metabolic diseases worldwide. Cyanobacteria, known as blue-green algae, are recognized to produce many secondary metabolites and may represent an interesting source of novel compounds. The aim of this study was to identify promising strains of cyanobacteria with activities towards obesity and steatosis. The reduction of neutral lipid accumulation was studied by the zebrafish Nile red fat metabolism assay. Steatosis was induced in HepG2 cells by fatty acid overloading and the reduction of lipid accumulation was analysed. A total of 138 extracts from 46 different cyanobacteria strains from LEGE culture collection were tested and promising bioactivities were obtained for each bioassay in some fractions. Toxicity evaluation and metabolite profiling by UPLC-MS-QTOF was further applied to narrow the selection of the most promising strains. The mass peaks obtained were analysed in different data bases and the comparison with our data revealed several hits of known compounds. Furthermore, the results also suggested the existence of several new compounds, highlighting the promising potential of these cyanobacteria strains. In the ongoing work, we intend to isolate novel bioactive compounds and elucidate their structures.

##### **Acknowledgments** 

This work was supported by the European ERA-NET Marine Biotechnology project CYANOBESITY (ERA-MBT/0001/2015), financed by national funds through FCT (Fundação para a Ciênca e a Tecnologia, Portugal).

#### **Antioxidant and Antiinflammatory Potential of the Marine Sponge *Cliona celata*** 

AlvesJoana^1^GasparHelena^2^SilvaJoana^1^AlvesCelso^1^PinteusSusete^1^TeodoroFernando^1^MartinsAlice^1^PedrosaRui^1^1MARE—Marine and Environmental Sciences Centre, ESTM, Polytechnic Institute of Leiria, 2520-641 Peniche, Portugal2BioISI—Biosystems and Integrative Sciences Institute, Faculty of Science, University of Lisbon, 1749-016 Lisbon, Portugal

Inflammation is a two edged-sword, as it can have both protective effects or collateral destructing consequences which combined with oxidative stress can lead to the development of deathly chronic inflammatory conditions. Marine sponges possess promising bioactive compounds with numerous therapeutic applications. The purpose of this research was to evaluate the antioxidant and antiinflammatory potential of crude extracts of *C. celata* collected at five different locations from the Portuguese coast. Crude extracts (methanol:dicloromethane 1:1) obtained from the five specimens of *C. celata* were analysed by HPLC-DAD, to evaluate their chemical profile. Selected extracts were fractionated by a preparative column chromatography, aiming identification of the fractions’ major compounds through GC-MS and NMR analysis. The antioxidant activity of the extracts was tested through DPPH, FRAP and ORAC assays. The cytotoxicity of extracts and lipopolysaccharides (LPS) was assessed in RAW 264.7 cells, using the MTT assay, while the antiinflammatory activity was determined using the nitric oxide (NO) assay, in which LPS were used as inducers of inflammation in the cellular model. At the maximum concentration (200 μg/mL) samples did not show a marked antioxidant activity. Additionally, in RAW 264.7 cells, extracts and LPS proved to be non-cytotoxic. Overall, the extracts reduced NO levels in LPS-induced RAW 264.7 cells, thus exhibiting antiinflammatory capacity against LPS (1.0 μg/mL), with one sample equalizing NO values to the ones of the control situation. *C. celata* is a known source of amino acid derived metabolites (alkaloids, clionamides and celenamides) and sterols (predominantly cholesterol and clionasterol). Fractions analysed evidenced the present of sterols, with cholesterol being identified as the major constituent.

##### **Acknowledgments** 

This work was supported by the Portuguese Foundation for Science and Technology (FCT) through Strategic Projects UID/MAR/04292/2013 and UID/Multi/04046/2019 granted to MARE—Marine and Environmental Sciences Centre, and BioISI—BioSystems and Integrative Sciences Institute, respectively, through Red2Discovery Project (PTDC/MAR-BIO/6149/2014), co-financed by COMPETE (POCI-01-0145-FEDER-016791), through Oncologia de Precisão: Terapias e Tecnologias Inovadoras project (POINT4PAC) (SAICTPAC/0019/2015—LISBOA-01-0145-FEDER-016405) and through CrossAtlantic Project (PTDC/BIA-OUT/29250/2017), co-financed by COMPETE (POCI-01-0145-FEDER-029250).

#### **Neuroprotective Activity of Organic Extracts from *Bifurcaria bifurcata*** 

SilvaJoana^1^,^2^AlvesCelso^1^GasparHelena^3^AlfonsoAmparo^2^PedrosaRui^1^1MARE—Marine and Environmental Sciences Centre, ESTM, Instituto Politécnico de Leiria, 2520-641 Peniche, Portugal2Department of Pharmacology, Faculty of Veterinary, University of Santiago de Compostela, 27002 Lugo, Spain3Faculty of Sciences, BioISI-Biosystems & Integrative Sciences Institute, University of Lisboa, Campo Grande, C8, 1749-016 Lisboa, Portugal

Pathogenesis of Parkinson’s disease (PD) remains unknown, however several studies suggest the involvement of distinct biological processes such as oxidative stress, mitochondrial dysfunction, and apoptosis leading to neuronal cell death. Despite the advances achieved in the last years, the current therapies for PD are very limited, being of utmost importance to develop new strategies, including the use of synthetic and natural antioxidants. The aim of this study was to evaluate the antioxidant and neuroprotective activities of ethyl acetate *Bifurcaría bifurcata* extract fractionated by column chromatography on an in vitro neuronal model of PD (SH-SY5Y). Ethyl acetate extract of *Bifurcaría bifurcata* was fractionated by column chromatography and eleven fractions (F1–F11) were obtained. The antioxidant activity of fractions were determined by DPPH radical scavenging activity, oxygen radical absorbance capacity (ORAC) and ferric reducing antioxidant power (FRAP). The neuroprotective effects of fractions in the presence of the neurotoxin 6-OHDA was followed by the MTT method. Moreover, Caspase-3 activity, reactive oxygen species (ROS), H_2_O_2_ production, mitochondrial membrane potential (MMP), and DNA fragmentation were also studied. Fractions F8–F11 exhibited significant antioxidant activities, being the fraction F8 the most potent. Regarding neuroprotective activities, fractions F8–F11 exhibited the highest potential against the neurotoxicity induced by 6-OHDA, prevent changes in mitochondrial membrane potential, reduced the production of ROS and H_2_O_2_, caspase-3 activity and DNA fragmentation, suggesting that these extracts may contain multi-target compounds acting on different pathways. Further studies will be performed in order to identify the bioactive compounds linked to the activities observed.

##### **Acknowledgments** 

This work was supported by the Portuguese Foundation for Science and Technology (FCT) through Strategic Projects UID/MAR/04292/2013 and UID/Multi/04046/2019 granted to MARE—Marine and Environmental Sciences Centre, and BioISI—BioSystems and Integrative Sciences Institute, respectively, through Red2Discovery Project (PTDC/MAR-BIO/6149/2014), co-financed by COMPETE (POCI-01-0145-FEDER-016791), through Oncologia de Precisão: Terapias e Tecnologias Inovadoras project (POINT4PAC) (SAICTPAC/0019/2015—LISBOA-01-0145-FEDER-016405) and through CrossAtlantic Project (PTDC/BIA-OUT/29250/2017), co-financed by COMPETE (POCI-01-0145-FEDER-029250).

#### **Antimicrobial and Antioxidative Activities of Marine Fungal Crude Extracts Isolated from the Portuguese Western Coast** 

CaladoMaria da Luz^1^AlvesCelso^1^SilvaJoana^1^PinteusSusete^1^PedrosaRui^2^GasparHelena^2^,^3^CamposMaria Jorge^2^1MARE—Marine and Environmental Sciences Centre, Instituto Politécnico de Leiria, 2520-641 Peniche, Portugal2MARE—Marine and Environmental Sciences Centre, ESTM, Instituto Politécnico de Leiria, 2520-641 Peniche, Portugal3BioISI—Biosystems and Integrative Sciences Institute, Faculdade de Ciências, Universidade de Lisboa, 1749-016 Lisboa, Portugal

Secondary metabolites produced by marine fungi have been recently recognized as potential alternative therapeutic agents, given their wide biological activities. In order to contribute to the bioprospection of novel and bioactive natural products, 20 crude ethyl acetate extracts produced by marine fungi *Sensu stricto* and halotolerant fungi isolated from Portuguese Western coast were tested for antimicrobial and antioxidative activities. The antimicrobial potential of the extracts was assessed by growth inhibition of *Staphylococcus aureus*, *Pseudomonas aeruginosa* and *Candida albicans*, and the antioxidative potential by 2,2-diphenyl-1-picrylhydrazyl radical scavenging (DPPH) and ferric reducing antioxidant power (FRAP) assays; the determination of total phenolics content (TPC) by Folin-Ciocalteu method was additionally performed. Extracts were tested at a maximum concentration of 200 μg/mL, and those that demonstrated a significant biological activity were tested at different concentrations to establish the IC_50_. The results revealed that none of these crude extracts inhibited significantly *P. aeruginosa* and *C. albicans* growth at 200 μg/mL; three of the extracts displayed a significant antibacterial activity against *S. aureus*, inhibiting 50% of bacterial growth at 39.18, 42.81 and 107.4 μg/mL. The results also showed that three of the crude extracts exhibited a high DPPH radical scavenging activity, with IC_50_ values of 9.33, 25.30 and 51.09 μg/mL; the same extracts presented the highest phenolics content. Only one of these extracts demonstrated high antioxidant activity by FRAP assay. Even though these preliminary results highlighted the potential of fungal crude extracts, further chromatographic/spectroscopic approaches should be performed in order to identify the bioactive compounds present.

##### **Acknowledgments** 

This study had the support of Fundação para a Ciência e Tecnologia (FCT), through the strategic project UID/MAR/04292/2013 granted to MARE and the grant awarded to Maria da Luz Calado, Smart Valorization of Endogenous Marine Biological Resources Under a Changing Climate (reference Centro-01-0145-FEDER- 000018).

#### **Deep Sea Actinomycetes from Estremadura Spur, Continental Portugal, Targeted as Antimicrobial Agents** 

LuppinoLuca^1^GriloInês R.^1^FonsecaGonçalo^1^RodriguesClara F.^2^SobralRita G.^1^GaudêncioSusana P.^1^1UCIBIO—Applied Molecular Biosciences Unit, Faculdade de Ciências e Tecnologia da Universidade NOVA de Lisboa, 2829-516 Caparica, Portugal2CESAM, Departamento de Biologia, Universidade de Aveiro, 3810-193 Aveiro, Portugal

Bacteria are an exceptional source of chemical diversity. Among them, species of the Order *Actinomycetales* (commonly named actinomycetes) are the single most productive source of microbial derived natural products, accounting for ca. 75% of all antibiotics discovered [1], as well as a broad range of anticancer agents. Although the actinomycetes are best known as soil bacteria, a growing interest in its distribution and ecological role in the marine environment has been observed. The oceans are a highly complex microbiological environment with typical microbial abundances of 10^6^ and 10^9^ per mL in seawater and ocean-bottom sediments respectively [2]. During the last decade, our group has been investigating the actinomycete biodiversity isolated from ocean sediments collected off the Portuguese Archipelagos (Madeira and Azores) and off the coast of Portugal mainland, from 10 m to 1300 m depth, which allowed to discover a high number of new actinomycete phylotypes from known MAR groups [3,4]. This work represents the first report of targeted identification of deep sea actinomycetes collected off the Portuguese Continental coast. Herein, 83 actinomycete strains were obtained from Estremadura Spur deep sea sediments (200–400 m depth) and screened for their ability to inhibit multi-drug resistant pathogenic bacteria MRSA, MSSA and *E. coli*, using techniques implemented in our lab [4,5]. Results showed the biotechnological potential of Estremadura Spur actinomycete isolates as a source of antimicrobial agents.

##### **Acknowledgments** 

This work was supported by the Applied Molecular Biosciences Unit—UCIBIO which is financed by national funds from FCT/MCTES (UID/Multi/04378/2019). FCT/MCTES through grants PTDC/QUIQUI/119116/2010, PTDC/GEO-FIQ/5162/2014, PTDC/BIA-MIC/31645/2017 and IF/00700/2014.

References

1. Bérdy, J. Bioactive microbial metabolites. *J. Antibiot.*
**2005**, *58*, 1.
2. Fenical, W.; Jensen, P.R. Developing a new resource for drug discovery: marine actinomycete bacteria. *Nat. Chem. Biol.*
**2006**, *2*, 666–673.
3. Jensen, P.R.; Mafnas, C. Biogeography of the marine actinomycete Salinispora. *Environ. Microbiol.*
**2006**, *8*, 1881–1888.
4. Prieto-Davo, A.; Dias, T.; Gomes, S.E.; Rodrigues, S.; Parera-Valadez, Y.; Borralho, P.M.; Pereira, F.; Rodrigues, C.M.P.; Santos-Sanches, I.; Gaudêncio, S.P. The Madeira Archipelago As a Significant Source of Marine-Derived Actinomycete Diversity with Anticancer and Antimicrobial Potential. *Front. Microbiol.*
**2016**, *7*, 1594, doi:10.3389/fmicb.2016.01594.
5. Bauermeister, A.; Velasco-Alzate, K.; Dias, T.; Macedo, H.; Ferreira, E.G.; Jimenez, P.C.; Lotufo; T.M.C.; Lopes, N.P.; Gaudêncio, S.P.; Costa-Lotufo, L.V. Metabolomic Fingerprinting of Salinispora From Atlantic Oceanic Islands. *Front. Microbiol.*
**2019**, *9*, 3021, doi:10.3389/fmicb.2018.03021.

#### **Marine Bacteria as a Potential Source for Discovering Novel Antimicrobial Bioactive Compounds** 

SegopaEllen K.^1^ZylLeonardo Van^1^BurgerAnita^1^TrindadeMaria^1^HalleenFrancois^2^1Institute for Microbial Biotechnology and Metagenomics (IMBM), University of the Western Cape, Bellville 7530, South Africa2Division of Plant Protection Nietvoorbiej, Agricultural Research Council, Stellenbosch 7600, South Africa

Due to the rising issue of antimicrobial resistance development to current antimicrobial drugs, there is a constant need for novel natural products, preferably with different modes of action to existing ones. Therefore research interest has turned to the use of secondary metabolites from microorganisms specifically from the marine environment, which is known to harbour microorganisms with structurally diverse and bioactive metabolites. The aim of this study was to further investigate the antimicrobial activity observed in a previous study at the IMBM. The marine bacterium; PE6-126 was isolated from the marine sponge, *Distaplia skoogi* collected in Algoa Bay (Port Elizabeth, South Africa). This organism was selected based on the antimicrobial activity and was identified by genome sequencing data as a *Bacillus cereus*. AntiSMASH analysis of the genome predicted eight secondary metabolite clusters, one of which showed 100% sequence identity to the Thuricin CD pathway in *Bacillus thuringiensis*. Considering that Thuricin CD has been shown to have antimicrobial activity against *B. cereus* (ATCC1072), it might be responsible for the observed anti-microbial activity of PE6-126 extract against *B. cereus*.

To date, the identity and properties of the compound conferring the antimicrobial activity remains unknown. Preliminary characterization of the extract showed that the active compound is heat stable and not susceptible to proteolytic digestion by Proteinase K, indicating that it might not be a peptide. Isolation of the compound is being pursued by employing a bioassay guided isolation technique using High Performance Liquid Chromatography (HPLC).

#### **Isolation and Bioactivity Screening of Deep-Sea Actinobacteria from the Madeira Archipelago** 

RibeiroInês^1^,^2^GirãoMariana^1^RegoAdriana^1^,^2^RibeiroCíntia^1^,^3^BrancoFrancisca^1^,^3^AlmeidaEduarda^1^,^3^Braga-HenriquesAndreia^4^,^5^MuchaAna P.^1^UrbatzkaRalph^1^LeãoPedro^1^CarvalhoMaria F.^1^1CIIMAR—Interdisciplinary Centre of Marine and Environmental Research, University of Porto, Terminal de Cruzeiros do Porto de Leixões, Avenida General Norton de Matos, S/N, 4450-208 Matosinhos, Portugal2Institute of Biomedical Sciences Abel Salazar, University of Porto, Rua de Jorge Viterbo Ferreira 228, 4050-313 Porto, Portugal3Faculty of Sciences, University of Porto, Rua do Campo Alegre, s/n, 4169-007 Porto, Portugal4MARE-Marine and Environmental Sciences Centre, Estação de Biologia Marinha do Funchal, Cais do Carvão, 9900-783 Funchal, Madeira Island, Portugal5ARDITI-Regional Agency for the Development of Research, Technology and Innovation, Oceanic Observatory of Madeira (OOM), Madeira Tecnopolo, Caminho da Penteada, 9020-105 Funchal, Portugal

Deep-sea environments present unique characteristics in terms of temperature, light, pressure, salinity, oxygen concentration and nutrient availability, forcing the adaptation of microbial life with the consequent development of differentiated metabolisms and physiologies. Actinobacteria are well known for their great ability to synthesize secondary metabolites with diverse biotechnological properties. Bioprospection of actinobacteria in underexplored environments, such as deep sea, may be a key to the discovery of new bioactive molecules. In this study, 24 deep-sea samples collected at the Madeira archipelago at depths between 400 and 3200 m were bioprospected for actinobacteria capable of producing metabolites with antimicrobial and anticancer activities. Two pre-treatments and three selective culture media supplemented with different antibiotics were used to promote the isolation of actinobacteria. Up to the moment, nine actinobacteria strains were isolated from the processed samples, and 16S rRNA gene sequencing analysis showed that these strains are phylogenetically related with the genera *Micrococcus*, *Streptomyces*, *Brevibacterium* and *Tsukamurella*. Antimicrobial screening of crude extracts obtained from liquid cultures of nine actinobacteria isolates revealed one extract of a *Streptomyces* strain capable of inhibiting the growth of *Staphylococcus aureus* and two other extracts of strains of the same genus capable of inhibiting the growth of *Candida albicans*. Screening of anticancer activity for these same extracts showed that two Streptomyces strains were able to reduce the viability of the human cancer cell line HepG2 in ca. 40%. The next steps of this work will focus on the isolation and identification of other strains and screening of their bioactive potential.

#### **Deep-Sea Actinobacteria from Azores: Diversity and Investigation of their Bioactive Potential** 

GirãoMariana^1^RibeiroInês^1^,^2^RegoAdriana^1^AlmeidaEduarda^1^,^3^RibeiroCíntia^1^,^3^BrancoFrancisca^1^,^3^Braga-HenriquesAndreia^4^,^5^MuchaAna P.^1^CarvalhoMaria F.^1^1CIIMAR-Interdisciplinary Centre of Marine and Environmental Research, University of Porto, Matosinhos, 4450-208 Porto, Portugal2ICBAS-Institute of Biomedical Sciences Abel Salazar, University of Porto, 4050-313 Porto, Portugal3FCUP-Faculty of Sciences of the University of Porto, 4169-007 Porto, Portugal4MARE-Marine and Environmental Sciences Centre, Estação de Biologia Marinha do Funchal, Cais do Carvão, 9900-783 Funchal, Madeira Island, Portugal5ARDITI-Regional Agency for the Development of Research, Technology and Innovation, Oceanic Observatory of Madeira (OOM), Madeira Tecnopolo, Caminho da Penteada, 9020-105 Funchal, Madeira Island, Portugal

Deep-sea environments are characterized by extreme and unique conditions that can drive their microbial dwellers to produce novel natural products. These molecules have been an increasing target of bioprospection in the last years as they can provide biotechnological and pharmaceutical solutions for several societal problems, in particular for human health. Within the scope of this work, 34 samples (including sediments and organisms from Porifera and Cnidaria phyla) were collected at the archipelago of Azores, Portugal, at depths between 150–2900 m, with the goal of isolating Actinobacteria and evaluating their ability to produce compounds with antimicrobial and anticancer activities. A total of 110 microbial isolates have been obtained. The major Actinobacteria strains belonged to the *Microbacterium* and *Rhodococcus* genera. Extracts from these cultures were tested against reference Gram-positive and Gram-negative bacteria and yeasts, as well as for their cytotoxicity towards a panel of cancer cell lines. We discuss the potential of deep-sea Azorean environments for bioprospection.

#### **Project I. Film: Optimization of Seaweed Bioactive Extracts and Its Use as an Additive in Nanofiber Food Coatings** 

ReboleiraJoão^1^AndradeMariana^2^Sanches-SilvaAna^3^,^4^SousaDora^5^MateusArtur^5^GanhãoRui^1^MendesSusana^1^VilarinhoFernanda^2^BernardinoSusana^1^1MARE—Marine and Environmental Sciences Centre, ESTM, Instituto Politécnico de Leiria, 2520-641 Peniche, Portugal2Department of Food and Nutrition, National Institute of Health Dr Ricardo Jorge (INSA), Avenida Padre Cruz, 1649-016 Lisbon, Portugal3Center for Study in Animal Science (CECA), ICETA, University of Oporto, 4051-401 Oporto, Portugal4National Institute for Agricultural and Veterinary Research (INIAV), Vairão, 4485-655 Vila do Conde, Portugal5Centre for Rapid and Sustainable Product Development, Polytechnic Institute of Leiria, Zona Industrial, Rua de Portugal, 2430-028 Marinha Grande, Portugal

Modern poultry packaging techniques, such as modified atmosphere packaging and vacuum packaging, are effective at reducing the impact of microbial spoilage over protein-rich foodstuffs. While a relatively high shelf-life is achieved through these methods, the suppression of microbial spoilage means lipid oxidation takes the limelight as the limiting factor in the storage of poultry and poultry products. Small gains in shelf-life for poultry products remain very desirable, and potentially profitable. The incorporation of antioxidant and antimicrobial agents in packaging films has demonstrated reliable increases in product stability. However, the synthetic source of these agents and/or their extraction procedures often make them undesirable from an environmental and economic perspective. The Portuguese coast presents itself as a source of macroalgae, a readily available and underdeveloped source of natural bioactive material, and their incorporation in food packaging can still present itself as a novel and effective method of solving problems in the food industry. Project I. Film attempts to fulfil this vision through the development of thermoplastic food coatings enriched with marine bioactives from readily available aquaculture macroalgae. Hydroethanolic extracts of red macroalgae *Gracilaria gracilis* and *Porphyra dioica* were evaluated for their antioxidant potential (DPPH and FRAP assays) and phenolic content, and extraction conditions were set for maximum yield of these parameters. These extracts were processed through electrospinning into a nanofiber coating, using high molecular weight PEO (polyethylene oxide) as the base polymer. Bioactive stability of the electrospun material was then evaluated through the same antioxidant assays.

##### **Acknowledgments** 

This work was supported by the research project “i.FILM—Multifunctional Films for Intelligent and Active Applications” (ref. 17921) cofounded by European Regional Development Fund (FEDER) through the Competitivenessand Internationalization Operational Program under the “Portugal 2020” Program, Call no. 33/SI/2015, Co-Promotion Projects and by the Integrated Programme of SR&TD “Smart Valorization of Endogenous Marine Biological Resources Under a Changing Climate” (reference Centro-01-0145-FEDER-000018), co-funded by Centro 2020 program, Portugal 2020, European Union, through the European Regional Development Fund. João Reboleira and Mariana Andrade are grateful for their research grant (2016/iFILM/BM) in the frame of iFILM project. Also, this study had the support of Fundação para a Ciência e Tecnologia (FCT), through the strategic project UID/MAR/04292/2019 granted to MARE.

#### **Bioactivity Screening of Cyanobacteria for Repression of Intestinal Lipid Absorption** 

da CostaSusana Lemos^1^,^2^,^3^RibeiroTiago^1^RosaFilipa^1^VasconcelosVítor^1^,^2^UrbatzkaRalph^1^1Interdisciplinary Centre of Marine and Environmental Research (CIIMAR/CIMAR), University of Porto, 4450-208 Matosinhos, Portugal2Faculty of Sciences, University of Porto,4169-007 Porto, Portugal3Abel Salazar Biomedical Sciences Institute, University of Porto, 4050-313 Porto, Portugal

Obesity is a worldwide epidemic that affects over 600 million people, regarded as a critical health risk to develop associated diseases such as diabetes, cardiovascular or even several types of cancer. Cyanobacteria are known for a high production of secondary metabolites that may reveal bioactivities for the treatment of obesity. In this work, we aimed to study cyanobacteria for their beneficial effects on obesity, by repressing intestinal lipid absorption using zebrafish larvae as a whole small animal model. Freshwater, estuarine and marine cyanobacterial strains were obtained from CIIMAR’s Blue Biotechnology and Ecotoxicology Culture Collection (LEGE-CC). After exposure to the cyanobacterial fractions, the ability to inhibit intestinal lipase and protease activity in the context of a whole organism was assessed. The activity was visualized in zebrafish larvae utilising a fluorescence-based method with PED6 and EnzChek, as phospholipase and protease reporters, respectively. The use of both reporters creates a more physiologically relevant readout of the complexity of digestive processes. The screening of cyanobacterial fractions allowed the identification of a few promising strains with over 60% inhibition of lipase activity. Those fractions with bioactivity and absence of general toxicity or malformations were selected for future works. Here, we intend to isolate the responsible compounds repressing intestinal lipid absorption.

##### **Acknowledgments** 

This work was supported by the European ERA-NET Marine Biotechnology project CYANOBESITY (ERA-MBT/0001/2015), financed by national funds through FCT (Foundation for Science and Technology, Portugal).

#### **TASCMAR EU Project. Marine Invertebrates and Associated Microorganisms, a Global Science for a Global Valorization** 

OuazzaniJamal^1^TASCMAR PartnersCollaborators^2^,^3^,^4^,^5^,^6^,^7^,^8^,^9^,^10^,^11^,^12^,^13^1Institut de Chimie des Substances Naturelles ICSN, CNRS, 91190 Gif sur Yvette, France2University of Athens, 15771 Athens, Greece3Tel Aviv University, 69978 Tel Aviv, Israel4Chulalongkorn University, 10330 Bangkok, Thailand5Université de la Réunion, 97715 Saint Denis, France6Crelux GmbH, 82152 Martinsried, Germany7BICT, 26900 Lodi, Italy8Pierre Guerin Technologies, 79210 Mauze, France9iMARE Natural S.L., 18600 Motril, Spain10Astareal, A.B.; 13440, Gustavsberg, Sweden11Apivita, 19003 Athens, Greece12T6 Ecosystems, 00184 Roma, Italy13Ecoocean, 37804 Kibbutz Sdo Yam, Israel

Mesophotic Coral Ecosystems (MCEs), extending from 30 to 150 m depth, are almost entirely unexplored, they are a treasure-trove for discovering new species and their associated bioactive chemicals. Organisms such as soft corals, sponges, and microbes living on coral reefs naturally produce potent cocktails of chemicals to defend themselves from competitors and harmful predators, or to contribute to multi-species ecosystem cohesion and development (the holobiont concept). EU H-2020 project TASCMAR aims to tackle major bottlenecks in the discovery, development, and commercialization of marine-derived chemicals with a specific focus on using new biological and chemical resources from MCEs (www.tascmar.eu). The project partners develop innovative technologies for the sustainable cultivation of marine resources, and isolation of chemicals in their natural environment without the need to harvest them. The project is specifically looking for new chemical compounds active against age-related illnesses such as Alzheimer’s, Parkinson’s, cancer, and aging diseases related to muscles and skin. TASCMAR is collecting samples of marine invertebrates from biodiversity hotspots around the world. Special emphasis is given to sustainable bioprospecting, from collection to cultivation, and developing technologies for intensification of the active bio-resource production. This also includes microbial symbionts (actinomycetes and fungi). Modern chemical approaches involving up-to-date extraction and structural elucidation technologies were engaged. Extracts, fractions and pure compounds were then screened on a panel of molecular and cell based bioassays related to anti-ageing. An overview of this unique international collaborative adventure will be presented.

#### **Effect of Time and Temperature on the Recovery of Fucoidans and Phlorotannins from *Fucus vesiculosus*** 

FerreiraRicardo M.^1^RamalhoAna^2^CostaRui^2^PereiraRui^3^SilvaArtur M. S.^1^CardosoSusana M.^1^1QOPNA & LAQV-REQUIMTE, Department of Chemistry, University of Aveiro, 3810-193 Aveiro, Portugal2Polytechnic Institute of Coimbra, ESAC, CERNAS, 3045-601 Coimbra, Portugal3ALGAplus, Produção e Comercialização de Algas e seus Derivados Lda., 830-196 Ílhavo, Portugal

Algae are rich sources of key nutrients and/or bioactive compounds, claimed to promote health and reduce disease risk. Among them, brown macroalgae are recognized for their richness in dietary fibers (fucoidans, laminarans and/or alginates), phlorotannins and fucoxanthin (**1**,**2**) [1]. This study aimed to evaluate the impact of different extraction temperatures (25 °C, 50 °C, 75 °C, 100 °C and 120 °C) and times (5′, 1 h, 2 h and 4 h) on the recovery of fucoidans and phlorotannins from the macroalgae *Fucus vesiculosus*, as well on the antioxidant ability of the resulting extracts. The amount of fucoidans and phlorotannins were estimated through the quantification of the amount of fucose and the 2,4-dimethoxybenzaldehyde assay, respectively. In addition, the antioxidant potential was evaluated by the scavenging capacity of the radical 2,2’-azino-bis(3-ethylbenzothiazoline-6-sulphonic acid) (ABTS).

The range of mass yields varied between 28–69%, while levels of recovered fucoidans and phlorotannins were between 2.80–47.26 gfuc/galgae and 0.115–0.256 mgEFG/g_algae_, respectively. These results indicated that the recovery of these bioactive compounds, particularly of fucoidans, were strongly influenced by the increment in the extraction temperature and time. Regardless that, the extracts only showed slight differences regarding their antioxidant capacity, as assessed by the ability to scavenge the ABTS radical (IC_50_ values of 0.09–0.13 mg/mL), a fact that is mostly associated with variations in the levels of phlorotannin (0.32–0.56 mgEFG/g_extract_).

##### **Acknowledgments** 

This work is co-financed by the European Regional Development Fund (ERDF), through the partnership agreement Portugal2020—Regional Operation Program CENTRO2020, under the project CENTRO-01-0145-FEDER-023780 HEPA: Healthier eating of pasta with algae. Thanks are due to the University of Aveiro and FCT/MCT for the financial support for the QOPNA research Unit (FCT UID/QUI/00062/2019) through national founds and, where applicable, co-financed by the FEDER, within the PT2020 Partnership Agreement, and to the Portuguese NMR Network. Susana Cardoso thanks the research contract under the project AgroForWealth (CENTRO-01-0145-FEDER-000001), funded by Centro2020, through FEDER and PT2020.

References

1. Afonso, N.C.; Catarino, M.D.; Silva, A.M.S.; Cardoso, S.M. Brown macroalgae as valuable food ingredients. *Antioxidants*
**2019**, *8*, 365, doi:10.3390/antiox8090365.

#### **A Sustainable Bioprocess Based on the Marine Bacterium *Termotoga neapolitana* for the Production of Energy and Functional Products** 

d’IppolitoGiulianaLandiSimoneEsercizioNunziaVastanoMarcoCasoLucioNuzzoGenoveffaFontanaAngeloIstituto di Chimica Biomolecolare ICB-CNR, Via Campi Flegrei 34, 80078 Pozzuoli (Napoli), Italy

*Thermotoga neapolitana* is a marine anaerobic bacterium with an optimal growth temperature of 80 °C, suitable for conversion of sugars into hydrogen with yields close to theoretical values. The fermentation process has shown general robustness, reproducibility and consistency and a low risk of contamination because of the hyperthermophilic conditions. The bacterium also operates an uncommon process named Capnophilic (CO_2_-requiring) Lactic Fermentation (CLF) to convert CO_2_ and acetate to 95% e.e. l-lactic acid. Within the frame of the European Project BioRECO2VER (Horizon 2020 Research and Innovation Programme under Grant Agreement, No. 760431), we are working to make this patented process the cornerstone for biotechnological applications based on CO_2_ valorization. In this contribution, we present this activity and the latest results related to the biochemical cross-talking and metabolic engineering of the pathways related to hydrogen and lactic acid production, as well as optimization of upstream and downstream processes including improvement of final product titer, definition of operational parameters, reactor design and the separation of the fermentation products. BioRECO2VER Project started in January 2018 and include two Research and Technology Organizations, 2 Universities, four SMEs and four large industries.

#### **Optimising Microalgae Metabolomics Using Light** 

HughesAlison H.^1^McKenzieDouglas^2^DuncanKatherine R.^1^1Strathclyde Institute of Pharmacy and Biomedical Sciences, Glasgow G4 0RE, UK2Xanthella Ltd., European Marine Science Park, Oban, Argyll PA37 1SZ, UK

Microalgae remain an underexplored source of high value metabolites, such as pharmaceuticals. In a laboratory setting, microalgal cultures often contain associated bacteria, whose growth must be kept at a minimum due to the comparatively slow growth of microalgae. This is difficult and expensive to achieve, especially at an industrial scale using photobioreactors. The use of narrowband LED wavelengths, particularly 405 nm, supresses bacterial growth without affecting microalgal growth. This has a knock-on effect on the specialised metabolite profile of the microalgal cultures when compared to growth under white light.

This study compares the effect of 405 nm and white light on the growth, gross primary productivity (GPP), and specialised metabolite production of four phylogenetically diverse strains of microalgae (*Nannochloropsis oculata*, *Tetraselmis tetrabrachia*, *Phaeodactylum tricornutum*, and *Rhodella maculata*). Screening of metabolite extracts against a panel of 10 human and fish pathogens (bacterial and fungal) was used to prioritise samples for LC-MS analysis. Comparative metabolomics coupled with dereplication tools were used to identify specialised metabolites produced explicitly under 405 nm light.

Although having similar growth rates, GPP shows differences in the utilisation of 405 nm vs. white light. The differences in bioactivity profiles is illustrated in OPLS-DA plots, which were also used to show contrast between species. The increase in lipid and carotenoid production are most likely responsible for the anti-microbial activity observed in all species. Production of high-value metabolites in addition to the suppression of associated bacteria and reduction of energy costs, could greatly improve the industrial exploitation of microalgae for healthcare and pharmaceuticals.

#### **New Colorless Scytonemins with Strong UV Absorption and Antimicrobial Activity** 

MartinsTeresa^1^ReisMariana^1^HassouaniMeryem^2^SabourBrahim^2^VasconcelosVitor^1^,^3^LeãoPedro^1^1Interdisciplinary Centre of Marine and Environmental Research (CIIMAR/CIMAR), University of Porto, Terminal de Cruzeiros do Porto de Leixões, 4450-208 Matosinhos, Portugal2Faculty of Sciences, Chouaib Doukkali University, Route Ben Maachou, B.P.:20, 24000 El Jadida, Morocco3Faculty of Sciences, University of Porto, Rua do Campo Alegre, 4169-007 Porto, Portugal

Ultraviolet (UV) radiation is the most harmful and mutagenic component of the solar spectrum, being the main cause of some forms of skin cancer. Negative effects of UV-B radiation, especially carcinogenicity, have been well known for several years but recent studies have also shown that UV-A leads to eye damage, loss of skin elasticity, wrinkles and consequently, premature ageing, while also stimulating skin cancer development. Currently used sunscreens employ UV-filtering agents that have been associated with environmental persistence and toxicity. Hence, as authorities re-evaluate the benefits of currently available sunscreen ingredients, there is a pressing need to discover new and effective sunscreens that do not pose a risk to the environment.

In Nature, microorganisms, and particularly cyanobacteria, have developed several protection strategies against the UV radiation, including the production of specialized UV-protective compounds such as scytonemin. In this work, we describe the isolation and structural elucidation of a series of new scytonemin analogs, from a Moroccan cyanobacterial mat. The compounds present diverse absorption properties, and used synergistically they can strongly absorb radiation throughout the UV-B and UV-A ranges. Unlike previous scytonemins that presented dark vivid colors, precluding a number of cosmetic applications, the new molecules present faint colors or are even colorless. Furthermore, these new scytonemins present interesting antimicrobial properties which were never reported for any molecule of this family. Considering their natural origin, bioactivity, lack of color and broad UV absorption range, these new scytonemins are good candidates for development into multifunctional ingredients for topical use.

#### **Universal Fermenter for Cultivation of Microorganisms from the Mesophotic Zone** 

FelezeuDoru^1^ChahdiJamal Ouazzani^2^Le GoffGéraldine^2^Allegret-BourdonChristophe^1^TouronArnaud^1^1Pierre Guerin Technologies, 79000 Niort, France2Cnrs, Institut De Chimie des Substances Naturelles Icsn, 91198 Gif-Sur-Yvette, France

Microbial fermentation technology has recently introduced new techniques to facilitate the implementation of experiments for the production of valuable microbial metabolites. However, the whole shape, accessories and processes have remained the same for centuries. Few operators act in the field of fermentation technology and the market is shared among them with equivalent offers.

With the experience of Platotex developed by CNRS and built by Pierre Guerin and an international benchmark and survey including various operators and end-users, CNRS and Pierre Guerin developed a clear idea on how to move beyond the state of the art by offering a groundbreaking new version of the Platotex, covering every application in the microbial fermentation field. This is what we call the “UNIVERSAL FERMENTER”, also called “UNIFERTEX”.

With this innovative approach, new technology and an available operational device for demonstration, H2020 TASCMAR Project has achieved one of its key objectives. Thanks to a lasting interaction and complementarity between the two involved partners, the successful building of an operational unique device was ashieved. The black box approach to the development of new technology consists of listing the ideal requirements for the equipment: liquid state cultivation & solid state cultivation, coupling extraction steps, up-scale & down-scale shape, liquid & solid sampling technology, liquid & viscous inoculation accessories, being compact from laboratory to industrial version, easy handling, remote control, international safety requirements. Although challenging it is the aim to integrate all of the requirements in one device. The prototype consists in a 500 L WV vessel with innovative tools and accessories, able to support micro-organisms cultivation under sterile conditions. We established the main characteristics of the prototype such as working volume capacity, shape of the vessel, raw materials, rough dimensions, accessories foreseen to be implemented within the equipment, instrumentation for measuring and controlling process parameters such as temperature, pressure, etc. Pierre Guerin project team has ‘translated’ the User Requirement Specifications into technical specifications in accordance to industrial regulations: design according to construction codes: CODAP, PED; compliance to international regulations and standards: ASME BPE, GAMP, 97/23/EC, ISA; using advanced tools including: INVENTOR CAD system for full 3D design and ANSYS MECHANICAL for mechanical constraints calculations.

#### **Effects of Co-Culturing and Stressor Stimulus on Functional Metabolites Production in Marine Microorganisms** 

OngJi Fa MarshallTanLik TongNatural Sciences and Science Education, National Institute of Education, Nanyang Technological University, 1 Nanyang Walk, Singapore 637616, Singapore

Marine microorganisms are prolific sources of novel bioactive metabolites that are important in today’s drug discovery and development programme. However, under routine laboratory culturing conditions, many biosynthetic genes of these microbes remain silent and are not expressed in vitro, hence limiting the chemical diversity of the metabolites that can be detected. The main reason for this phenomenon is that microorganisms grown in pure culture under routine conditions, lack the activating signals that are often required to trigger/activate the biosynthetic gene clusters. Strategies involving external physical stressor stimulus, such as elevated temperature, and/or co-culturing of different microbial strains, tries to mimic the complex microbial communities where these microorganisms co-exist. In this preliminary study, a series of antagonistic experiments involving co-cultivation of marine bacterial strains as well as subjecting each bacterial strain to elevated temperature have resulted in the production of new metabolites. The detection of new metabolites were based on a combination of ^1^H-NMR metabolomics approach as well as LC-MS system. This research highlights the potential use of co-cultivation and the effects of stressor stimulus on enhancing the chemical diversity of metabolites produced by marine microorganisms.

##### **Acknowledgments** 

This research is supported by the National Research Foundation, Prime Minister’s Office, Singapore under its Marine Science Research and Development programme (Award Nos. MSRDP-P15 and MSRDP-P34).

#### **Alginate Biogels Enriched with Kefir Whey and *Gracilaria gracilis* Extracts: Exploring the Pharmaceutical Potential of Combined Marine and Dairy Biotechnology** 

ReboleiraJoão^1^SaturnoCamila^1^BragançaAna Rita^1^AdãoPedro^1^AfonsoClélia^1^BernardinoRaul^1^,^2^BernardinoSusana^1^1MARE—Marine and Environmental Sciences Centre, ESTM, Instituto Politécnico de Leiria, 2520-641 Peniche, Portugal2LSRE-LCM/IPLeiria Laboratory of Separation and Reaction Engineering-Laboratory of Catalysis and Materials, Polytechnic Institute of Leiria, 2411-901 Leiria, Portugal

Despite modern trends leading to a phasing out of synthetic chemicals in the pharmaceutical and cosmetics industries, these are still abundantly used as both active agents and additives in many consumer-grade products and formulations. There is an increasing awareness of the multi-layered benefits associated with replacing synthetic compounds with natural bioactives that keeps fuelling the search for new natural sources. These benefits include reduced long-term health issues, monetization of local natural resources, implementation of novel and sustainable extraction techniques, and alignment with modern consumer preferences. Novel sources of natural bioactives were tested for their synergetic potential as constituents of a biogel for cutaneous treatment of wounds. These included kefir whey and aqueous extracts of the red macroalgae *Gracilaria gracilis* which were selected due to reported antimicrobial and antioxidant activities, determined in the initial stages of project “i.FILM—Multifunctional Films for Intelligent and Active Applications”. Kefir growth parameters and conditions were optimized in order to maximize the inhibitory effects on several pathogenic strains, including Escherichia coli, Staphylococcus aureus and Pseudomonas aeruginosa. A similar approach was taken for the extraction of *G. gracilis* biomass, maximizing antioxidant activity on DPPH and FRAP assays. Biogel formulations, including thickening and calcium scavenging agent concentrations were selected based on viscosity, skin feel and visual evaluation. Biogel effectiveness was estimated through growth kinetics assays and the rheological and colour stability was measured over the course of four weeks, as to estimate shelf-life.

##### **Acknowledgments** 

This work was supported by the research project “i.FILM—Multifunctional Films for Intelligent and Active Applications” (ref. 17921) cofounded by European Regional Development Fund (FEDER) through the Competitiveness and Internationalization Operational Program under the “Portugal 2020” Program, Call no. 33/SI/2015, Co-Promotion Projects and by the Integrated Programme of SR&TD “Smart Valorization of Endogenous Marine Biological Resources Under a Changing Climate” (reference Centro-01-0145-FEDER-000018), co-funded by Centro 2020 program, Portugal 2020, European Union, through the European Regional Development Fund. João Reboleira is grateful for his research grant (2016/iFILM/BM) in the frame of iFILM project. Also, this study had the support of Fundação para a Ciência e Tecnologia (FCT), through the strategic project UID/MAR/04292/2019 granted to MARE.

#### **Extraction Optimization of Phycobiliprotein Pigments from *Gracilaria gracilis* for Natural Food Colorants** 

PereiraTatianaBarrosoSóniaMendesSusanaBaptistaTeresaGilMaria M.MARE—Marine and Environmental Sciences Centre, ESTM, Polytechnique Institute of Leiria, 2520-641 Peniche, Portugal

Colorants have been used in the food industry for centuries, but nowadays with the ever-changing consumption trends, there is a high demand for natural alternatives capable of replacing the widely used synthetic colorants. Phycobiliproteins are water-soluble proteins mainly present in red algae and cyanobacteria with great economic potential in pharmaceutical and biomedical industries as well as food and cosmetic colorants. *Gracilaria gracilis* is an alga present on the Portuguese coast, which contains phycobiliproteins, mainly phycoerythrins, as accessory pigments. The extraction of phycoerythrins from *Gracilaria gracilis* was optimized, using several extraction methods, namely maceration, ultrasounds, sonicator, and freeze/thaw. Different extraction conditions such as the concentration of phosphate buffer (C), solvent/biomass ratio (R), homogenization time (T1) and extraction time (T2) were optimized using a Response Surface Methodology. Maceration was the most efficient method (optimal conditions T1 = T2 = 10 min.; C = 0.1 M; R = 1:50) reaching values of 3.6 mg of phycoerythrin/g of algae, giving yields ~45% higher than with the other methods tested. As regards the conditions, T2 was the most influential variable in the yield while the increase of C resulted in lower values. Moreover, a stability study was performed at different storage temperatures (−20; 5; 19 and 40 °C) and pH’s (4; 6.9 and 8) for 10 days showing greater stability at −20 °C and 5 °C and at pH 6.9. Ultimately, the phycoerythrins were semi-purified by precipitation with ammonium sulfate followed by dialysis and used as colorant in pancakes. This study has shown that *G. gracilis* may be a good source of phycoerythrins for food applications.

##### **Acknowledgments** 

The authors thank FCT, Portugal, for financial support (UID/MAR/04292/2019). This work was also supported by projects SmartBioR (CENTRO-01-0145-FEDER-000018, Integrated Programme SR&TD co-funded by Centro 2020 program, Portugal 2020, European Union, through the European Regional Development Fund) and SeaWeedFeeds (16-02-01-FMP-84, Operational Programme MAR2020.

#### **Comparing the Detection of Histamine by Using GC-MS and ELISA Methods: Clues for a Faster and Direct Detection of Biogenic Amines in Seafood Products** 

EspalhaCláudia^1^FernandesJorge^1^,^2^MadeiraCarolina^3^NoronhaJoão Paulo^4^VassilenkoValentina^1^,^2^DinizMário S.^3^1Laboratory for Instrumentation, Biomedical Engineering and Radiation Physics, Physics Department, NOVA School of Science and Technology, NOVA University of Lisbon, 2896-516 Caparica, Portugal2NMT—Tecnologia, Inovação e Consultoria, S.A, Madan Parque, Caparica, Portugal, and LIBPhys—Laboratory for Instrumentation, Biomedical Engineering and Radiation Physics, Department of Physics, NOVA School of Science and Technology, NOVA University of Lisbon, 2896-516 Caparica, Portugal3UCIBIO, REQUIMTE, Department of Chemistry, NOVA School of Science and Technology, NOVA University of Lisbon, 2896-516 Caparica, Portugal4LAQV, REQUIMTE, Department of Chemistry, NOVA School of Science and Technology, NOVA University of Lisbon, 2896-516 Caparica, Portugal

The presence of biogenic amines (e.g., histamine, spermidine, putrescine) in seafood is of great concern to the food industry and consumers since it can present some risks to human health [1]. Therefore, an efficient and fast evaluation of the quality of food products is fundamental to strength the confidence of consumers. Accordingly, there is an increasing demand to decrease the allowed limits of biogenic amines in food. In addition, there is a need to develop new methodologies to detect biogenic amines allowing faster and easy detection of biogenic amines [2]. The development of new methods and new technology approaches for the assessment of seafood quality in addition to the conventional methodologies have been encouraged in the most developed countries and by the European Union [2]. In this study we present, for the first time a method for the analysis of biogenic amines (histamine and spermidine) by gas chromatography-ion mobility spectrometer (GC-IMS) device which analyzes the VOCs pattern these amines [3].

Finally, a conventional technique based on an immunoassay (ELISA) was also used to survey histamine and spermidine in seafood samples and compared with reference standards. The results are compared with results from GC-IMS and later can be uturnoversed to optimize this technique.

##### **Acknowledgments** 

This work was also supported by the Applied Molecular Biosciences Unit- UCIBIO which is financed by national funds from FCT/MCTES (UID/Multi/04378/2019) and co-financed by the ERDF under the PT2020 Partnership Agreement (POCI-01-0145-FEDER-007728) and the project 3Qs for quality—Development of new devices and techniques for seafood quality assessment (PTDC/MAR-BIO/6044/2014).

References

1. Pierina, V. Biogenic amines in raw and processed seafood. *Front. Microbiol.*
**2012**, *3*, 188.
2. Alasalvar, C.; Miyashita, K.; Shahidi, F.; Wanasundara, U. (Eds.) *Handbook of Seafood Quality, Safety, and Health Applications*; Blackwell Publishing Ltd.: Hoboken, NJ, USA, 2011; ISBN 978-1-4051-8070-2.
3. Ulanowska, A.; Ligor, M.; Amann, A.; Buszewski, B. Determination of Volatile Organic Compounds in Exhaled Breath by Ion Mobility Spectrometry. *Chem. Anal.*
**2008**, *53*, 953–965.

#### **Porphyrin Pigments in Polychaeta: Explorations on the Evolution of Haem Metabolism in Marine Eumetazoans** 

MartinsC.^1^RodrigoA. P.^1^MadeiraC.^1^D’AmbrosioM.^1^GonçalvesC.^1^ParolaA. J.^2^GrossoA. R.^1^BaptistaP. V.^1^FernandesA. R.^1^CostaP. M.^1^1UCIBIO—Applied Molecular Biosciences Unit, Departamento de Ciências da Vida, Faculdade de Ciências e Tecnologia da Universidade Nova de Lisboa, 2829-516 Caparica, Portugal2LAQV—Associate Laboratory for Green Chemistry, Departamento de Química, Faculdade de Ciências e Tecnologia da Universidade Nova de Lisboa, 2829-516 Caparica, Portugal

Porphyrins are haem-derived tetrapyrrole heterocyclic compounds that are synthetized by all major animal groups, among which biliverdin and bilirubin are well-known representatives. Porphyrins are prized targets for biotechnology as anti-oxidants and photosensitisers, depending on numerous modifications to the basic tetrapyrrole structure (such as alkylation). Naturally, coastal marine animals, owing to their colourful patterns, abundance and diversity, are promising, albeit neglected, subjects for the discovery of novel porphyrins. Our case study, the intertidal Polychaeta *Eulalia*, has been found to produce a wide variety of porphryrinoids that, altogether, contribute to its uncanny bright-green colour. Making use of RNASeq and pathway analysis, we identified five major genes that putatively code for proteins genes involved in haem metabolism: protoporphyrinogen oxidase (PPOX), uroporphyrinogen decarboxylase (UROD), uroporphyrinogen synthase (UROS), ferrochelatase (FECH) and δ-aminolevulinic acid dehydratase (ALAD). Multi-sequence Bayesian phylogenetics showed that haem metabolism is well-conserved among eumetazoans, even though the evolution of the metabolic pathway does not entirely match the animal tree-of-life across representative taxa. While the Polychaeta were more closely related to molluscs, a higher-order clade grouped the two taxa with cnidarians, echinoderms and, surprisingly, with *Limulus*. In their turn, vertebrates (humans included) and crustaceans were clustered apart, with the latter being set farthest. These relationships do not necessarily reflect those of each individual gene, meaning different rates of divergence, which could explain the wide variability of porphyrinoids in marine organisms like *Eulalia*. Our findings demonstrate that the evolution of haem metabolism reflects marine diversity, leading to a higher multiplicity of porphyrinoid pigments than anticipated.

##### **Acknowledgments** 

The authors acknowledge Fundação para a Ciência e Tecnologia (FCT) for funding project GreenTech (PTDC/MAR-BIO/0113/2014). This work was supported by UCIBIO—Applied Molecular Biosciences Unit, and LAQV—Associate Laboratory for Green Chemistry, financed by national funds from FCT (UID/Multi/04378/2019 and UID/QUI/50006/2019, respectively). FCT is also acknowledged for the grants SFRH/BD/120030/2016 to C.M. SFRH/BD/109462/2015 to A.P.R. and IF/00265/2015 to P.M.C.

#### **Disclosing New Photosensitizers for Photodynamic Therapy: Toxicity Testing of Porphyrioids from a Marine Polychaeta** 

D’AmbrosioMariaelenaGonçalvesCátiaSantosA. CatarinaCostaPedro M.UCIBIO—Applied Molecular Biosciences Unit, Faculdade de Ciências e Tecnologia, Universidade Nova de Lisboa, Campus de Caparica, 2829-516 Caparica, Portugal

Photodynamic therapy (PDT) is a frontier alternative treatment for tumour and other skin diseases. It is based on the photoactivation of photosensitizers (PSs), that, when exposed to light, produce reactive oxygen species. As therapeutic, PDT gathers attention due its selectivity when employing localized light, cost-effectiveness and reduced side-effects when compared to mainstream therapies. Porphyrinoid pigments, which are some of the best-known PSs, are naturally synthetized by many animals. Naturally-colourful organisms such as intertidal Polychaeta are thus potential sources of novel PSs. The present work focused on the marine annelid *Eulalia viridis*, which has distinctive mixtures of porphyrin-like pigments in skin (green) and proboscis (yellow). To test the worm’s light-mediated toxicity of more hydrophilic pigments, in vivo assays were carried out deploying *Daphnia pulex* as model. Daphnids (12–24 h old) were exposed to different dilutions of photoactivated or non-photoactivated crude pigments mixtures, for 24 or 48 h. The results showed differential toxicity of “yellow” and “green” pigments, with the latter yielding higher immobilization in tests with non-photo-activated pigments. The differences between the two pigments were more obvious at 24 h. The pattern of effect is, however, complex and modulated by pre-exposing pigments to light. Additionally, ex vivo assays on the gills of the mussel *Mytilus* showed similar patterns of effects on cell and DNA integrity. Altogether, the findings suggest that *Eulalia* pigments hold inherent toxicity modulated by light, which is indicative of the presence of potential PSs within pigment extracts, making this and other marine annelids relevant targets for bioprospecting novel photosensitizers.

##### **Acknowledgments** 

The authors acknowledge Fundação para a Ciência e Tecnologia (FCT) for funding project GreenTech—“Of pigments and toxins: an integrative approach to the biotechnological potential of a marine polychaete” (PTDC/MAR-BIO/0113/2014). This work was supported by UCIBIO—Applied Molecular Biosciences Unit, financed by national funds from FCT/MCTES (UID/Multi/04378/2019).

#### **Microanatomical and Histochemical Traits as Biomarkers of Toxin Secretion in Marine Invertebrates** 

GonçalvesCátia^1^CalmãoMariana^1^,^2^RodrigoAna P.^1^CostaPedro M.^1^1UCIBIO—Applied Molecular Biosciences Unit, Departamento de Ciências da Vida, Faculdade de Ciências e Tecnologia da Universidade Nova de Lisboa, 2829-516 Caparica, Portugal2Departamento de Química, Faculdade de Ciências e Tecnologia da Universidade Nova de Lisboa, 2829-516 Caparica, Portugal

Over the past few years, the oceans’ extraordinary biodiversity has been gathering focus as source of novel marine natural products, becoming one of the pillars of the new approach for the sustainable exploitation of the seas, known as Blue Biotechnology. Despite many challenges, the application of molecular biology, toxicology and pharmacology led to the development of the first commercial healthcare products from marine bioproducts, such as painkillers and cosmetics. Not surprisingly, toxins take the lead in biotechnology-oriented drug discovery, due to their ability to interfere with specific biological pathways. Tackling through such immense biodiversity to find species secreting compounds of interest is, however, a major challenge. Still, studying anatomy, physiology and biochemistry of marine animals can offer vital clues. Taking potentially venomous annelids and gastropods from the Portuguese coastline as case-studies, the present work aims at identifying key anatomical traits that act as biomarkers for toxin-secreting invertebrates. By combining microanatomy and biochemistry, it was possible to identify glands that secrete cysteine-rich proteins and peptides, commonly found in venom and poison glands. Biochemical assays to quantify the concentration of thiols in different organs of the gastropod *Nucella* sp. validated novel fluorescence-based histochemical methods applied also to a range of annelids, assisting the identification of toxin-secreting tissue and accompanying toxin-delivery structures (such as ducts and jaws), all of which hallmarks of venomous animals. The results show that cost-effective methods for the tracking of thiol-rich compounds can be used to identify new target species for drug discovery, therefore aiding systematising marine bioprospecting.

##### **Acknowledgments** 

The authors acknowledge Fundação para a Ciência e Tecnologia (FCT) for funding project GreenTech—“Of pigments and toxins: an integrative approach to the biotechnological potential of a marine polychaete” (PTDC/MAR-BIO/0113/2014). This work was supported by UCIBIO—Applied Molecular Biosciences Unit, financed by national funds from FCT/MCTES (UID/Multi/04378/2019).

#### **Extraction of Polymers and Bioactive Molecules from Fishery By-Products and Waste with Green Technology** 

McReynoldsColinClaverieMarionFernandesSusana C. M.CNRS/Univ Pau & Pays Adour/E2S UPPA, Institut des Sciences Analytiques et de Physico-Chimie pour l’Environnement et les Materiaux, UMR5254, 64600 Anglet, France

Fisheries and the processing of fishery products result in the creation of large quantities of low-value by-products and waste. By-catch and ‘trash’ fish are often discarded at sea, resulting in ecological damage and economic penalties for fishermen. Reducing the environmental impact of extractive fisheries requires a holistic approach, including creating multiple product streams to discourage waste and detrimental practices. 

These by-products contain highly valuable compounds that are yet to reach their full potential [1,2]. We are concentrating on the use of low energy, novel techniques in an effort to produce high value compounds: optimizing for the rate of biocompound extraction and reducing the steps involved in the overall process. Indeed many of these compounds are sensitive to the chemicals (strong acids and alkaline treatments) and temperatures involved in conventional methods. In this way, enzymatic extraction combined with electrochemical pretreatments will be used to target temperature- and chemically sensitive polymers, peptides, and other bioactive molecules from seaweed waste (Gelidium sesquipedale); and whole, low-value fish (Boops boops, Sarpa salpa and Silurus glanis), among other sources. Extracts will be characterized on both macro- (viscocity) and molecular levels (UV-Vis, FTIR, NMR…).

The resulting polymers and/or molecules will be used as the raw material to develop bio-inspired, multifunctional materials, such as UV-absorbing films1, tissue engineering and regenerative medecine2 and food packaging.

References

1. Fernandes, S.C.; Alonso-Varona, A.; Palomares, T.; Zubillaga, V.; Labidi, J.; Bulone, V. Exploiting Mycosporines as Natural Molecular Sunscreens for the Fabrication of UV-Absorbing Green Materials. *ACS Appl. Mater. Interfaces*
**2015**, *7*, 16558–16564.
2. Zubillaga, V.; Salaberria, A.M.; Palomares, T.; Alonso-Varona, A.; Kootala, S.; Labidi, J.; Fernandes, S.C. Chitin Nanoforms Provide Mechanical and Topological Cues to Support Growth of Human Adipose Stem Cells in Chitosan Matrices*. Biomacromolecules*
**2018**, *19*, 3000–3012.

#### **Valorization of Marine Bioresources by the Production of Green Functional Biomaterials** 

ClaverieMarion^1^McReynoldsColin^1^ThomasMartin^1^PetitpasArnaud^1^FernandesSusana C. M.^1^,^2^1CNRS/Univ Pau & Pays Adour/E2S UPPA, Institut des Sciences Analytiques et de Physico-Chimie pour l’Environnement et les Materiaux, UMR5254, 64600 Anglet, France2Uppsala University, Department of Chemistry—Angstrom Laboratory, Polymer Chemistry Division, Lagerhyddsvagen 1, Uppsala 75120, Sweden

Marine organisms are an extraordinary and under-exploited source of natural compounds (from molecular to macromolecular scale) with unique physicochemical, biological and structural properties. Inspired by hierarchical structures and functionalities observed in marine organisms, our project aims to respond to challenges in marine environment protection and human health.

Among the enormous variety of natural molecules and materials that can be extracted from marine biological resources (namely algae and animals), polysaccharides and small bioactive molecules have raised considerable interest due to their properties like biodegradability, biocompatibility, bioactivity and high availability. By processing these compounds through a global eco-friendly approach, we aim to create multifunctional materials having a low (if any) impact on the environment.

In particular, mimicking the functionality of reef fish mucus, we are developing UV-absorbing materials with potential applications in cosmetics and biomedical applications [1]. Through grafting of natural sunscreen molecules call mycosporine like amino acids (MAAs) extracted from algae onto marine biopolymers, we develop UV-absorbing materials that exclusively consist of natural compounds. Moreover, we have shown that these materials are biocompatible, photoresistant thermoresistant, and exhibit a highly efficient absorption of both UV-A and UV-B radiations. Thus, they have the potential to provide an efficient protection against both types of UV radiations and overcome several shortfalls of the current UV-protective products. Development of novel materials is still ongoing and analyses of their protective properties as well as their impact on the environment will be undertaken.

References

1. Fernandes, S.C.M.; Alonso-Varona, A.; Palomares, T.; Zubillaga, V.; Labidi, J.; Bulone, V. Exploiting Mycosporines as Natural Molecular Sunscreens for the Fabrication of UV-Absorbing Green Materials. *ACS Appl. Mater. Interfaces*
**2015**, *7*, 16558.

#### **Exopolysaccharide Production by Marine-Derived Bacteria from Estremadura Spur Sediments** 

TorresCristiana A. V.^1^FernandesMafalda^1^GaldonLorena^1^RodriguesClara^2^FreitasFilomena^1^GaudêncioSusana P.^1^ReisMaria A. M.^1^1UCIBIO, Departamento de Química, Faculdade de Ciências e Tecnologia da Universidade NOVA de Lisboa, 2829-516 Caparica, Portugal2CESAM, Departamento de Biologia, Universidade de Aveiro, 3810-193 Aveiro, Portugal

Exopolysaccharides (EPS) are biodegradable polymers that can be obtained through plants (pectin), animals (chitin) and secreted by microorganisms (xanthan, gellan gums) as a protective barrier against harmful conditions. EPS can be used in a wide range of biotechnological applications such as thickening, stabilizing and texturizing agents in the food industry, flocculating agents in the waste water treatment industry or anti-aging molecules in the cosmetic industry. Microbial EPS, such as, were isolated from terrestrial sources and successfully exploited in various industrial sectors. Marine environments are highly complex habitats that exhibit extremely variable conditions of temperature, pH, salinity and pressure. It is expected, therefore, that bacteria isolated from these environments, have different mechanisms of adaptation in relation to terrestrial ones, including, for example, the synthesis of exopolysaccharides (EPS) with distinct and diversified composition. In this bioprospecting work 30 marine-derived bacteria isolated from deep sea sediments (300–400 m) from the Estremadura Spur, Portugal, were evaluated for their capability of producing EPS through culture in bioreactor in saline culture medium containing glucose as carbon source [1]. Their ability to produce EPS under saline conditions was demonstrated in prelaminar tests. The sugars compositional analysis revealed that most of the EPS produced by the marine isolated bacteria are composed of mannose, glucose, arabinose, ribose, and was also detected the presence of rare sugar residues, namely, rhamnose, glucosamine, fucose, and uronic acids. These results encourage the biotechnological exploitation of these strains for the production of valuable innovative marine EPS with unique characteristics and properties with unique composition having growing biotechnological applications.

##### **Acknowledgments** 

This work was supported by the Applied Molecular Biosciences Unit-UCIBIO which is financed by national funds from FCT/MCTES (UID/Multi/04378/2019). FCT/MCTES through grants PTDC/QUIQUI/119116/2010, PTDC/GEO-FIQ/5162/2014 and IF/00700/2014.

References

1. Roca, C.; Lehmann, M.; Torres C.A.V.; Baptita, S.; Gaudêncio, S.P.; Freitas, F.; Reis A.M.R. Exopolysaccharide production by a marine *Pseudoalteromonas* sp. strain isolated from Madeira Archipelago ocean sediments. *New Biotechnol.*
**2016**, *33*, 460–466.

#### **Native and Modified Porphyrans as Protective Food Coatings with Antioxidant Activity** 

AdãoPedro^1^PereiraBeatriz^1^TelesMarco^1^ReboleiraJoão^1^TeixeiraCarlos M.^2^PessoaJoão Costa^2^DiasJuliana^3^BernardinoSusana^1^1MARE—Marine and Environmental Sciences Centre, ESTM, Polytechnic Institute of Leiria, 2520-641 Peniche, Portugal2Centro Química Estrutural, Departamento de Engenharia Química, Instituto Superior Técnico, Universidade de Lisboa, Av. Rovísco Pais, 1049-001 Lisboa, Portugal3Centre for Rapid and Sustainable Product Development, Polytechnic Institute of Leiria, Zona Industrial, Rua de Portugal, 2430-028 Marinha Grande, Portugal

**Figure 52:**
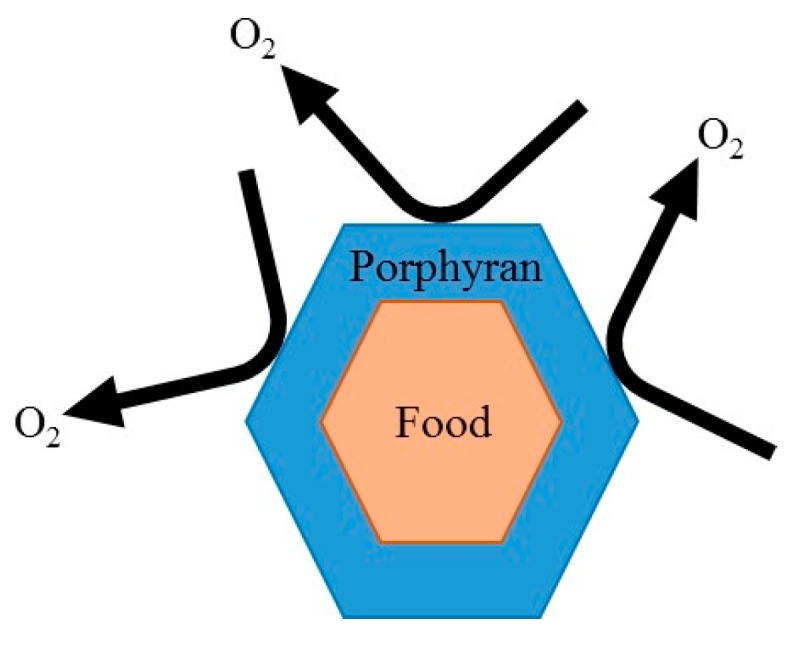
Porphyran as a protective antioxidant coating for food.

The antioxidant and antifungal properties of porphyran, a sulphated polysaccharide extracted from the *Porphyra* red algae, has been the focus of recent research efforts. In light of this, our group sought to explore the potential of porphyran as a protective coating for perishable foods (Figure 52). The objective is to provide a viable and non-toxic alternative to common antioxidants such as BHT and propyl gallate. Native porphyran extracted from *Porphyra dioica* was tested for its antioxidant properties. Modified variants of porphyran were also prepared by covalently grafting amino acids and phenols onto the polysaccharide chain, with the objective of enhancing the existing antioxidant activity. In the presence of the NaFeEDTA/H_2_O_2_/acetic acid/methyl red system, native and modified porphyrans exhibit supressing capacity of the oxidation of methyl red at a 0.2 mg/mL concentration. In the DPPH scavenging and ferric reducing power assays, the native porphyran displayed weak activities, while more pronounced activities were observed with some of the modified variants.

##### **Acknowledgments** 

The authors thank MARE—Marine and Environmental Sciences Centre, Instituto Politécnico de Leiria, (UID/MAR/04292/2019, FCT/MCTES), Centro de Química Estrutural (UID/QUI/UI0100/2019, FCT/MCTES), the IST-UL Centers of the Portuguese NMR and Mass Spectrometry Networks (REM2013, RNNMR, SAICT nº 22125), the SR&TD “SmartBioR- Smart Valorization of Endogenous Marine Biological Resources Under a Changing Climate” project (Centro-01-0145-FEDER-000018) co-funded by Centro 2020, Portugal 2020 and European Regional Development Fund (FEDER), research project “i.Film—Multifunctional Films for Intelligent and Active Applications” (nº17921, FEDER), grants 2016/iFILM/BM and Centro-01-0145-FEDER-000018-BPD4.

#### **Nutraceutial Potential of SC-CO_2_ Extracted Fatty Acids from Marine-Derived Actinomycetes** 

CoelhoCláudia T.^1^,^2^PaivaAlexandre^2^GaudêncioSusana P.^1^1UCIBIO—Applied Molecular Biosciences Unit, Blue Biotechnilogy and Biomedicine Lab., Faculdade de Ciências e Tecnologia da Universidade NOVA de Lisboa, 2829-516 Caparica, Portugal2LAQV—REQUIMTE, Faculdade de Ciências e Tecnologia da Universidade NOVA de Lisboa, 2829-516 Caparica, Portugal

Omega-3 and omega-6 fatty acids, are essencial in human diet since they are not synthetized by the human metabolism. Marine fish are the most common source of these nutrients. Fish also require an external source of essential fatty acids (EFAs), which they rely on for their cellular membrane stability and their reproductive success [1,2]. These EFAs include fatty acids from the above mentioned ω-3 and ω-6 groups, with chain lengths including C18, C20 and C22 [3]. The fishmeal used in the aquaculture feed is derived mainly from wild caught fish, limited by natural resource availability, forcing the industry to search for viable alternatives. Actinomycetes have already showed to be effective against the most commonly found pathogens in aquaculture (without decreasing the populations of beneficial bacteria) and a good substitute for fish meal as a protein source [4]. Considering the pollutant effect of the organic solvents commonly used for lipid extraction, SC-CO_2_ was successfully tested in our work as an alternative solvent. The lipidic and fatty acid content produced by two actinomycetes strains, collected off the Madeira Archipelago [5], was analyzed. The content of fatty acid methylesters (FAMEs) was estimated by gas chromatography (GC). Free fatty acid (FFA) and unsaponifiable matter (UM) quantifications were also performed [6]. Results revealed FAMEs were present in the following percentages: 19% of C16; 57–73% of C18; and 8–24% of C20/22. These results show the ability of marine-derived actinomycetes to produce fatty acids with the desired chain length (C18 enriched), indicating a great potential for the development of feeding and nutraceuticals applications.

##### **Acknowledgments** 

This work was supported by the Applied Molecular Biosciences Unit—UCIBIO which is financed by national funds from FCT/MCTES (UID/Multi/04378/2019) and by the Associate Laboratory for Green Chemistry- LAQV which is financed by national funds from FCT/MCTES (UID/QUI/50006/2019). FCT/MCTES through grants PTDC/QUIQUI/119116/2010, IF/00700/2014 and IF/01146/2015.

References

1. Kaur, N.; Chugh, V.; Gupta, A.K. Essential fatty acids as functional components of foods—A review. *J. Food Sci. Technol.*
**2014**, *51*, 2289–2303.
2. March, B.E. Essential fatty acids in fish physiology. *Can. J. Phys. Pharmac.*
**1993**, *71*, 684–689.
3. Steffens, W. Effects of variation in essential fatty acids in fish feeds on nutritive value of freshwater fish for humans. *Aquaculture*
**1997**, *151*, 97–119.
4. Garcia, B.; Campa-Córdova, A.I.; Saucedo, P.E.; Gonzaléz, M.C.; Marrero, R.M.; Mazón-Suástegui, J.M. Isolation and in vitro selection of actinomycetes strains as potential probiotics for aquaculture. *Vet. World*
**2015**, *8*, 170–176.
5. Prieto-Davo, A.; Dias, T.; Gomes, S.E.; Rodrigues, S.; Parera-Valadez, Y.; Borralho, P.M.; Pereira, F.; Rodrigues, C.M.P.; Santos-Sanches, I.; Gaudêncio, S.P. The Madeira Archipelago as a Significant Source of Marine-Derived Actinomycete Diversity with Anticancer and Antimicrobial Potential. *Front. Microbiol.*
**2016**, *7*, 1594, doi:10.3389/fmicb.2016.01594.
6. Ribeiro, H.; Marto, J.; Raposo, S.; Agapito, M.; Issac, V.; Chiari, B.G.; Lisboa, P.F.; Paiva, A.; Barreiros, S.; Simões, P. From coffee industry waste materials to skin-friendly products with improved skin fat levels. *Eur. J. Lipid Sci. Technol.*
**2013**, *115*, 330–336.

#### **Gonad Quality of Wild and Enhanced Gonads of the Sea Urchin *Paracentrotus lividus*** 

MoreiraN.^1^PomboAna^1^RaposoAndreia^1^SantosP. M^1^NevesMarta^1^FerreiraS. M. F^1^,^2^GanhãoRui^1^1MARE—Marine and Environmental Sciences Centre, ESTM, Polytechnic Institute of Leiria, 2520-641 Peniche, Portugal2CFE—Centre for Functional Ecology, Department of Life Science, University of Coimbra, Apartado 3046, 3001-401 Coimbra, Portugal

*Paracentrotus lividus* is the main echinoid consumed in Atlantic Europe. Due to the high demand of sea urchins, natural stocks have become seriously over-fished worldwide. In this context, aquaculture gains extreme attraction, both from a commercial and a preservation/restoration perspective. In echinoculture it is possible to do a gonad enhancement, whereby harvested adult sea urchins are maintained in captivity and fed with prepared diets, to improve gonad yield (GI) and the over quality. This study aimed to assess the gonad quality of sea urchin *P. lividus* from wild and enhanced individuals. For that, adults were collected in intertidal rocky pools from the coast of Peniche (Portugal) and maintained in recirculating aquaculture systems (RAS), during 12 weeks (Aqua). At the end of the trial it was conducted biometric, texture and biochemical analysis simultaneously in enhanced and wild sea urchin gonads. In terms of texture measurements, Aqua produced firm and resilient gonads, similar to wild *P. lividus* (*p* > 0.05). GI was higher in wild individuals (10%) compared to enhanced ones. In general, the proximate composition and the carotenoids content were similar between enhanced and wild sea urchins. The biochemical analysis showed that the total lipid content was much lower than the protein content. On the other hand, enhanced *P. lividus* produced gonads with more quantity of lipids compared to the correspondent wild individuals and the polyunsaturated fatty acids were the class with more representation (36.76%; *p* < 0.05*).* For all groups of sea urchins, a ω-3/ω-6 >1 (recommended value) was obtained.

##### **Acknowledgments** 

This project has the financial support of Operational Programme MAR2020 through the project 02-02-01-FEAMP-0004: Ouriceira Aqua: Aquaculture and Enhancement of Gonad Production in the Sea Urchin (*Paracentrotus lividus*) and by the Integrated Program of SR&TD “Smart Valorization of Endogenous Marine Biological Resources Under a Changing Climate” (reference Centro-01-0145-FEDER-000018), co-funded by Centro 2020 program, Portugal 2020, European Union, through the European Regional Development Fund. This study also had the support of Fundação para a Ciência e Tecnologia (FCT), through the strategic project ID/MAR/04292/2019 granted to MARE—Marine and Environmental Sciences Centre.

#### **Bioprospecting the Deep-Sea Sponge Microbiome for Novel Antibiotics** 

WilliamsS.^1^StennettH. L.^1^TiwariK.^1^BackC.^1^WillisC. L.^2^HendryK.^3^RaceP. R.^1^CurnowP.^1^1School of Biochemistry, Faculty of Life Sciences, University of Bristol, Bristol BS8 1TD, UK2School of Chemistry, Faculty of Science, University of Bristol, Britol BS8 1TS, UK3School of Earth Sciences, Faculty of Science, University of Bristol, Bristol BS8 1TS, UK

Antimicrobial resistance has emerged as a serious global threat, discovery of new antibiotics is vital to combat this threat and recent years have seen a renewed interest in microbial metabolites; in particular rare or novel species from unexplored environments as untapped sources of unique biochemistry and bioactive molecules [1]. The deep sea remains the most underexplored microbial biosphere on the planet, with 95% of the ocean >1 km deep and subject to extreme pressure, salinity and temperature; once believed to be a biological desert, the deep sea demonstrates a rich biodiversity and a vast catalogue of novel genes [2]. This project utilizes a deep-sea (0.5–4 km) sponge collection as a resource for antibiotic discovery from sponge associated bacteria, a well validated source of numerous promising antimicrobial metabolites [3]. Here I summarise progress made so far: (i) Antimicrobial activity screening of sponge associated bacteria leading to the discovery and isolation of a bioactive metabolite with a broad spectrum of activity now believed to be the quinocycline antibiotic kosinostatin [4], (ii) Hybrid de novo genome assembly of the deep-sea *Micromonspora* sp. responsible for the biosynthesis of kosinostatin, assembled to a single 6.6 Mb contig and subsequent mining of numerous silent biosynthetic gene clusters with the antiSMASH software, (iii) Characterisation of the relatively unknown deep-sea sponge microbiome through culture dependent methods, as well as 16S gene metagenomic analysis to ensure culturing efforts prioritise on samples with high degree of microbial novelty or significant proportion of actinomycetes.

References

1. Lewis, K. Platforms for antibiotic discovery. *Nat. Rev. Drug Discov.*
**2013**, *12*, 371.
2. Sunagawa, S.; Coelho, L.P.; Chaffron, S.; Kultima, J.R.; Labadie, K.; Salazar, G.; Djahanschiri, B.; Zeller, G.; Mende, D.R.; Alberti, A.; et al. Structure and function of the global ocean microbiome. *Science*
**2015**, *348*, 6237.
3. Indraningrat, A.; Smidt, H.; Sipkema, D. Bioprospecting Sponge-Associated Microbes for Antimicrobial Compounds. *Mar. Drugs*
**2016**, *14*, 87.
4. Furumai, T.; Igarashi, Y.; Higuchi, H.; Saito, N.; Oki, T. Kosinostatin, a Quinocycline Antibiotic with Antitumor Activity from *Micromonospora* sp. TP-A0468. *J. Antibiot.*
**2002**, *55*, 128–133.

## Figures and Tables

**Figure 2:**
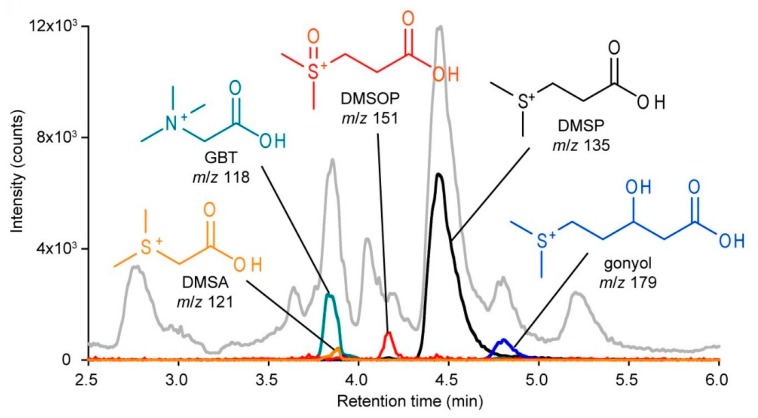
Zwitterionic metabolites from microalgae with multiple functions in the marine envirionment [1].

**Figure 3:**
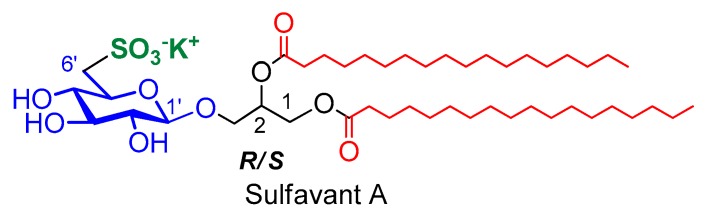
Structure of Sulfant A.

**Figure 4:**
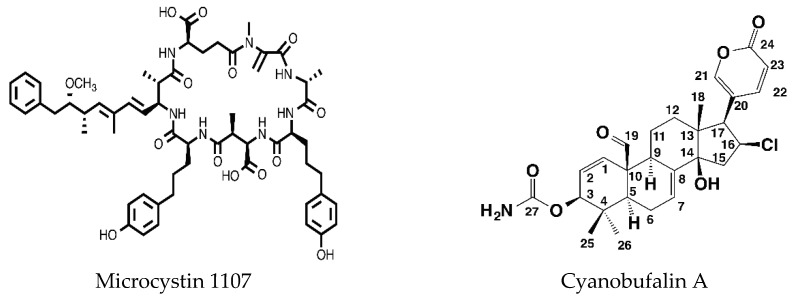
Chemical structures of microcystin 1107 and cyanobufalin A.

**Figure 5:**
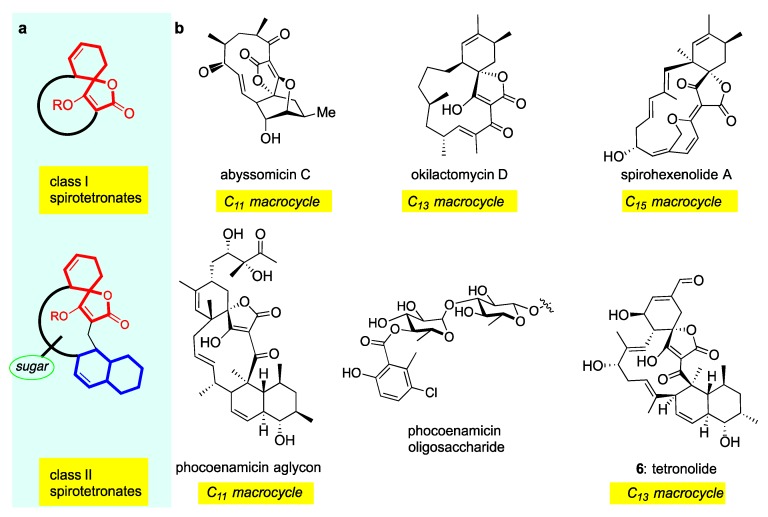
(**a**) General motif of class I and class II spirotetronates; (**b**) Representative examples of class I and class II spirotetronates.

**Figure 6:**
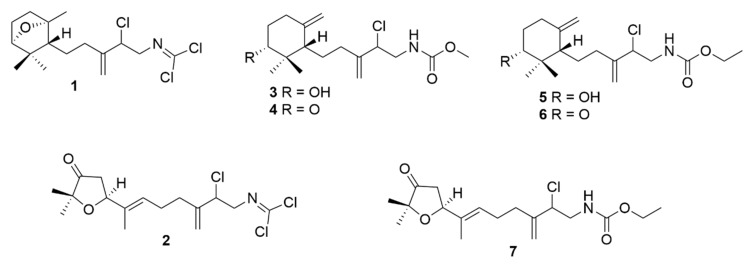
Structures of the new compounds from a distinct *P. pustulosa* clade.

**Figure 7:**
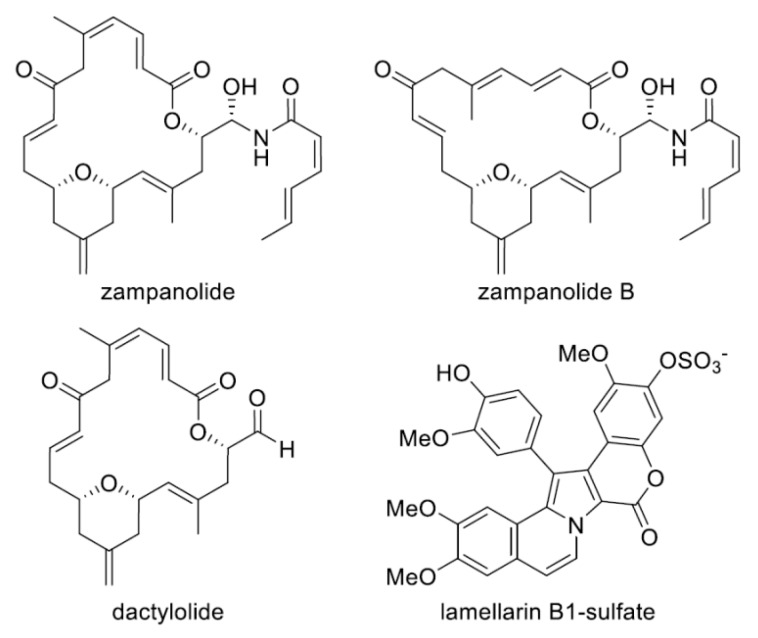
Representative macrolides from *C. mycfijiensis* and lamellarins from *D. ternerratum*.

**Figure 8:**
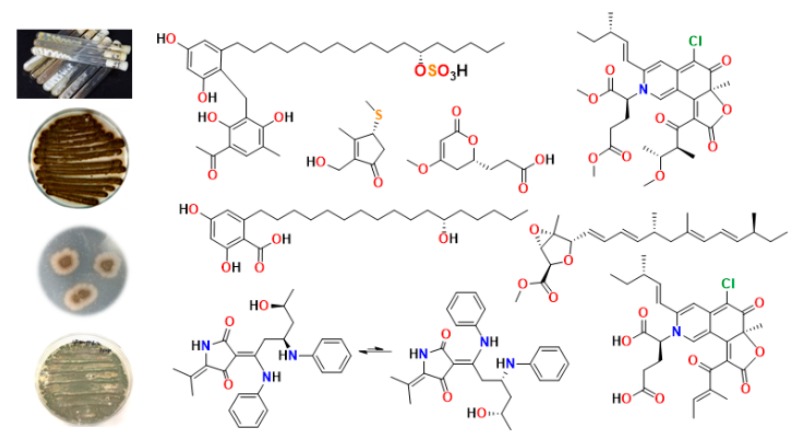
Selected deep-sea derived strains and compounds.

**Figure 9:**
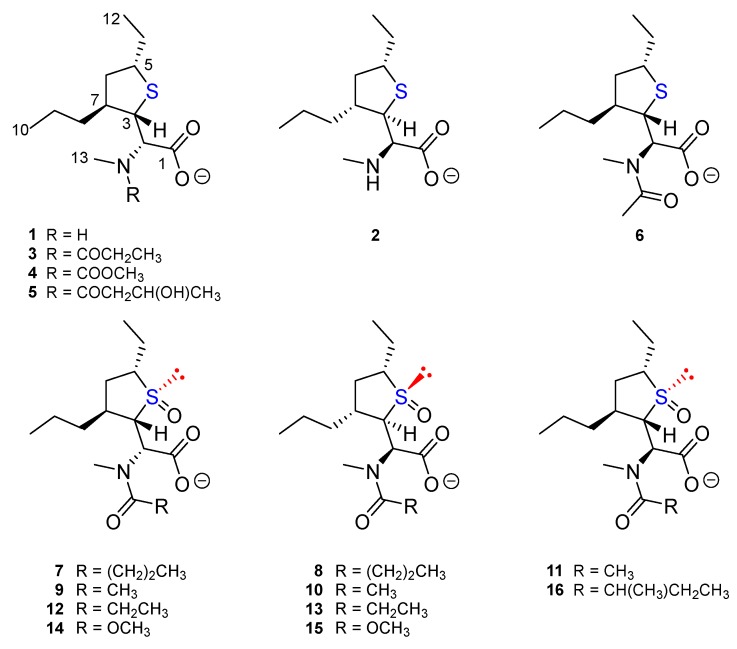
Chemical structures of nebulosins.

**Figure 10:**
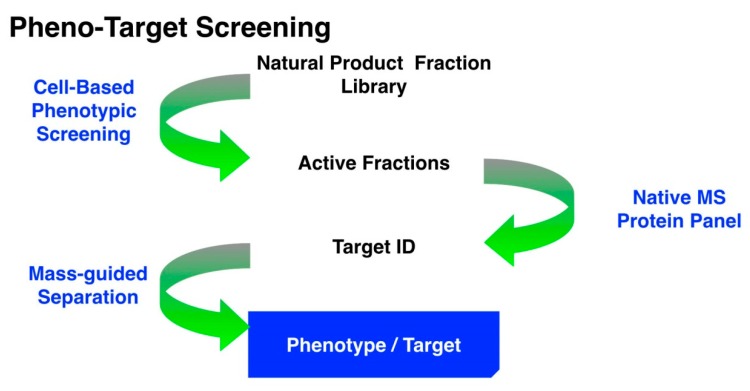
Pheno-Target Screening.

**Figure 11:**
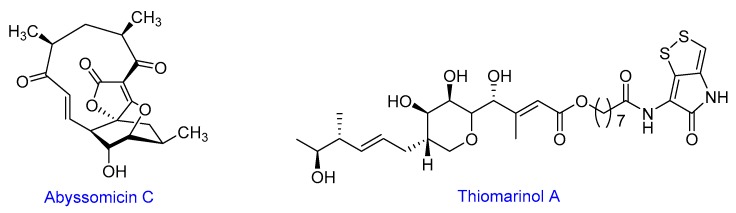
Structures of polyketide derived marine natural products**.**

**Figure 12:**
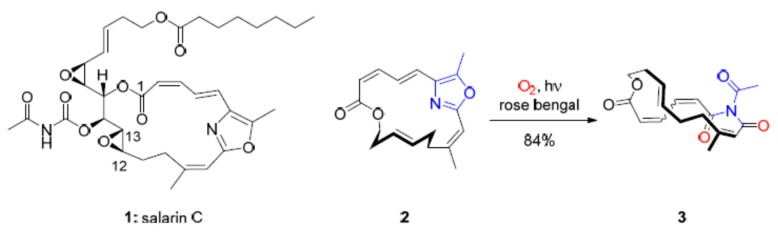
Biomimetic oxidation of the macrocyclic core of the marine natural product salarin C (**1**).

**Figure 13:**
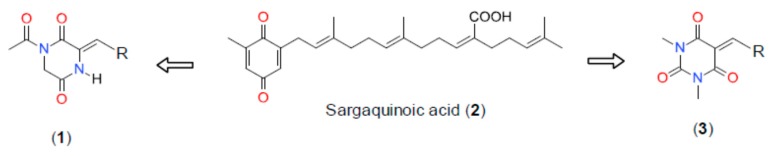
The design of sargaquinoic acid analogues.

**Figure 14:**
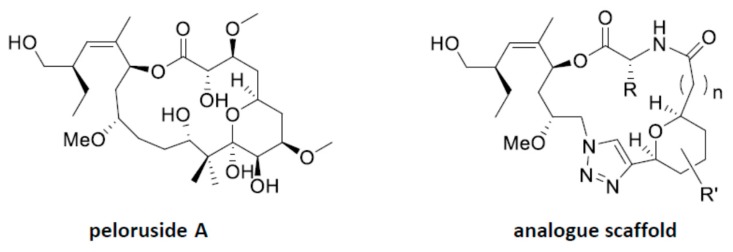
Chemical Structure of ploruside A and its analogue scaffold.

